# Identification of hub programmed cell death-related genes and immune infiltration in Crohn’s disease using bioinformatics

**DOI:** 10.3389/fgene.2024.1425062

**Published:** 2024-12-18

**Authors:** Biyao Wang, Hailing Liu, Qin Guo, Xiang Gao, Kang Chao, Qingfan Yang

**Affiliations:** ^1^ Department of Gastroenterology, The Sixth Affiliated Hospital, Sun Yat-sen University, Guangzhou, China; ^2^ Department of Small Bowel Endoscopy, The Sixth Affiliated Hospital, Sun Yat-sen University, Guangzhou, China; ^3^ Department of Pathology, The Sixth Affiliated Hospital, Sun Yat-sen University, Guangzhou, China; ^4^ Biomedical Innovation Center, The Sixth Affiliated Hospital, Sun Yat-sen University, Guangzhou, China; ^5^ Guangdong Provincial Key Laboratory of Colorectal and Pelvic Floor Diseases, The Sixth Affiliated Hospital, Sun Yat-sen University, Guangzhou, China

**Keywords:** Crohn’s disease, programmed cell death, bioinformatics, immune infiltration, functional enrichment analysis

## Abstract

**Background:**

Crohn’s disease (CD) is an immune-mediated disorder characterized by immune cell infiltration that induces persistent chronic inflammation of the gastrointestinal tract. Programmed cell death (PCD) plays a critical role in the pathogenesis of CD. This study identified vital PCD-related genes in CD based on immune infiltration using bioinformatic analysis.

**Methods:**

We obtained two CD datasets from the Gene Expression Omnibus (GEO) database and examined immune cell infiltration to investigate immune cell dysregulation in CD. PCD-related genes were retrieved from the GeneCards database. Based on the differentially expressed genes (DEGs) and PCD gene sets, PCD-related DEGs were identified. Candidate hub genes were identified using a protein-protein interaction (PPI) network, and their diagnostic effectiveness was predicted using receiver operating characteristic (ROC) curve analysis. Functional enrichment and immune infiltration analyses were used to assess the distinct roles of the hub genes. Finally, the miRWalk and ENCORI databases were used to predict which microRNAs (miRNAs) regulated the hub genes and to verify gene expression in CD colonic tissues via transcriptome sequencing.

**Results:**

A total of 335 PCD-related DEGs and 3 hub genes (*MMP1*, *SAA1*, and *PLAU*) were identified. Gene Ontology (GO) and Kyoto Encyclopedia of Genes and Genomes (KEGG) functional analyses indicated the enrichment of these genes in the immune response. Infiltration analysis of immune cells showed abundant endothelial cells, plasma cells, dendritic cells, and monocytes in the CD samples. Based on the correlation analysis, the three hub genes were positively correlated with monocytes and negatively correlated with CD8 naïve T-cells. *MMP1, SAA1*, and *PLAU* correlated with the pathogenicity of CD and had good diagnostic value for CD. The three hub genes were highly expressed in the CD tissues, as confirmed using transcriptome sequencing.

**Conclusion:**

This study identified *MMP1*, *SAA1*, and *PLAU* as hub genes involved in PCD in patients with CD. These genes regulate immune cell function and their expression levels are closely related to immune cell infiltration. These findings provide novel insights into the mechanisms underlying CD pathogenesis. The identified PCD genes and regulatory miRNAs are potential biomarkers and therapeutic targets for CD.

## 1 Introduction

Crohn’s disease (CD) is an inflammatory bowel disease affecting any part of the gastrointestinal tract (Petagna et al., 2020). The global incidence and prevalence of CD have rapidly increased (Yao et al., 2022). Patients with CD usually present with abdominal pain and severe diarrhea, accompanied by fever and serious weight loss (Yu et al., 2021). The exact etiology of CD remains largely unknown; however, genetic susceptibility, environment, microorganisms, and immune damage have been implicated as contributors that trigger inflammatory processes in the intestinal mucosa (Bunu et al., 2019; Thia et al., 2010). Macrophages, B cells, and regulatory T cells are involved in CD pathogenesis. For instance, CD14^+^ macrophages participate in CD by inducing activation-induced cell death resistance in CD4^+^ T-cells. Thus, immune-related genes are attractive targets for modulating the progression of CD (Torres et al., 2017). However, the exact mechanism by which immune cells participate in CD remains to be fully elucidated.

CD progression is associated with cell death. Programmed cell death (PCD) is a molecular regulator of cell death including apoptosis, autophagy, necrosis, ferroptosis, necroptosis, and pyroptosis (Moujalled et al., 2021; Peng et al., 2022). Ferroptosis is a recently discovered type of PCD, and its occurrence is associated with elevated iron deposition, glutathione (GSH) exhaustion, and lipid peroxidation, which aggravate intestinal cell death and sustain inflammation in CD (Xu et al., 2021; Ma et al., 2021). In addition, Gasdermin D is involved in the pathogenesis of CD by inducing lytic cell death via pyroptosis (Kayagaki et al., 2015). This demonstrates that abnormalities in PCD signaling cascades can be observed in CD and may constitute a vicious cycle that leads to the perpetuation of intestinal inflammation. However, data on key PCD-related biomarkers in CD and whether or how PCD is involved in the progression of CD based on immune infiltration remain limited.

With advancements in genomic sequencing technology, several microarray datasets have become ideal sources for quantifying immune cell infiltration in various diseases. To determine whether PCD is involved in the progression of CD, microarray data were collected from the Gene Expression Omnibus (GEO) database of intestinal tissues of patients with CD and healthy individuals. Subsequently, PCD-related differentially expressed genes (DEGs) were screened using bioinformatics methods, and immune infiltration analysis was conducted to analyze their correlation. The main functions and signaling pathways in which these genes were enriched were identified, and the microRNAs (miRNAs) regulating these genes were predicted. Our findings provide novel insights into the relationship between PCD and immune infiltration at the molecular level, and suggest potential therapeutic targets for patients with CD.

## 2 Materials and methods

### 2.1 Microarrays dataset and samples

Microarray datasets used to investigate gene expression in CD tissues were downloaded from the GEO database (http://www.ncbi.nlm.nih.gov/geo). GSE36807 was annotated by GPL570 as a Series Matrix File, including 13 colon tissue samples from patients with CD and 7 healthy controls. In contrast, GSE10616 was annotated by GPL5760 as a Series Matrix File, including 14 colon tissue samples from patients with CD and 11 healthy controls. PCD-related genes were obtained from the GeneCards database (https://www.genecards.org/) and included 14,446 apoptosis-; 7,236 autophagy-; 8,926 necrosis-; 442 ferroptosis-; 614 necroptosis-; and 254 pyroptosis-related genes.

### 2.2 Identification of DEGs associated with CD and PCD

Analysis of the gene expression matrix from the dataset was performed using the “limma” package in R (version 3.6.0) to obtain DEGs between CD and healthy samples. To ensure a certain statistical significance and avoid missing some key DEGs in the preliminary screening, we adopted the standard of |logFC| > 0.585 and *p* < 0.05 to dentify DEGs as in previous literature (Li et al., 2024; Ramalingam and Hwang, 2021). Subsequently, overlapping DEGs and PCD-related genes in CD were identified as PCD-related DEGs.

### 2.3 Analysis of immune cell infiltration

The xCell algorithm was used to quantify the infiltration of immune cells and determine the level of immune infiltration in the GSE10616 dataset. Microenvironmental and immune scores were calculated using the xCell algorithm. This method integrates the advantages of gene enrichment analysis through deconvolution and evaluates immune-cell enrichment (Aran et al., 2017). We conducted a literature review of immune-related genes (Liu et al., 2021). Spearman’s correlation analysis was used to determine the relationship between hub genes and infiltrating immune cells.

### 2.4 Functional enrichment analysis

Functional enrichment analysis of the DEGs was performed using Metascape and WebGestalt. These enrichment analysis tools have different algorithms that can perform mutual verifications. Gene set enrichment analysis (GSEA) was used to sort genes according to the degree of differential expression between the two samples (Subramanian et al., 2005). DEGs were uploaded to the GSEA website for further analysis. Gene Ontology (GO) and the Kyoto Encyclopedia of Genes and Genomes (KEGG) were used to assess the distinct roles of the subclusters. The Metascape database was used for functional and pathway enrichment analyses. To ensure rigor and reliability, the screening criteria were set to the commonly used Min overlap ≥3 and *p* ≤ 0.01, which were considered statistically significant (Zhou et al., 2019; Wang et al., 2021).

### 2.5 Protein–protein interaction network analysis

To predict protein–protein interactions (PPI), STRING, an online database that can retrieve the interactions between a group of proteins, was utilized in the PPI network analysis (Szklarczyk et al., 2017). The network was set to the default cutoff (interaction score >0.4) in the STRING online database. Genes were represented by nodes, and the interactions between the genes were indicated by edges. Molecular complex detection (MCODE) was used for clustering analysis of gene networks to identify key PPI network modules (Bandettini et al., 2012). In a module, different genes have different module scores according to which hub genes can be selected.

### 2.6 Hub gene multi-factor regulatory network

The R package “RcisTarget” (Dweep et al., 2011) was used in this study to predict transcription factors (TFs). RcisTarget bases all its calculations on the presence of specific motifs, with each motif’s normalized enrichment score (NES) determined by the total number of motifs in the database. In addition to the motifs labeled in the source data, we inferred further annotations based on motif similarity and gene sequences. To assess the overrepresentation of each motif across a gene set, the area under the curve (AUC) for each motif–motif set pair was first determined based on the recovery curve of the gene set versus motif ordering. The AUC distribution of all motifs within the gene set was used to determine the NES of each motif. The gene-motif ranking database used by RcisTarget was Rcistarget. hg19. motifdb.cisbpont.500 bp. We used miRWalk and the ENCORI database to predict targeted pivotal miRNAs and build hub gene-miRNA interaction networks (Chen et al., 2022). Only the predicted results were validated using TargetScan (http://www.targetscan.org/)and miRDB (http://www. mirdb. org/) to ensure accuracy. Finally, we constructed a ceRNA network using Cytoscape (v3.7).

### 2.7 Analysis of the diagnostic value of hub genes

For validation studies, receiver operating characteristic (ROC) curves of hub genes were plotted using R to determine the cut-off values, and the AUC was calculated to assess the clinical diagnostic significance of these hub genes.

### 2.8 Transcriptome sequencing of colonic mucosa in patients with CD

#### 2.8.1 Patient selection

Inflamed and non-inflamed samples were obtained from archived tissues of patients who underwent a colonoscopy at the Sixth Affiliated Hospital of Sun Yat-sen University, Guangzhou, China. Patients meeting the following criteria were recruited: (1) adults aged 18–60 years; (2) diagnosed with CD based on established international criteria. Exclusion criteria were: (1) recent antibiotic treatment (within 2 weeks before commencement of the study); (2) a history of CD-related surgery or concurrent conditions such as infection, short bowel syndrome, or any condition requiring surgery. Archived endoscopic biopsy samples were collected from the terminal ileum between 1 July 2022, and 1 July 2023. The absence of inflammatory lesions in non-inflamed samples was confirmed by pathological examination. Samples from 19 patients (11 inflamed and 8 non-inflamed mucosal tissues) were selected for transcriptome sequencing.

#### 2.8.2 Transcriptome sequencing

Total genes were isolated and quantified from the mucosal tissues. Determination of the concentration and purity of each sample was performed using a Qubit 4.0 fluorometer (Thermo Fisher Scientific, Waltham, MA, United States) and a NanoDrop One spectrophotometer (Thermo Fisher Scientific). RNA integrity was assessed using the Agilent 4,200 tapestation system (Agilent Technologies, Santa Clara, CA, United States). The sequencing library of each RNA sample was prepared using the ALFA-SEQ RNA Library Prep Kit according to the manufacturer’s instructions. The expression levels of genes were quantified according to the protocol provided by the manufacturer. Differential expression analyses for RNA-sequencing were performed using the limma R package. Written informed consent was obtained from all patients, and the study was approved by the Institutional Review Board of the Sixth Affiliated Hospital, Sun Yat-sen University (ID 2024ZSLYEC-625).

### 2.9 Statistical analysis methods

All statistical tests and visual analyses were performed with R (version 3.6.0). Correlation coefficient r-values >0.5 were considered to be representative of a moderate and above correlation. All statistical tests were two-sided, and statistical significance was set at *P* < 0.05.

## 3 Results

### 3.1 Identification of PCD-related DEGs in CD

**FIGURE 1:**
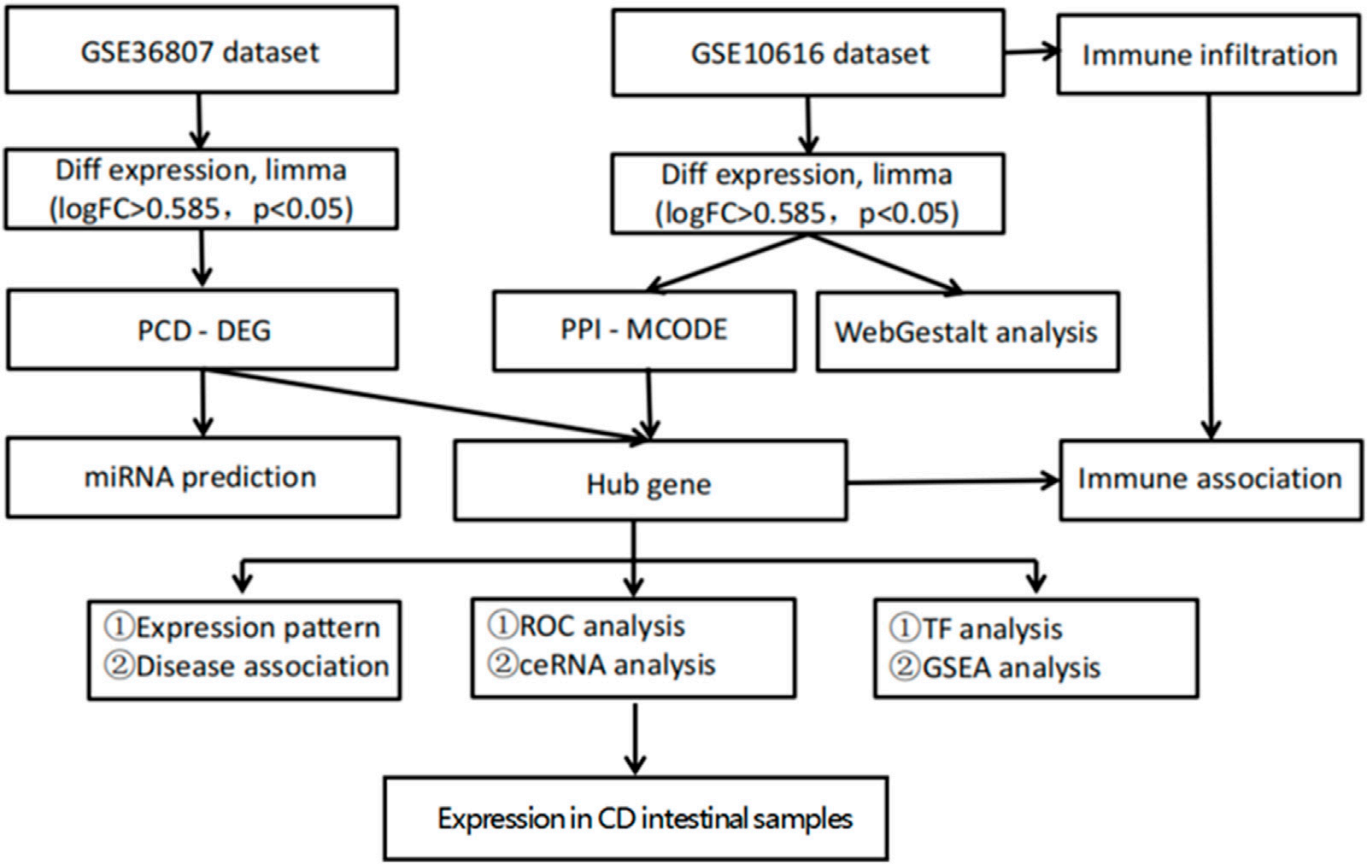
Flow chart of the bioinformatics analysis. Sequencing data from CD patients and healthy controls in GSE10616 and GSE36807 datasets were analyzed using bioinformatics to identify key programmed cell death genes in CD.

**FIGURE 2:**
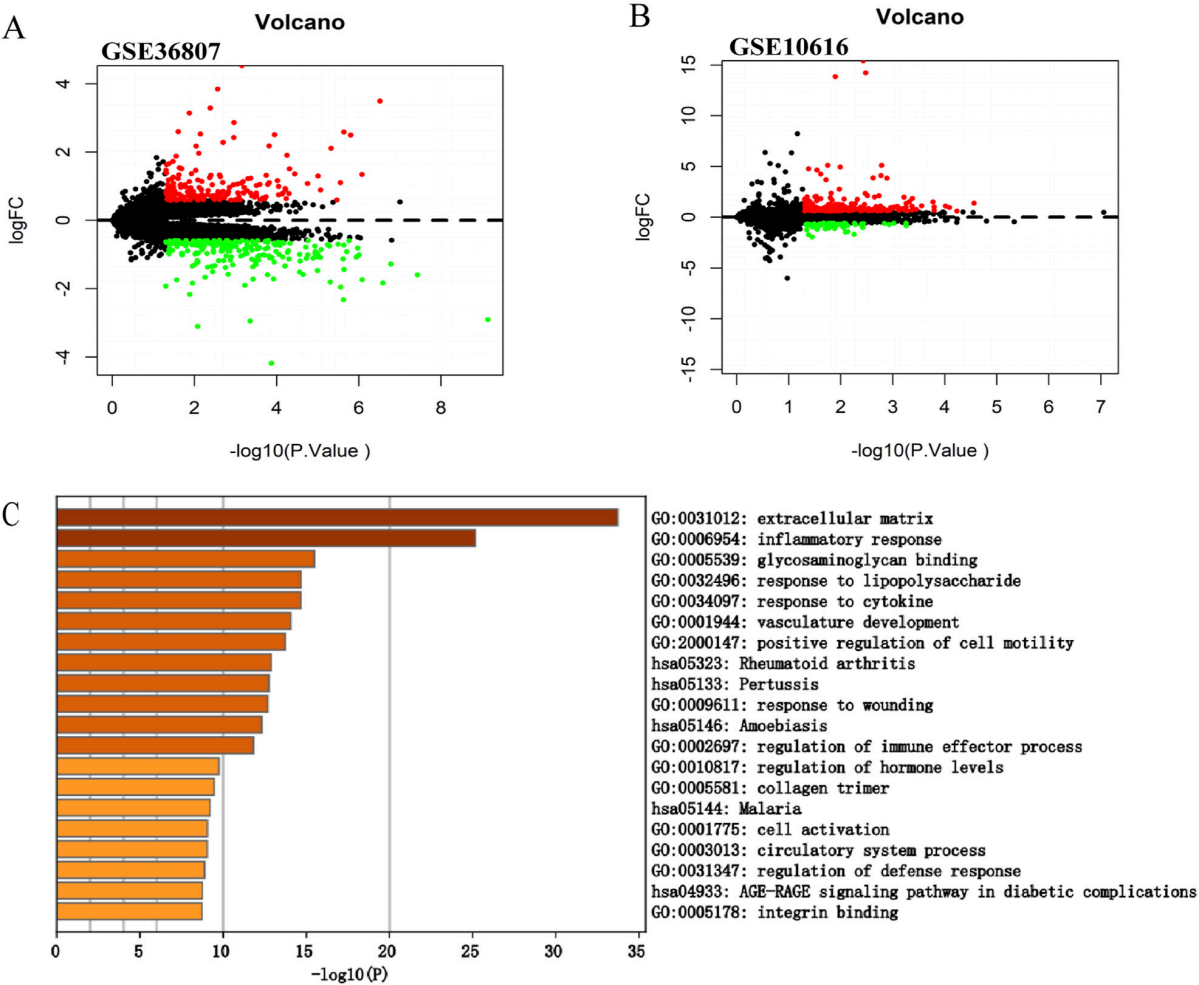
Screening of the differentially expressed genes in CD. **(A)** Volcano plot of GSE36807. **(B)** Volcano plot of GSE10616. The |logFC| > 0.585 and *p* < 0.05 were taken for filtering. Red dots represent upregulated genes, green dots represent downregulated genes, and black dots indicate genes without significant differences. **(C)** Functional enrichment analysis of differentially expressed genes in GSE10616. Metascape constructed a bar chart of 20 biological pathways, among which biological pathways with *P* < 0.01 were statistically significant. The results showed that the biological processes significantly enriched were in the extracellular matrix.

**FIGURE 3:**
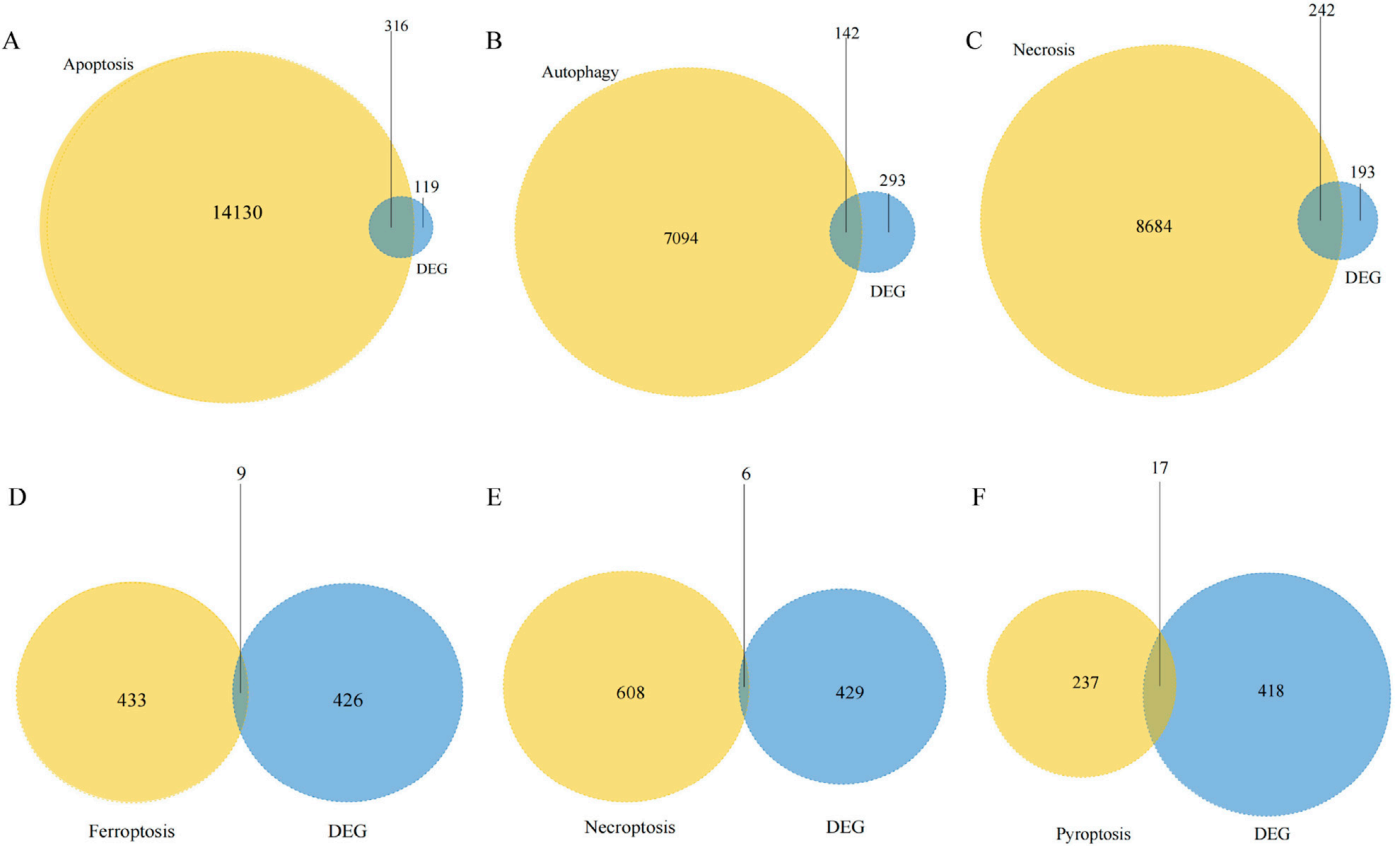
Venn diagram of differentially expressed programmed cell death (PCD) genes. **(A–F)** We intersected the death-related genes dataset with GSE36807 to identify PCD-related DEGs in CD. These included 316 apoptosis-, 142 autophagy-, 242 necrosis-, 9 ferroptosis-, 6 necroptosis-, and 17 pyroptosis-related genes.

A flowchart of the study is illustrated in Figure 1. Analysis of the GSE36807 dataset identified 435 DEGs, including 200 upregulated and 235 downregulated genes (Figure 2A). After identifying overlapping DEGs and PCD-related genes for the intersection, 335 genes were identified as PCD-related DEGs, including 316 apoptosis-, 142 autophagy-, 242 necrosis-, 9 ferroptosis-, 6 necroptosis-, and 17 pyroptosis-related genes (Figure 3). We obtained 2,608 miRNAs of PCD-related DEGs using the miRWalk database, and cross-identified them with 47 miRNAs related to CD obtained from the HMDD database. hsa-miR-107 and hsa-miR-4447 were identified as miRNAs that regulate PCD in CD. Supplementary Figure S1 shows the interactions between these two miRNAs and PCD.

### 3.2 Validation of the expression of the DEGs in CD

We selected another microarray dataset, GSE10616, to analyze the DEGs in patients with CD. In total, 272 DEGs were identified, including 59 upregulated and 213 downregulated genes (Figure 2B). The DEGs were uploaded to Metascape for further analysis (Figure 2C). The enriched pathways were mostly related to extracellular matrix, inflammatory response, and glycosaminoglycan binding. In addition, we uploaded the DEGs to WebGestalt for further analysis. The enriched pathways were mostly related to the inflammatory response, immune response, and regulation of the response to external stimuli, which identified CD as a complex inflammatory disease, with the immune response and extracellular matrix involved in its pathogenesis.

### 3.3 Construction of PPI network, analysis of modules, and identification of hub genes

**FIGURE 4:**
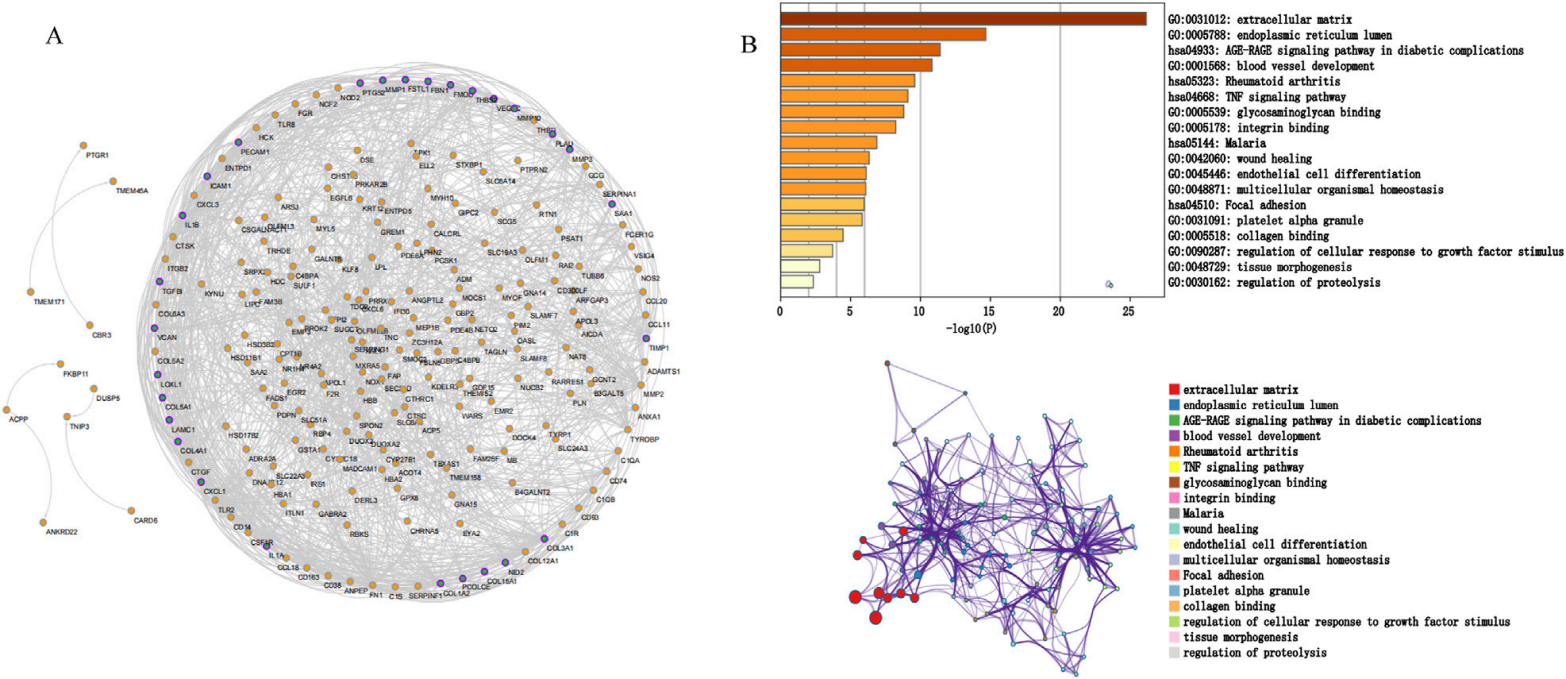
Protein interaction network. **(A)** PPI network. The nodes represent proteins, the edges represent their interaction. **(B)** Functional enrichment analysis of the top cluster. The extracellular matrix was significantly enriched.

We screened potential immune-related genes by overlapping the immune cells and DEGs in GSE10616 and used a PPI network to identify key immune-related genes (Figure 4A). Eight clusters were obtained using MCODE and the top cluster contained 28 genes. We uploaded these 28 genes to Metascape for analysis and found that the biological pathways were significantly enriched in the extracellular matrix, endoplasmic reticulum lumen, and tissue morphogenesis (Figure 4B). Finally, *MMP1*, *SAA1*, and *PLAU*, which were obtained by overlapping the PCD-related DEGs derived from GSE36807 with the top cluster, were selected as the hub genes.

### 3.4 Functional enrichment analysis

**FIGURE 5:**
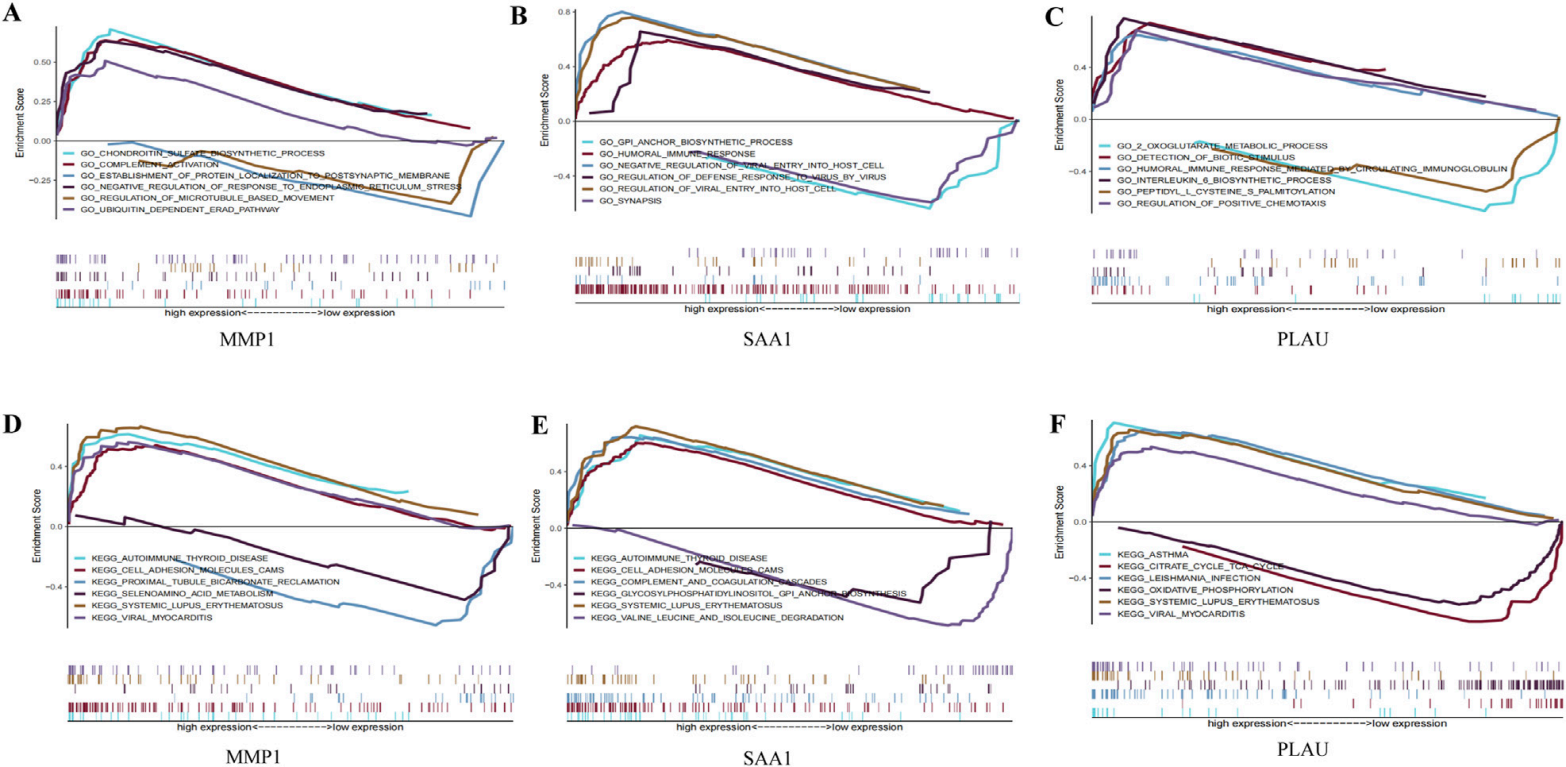
GO enrichment and KEGG pathway analysis of the hub genes. **(A–C)** A merged enrichment plot of MMP1, PLAU, and SAA1 from GO analysis including enrichment score and gene sets. **(D–F)** A merged enrichment plot of MMP1, PLAU, and SAA1 from KEGG analysis.

GO and KEGG analyses were performed on the three hub genes to provide a preliminary outlook on their biological functions (Figure 5). Through GO analysis, the pathways enriched by *MMP1* included chondroitin sulfate biosynthesis and complement activation, whereas those enriched by *SAA1* included the glycosylphosphatidylinositol anchor biosynthetic process and humoral immune response. The pathways enriched by *PLAU* included two oxoglutarate metabolic processes and detection of biotic stimuli. KEGG functional analysis revealed that *MMP1* was enriched in autoimmune thyroid disease and cell adhesion molecule cams, *SAA1* was enriched in autoimmune thyroid disease and cell adhesion molecule cams, and *PLAU* was enriched in asthma and the citrate cycle. These results suggest that these three hub genes participate in the progression of CD by influencing immune response and metabolism.

### 3.5 Immune infiltration analyses

**FIGURE 6:**
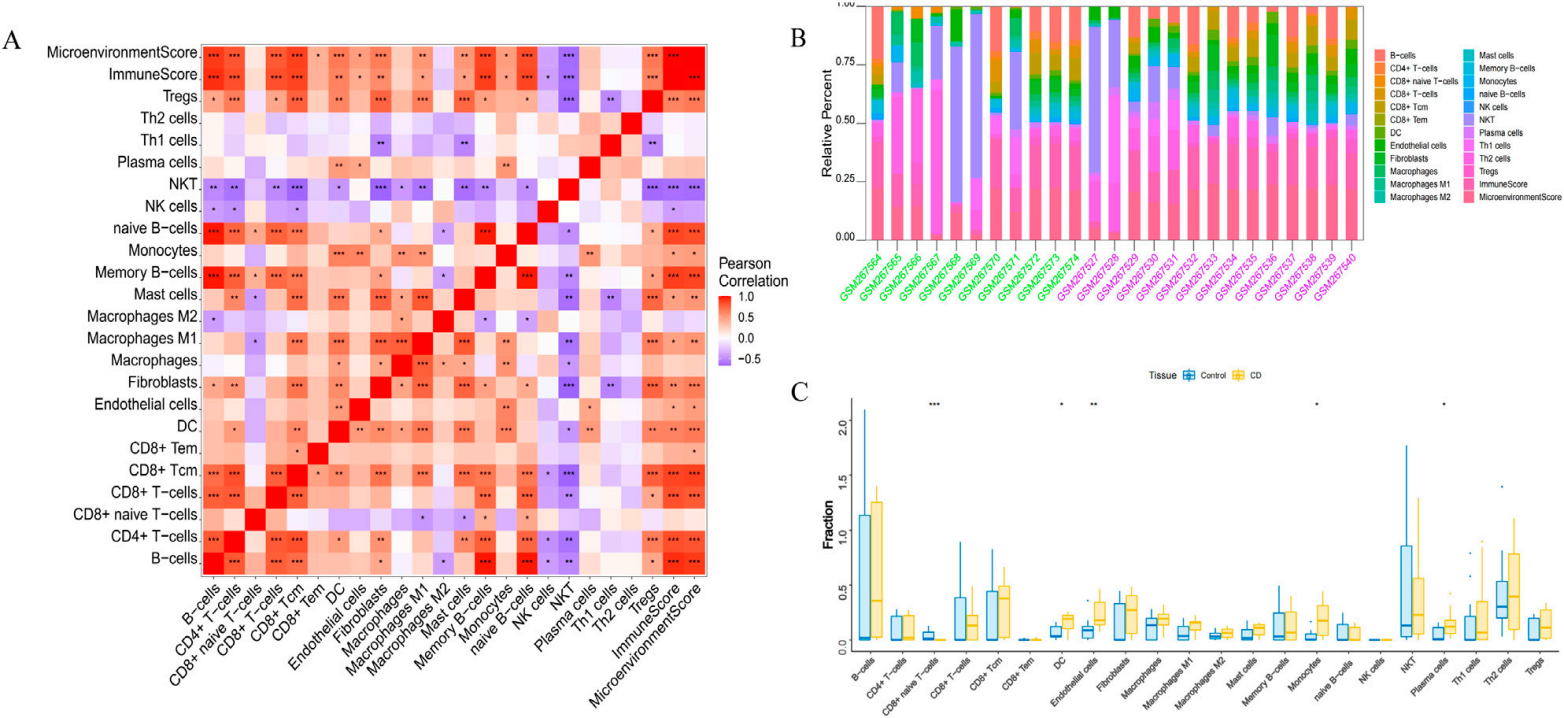
Infiltration of immune-related cells in CD and healthy control samples. **(A)** Correlation analysis of immune cells in CD. Red indicates positive correlation, purple indicates negative correlation; the higher the absolute value, the stronger the correlation between immune cells. **(B)** The box-plot diagram indicates the relative percentage of immune cells. **(C)** The diagram displays the proportion of differences between healthy and CD samples for each immune cell type. *P < 0.05, **P < 0.01, ***P < 0.001.

**FIGURE 7:**
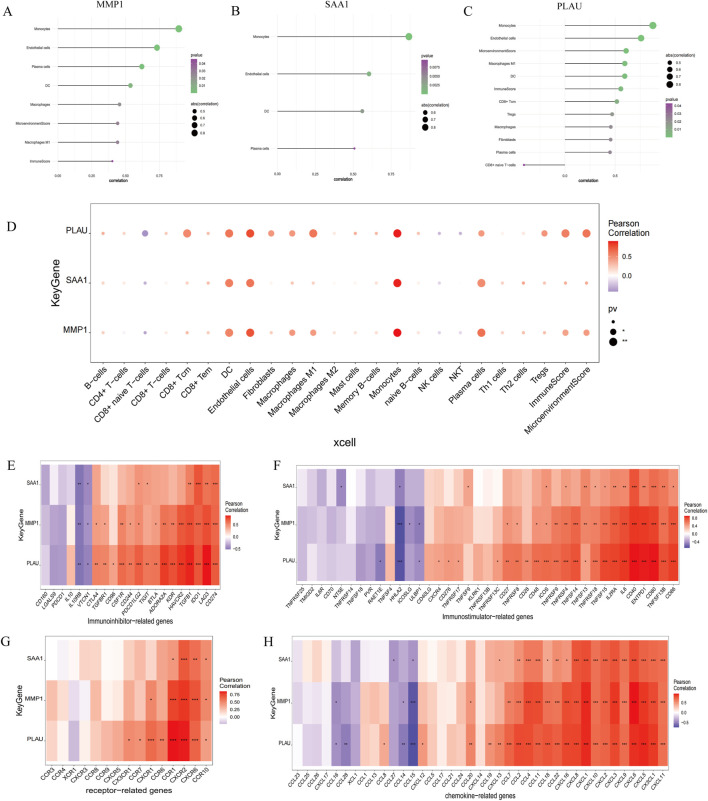
Association between the hub genes and immune cell infiltration. **(A–D)** Correlations between hub genes and the infiltration level. **(E–H)** Correlation between hub genes and immunoinhibitory factors, immunostimulatory factors, receptors, and chemokines.

Endothelial cells, plasma cells, dendritic cells (DC), and monocytes were more abundant in the intestinal tissues of patients with CD than in healthy controls. In contrast, CD8 naive T cells were less expressed in patients with CD. The interactions between immune cells are shown in Figure 6. Further exploration of the correlation between the three hub genes and immune cells indicated that *MMP1* and *SAAI* were positively correlated with monocytes, endothelial cells, plasma cells, and DC. In addition, *PLAU* was positively correlated with monocytes, endothelial cells, macrophages M1, and DC, but negatively correlated with CD8 naive T-cells (Figures 7A–D). In general, immune cells secrete immune-related cytokines. Next, we obtained correlations between the hub genes and different immune factors, including immunoinhibitory factors, immunostimulatory factors, receptors, and chemokines, from the TISIDB database. Graphs of the relationships between immune factors and CD core genes were constructed (Figures 7E–H). These findings suggest that PCD is critical for CD development through interactions between hub genes and immune-infiltrating cells.

### 3.6 Prognostic value of *MMP1*, *SAA1*, and *PLAU* in CD

**FIGURE 8:**
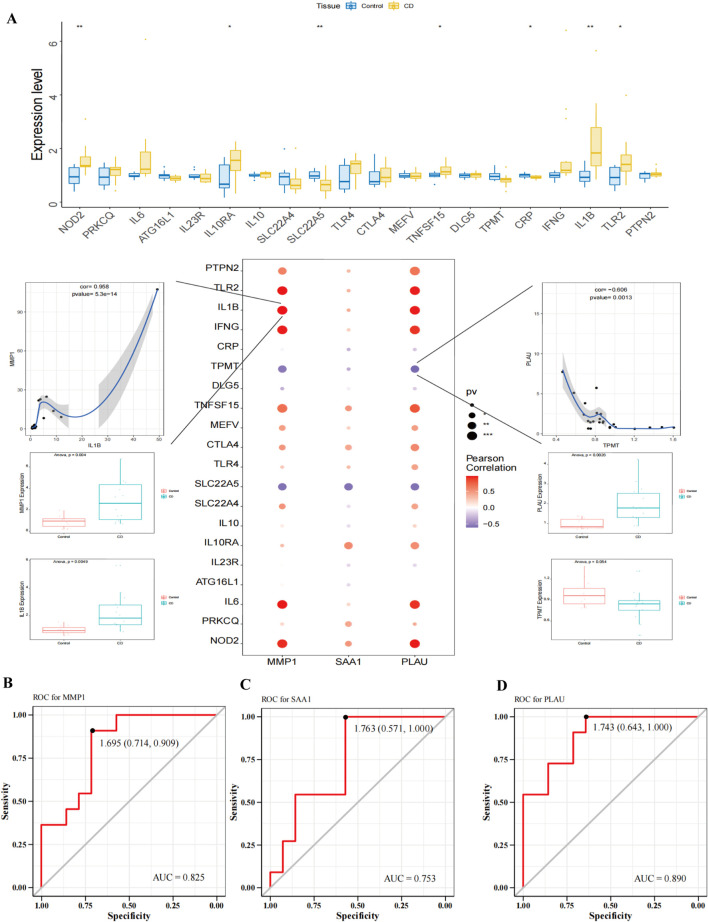
Prognostic value of MMP1, SAA1, and PLAU in CD. **(A)** The relationship between hub genes and pathogenicity genes related to CD. The results showed that the expression of hub genes significantly correlated with the CD pathogenicity-related genes. **(B–D)** The ROC curve of the MMP1, SAA1, and PLAU, respectively. The area under curve (AUC) values were >0.7, indicating that the three hub genes had good diagnostic values in CD.

We obtained 5,150 pathogenicity-related genes from the GeneCards datasets and extracted the expression levels of the top 20 genes and the relevance score of the three hub genes. Figure 8A shows the expression levels of the top 20 pathogenicity-related genes in the disease and control groups. The results showed that nucleotide-binding oligomerization domain containing 2 (NOD2), interleukin10RA (IL10RA), solute carrier 22A5 (SLC22A5), tumor necrosis factor superfamily member 15 (TNFSF15), and IL1B were highly expressed in the disease group. Moreover, the expression of hub genes significantly correlated with that of pathogenicity-related genes in CD. *MMP1* positively correlated with *IL1B* (r = 0.958, *P* = 5.3e-14), and *PLAU* negatively correlated with Thiopurine S-methyltransferase (TPMT) (r = −0.606, *P* = 0.0013). Taken together, our findings suggest the involvement of *MMP1*, *SAA1*, and *PLAU* in CD pathogenesis. To further explore the diagnostic value of these three hub genes as potential biological markers for CD, we performed an ROC curve analysis of the colon tissues of patients with CD and healthy subjects. The ROC curves of the three hub genes revealed that the AUC values of *MMP1*, *SAA1*, and *PLAU* were 0.825, 0.753, and 0.890, respectively, indicating a high diagnostic efficiency of these three genes (Figures 8B–D). Additional statistical data such as sensitivity, specificity, and optimal cut-off points are shown in Supplementary Table S1.

### 3.7 Transcription factor analysis and miRNA interaction

These three hub genes were regulated by common TFs and other shared regulatory processes. Enrichment analysis was performed on the cumulative recovery curve, motif–TF annotations, and significant gene selection results for these TFs. The results indicated that the motif with the highest NES (11.6) was cisbp_M4556, which was enriched for *MMP1* and *SAA1*. Hub-gene-enriched motifs and their associated TFs are shown in Supplementary Figure S2. Additionally, we performed reverse prediction of the three hub genes using TargetScan and miRDB, identifying 14 mRNA–miRNA relationship pairs. Based on these miRNAs; 1,110 interaction pairs (including 4 miRNAs and 955 lncRNAs) were predicted. As illustrated in Supplementary Figure S3, only miR-660–5p, miR-3173–5p, miR-664b-3p, and miR-455–3p had upstream lncRNAs and demonstrated higher reliability, all of which targeted PLAU.

### 3.8 Validation of potential biomarkers using transcriptome sequencing

**FIGURE 9:**
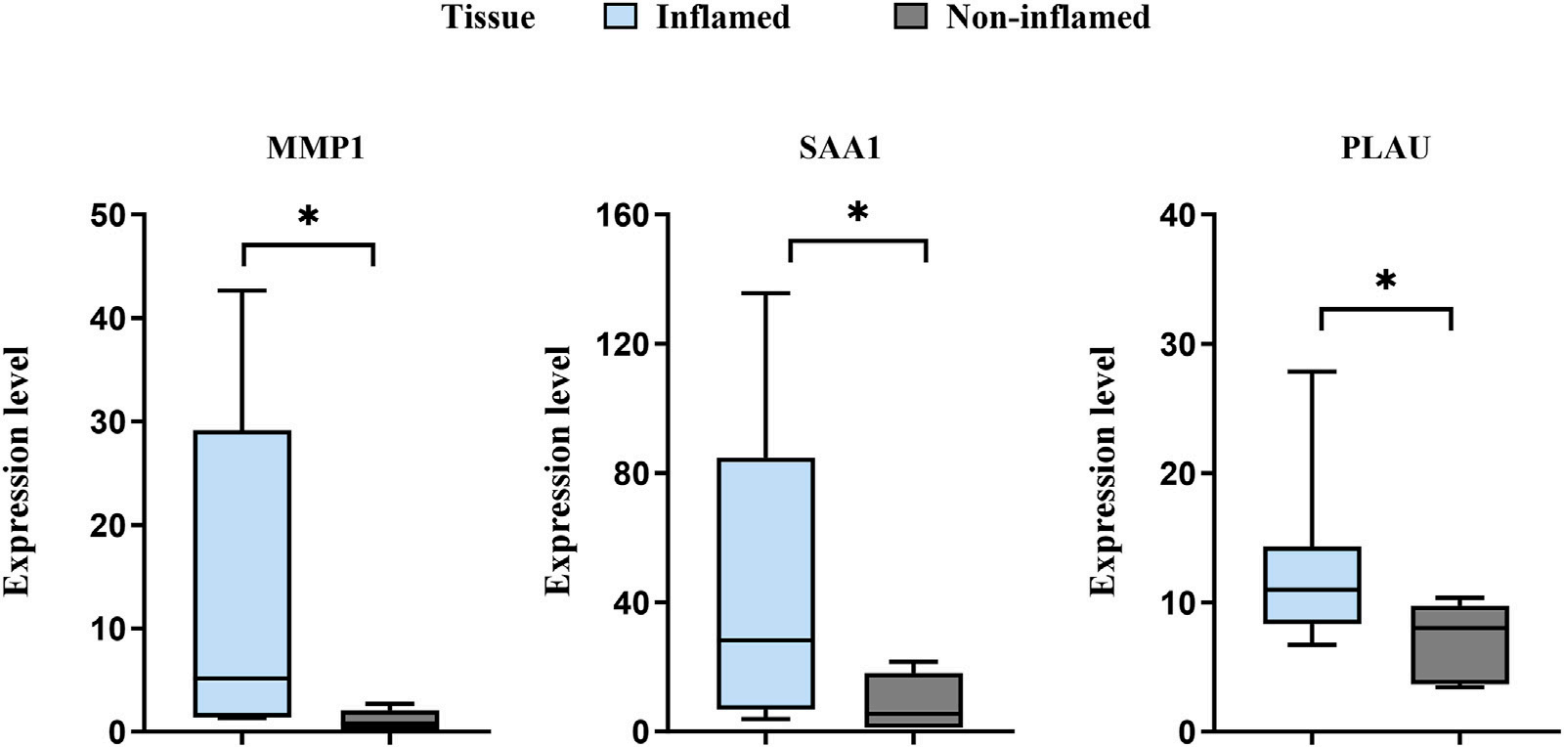
Sequencing of the CD mucosal transcriptome results showed that the expression levels of MMP1, SAA1, and PLAU were higher in CD-inflamed colon mucosa than in non-inflamed controls. *P < 0.05.

The expression levels of *MMP1*, *SAA1*, and *PLAU* were upregulated in CD samples from both GSE10616 and GSE36807 datasets. Transcriptome sequencing of the mucosal tissues of patients with CD was performed to validate the differential expression of hub genes in the inflamed mucosal tissues and non-inflamed control groups. The data demonstrated that the expression levels of *MMP1, SAA1,* and *PLAU* were upregulated in the inflamed intestinal mucosa of patients with CD, validating our results from bioinformatics analyses (*P* < 0.05) (Figure 9). Taken together, our results suggest that these hub genes are involved in CD pathogenesis.

## 4 Discussion

The association between PCD and CD progression has been well documented (Zhou and Bao, 2020; Günther et al., 2013). CD tissue often exhibits high levels of immune cell infiltration. Exploring the mechanisms and functions of PCD-related genes and the regulation of immune cell infiltration will shed some light on the development of CD. In this study, we identified 272 DEGs from the dataset GSE10616, including 59 upregulated and 213 downregulated genes. The pathway enrichment analyses of DEGs performed using Metascape and WebGestalt indicated that these genes were mainly involved in extracellular matrix, inflammatory, and immune responses. We screened the potential immune-related genes by overlapping the immune cells and DEGs and used a PPI network to identify the key immune-related genes. *MMP1*, *SAA1*, and *PLAU* from the intersection of the top 28 key immune-related genes and PCD-related DEGs in GSE36807 were identified as hub genes.

GO and KEGG analyses showed that the three hub genes were mainly associated with immune-related signaling pathways, and the function of hub genes was significantly associated with immune cells in CD, suggesting further exploration into the correlation between hub genes and immune infiltration. As *MMP1*, *SAA1*, and *PLAU* are key PCD-related genes overexpressed in CD tissues, we assessed the diagnostic value of these three genes. As expected, these three hub genes had a high diagnostic value for CD.

Matrix metalloproteinases (MMPs) are zinc-dependent endopeptidases that play crucial roles in cellular regeneration, programmed death, and other essential functions (von Lampe et al., 2000; Mei et al., 2023). Previous studies have shown that MMPs, including *MMP2* and *MMP9,* are important in CD pathogenesis and are diagnostic markers for the disease (Jakubowska et al., 2016). *MMP3* and *MMP9* also modulate cellular autophagy, apoptosis, and extracellular matrix metabolism in human intervertebral disc cells (Wu et al., 2017). *MMP1* regulates the balance of extracellular matrix components between synthesis and breakdown, which can lead to progressive tissue destruction or excessive collagen deposition, resulting in ulcers, fistulas, and fibrosis in CD (Sosna et al., 2023). However, the mechanism by which *MMP1* regulates CD pathogenesis via PCD remains unclear. Our results suggest that *MMP1* may influence necrosis, apoptosis, and autophagy, thereby affecting CD. Previous studies align with our findings, as *MMP1* has been implicated in lung cancer progression through PCD modulation (Wang et al., 2024; Rebelo et al., 2018). In terms of immune cell regulation, studies have found significant differences in *MMP1* expression and immune cell infiltration levels in hepatocellular carcinoma (HCC), where high *MMP1* expression positively correlates with DC, Th1/2 cells, endothelial cells, and monocytes (Dai et al., 2022). Thiesen et al. also reported that classical monocytes migrate effectively toward C-C motif chemokine ligand 2 (CCL2) and release high levels of *MMP1* when stimulated with immune complexes or lipopolysaccharides (LPS), suggesting that *MMP1* is closely related to monocyte function in CD (Thiesen et al., 2014). Similarly, we found a close association between *MMP1* and immune cell infiltration by monocytes, endothelial cells, plasma cells, and DC. We speculated that *MMP1* may contribute to CD progression by affecting PCD in DC, endothelial cells, and monocytes. Furthermore, *MMP1* was enriched in the tumor necrosis factor-alpha (TNF-α) signaling pathway, a key pathway in intestinal inflammation, suggesting that TNF-α signaling may be a potential target of *MMP1* in CD. The expression level of *MMP1* is considered to be a predictor of the effectiveness of anti-TNF treatment, as shown by several studies (Yu et al., 2021), which is similar to our results. In addition, endoscopy healing index (EHI) is a composite index for evaluating CD which contains a variety of serum biomarkers, including *MMP1* (D'Haens et al., 2020). Monitoring the expression of *MMP1* may provide information about tissue remodeling, supplement the lack of inflammatory markers, and improve the accuracy of disease activity and severity assessment. Inhibitors targeting *MMP1* may become a new strategy for the treatment of CD (Altadill et al., 2021).

*SAA1* encodes serum amyloid A (SAA), a predictive indicator of IBD activity (Ye and Sun, 2015; Duarte-Delgado et al., 2018). Our findings revealed that *SAA1* is a key PCD-related gene in CD and is associated with necrosis, apoptosis, and autophagy. However, the mechanism by which *SAA1* regulates CD pathogenesis via PCD remains unclear. *SAA1* is thought to enhance high-density lipoprotein oxidation, activate immune cells such as monocytes to produce IL-1 and IL-23, stimulate protective IL-22-producing neutrophils to support the epithelial barrier, and form immune complexes (Zhou et al., 2017). Recent studies have suggested that *SAA1* is involved in the immune microenvironment of clear cell renal cell carcinoma (ccRCC). SAA1 is highly expressed in ccRCC and significantly correlated with mast cells (Xu et al., 2023). We found a positive association between *SAA1* expression and monocytes, suggesting that *SAA1* may affect CD by regulating the PCD of monocytes; however, further studies are warranted to explore this mechanism. Previous study reported the sensitivity of *SAA1* in the diagnosis of inflammatory infection is higher than that of C-reactive protein (CRP). Moreover, *SAA1* has a certain correlation with the clinical activity and endoscopic activity of CD (Stute et al., 2024). Combining *SAA1* with fecal calprotectin (FCP) may improve the sensitivity and specificity of CD diagnosis (Bahaa et al., 2024). Therefore, *SAA1* may help to monitor the disease activity of patients with CD and reduce the dependence on invasive examination.

*PLAU* encodes the urokinase plasminogen activator (uPA) (Li et al., 2021). A recent meta-analysis of 15 IBD datasets showed that uPA expression was linked to IBD inflammatory states (Cheng et al., 2022). Previous studies have also demonstrated the coordinated upregulation of uPAR and its ligand uPA in CD during intestinal epithelial barrier breakdown, where disrupted tight junctions and increased cell death suggest that the uPA-uPAR complex may affect the mucosal barrier by mediating PCD in target cells, identifying it as a potential CD target. Moreover, the high expression of uPA-uPAR in anti-TNF-resistant patients implies that the uPA-uPAR pathway may play an important role in these patients (Hamada et al., 2024; Cheng et al., 2022). Therefore, small molecule inhibitors or antibodies targeting uPA binding sites on uPAR may be beneficial to the development of CD therapy. In our study, we confirmed that *PLAU* was upregulated during intestinal mucosal inflammation in CD and associated with apoptosis and autophagy. Therefore, future studies using freshly prepared tissue explants from patients will be helpful to further investigate the role of *PLAU* in CD. *PLAU* is associated with six types of infiltrating immune cells in hepatocellular carcinoma (LIHC), including CD4^+^ T cells, neutrophils, CD8^+^ T cells, macrophages, B cells, and dendritic cells (Wu et al., 2023). In our study, *PLAU* expression negatively correlated with naïve CD8^+^ T cells, further implicating the possibility that *PLAU* may affect CD by regulating the PCD of naïve CD8^+^ T cells. We also observed a high AUC value of the ROC curves of *PLAU* in diagnosing CD. Therefore, these results reveal that *PLAU* may complement existing inflammatory markers (such as CRP and FCP) and provide additional information about the disease microenvironment and immune status. In addition, azathioprine (AZA) is a commonly used drug to control CD, and our results indicated that *PLAU* was negatively correlated with TPMT, an enzyme involved in azathioprine (AZA) metabolism, and an important risk predictor for AZA-induced leukopenia (Kakuta et al., 2017). These findings suggest that the potential application of *PLAU* in CD may involve its effects on programmed cell death, immune microenvironment and thiopurine metabolism.

In the present study, we constructed an miRNA-gene network to illustrate the internal association between hub genes and miRNAs. Several miRNAs are involved in the regulation of hub genes, and may be considered crucial regulators of CD pathogenesis. In this study, we identified 14 miRNAs that target hub genes. miR-660–5p, miR-3173–5, miR-664b-3p, and miR-455–3p were highly correlated with hub genes. Further studies are required to determine the specific molecular mechanisms involved.

Finally, we performed transcriptome sequencing of CD colonic mucosa to validate hub gene expression. The expression of hub genes was considerably higher in inflamed tissues than in non-inflamed tissues. Our research, combined with the results of bioinformatics analysis and transcriptome sequencing, showed significant differences in hub genes in samples from patients with CD. We found that the expression of hub genes was significantly correlated with the expression levels of CD pathogenicity-related factors, such as IL1B, IL6, and nucleotide-binding oligomerization domain containing 2 (NOD2) and had great diagnostic power for CD (all AUC >0.7). This suggests that *MMP1*, *SAA1*, and *PLAU* are potential biomarkers for their involvement in CD pathogenesis.

This study has some limitations. First, although we performed expression validation in the intestinal samples of patients with CD via transcriptome sequencing, the sample size was small. Therefore, the correlation between hub genes and clinical disease activity, disease phenotype, mucosal healing, histological remission and so on could not be further analyzed, limiting the extrapolation of our results and the guiding value of clinical application. Future studies should include larger sample sizes to further identify the expression of hub genes in CD, and comprehensive clinical information should be obtained to analyze the relationship between hub genes and clinical characteristics. In addition, *MMP1, SAA1*, and *PLAU* play a role in normal physiological processes and pathological states and the expression of these genes may be affected by a variety of factors, including the severity of inflammation, the location and scope of lesions, limiting the accuracy of MMP1, SAA1 and PLAU as a single biomarker. Different individuals may respond differently to hub genes, which affects the universality of hub genes as a biomarker and therapeutic target. Finally, the hub genes, pathways, and immune characteristics identified using current bioinformatics analyses should be further validated to better determine whether the hub genes regulate the pathogenesis of CD through PCD.

## 5 Conclusion

In this study, *MMP1*, *SAA1*, and *PLAU* were identified as key PCD genes in CD. Correlation analysis of immune infiltration implied that *MMP1*, *SAA1*, and *PLAU* may participate in CD pathogenesis by regulating immune cell function. The miR-660–5p, miR-3173–5, miR-664b-3p, and miR-455–3p may also be associated with CD. These findings provide novel insights into the mechanisms underlying CD and identify key PCD-related genes and miRNAs as potential biomarkers and therapeutic targets for CD. However, the relationship between cell death-related genes and immune cell infiltration warrants further investigation. The mechanisms of hub genes and immune infiltration in CD diagnosis require further investigation.

## Data Availability

The original contributions presented in the study are included in the article/Supplementary Material, further inquiries can be directed to the corresponding author.
