# Supplementary material for: Identification of hub programmed cell death-related genes and immune infiltration in Crohn’s disease using bioinformatics
Source: Front Genet. 2024 Dec 18;15:1425062. doi: 10.3389/fgene.2024.1425062 (PMC11688285; doi:10.3389/fgene.2024.1425062)
Supplement: Supplementary file 1 [file DataSheet3.zip › Input data and script2/GSEA analysis/MMP1/plot_go/1.GSEA GO.pdf]

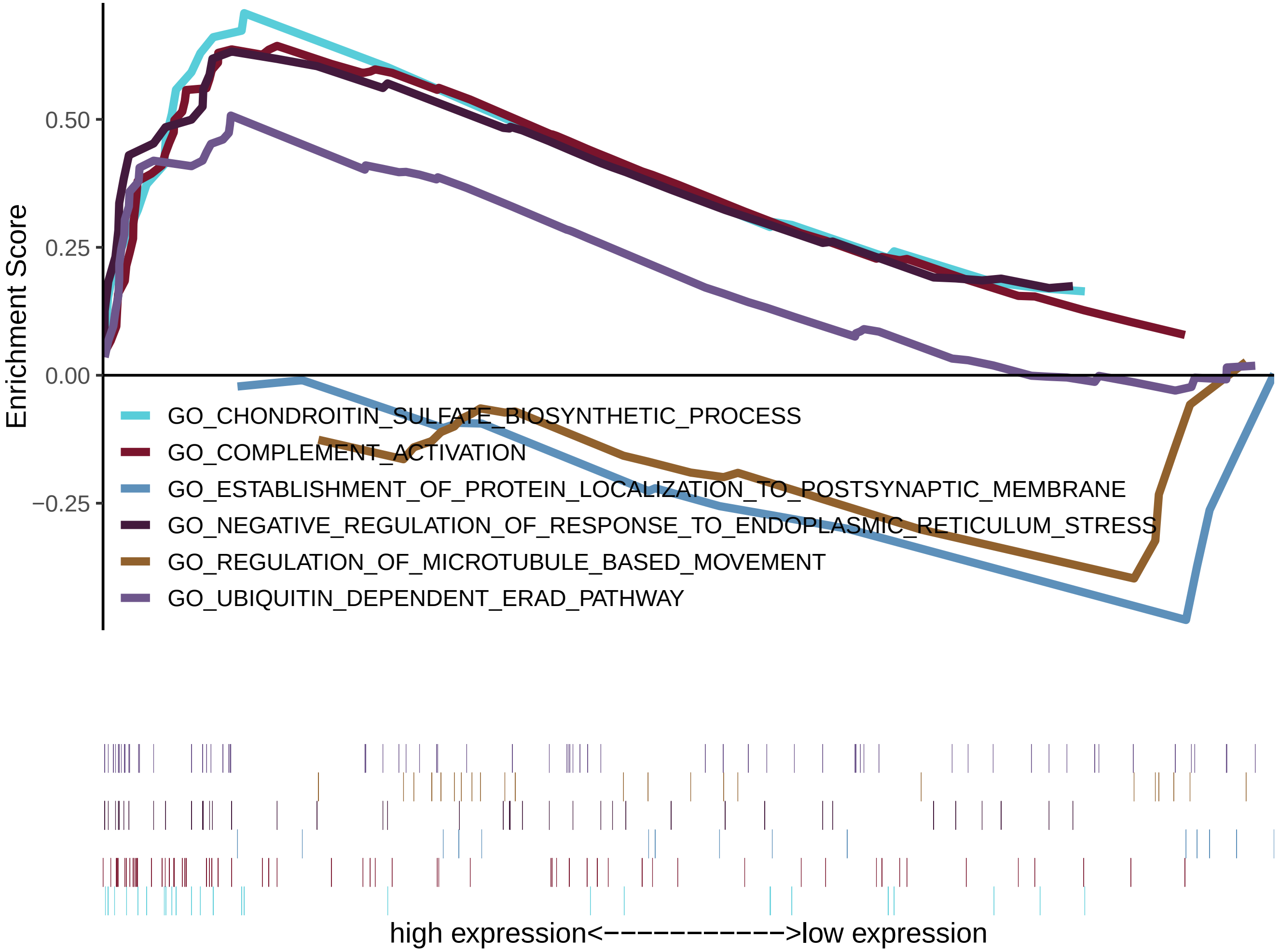
Enrichment Score
0.50
0.25
0.00
−0.25
GO_CHONDROITIN_SULFATE_BIOSYNTHETIC_PROCESS
GO_COMPLEMENT_ACTIVATION
GO_ESTABLISHMENT_OF_PROTEIN_LOCALIZATION_TO_POSTSYNAPTIC_MEMBRANE
GO_NEGATIVE_REGULATION_OF_RESPONSE_TO_ENDOPLASMIC_RETICULUM_STRESS
GO_REGULATION_OF_MICROTUBULE_BASED_MOVEMENT
GO_UBIQUITIN_DEPENDENT_ERAD_PATHWAY
high expression<−−−−−−−−−−−>low expression
