# Supplementary figures and images for: Identification of hub programmed cell death-related genes and immune infiltration in Crohn’s disease using bioinformatics (part 2 of 2)
Source: Front Genet. 2024 Dec 18;15:1425062. doi: 10.3389/fgene.2024.1425062 (PMC11688285; doi:10.3389/fgene.2024.1425062)

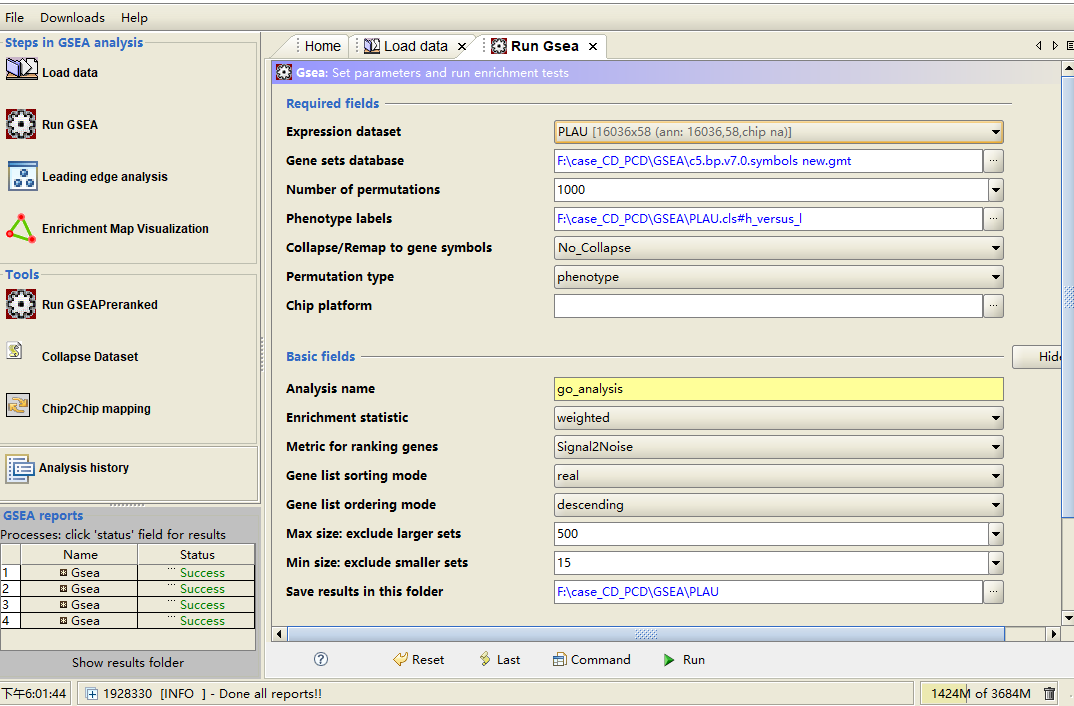

Supplement: Supplementary file 1 [file DataSheet3.zip › Input data and script2/GSEA analysis/PLAU/σ▒Åσ╣òμê¬σ¢╛ 2022-04-06 180233.png]

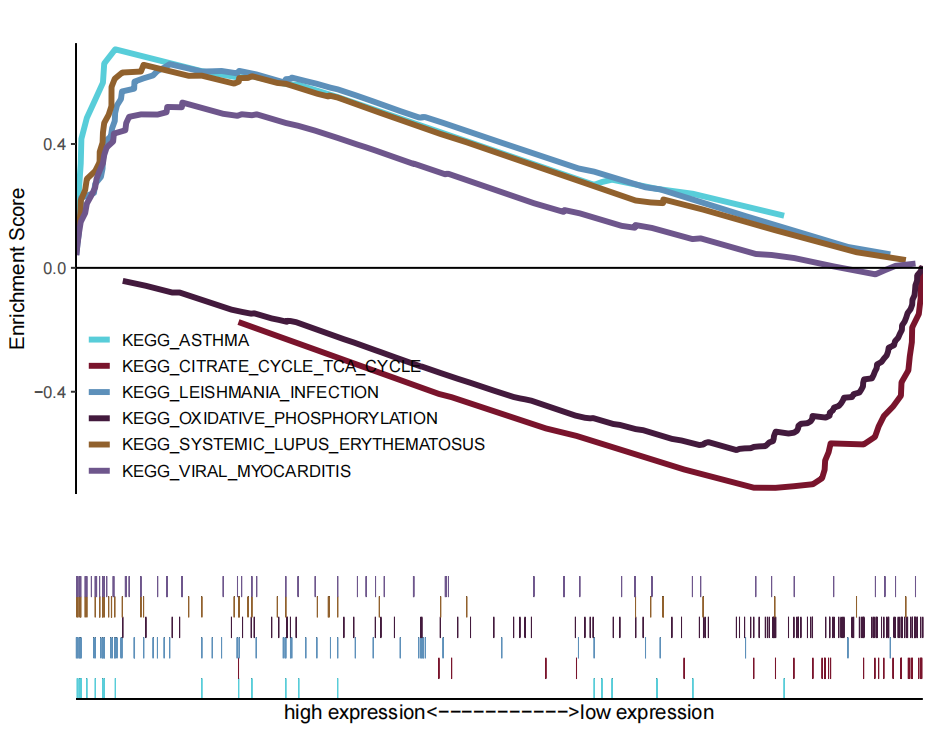

Supplement: Supplementary file 1 [file DataSheet3.zip › Input data and script2/GSEA analysis/PLAU/2.GSEA KEGG_00.png]

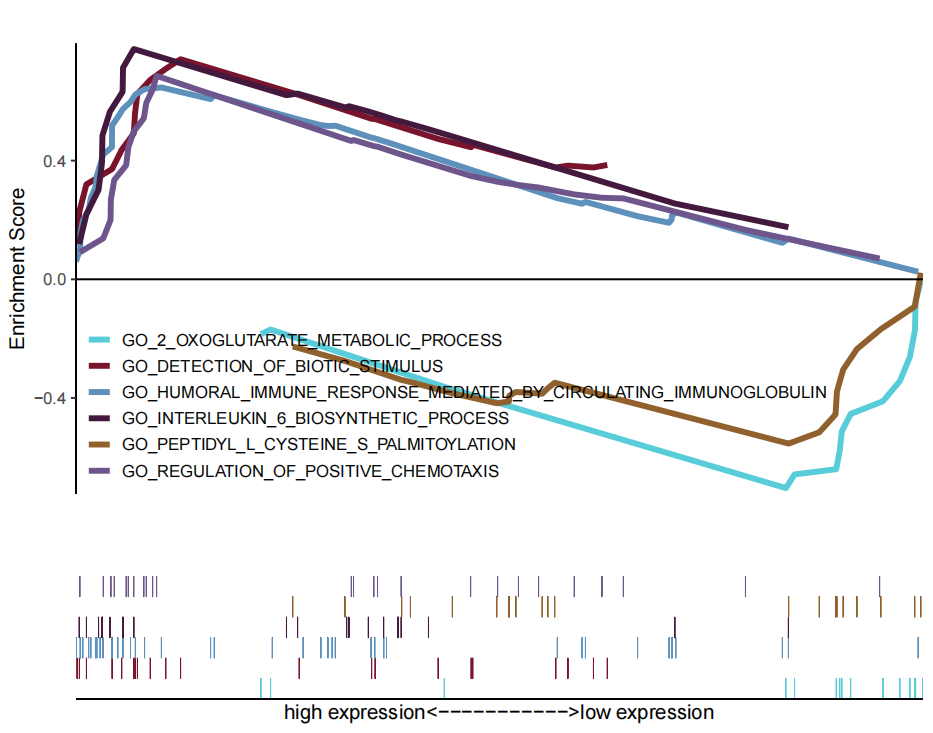

Supplement: Supplementary file 1 [file DataSheet3.zip › Input data and script2/GSEA analysis/PLAU/1.GSEA GO_00.png]

Enrichment Score

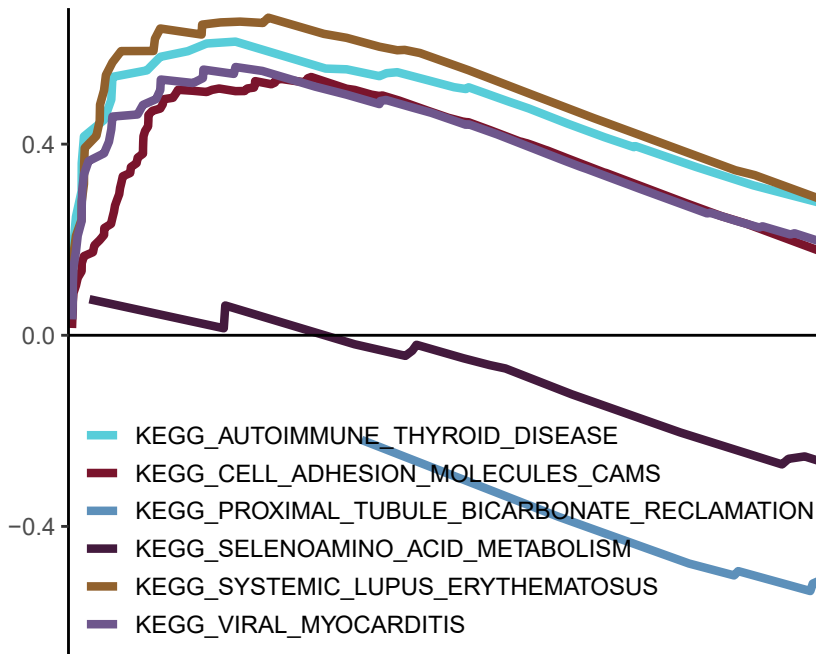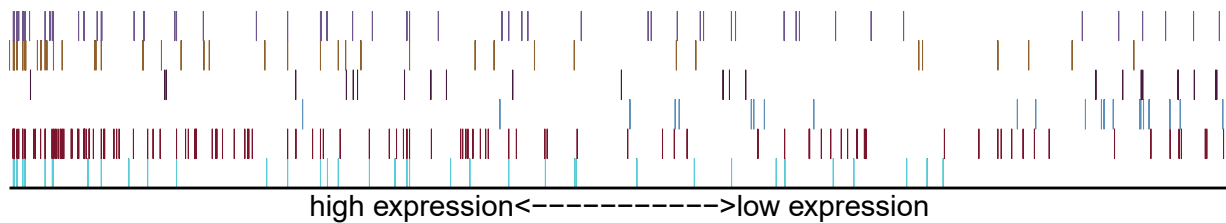

Supplement: Supplementary file 1 [file DataSheet3.zip › Input data and script2/GSEA analysis/MMP1/2.GSEA KEGG.pdf]

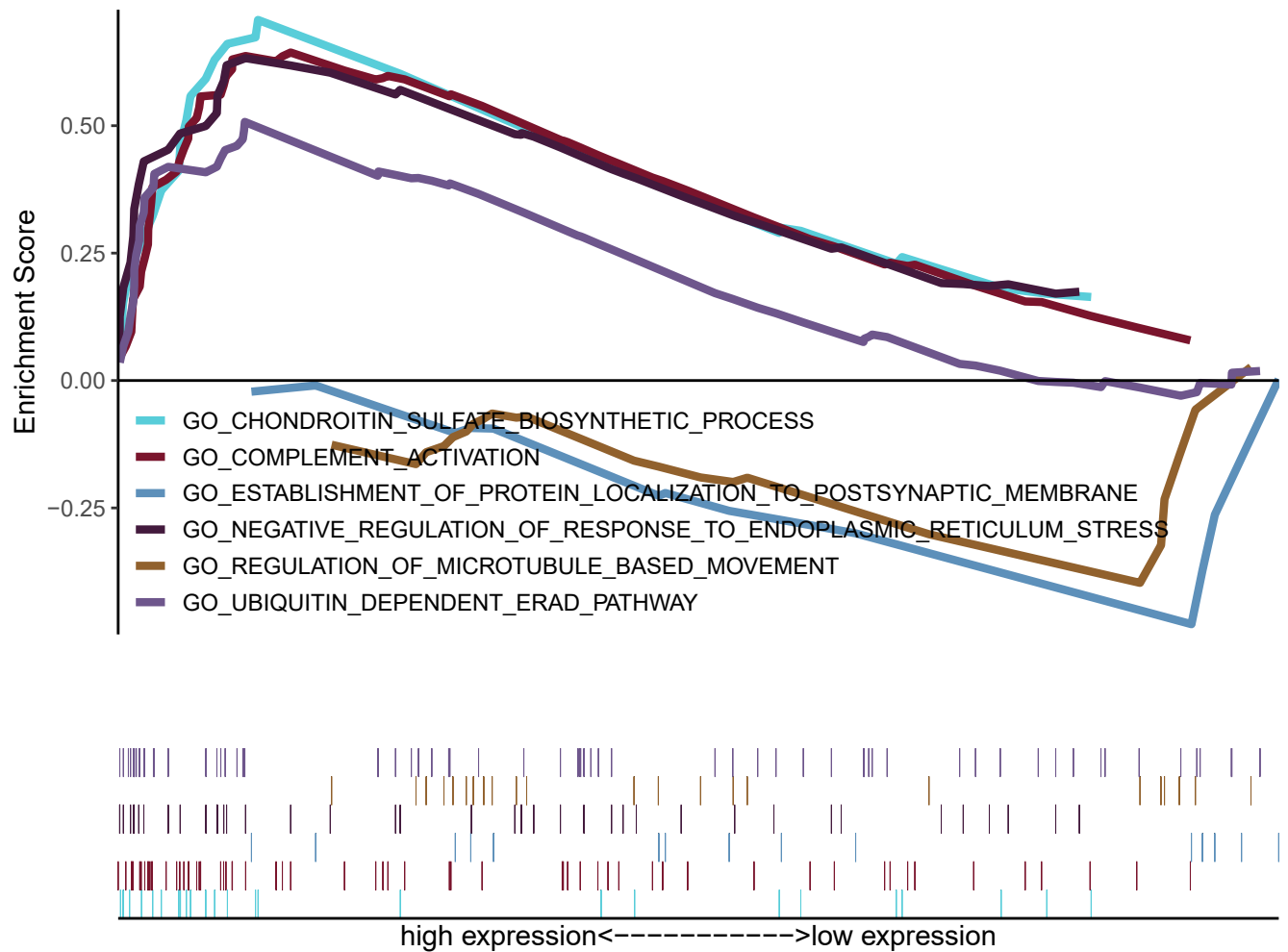

Supplement: Supplementary file 1 [file DataSheet3.zip › Input data and script2/GSEA analysis/MMP1/1.GSEA GO.pdf]

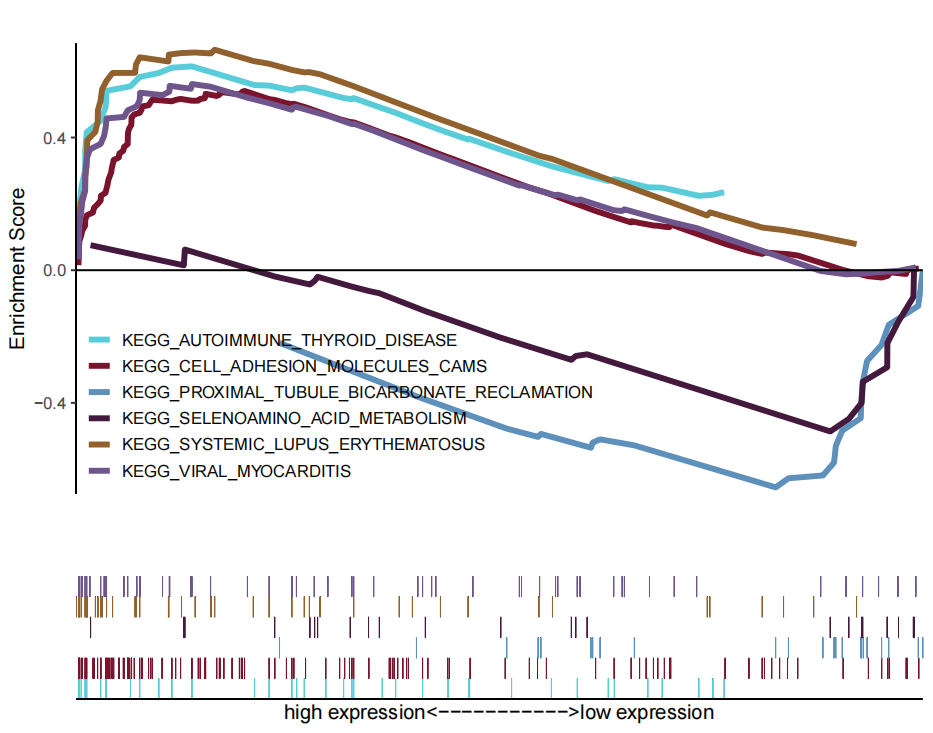

Supplement: Supplementary file 1 [file DataSheet3.zip › Input data and script2/GSEA analysis/MMP1/2.GSEA KEGG_00.png]

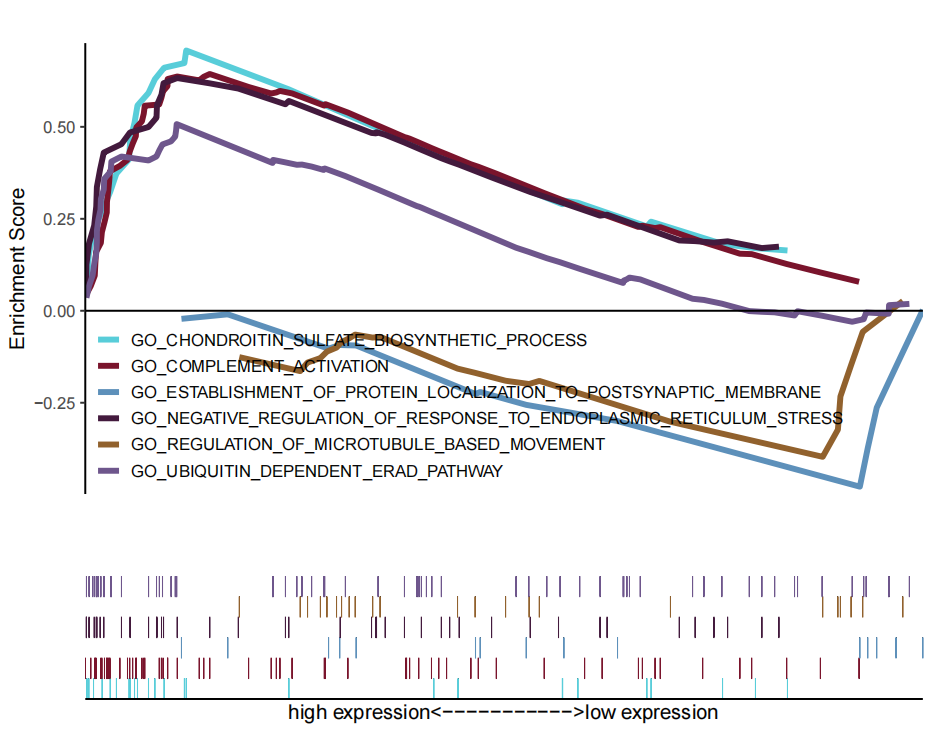

Supplement: Supplementary file 1 [file DataSheet3.zip › Input data and script2/GSEA analysis/MMP1/1.GSEA GO_00.png]

Enrichment Score

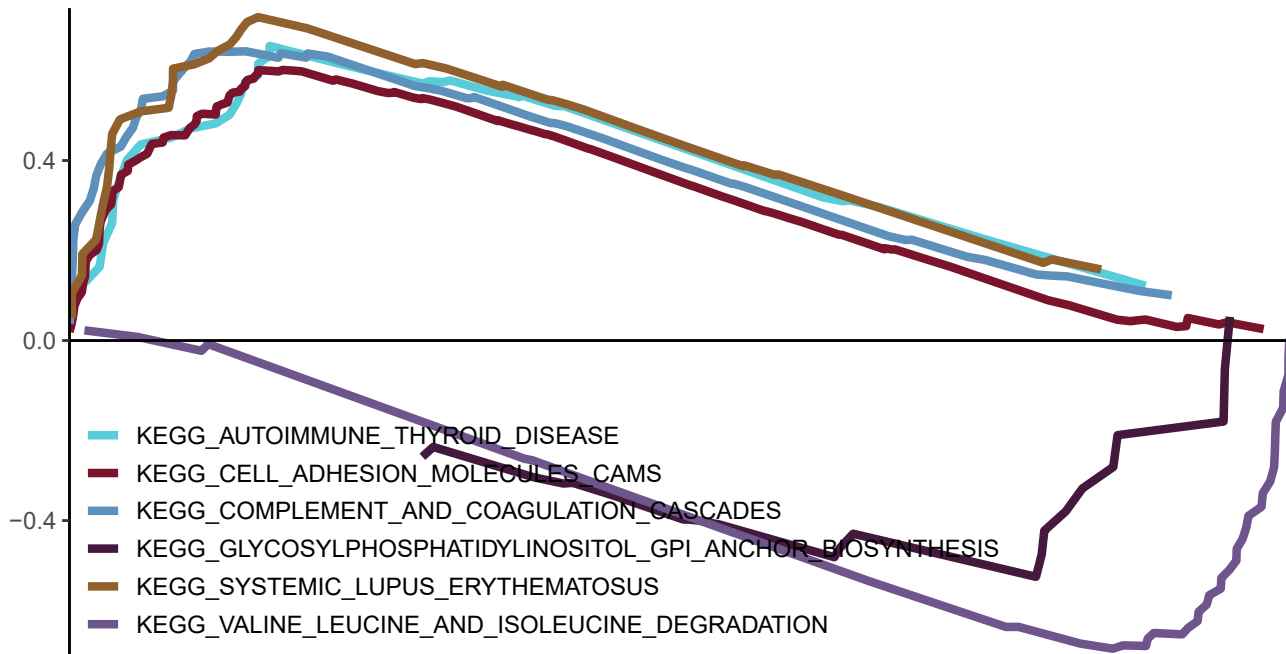

high expression<----->low expression

Supplement: Supplementary file 1 [file DataSheet3.zip › Input data and script2/GSEA analysis/SAA1/2.GSEA KEGG.pdf]

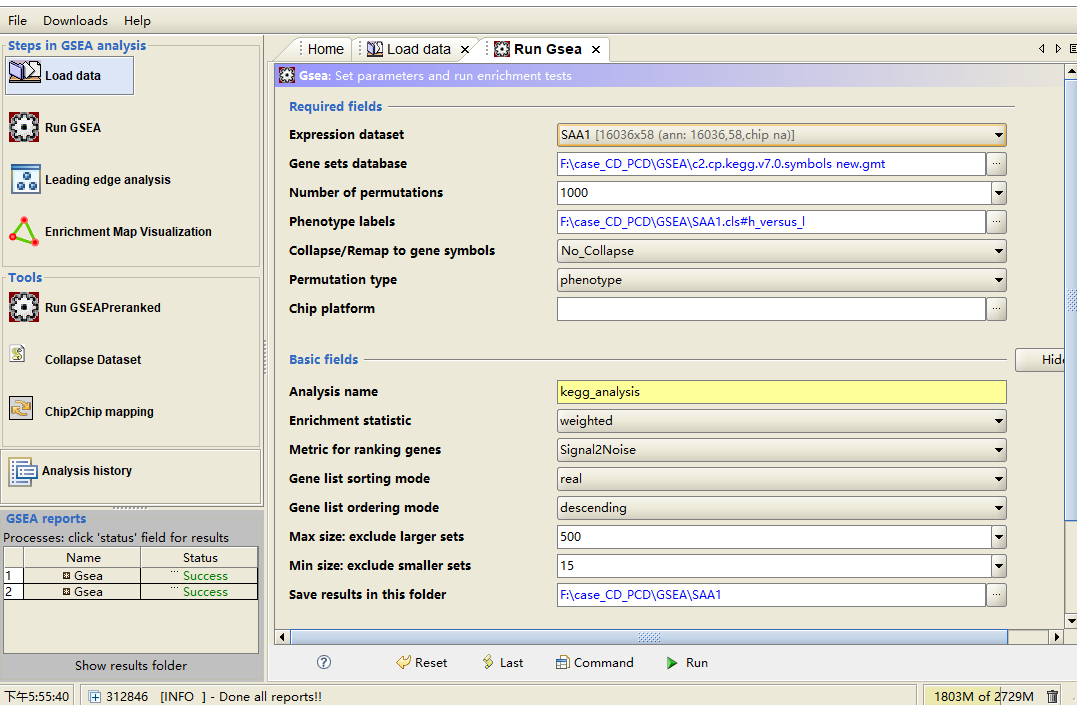

Supplement: Supplementary file 1 [file DataSheet3.zip › Input data and script2/GSEA analysis/SAA1/σ▒Åσ╣òμê¬σ¢╛ 2022-04-06 175549.png]

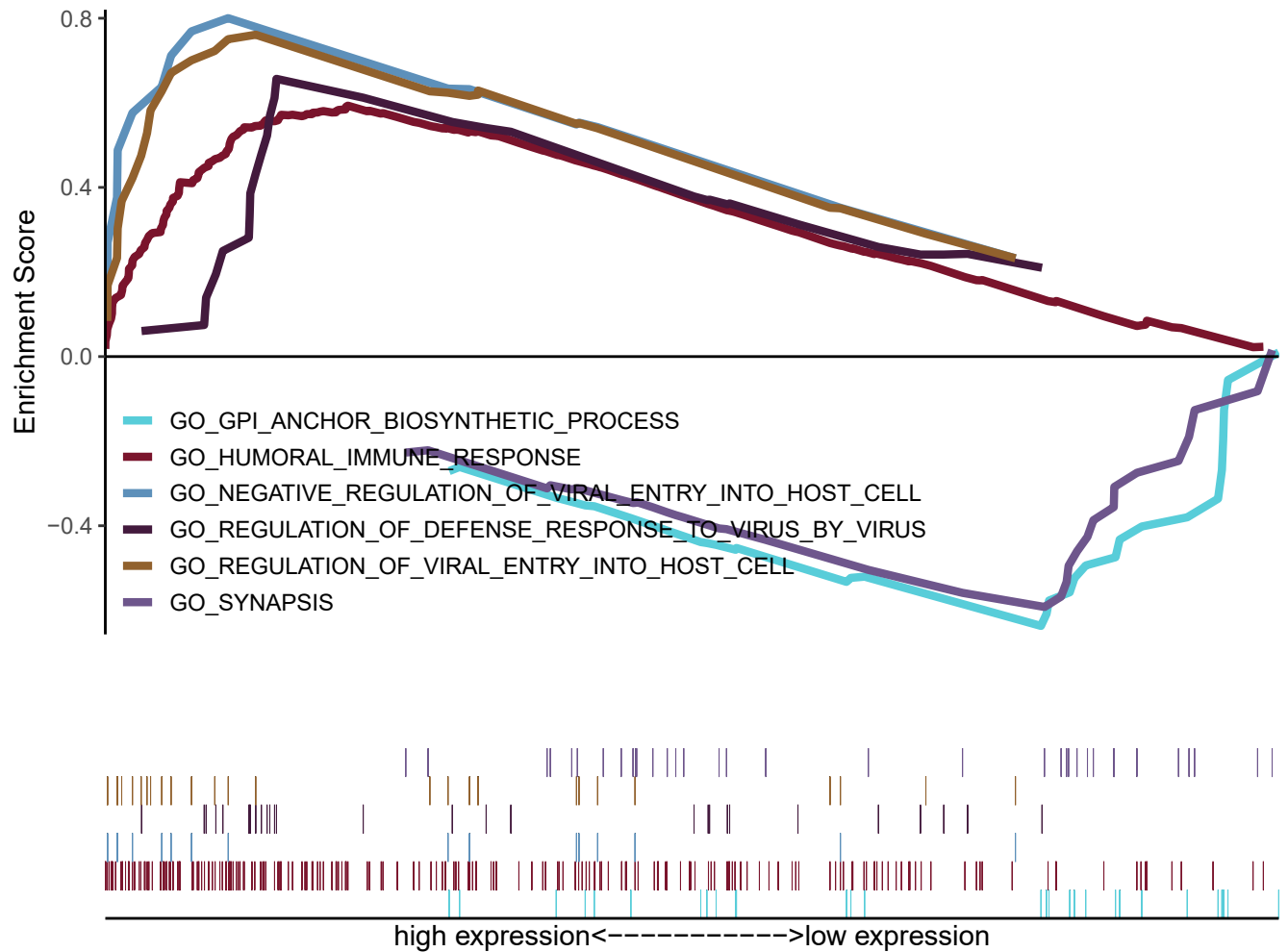

Supplement: Supplementary file 1 [file DataSheet3.zip › Input data and script2/GSEA analysis/SAA1/1.GSEA GO.pdf]

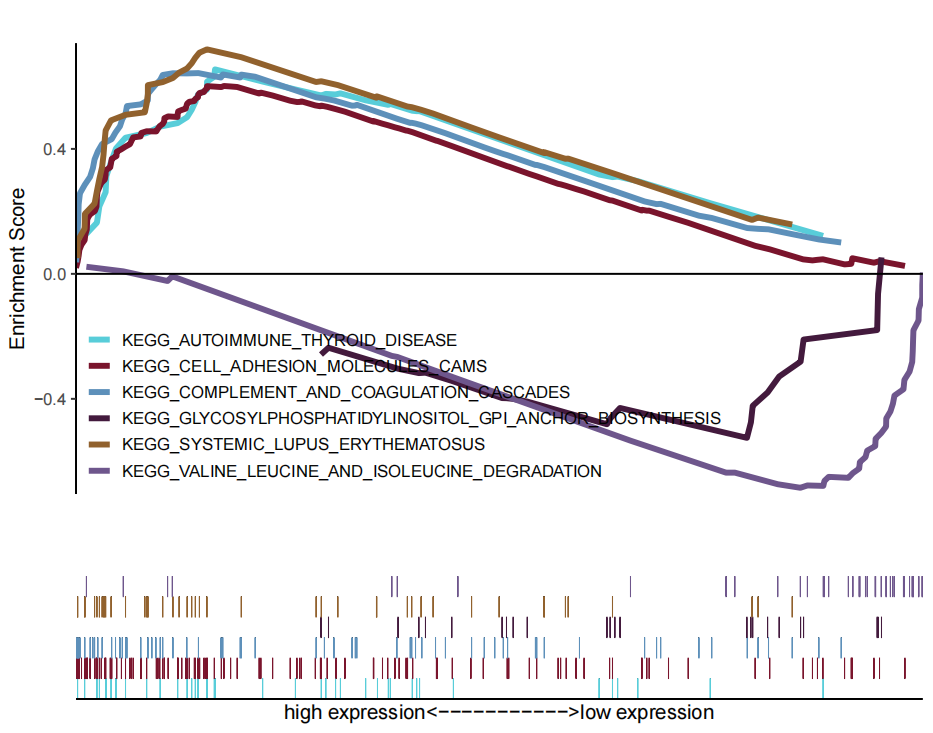

Supplement: Supplementary file 1 [file DataSheet3.zip › Input data and script2/GSEA analysis/SAA1/2.GSEA KEGG_00.png]

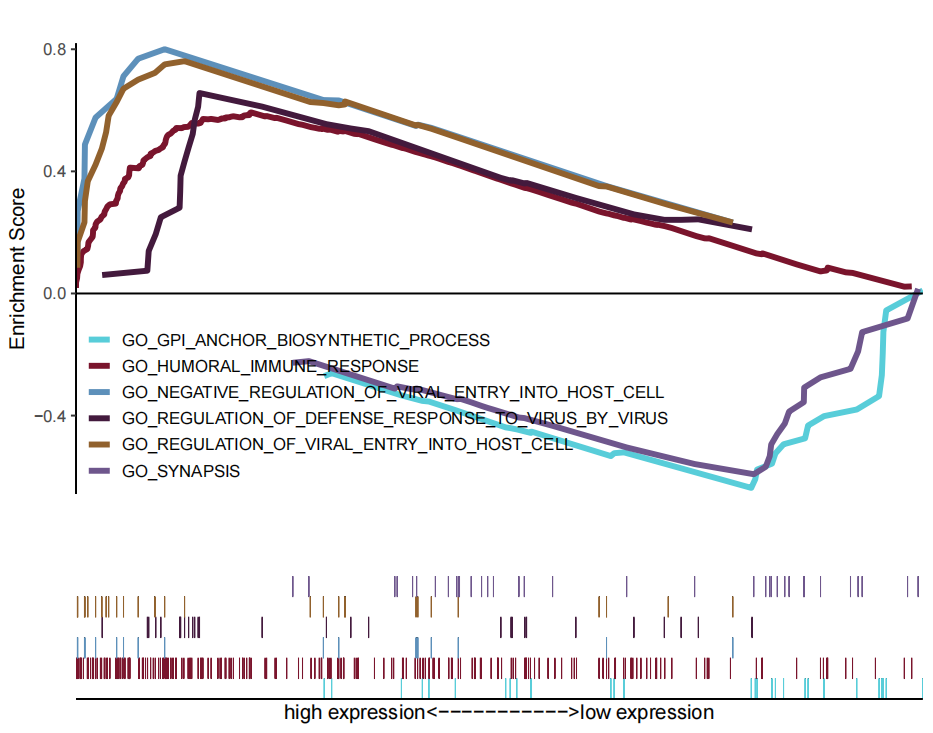

Supplement: Supplementary file 1 [file DataSheet3.zip › Input data and script2/GSEA analysis/SAA1/1.GSEA GO_00.png]

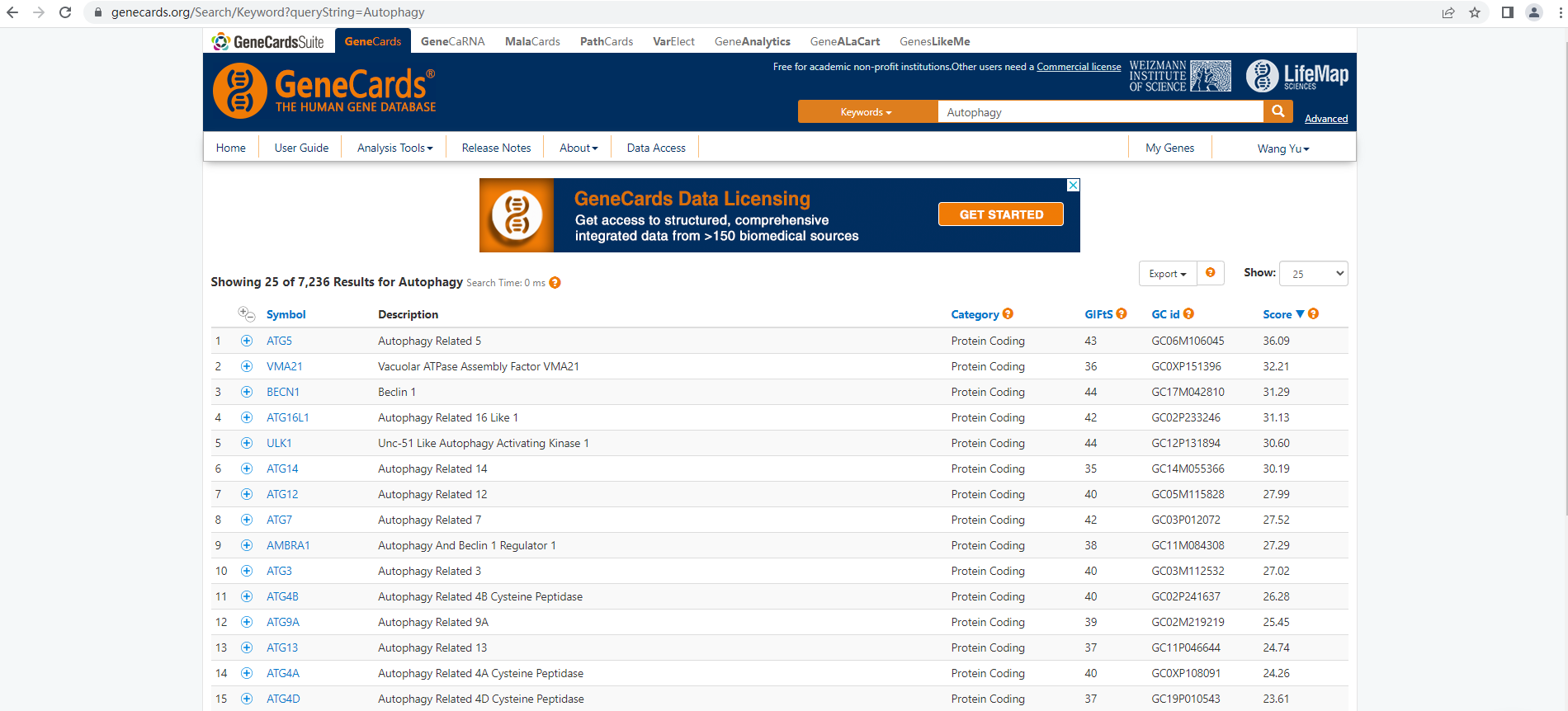

Supplement: Supplementary file 1 [file DataSheet3.zip › Input data and script2/Proerammed cell death genes collection/Autophagy_gene_collection/σ▒Åσ╣òμê¬σ¢╛ 2022-03-28 171422.png]

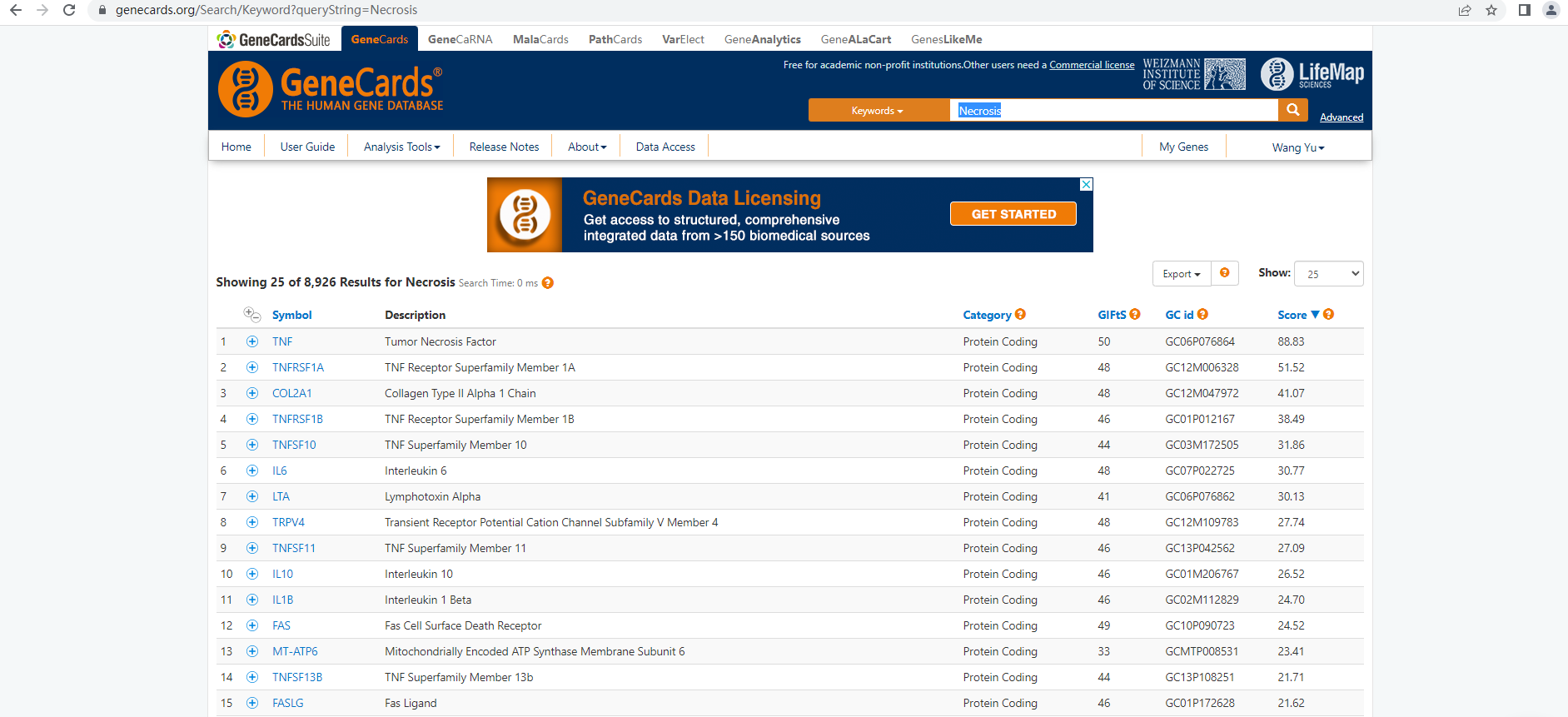

Supplement: Supplementary file 1 [file DataSheet3.zip › Input data and script2/Proerammed cell death genes collection/Necrosis_gene_collection/σ▒Åσ╣òμê¬σ¢╛ 2022-03-28 171941.png]

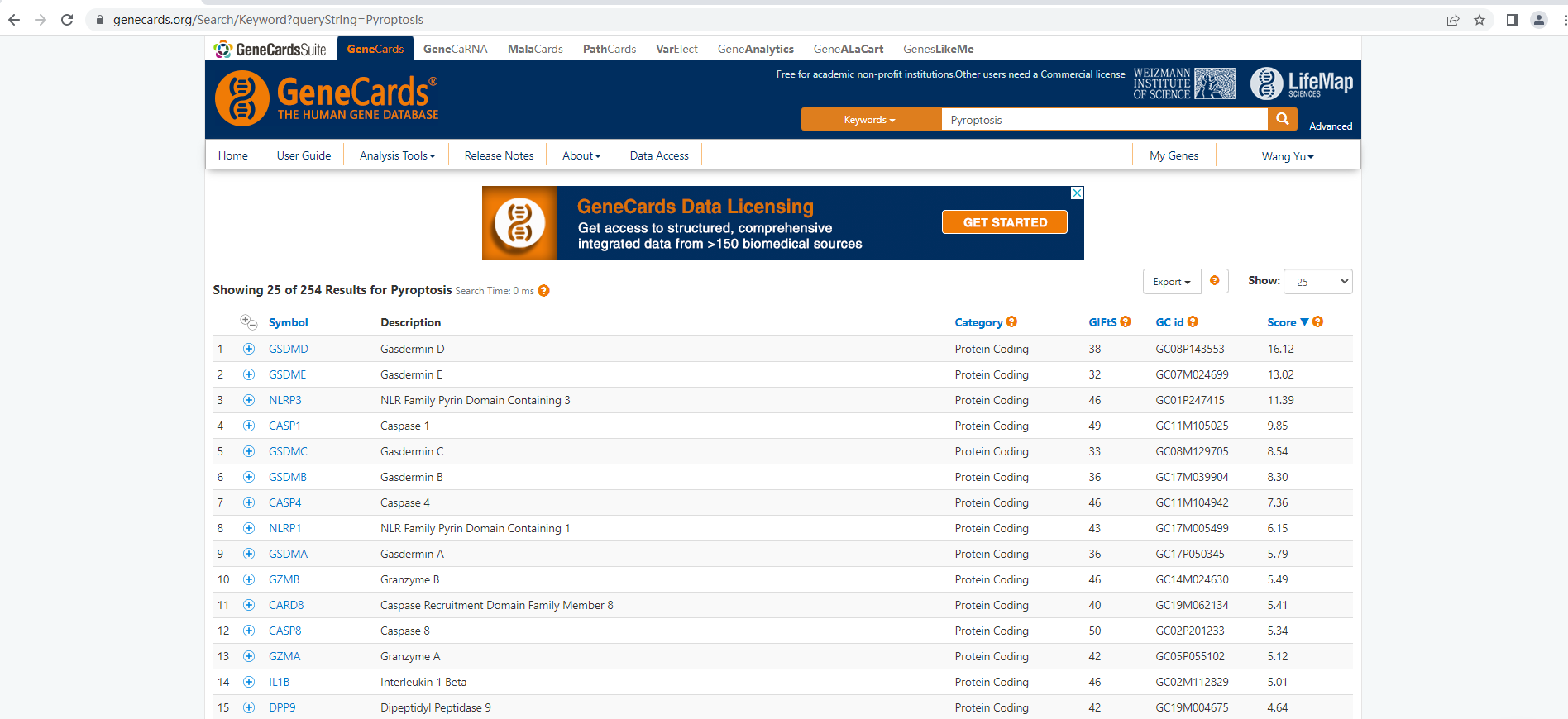

Supplement: Supplementary file 1 [file DataSheet3.zip › Input data and script2/Proerammed cell death genes collection/Pyroptosis_gene_collection/σ▒Åσ╣òμê¬σ¢╛ 2022-03-28 171638.png]

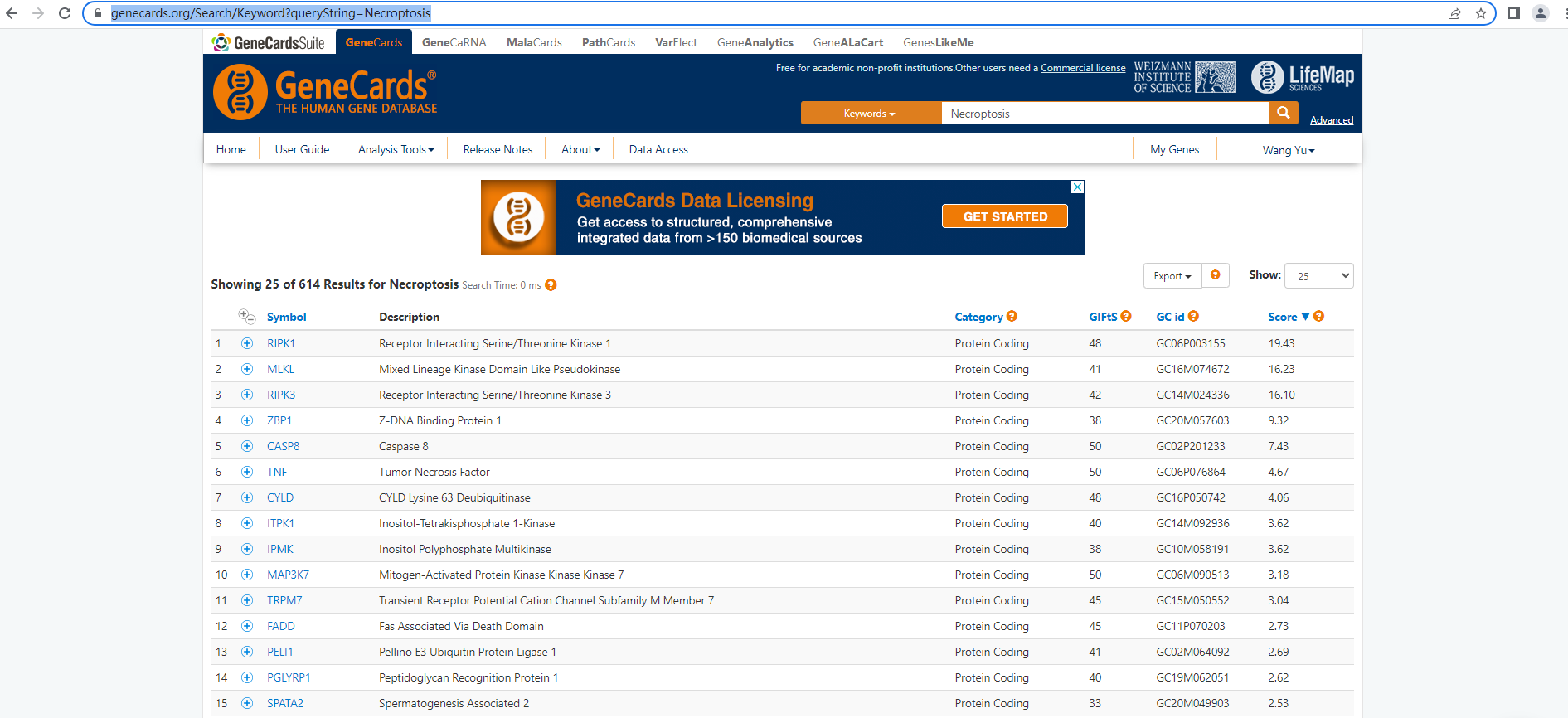

Supplement: Supplementary file 1 [file DataSheet3.zip › Input data and script2/Proerammed cell death genes collection/Necroptosis_gene_collection/σ▒Åσ╣òμê¬σ¢╛ 2022-03-28 171301.png]

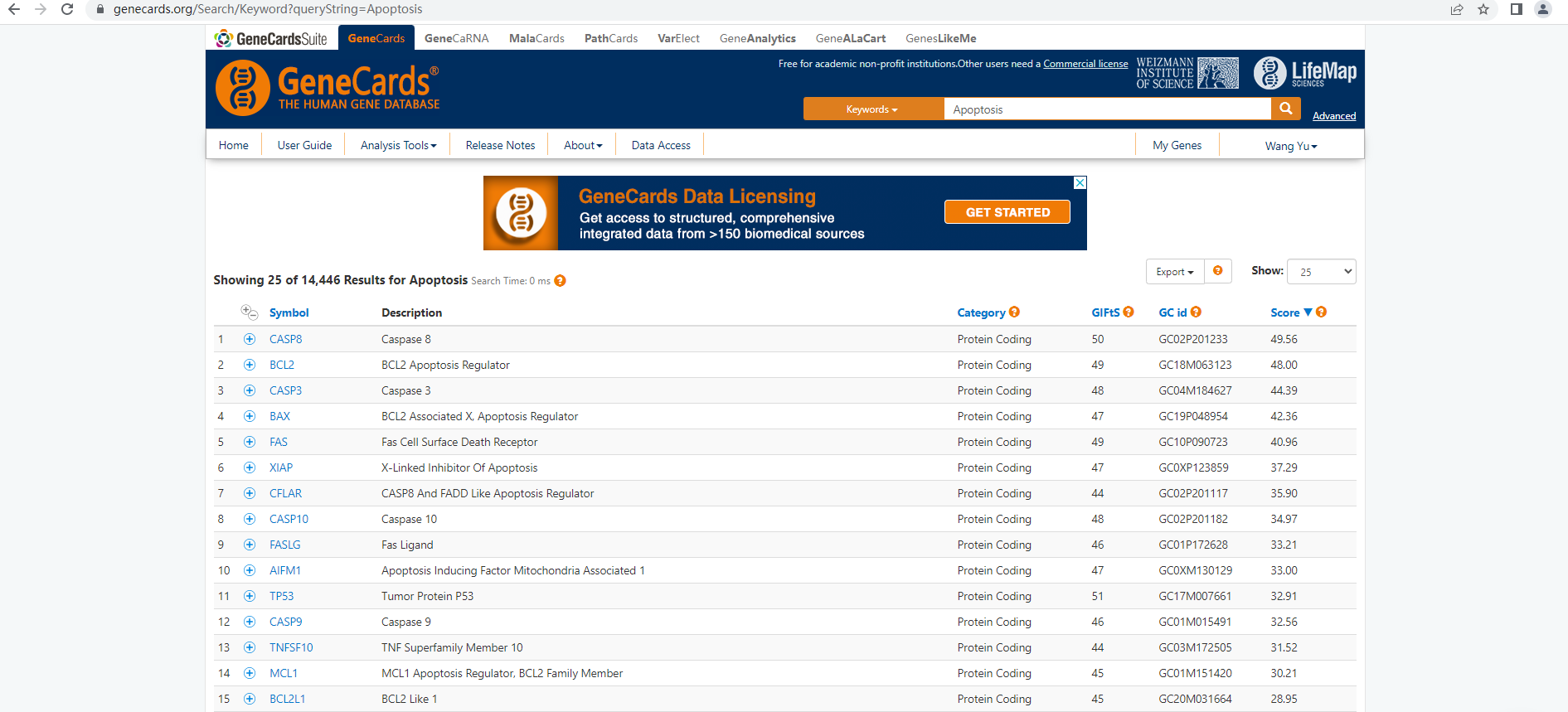

Supplement: Supplementary file 1 [file DataSheet3.zip › Input data and script2/Proerammed cell death genes collection/Apoptosis_gene_collection/σ▒Åσ╣òμê¬σ¢╛ 2022-03-28 171122.png]

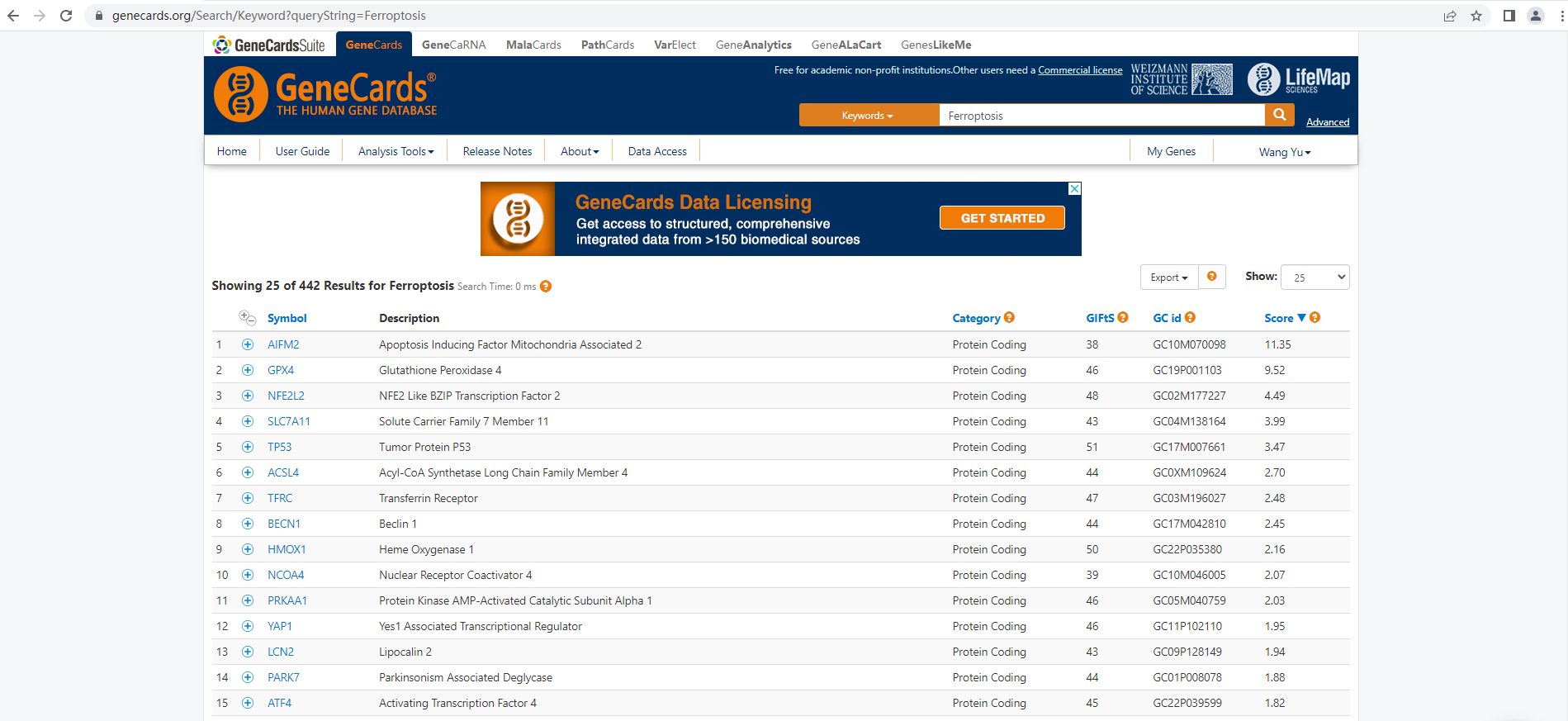

Supplement: Supplementary file 1 [file DataSheet3.zip › Input data and script2/Proerammed cell death genes collection/Ferroptosis_gene_collection/σ▒Åσ╣òμê¬σ¢╛ 2022-03-28 171525.png]

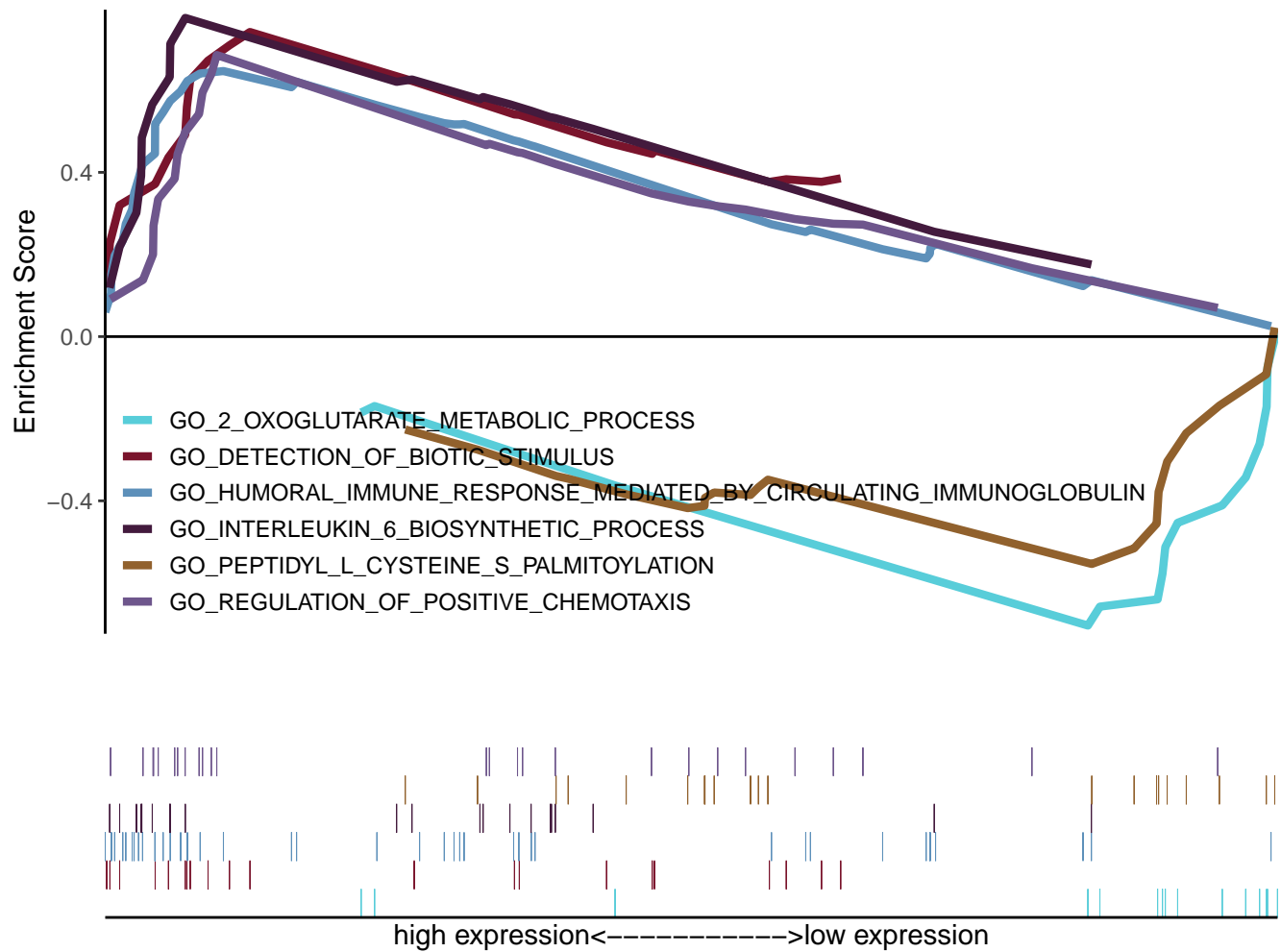

Supplement: Supplementary file 1 [file DataSheet3.zip › Input data and script2/GSEA analysis/PLAU/plot_go/1.GSEA GO.pdf]

Enrichment Score

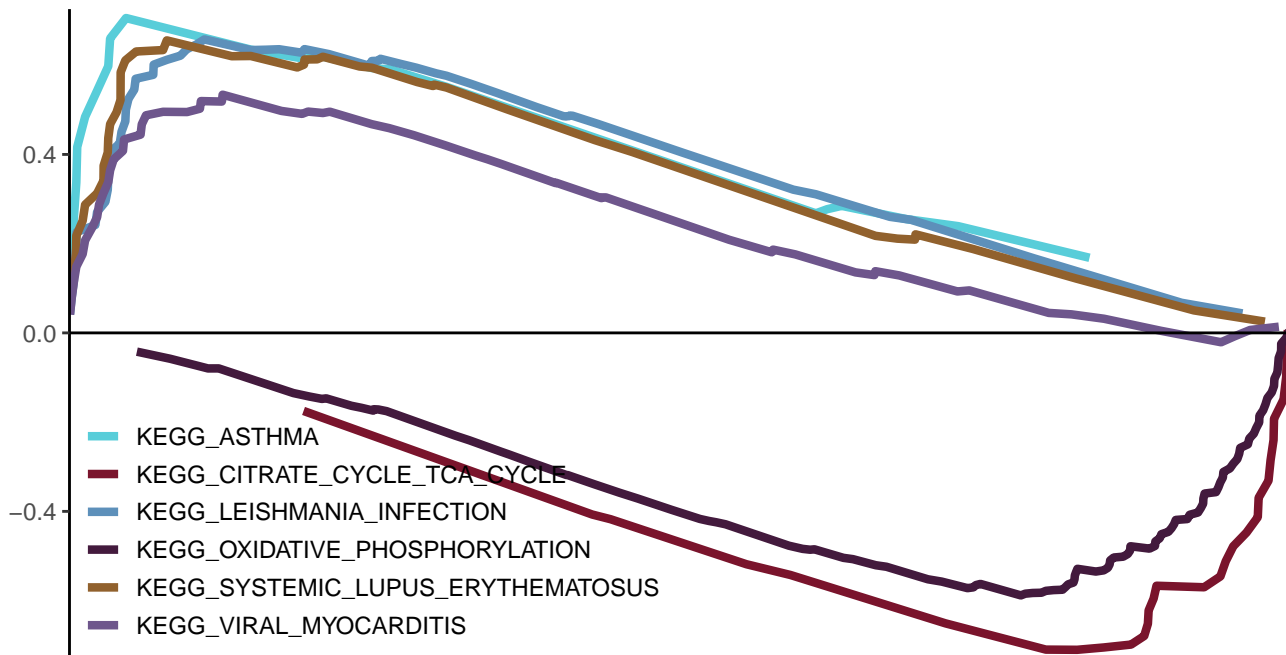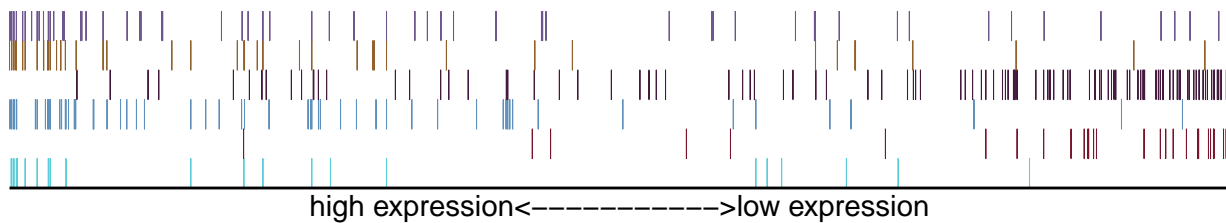

Supplement: Supplementary file 1 [file DataSheet3.zip › Input data and script2/GSEA analysis/PLAU/plot_kegg/2.GSEA KEGG.pdf]

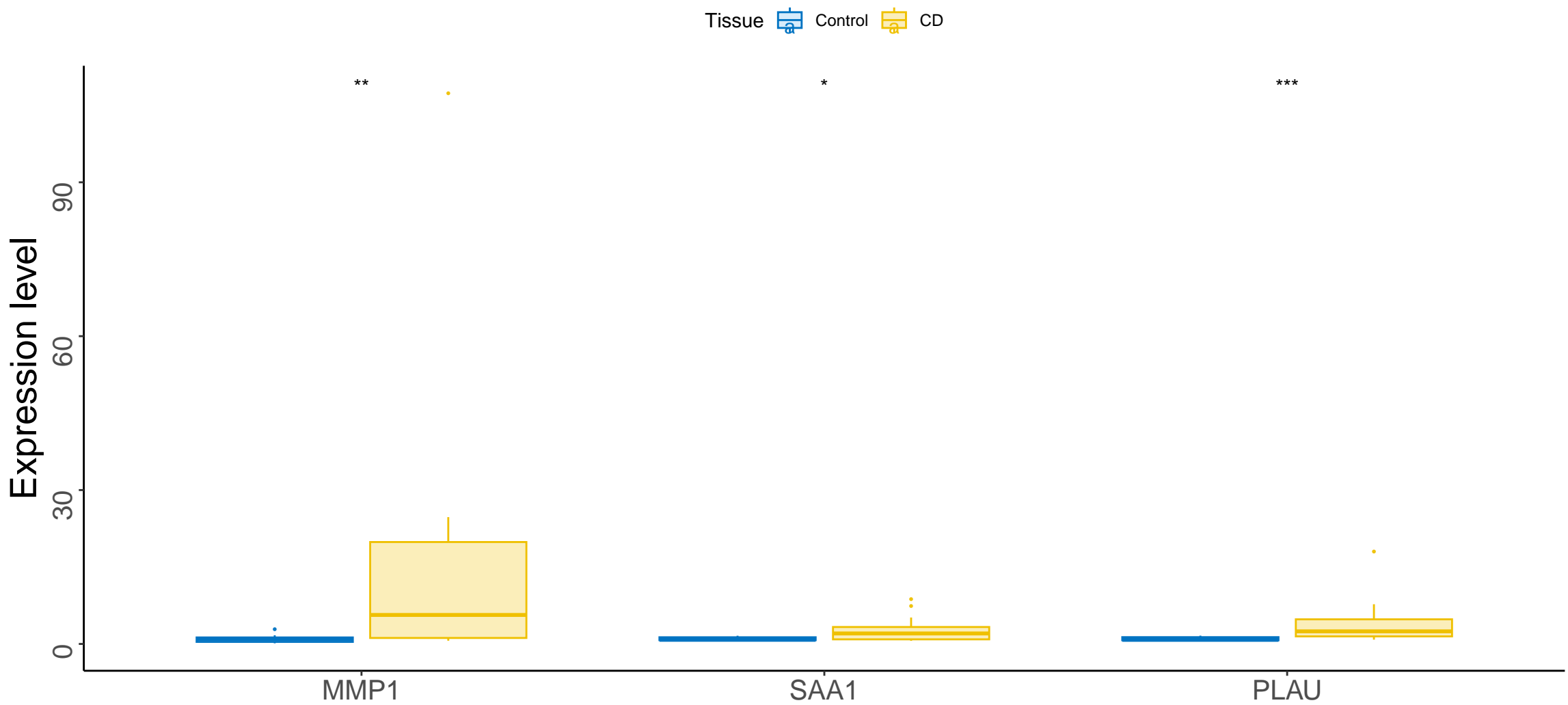

Supplement: Supplementary file 2 [file DataSheet4.zip › Input data and script3/ROC/Expression level.pdf]

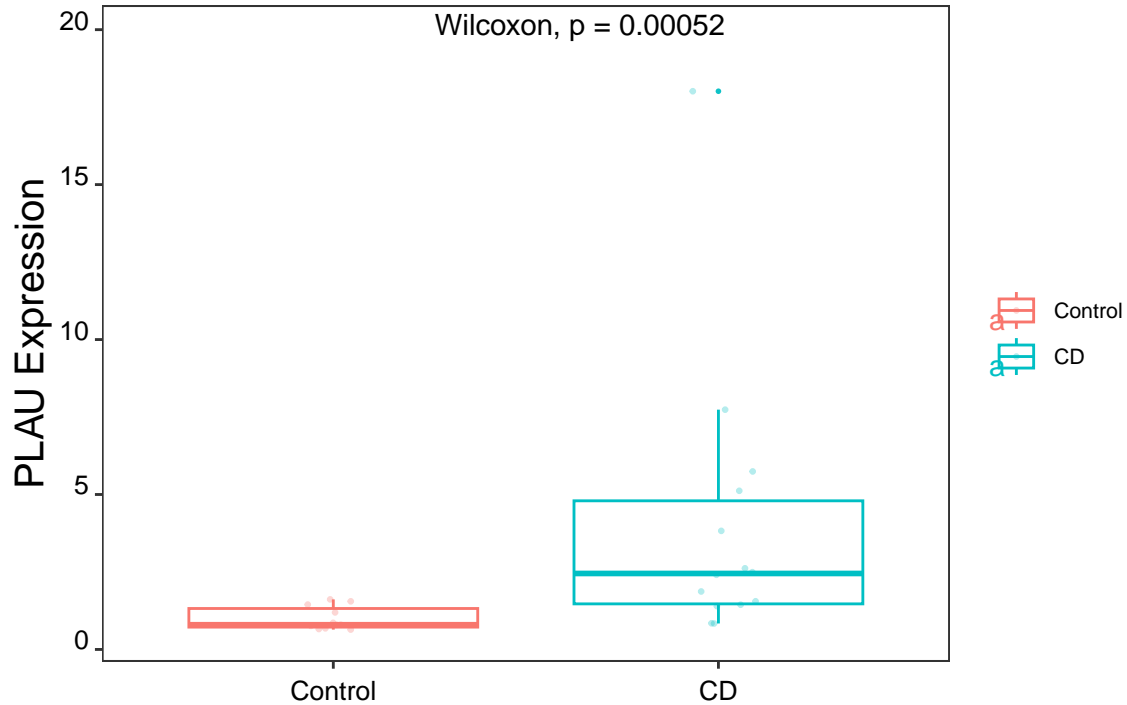

Supplement: Supplementary file 2 [file DataSheet4.zip › Input data and script3/ROC/PLAU.HealthyDisease.pdf]

ROC for MMP1

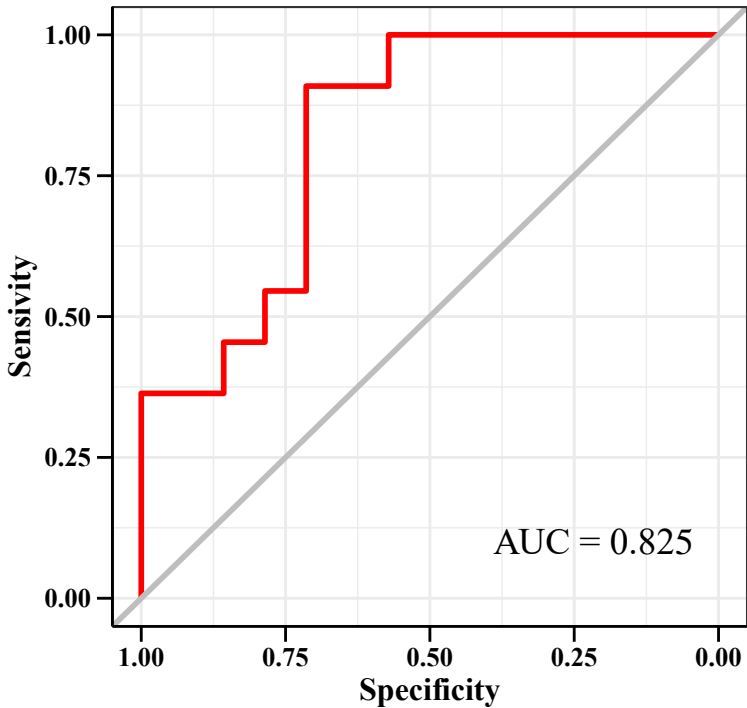

Supplement: Supplementary file 2 [file DataSheet4.zip › Input data and script3/ROC/1.ROC for MMP1.pdf]

ROC for PLAU

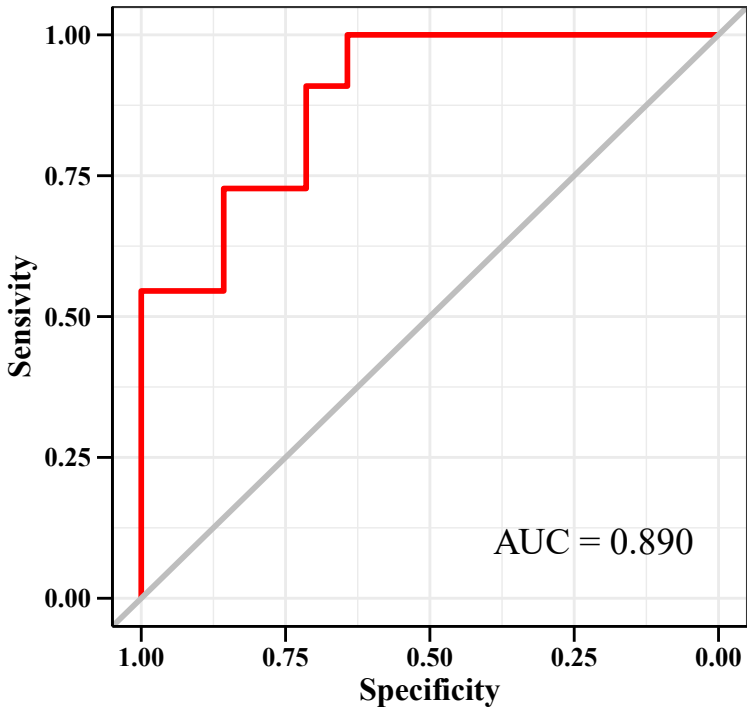

Supplement: Supplementary file 2 [file DataSheet4.zip › Input data and script3/ROC/2.ROC for PLAU.pdf]

ROC for SAA1

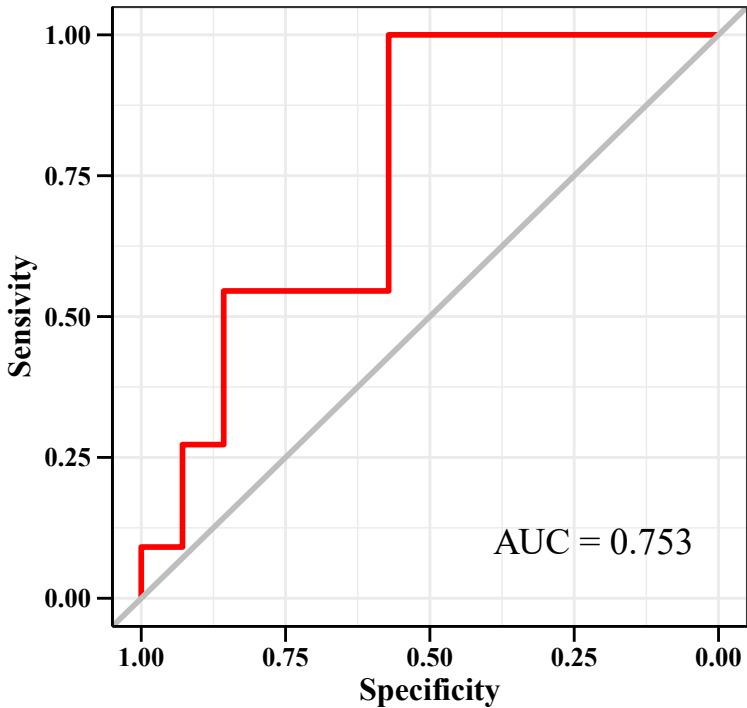

Supplement: Supplementary file 2 [file DataSheet4.zip › Input data and script3/ROC/3.ROC for SAA1.pdf]

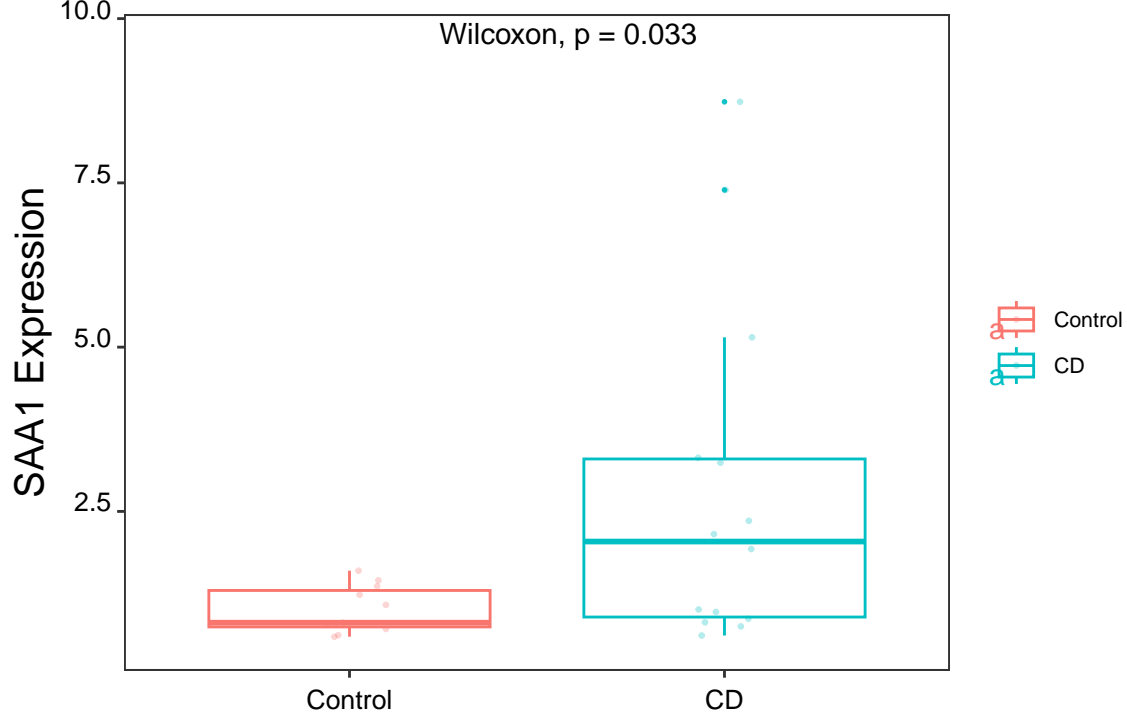

Supplement: Supplementary file 2 [file DataSheet4.zip › Input data and script3/ROC/SAA1.HealthyDisease.pdf]

MMP1 Expression

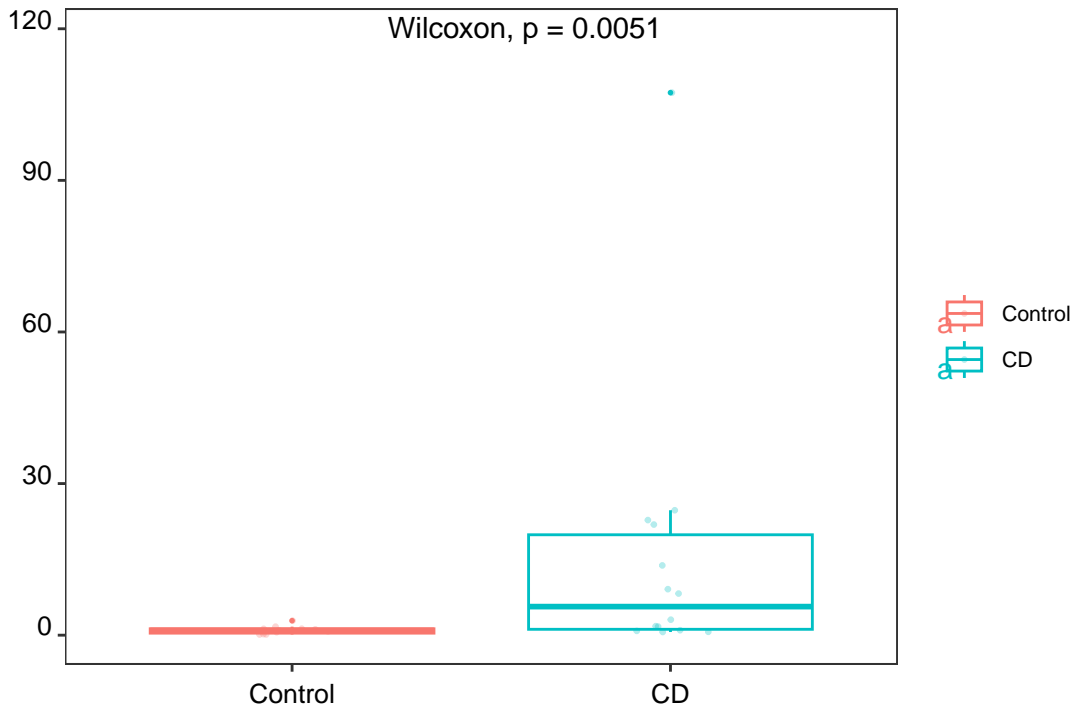

Supplement: Supplementary file 2 [file DataSheet4.zip › Input data and script3/ROC/MMP1.HealthyDisease.pdf]

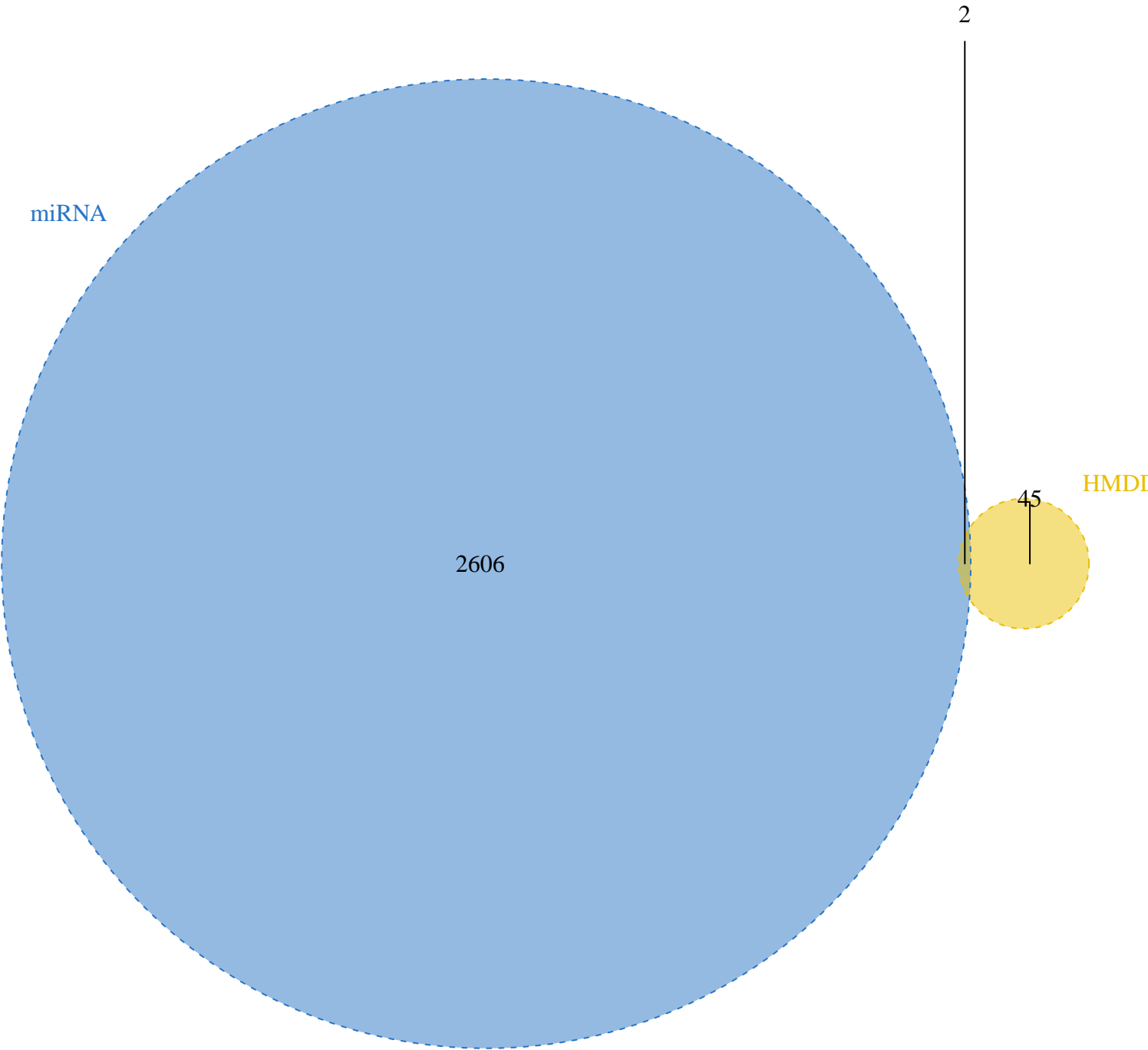

Supplement: Supplementary file 2 [file DataSheet4.zip › Input data and script3/ceRNAnet/Venn.pdf]

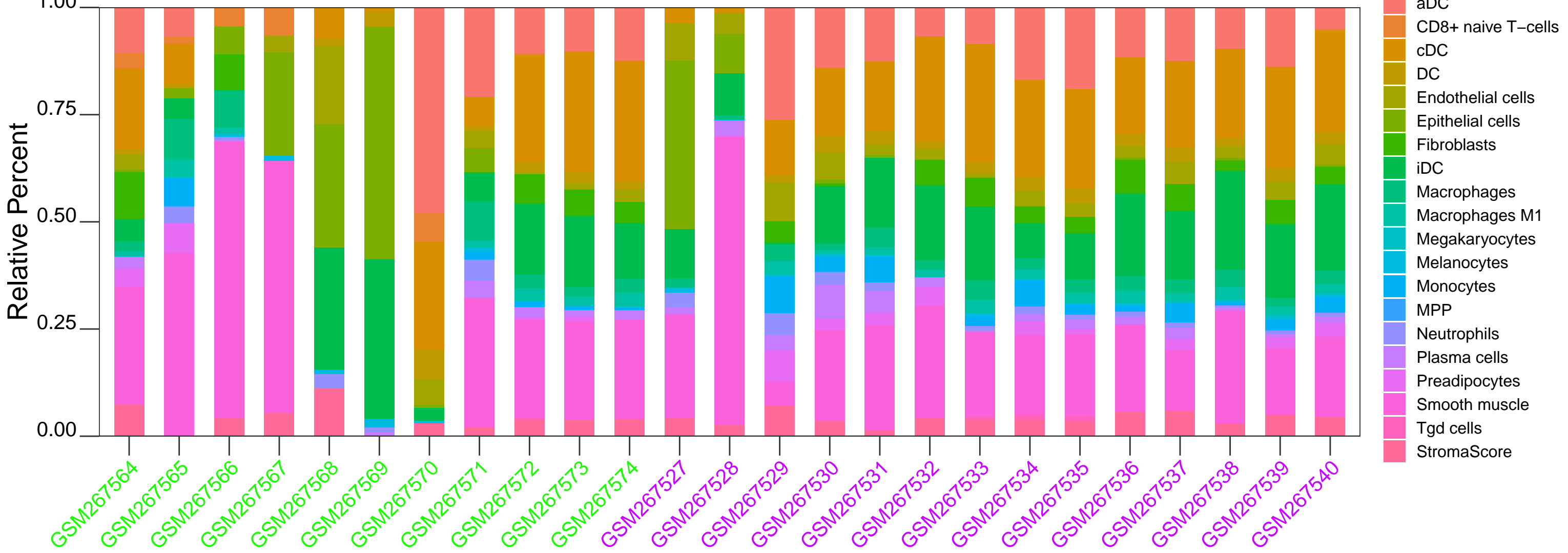

Supplement: Supplementary file 2 [file DataSheet4.zip › Input data and script3/Xcell-Immune infiltration/1.Immune infiltration.pdf]

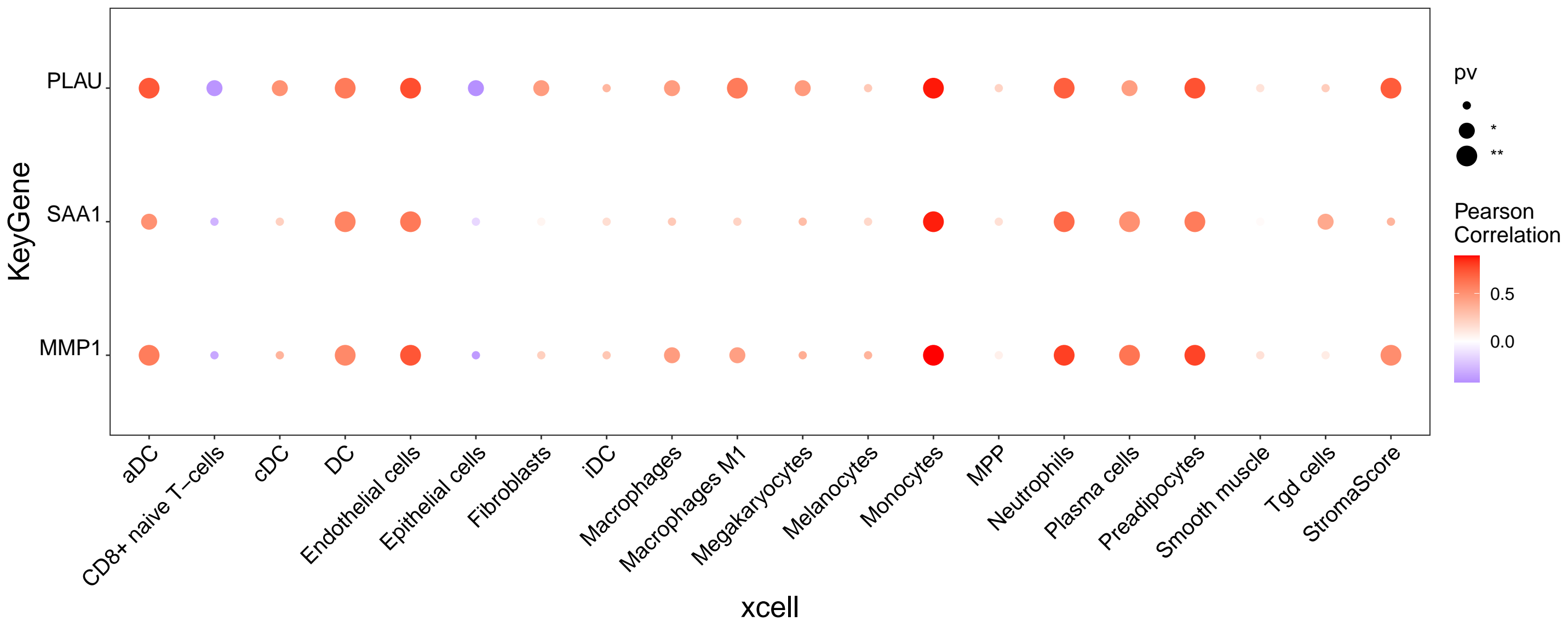

Supplement: Supplementary file 2 [file DataSheet4.zip › Input data and script3/Xcell-Immune infiltration/4.KeyGene ~ Xcell.pdf]

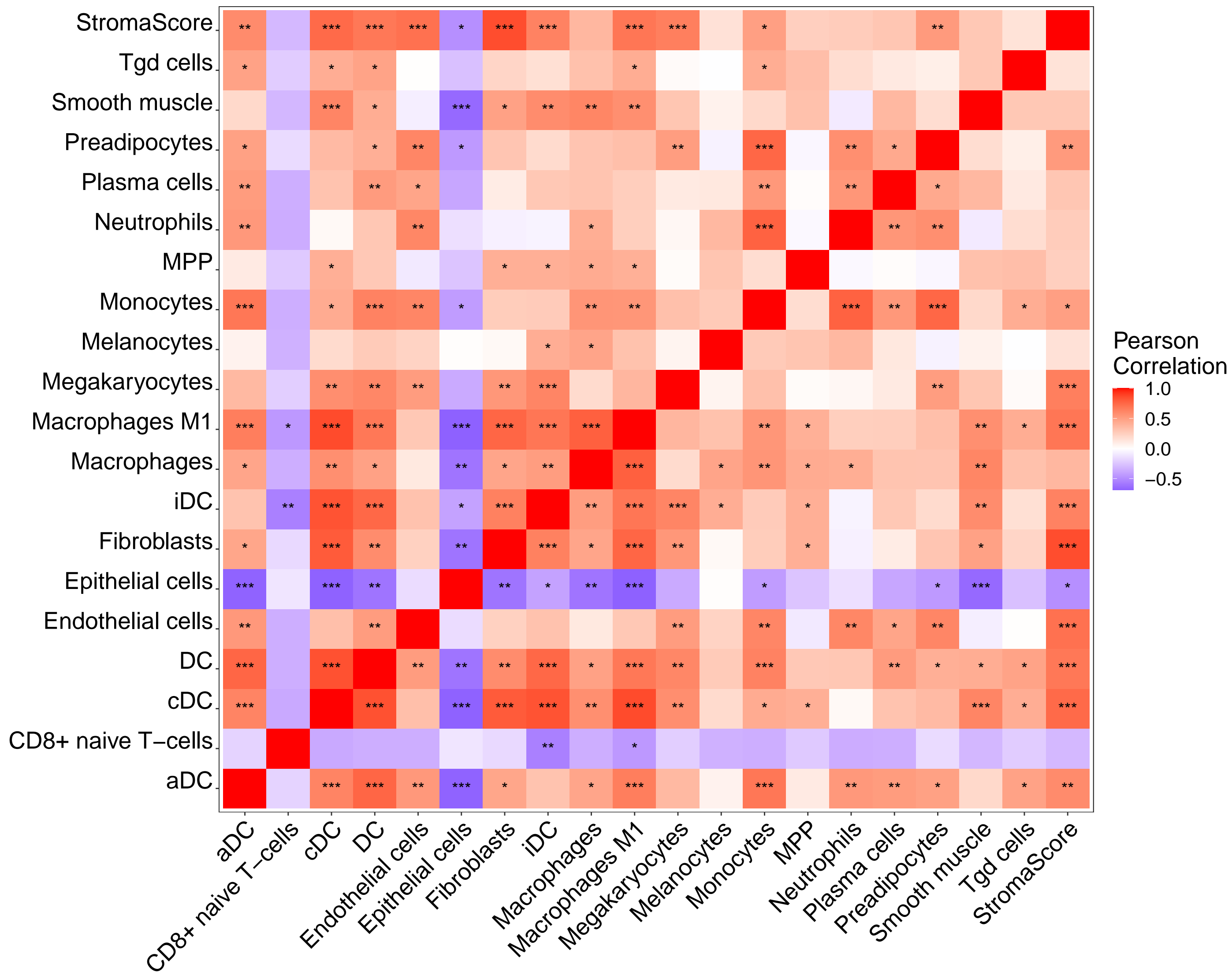

Supplement: Supplementary file 2 [file DataSheet4.zip › Input data and script3/Xcell-Immune infiltration/2.Immune cell correlation map.pdf]

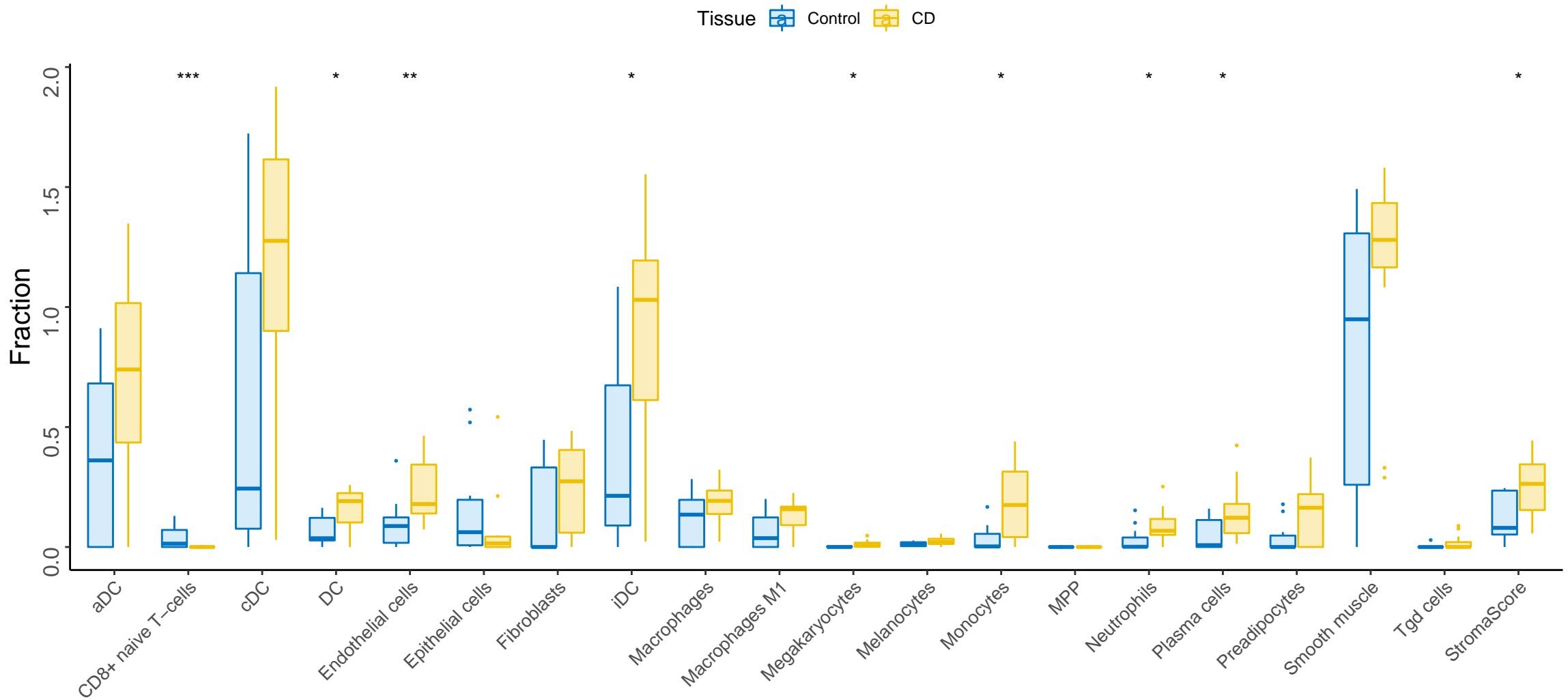

Supplement: Supplementary file 2 [file DataSheet4.zip › Input data and script3/Xcell-Immune infiltration/3.Immune cell comparison.pdf]

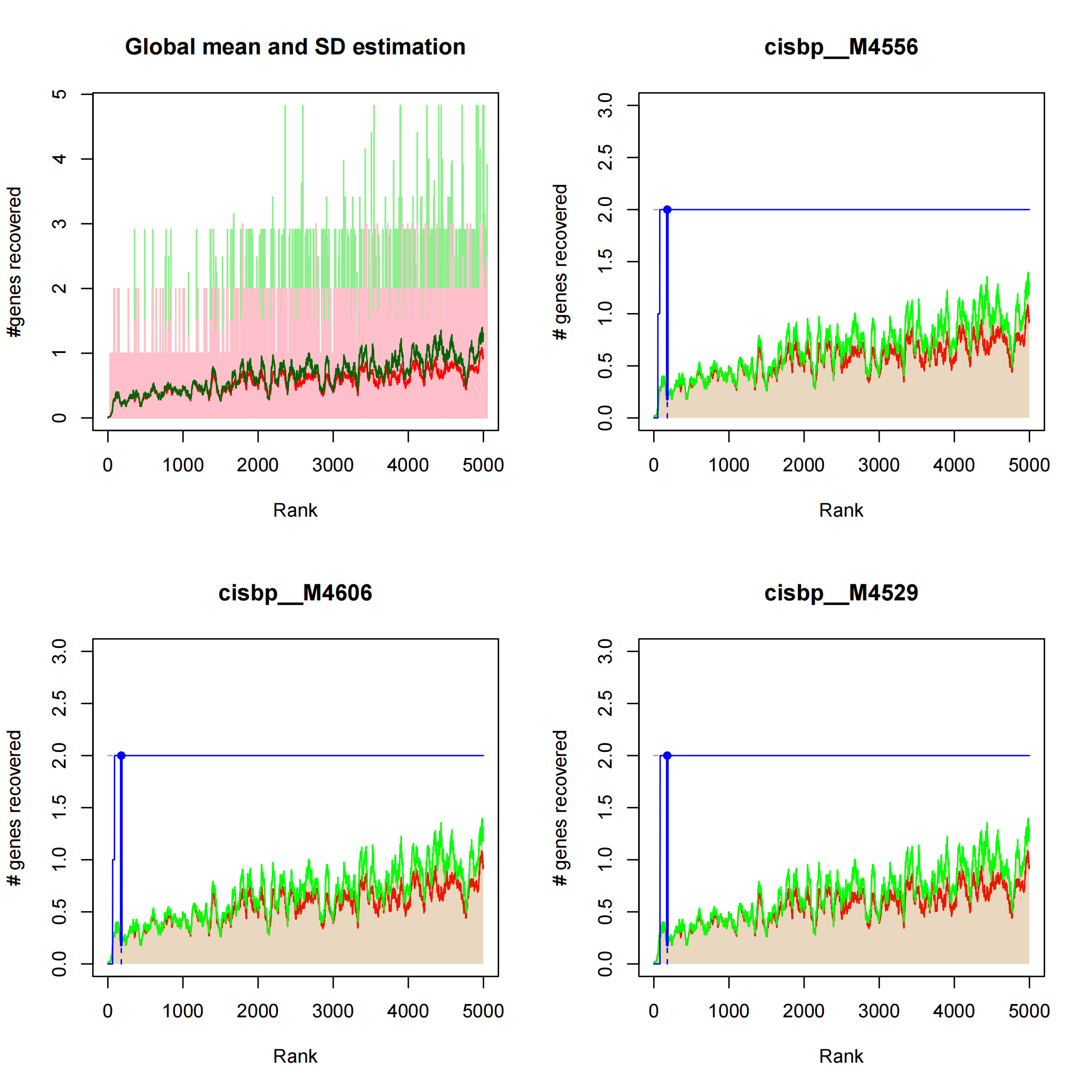

Supplement: Supplementary file 2 [file DataSheet4.zip › Input data and script3/TF/motif enrichment best_00.png]

**Global mean and SD estimation**

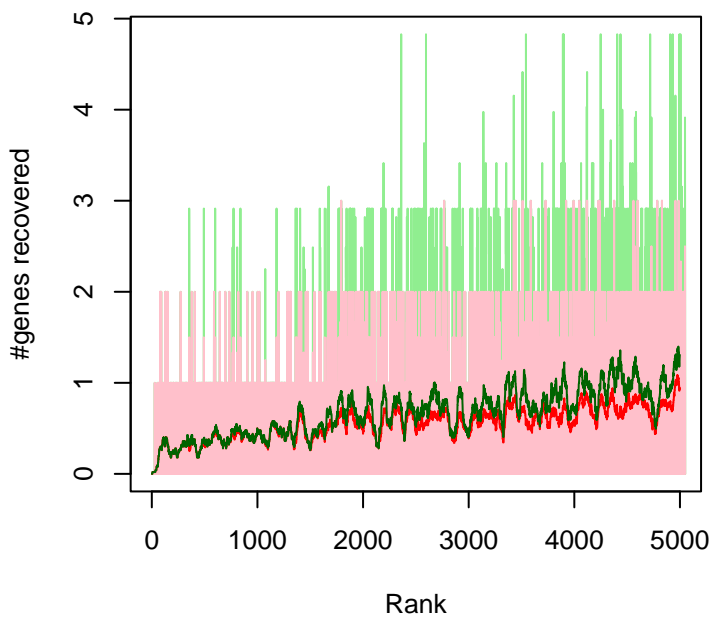

**cisbp\_M4556**

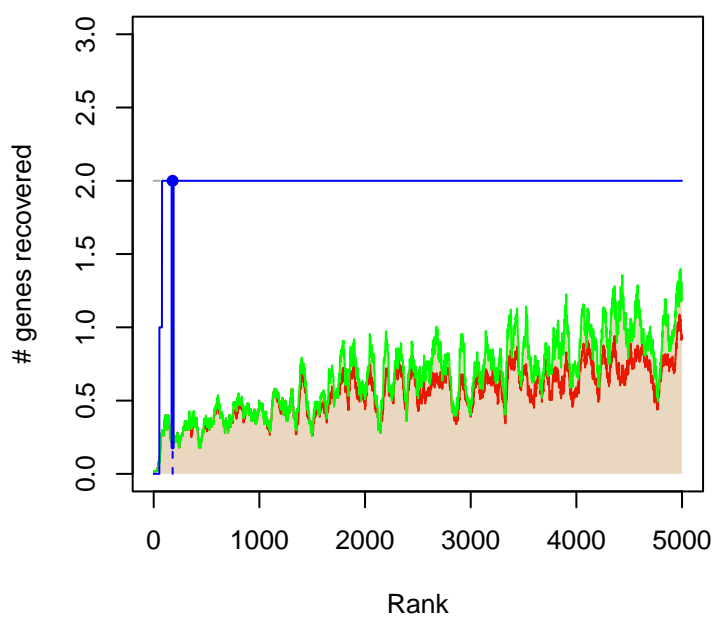

**cisbp\_M4606**

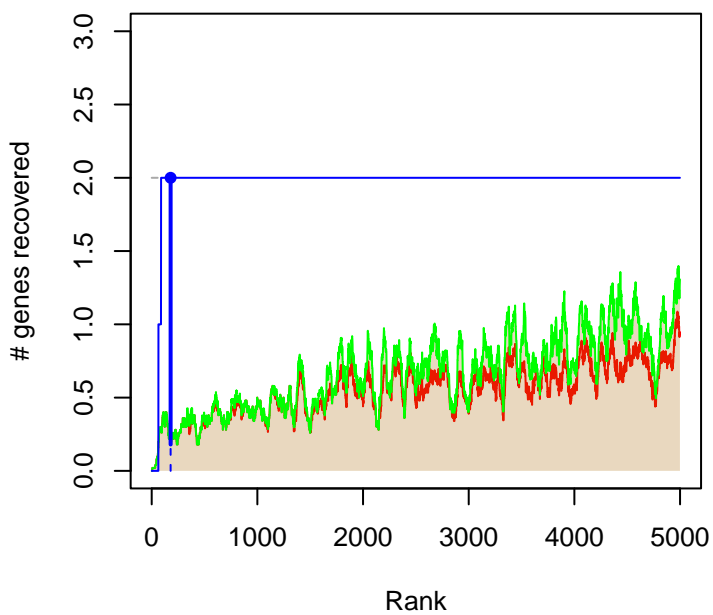

**cisbp\_M4529**

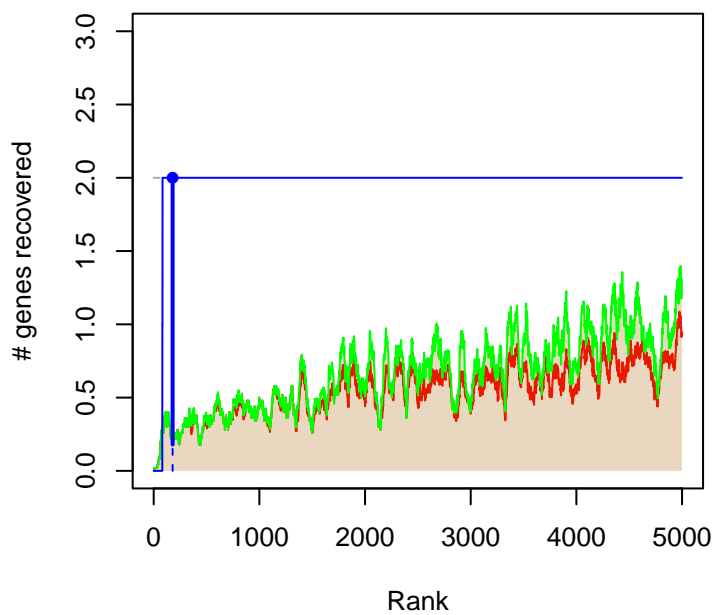

Supplement: Supplementary file 2 [file DataSheet4.zip › Input data and script3/TF/motif enrichment best.pdf]

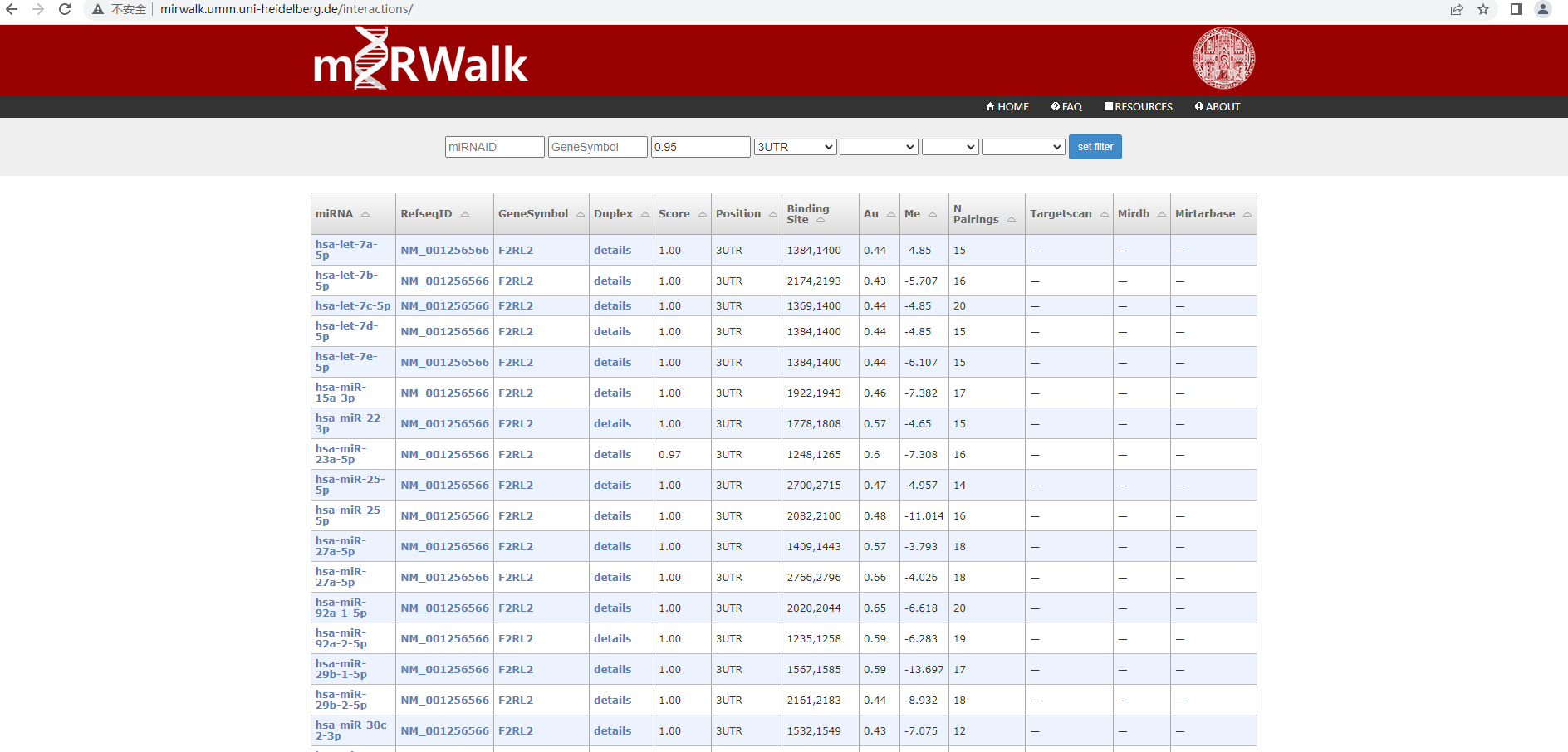

Supplement: Supplementary file 2 [file DataSheet4.zip › Input data and script3/ceRNAnet/Apoptosis_gene/σ▒Åσ╣òμê¬σ¢╛ 2022-04-15 154231.png]

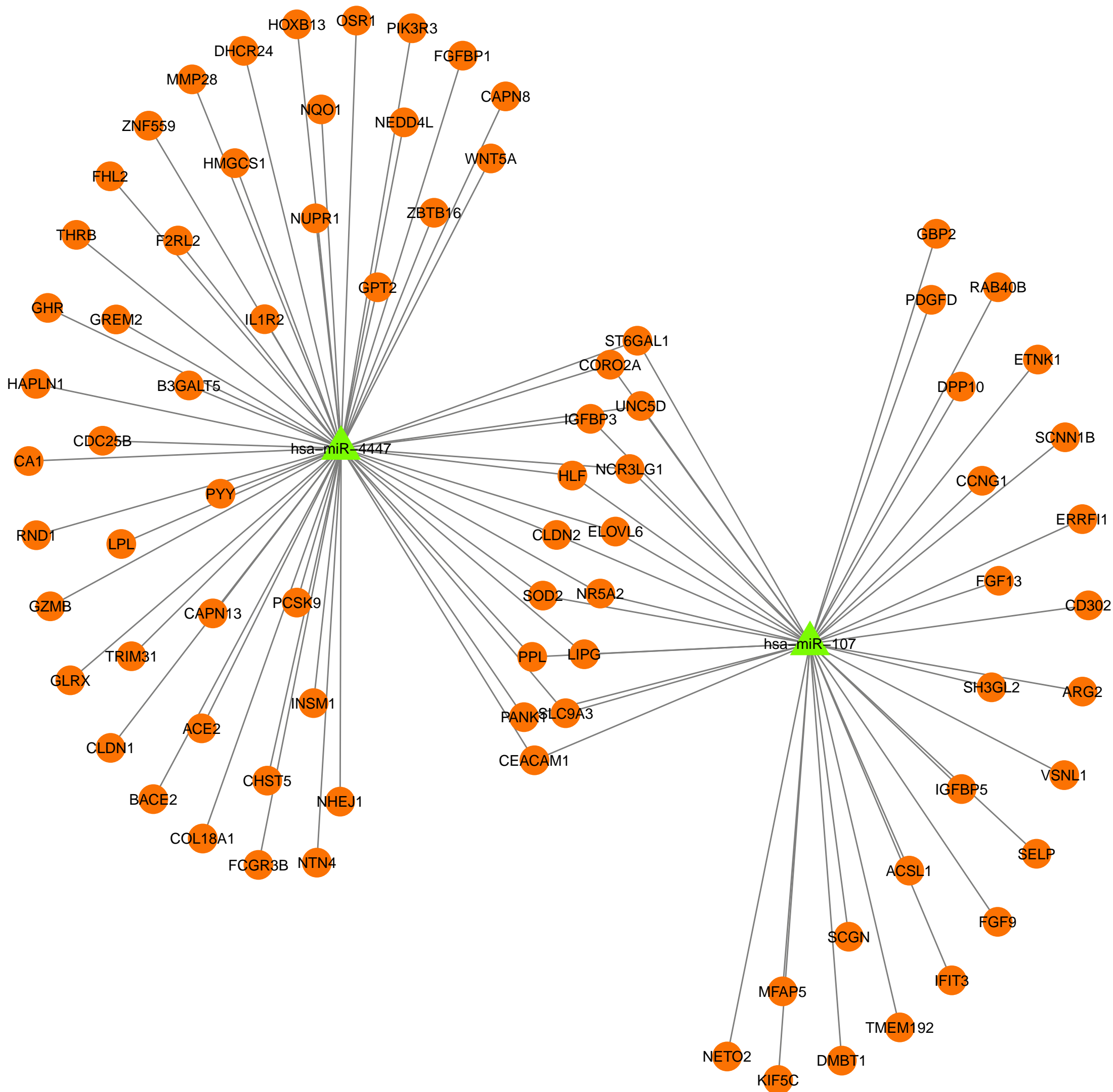

Supplement: Supplementary file 2 [file DataSheet4.zip › Input data and script3/ceRNAnet/Apoptosis_gene/netfile1.pdf]

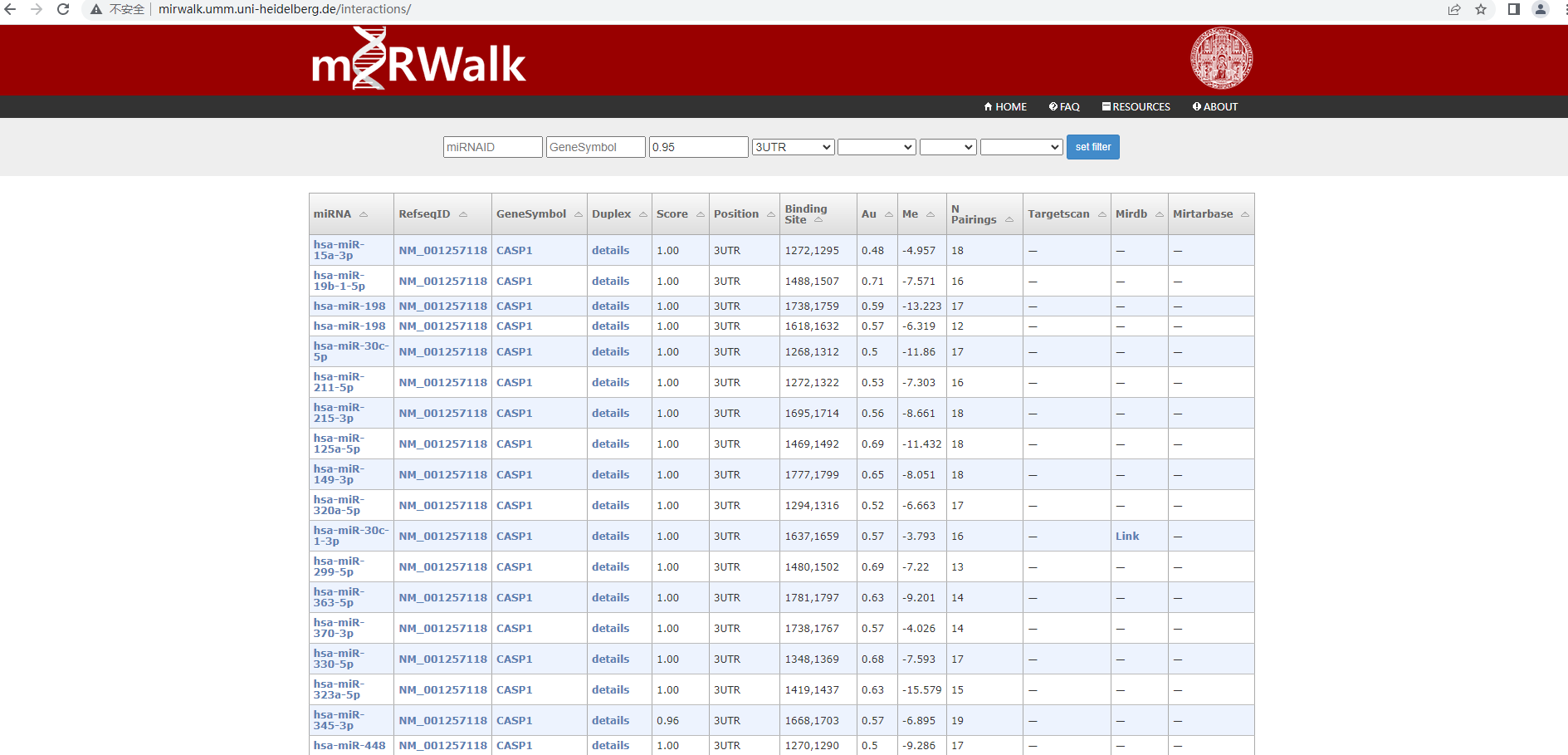

Supplement: Supplementary file 2 [file DataSheet4.zip › Input data and script3/ceRNAnet/Necroptosis_gene/σ▒Åσ╣òμê¬σ¢╛ 2022-04-15 153656.png]

**hsa-miR-4447**

**NQO1**

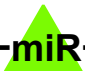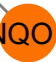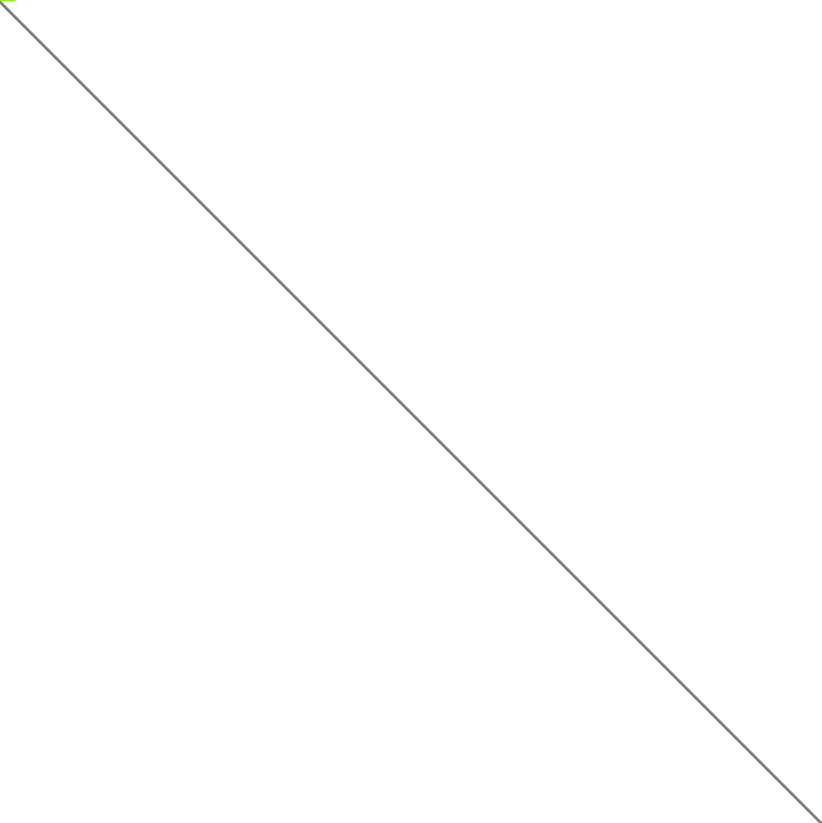

Supplement: Supplementary file 2 [file DataSheet4.zip › Input data and script3/ceRNAnet/Necroptosis_gene/net1.pdf]

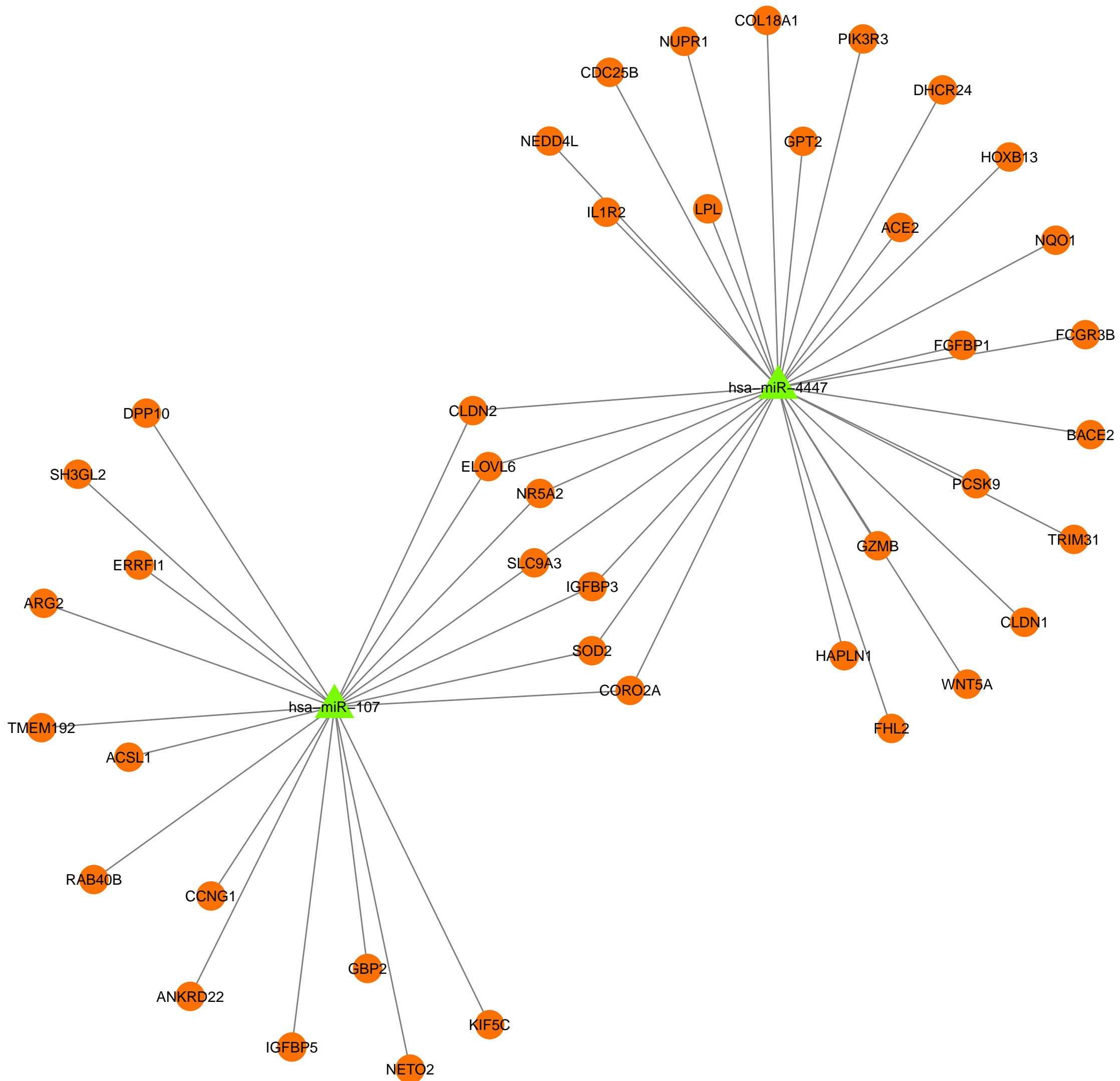

Supplement: Supplementary file 2 [file DataSheet4.zip › Input data and script3/ceRNAnet/Autophagy_gene/netfile1.pdf]

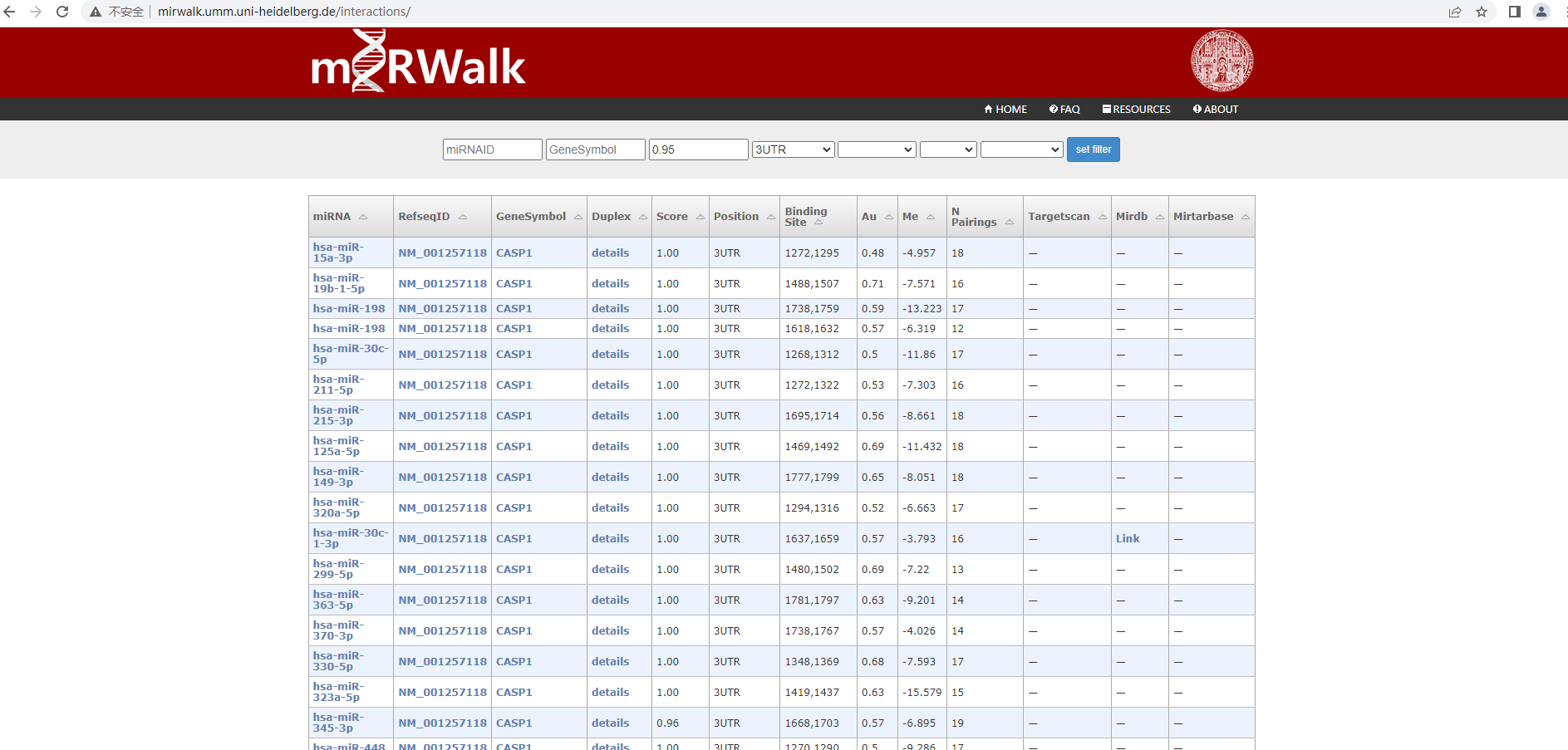

Supplement: Supplementary file 2 [file DataSheet4.zip › Input data and script3/ceRNAnet/Autophagy_gene/σ▒Åσ╣òμê¬σ¢╛ 2022-04-15 153911.png]

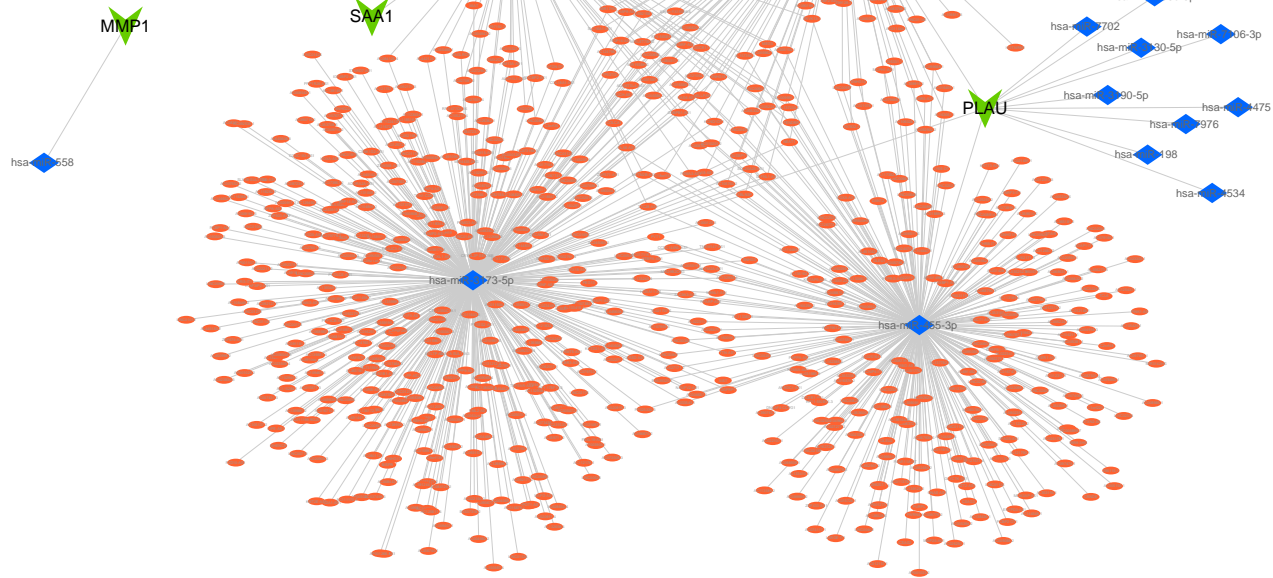

Supplement: Supplementary file 2 [file DataSheet4.zip › Input data and script3/ceRNAnet/GSE10616/netfile.txt.pdf]

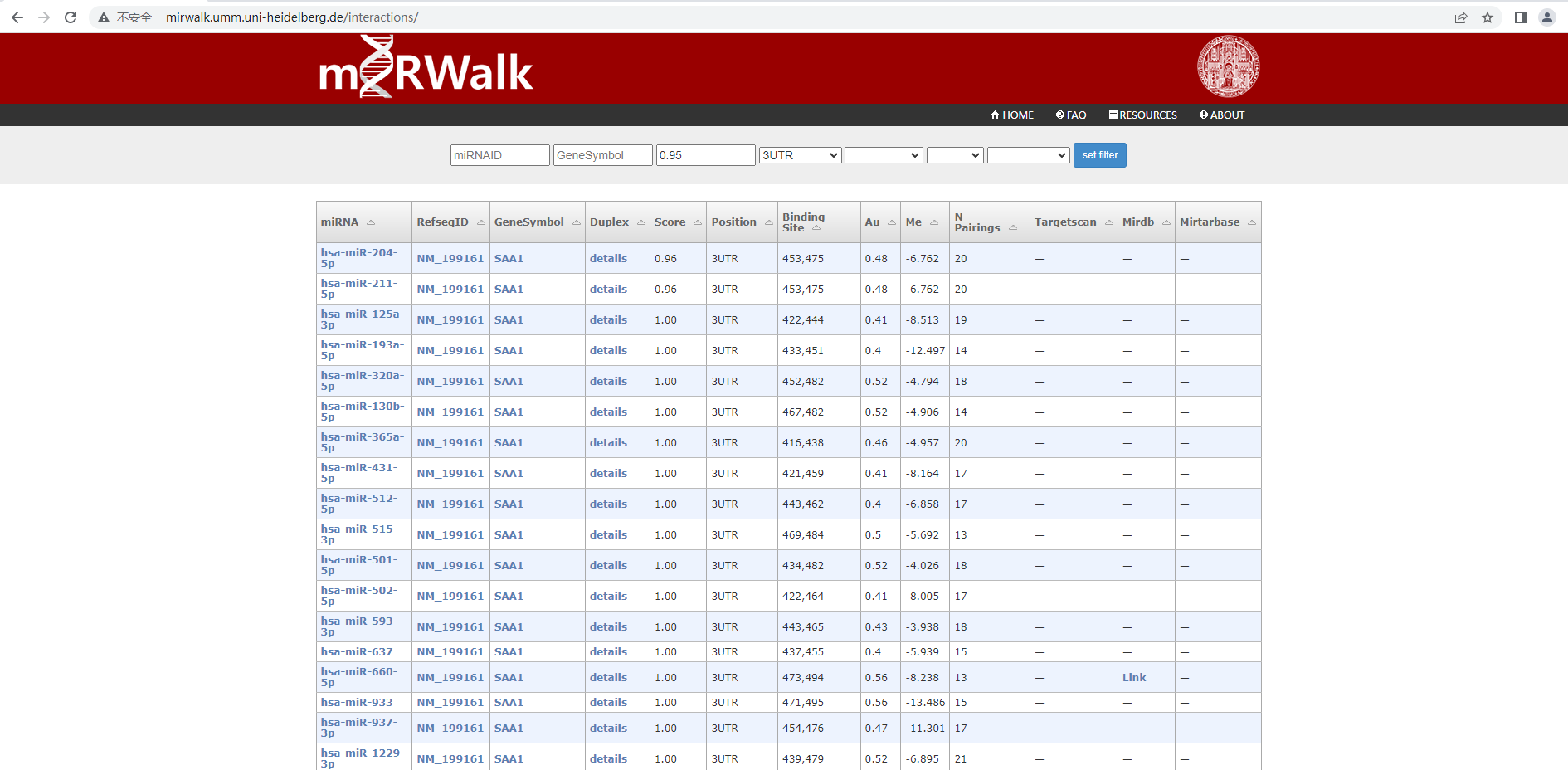

Supplement: Supplementary file 2 [file DataSheet4.zip › Input data and script3/ceRNAnet/GSE10616/σ▒Åσ╣òμê¬σ¢╛ 2022-04-08 095429.png]

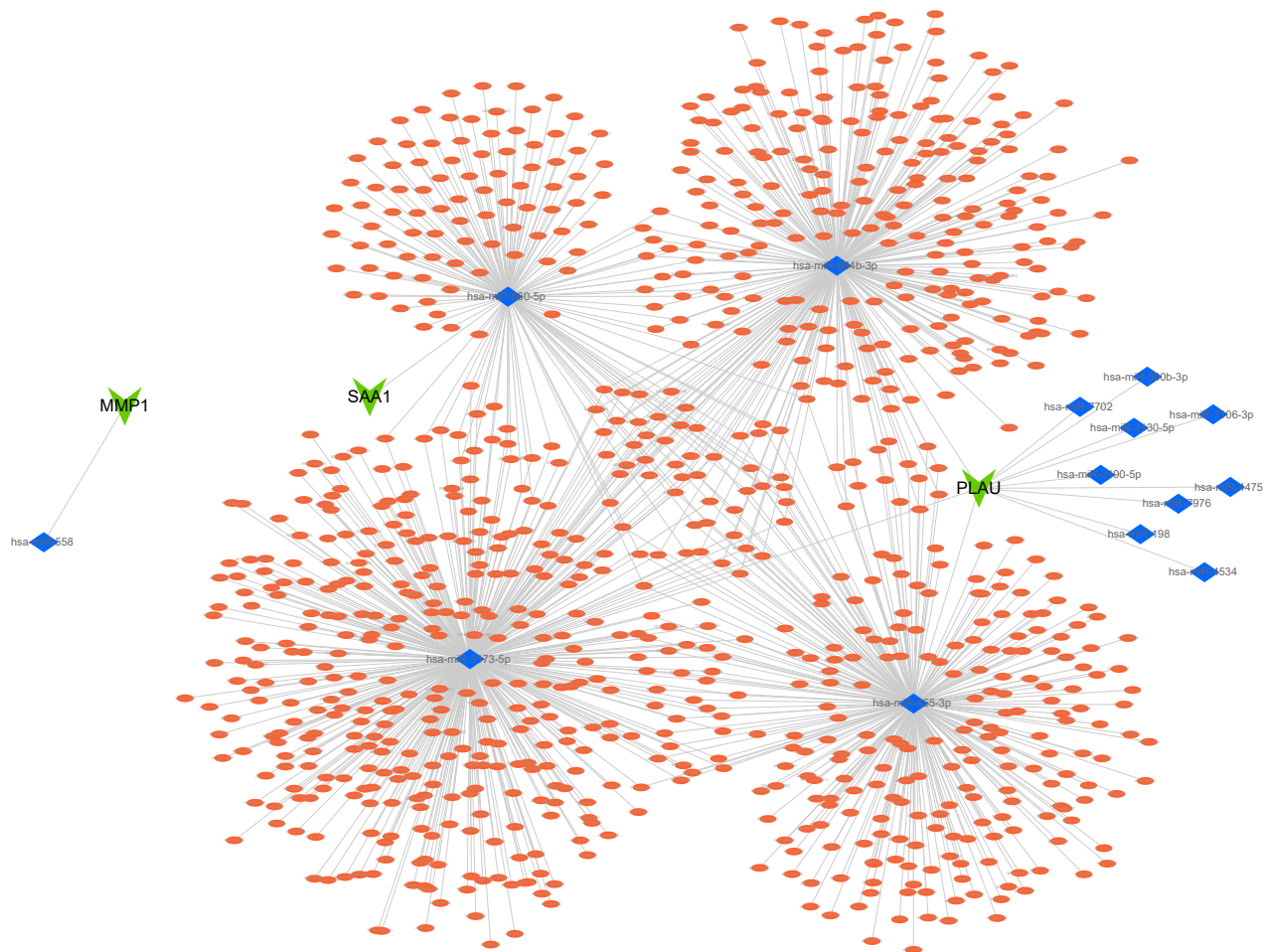

SAA1

Supplement: Supplementary file 2 [file DataSheet4.zip › Input data and script3/ceRNAnet/GSE10616/netfile.pdf]

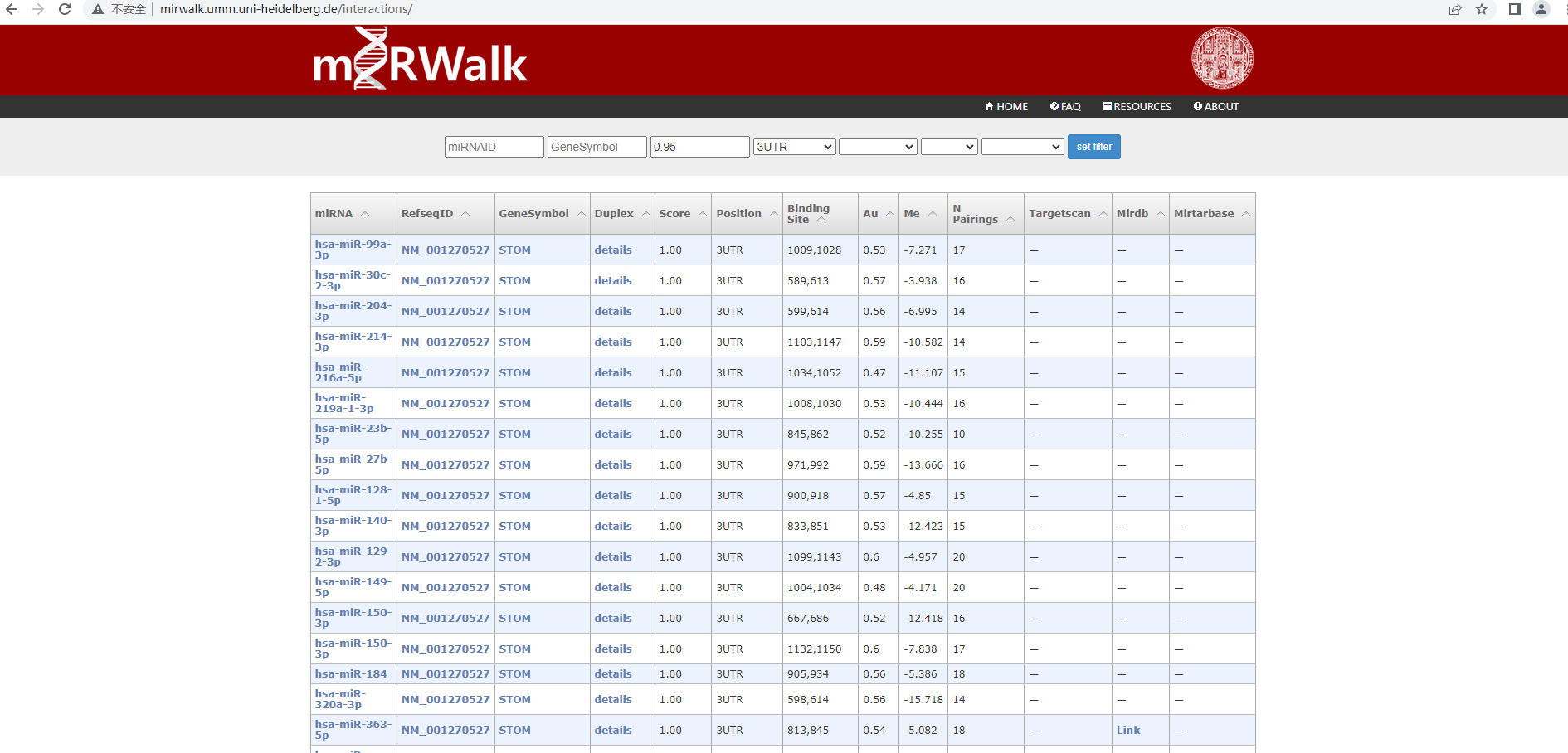

Supplement: Supplementary file 2 [file DataSheet4.zip › Input data and script3/ceRNAnet/Ferroptosis_gene/σ▒Åσ╣òμê¬σ¢╛ 2022-04-15 153757.png]

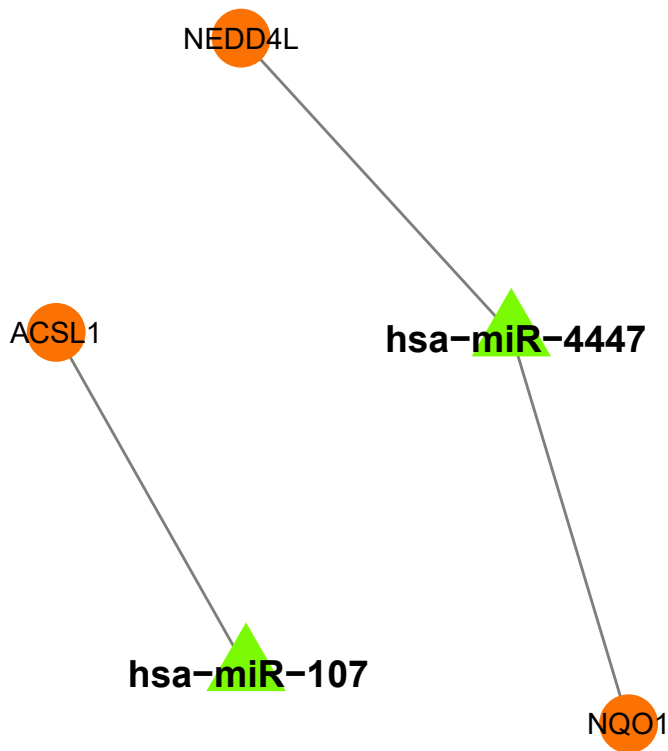

Supplement: Supplementary file 2 [file DataSheet4.zip › Input data and script3/ceRNAnet/Ferroptosis_gene/net1.pdf]
