# Supplementary figures and images for: Identification of hub programmed cell death-related genes and immune infiltration in Crohn’s disease using bioinformatics (part 1 of 2)
Source: Front Genet. 2024 Dec 18;15:1425062. doi: 10.3389/fgene.2024.1425062 (PMC11688285; doi:10.3389/fgene.2024.1425062)

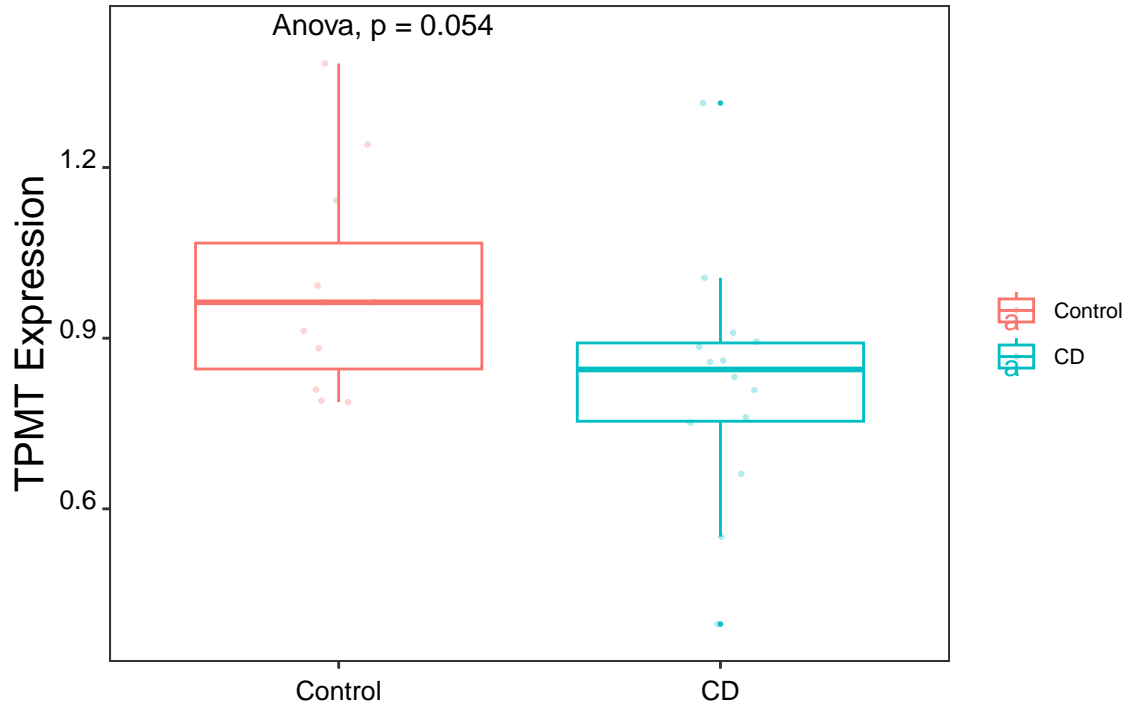

Supplement: Supplementary file 1 [file DataSheet3.zip › Input data and script2/DiseaseGene/TPMT.HealthyDisease.pdf]

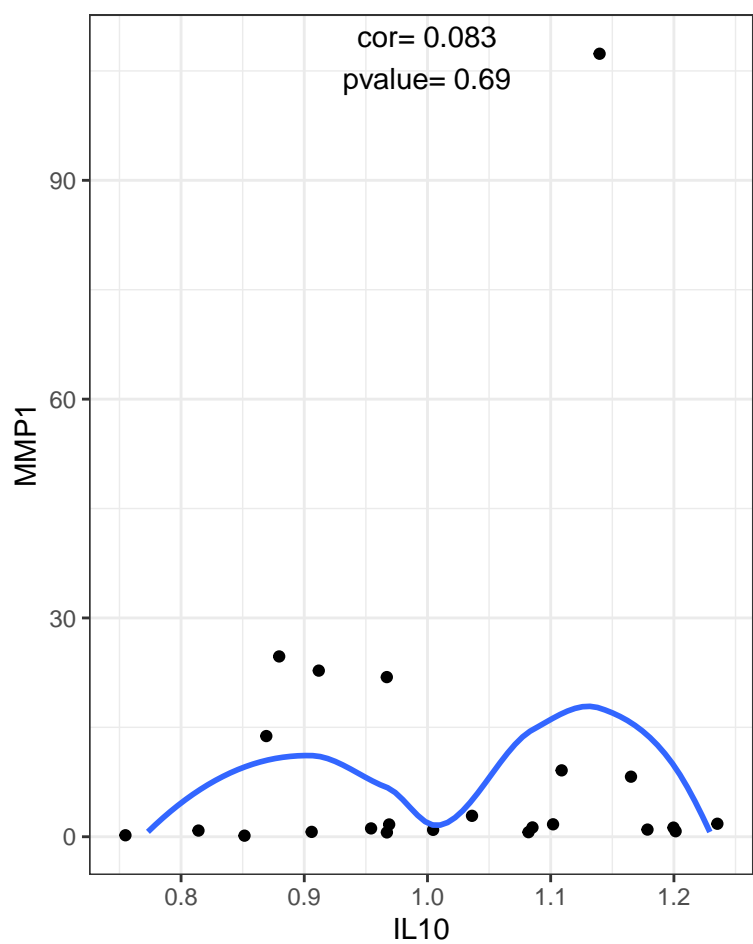

Supplement: Supplementary file 1 [file DataSheet3.zip › Input data and script2/DiseaseGene/MMP1 ~ IL10.pdf]

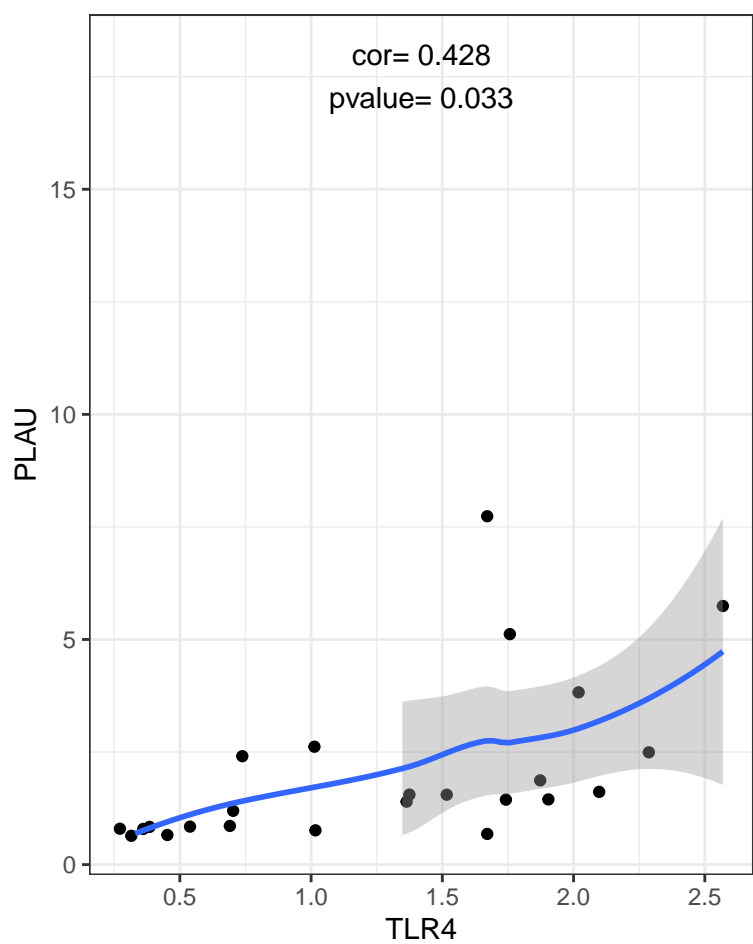

Supplement: Supplementary file 1 [file DataSheet3.zip › Input data and script2/DiseaseGene/PLAU ~ TLR4.pdf]

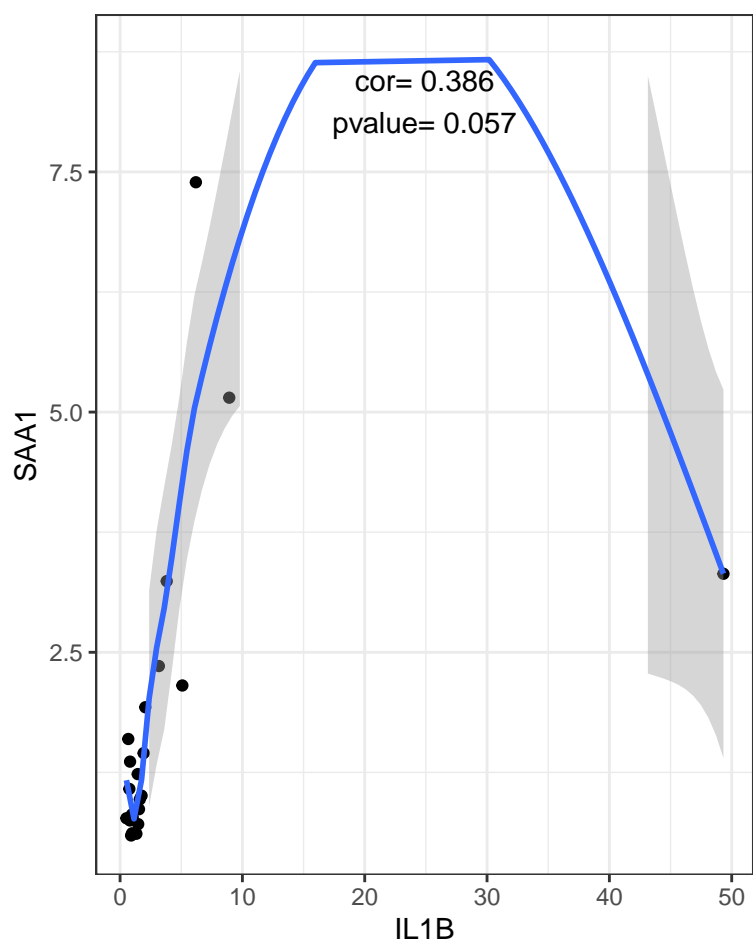

Supplement: Supplementary file 1 [file DataSheet3.zip › Input data and script2/DiseaseGene/SAA1 ~ IL1B.pdf]

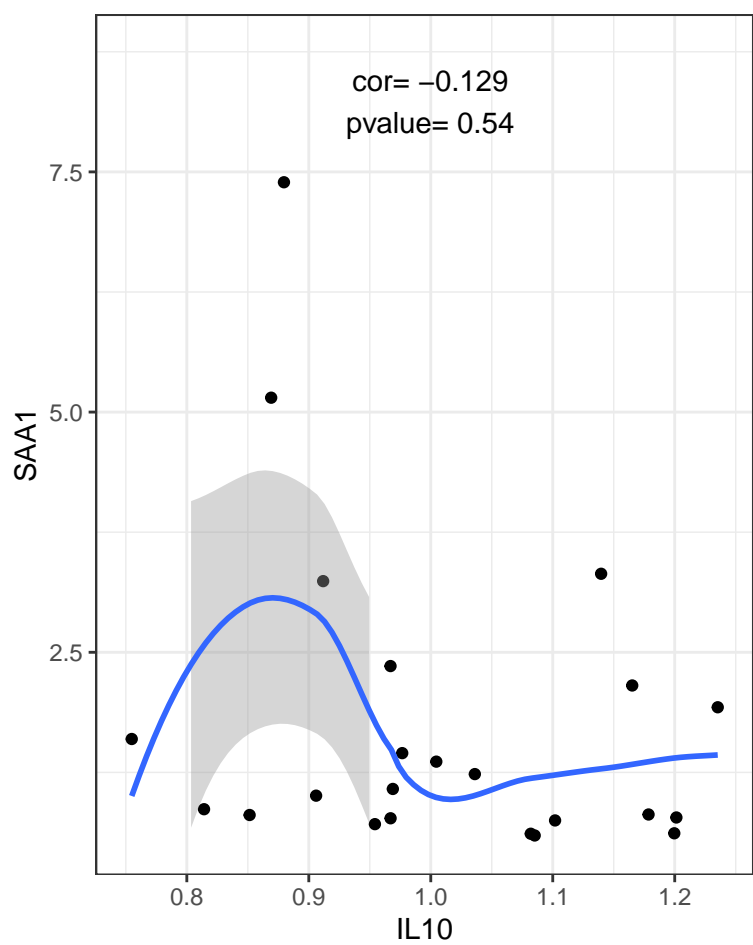

Supplement: Supplementary file 1 [file DataSheet3.zip › Input data and script2/DiseaseGene/SAA1 ~ IL10.pdf]

ATG16L1 Expression

Anova,  $p = 0.052$

Control

CD

Control  
CD

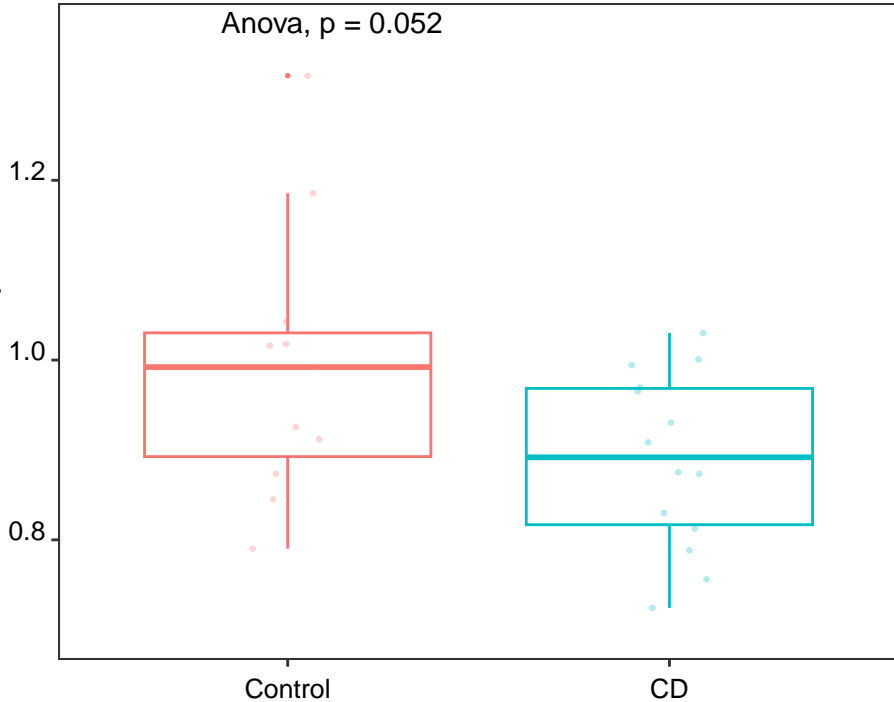

Supplement: Supplementary file 1 [file DataSheet3.zip › Input data and script2/DiseaseGene/ATG16L1.HealthyDisease.pdf]

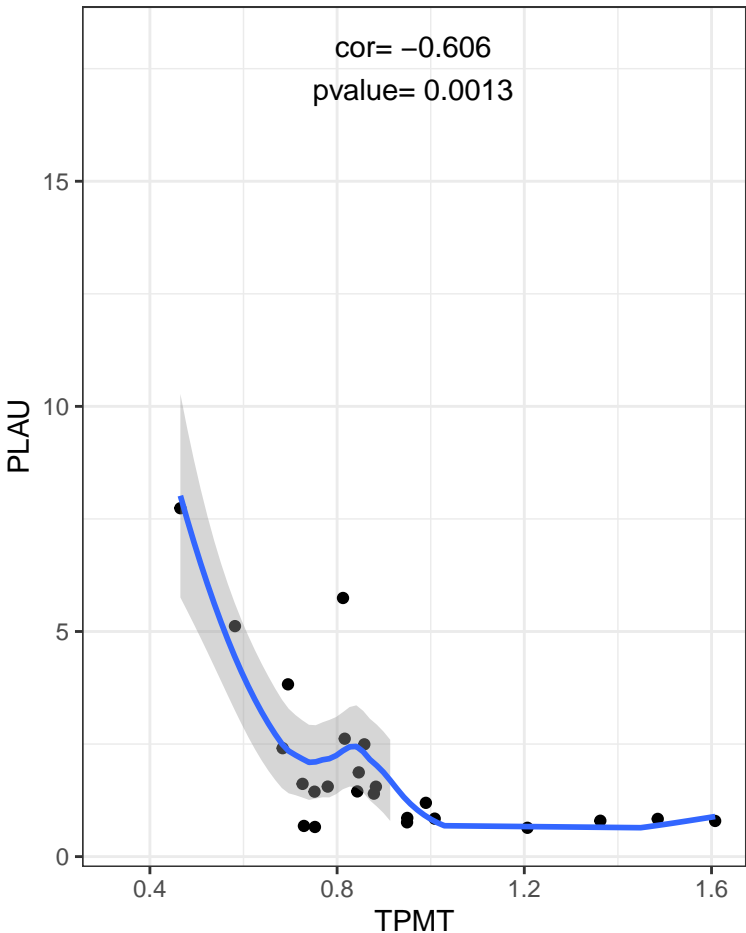

Supplement: Supplementary file 1 [file DataSheet3.zip › Input data and script2/DiseaseGene/PLAU ~ TPMT.pdf]

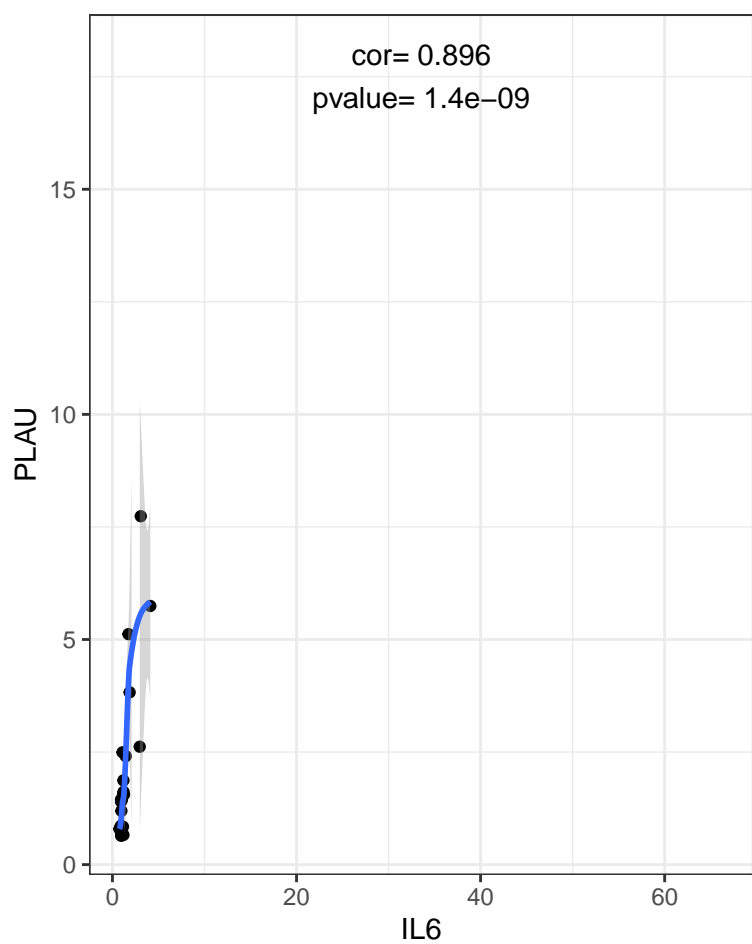

Supplement: Supplementary file 1 [file DataSheet3.zip › Input data and script2/DiseaseGene/PLAU ~ IL6.pdf]

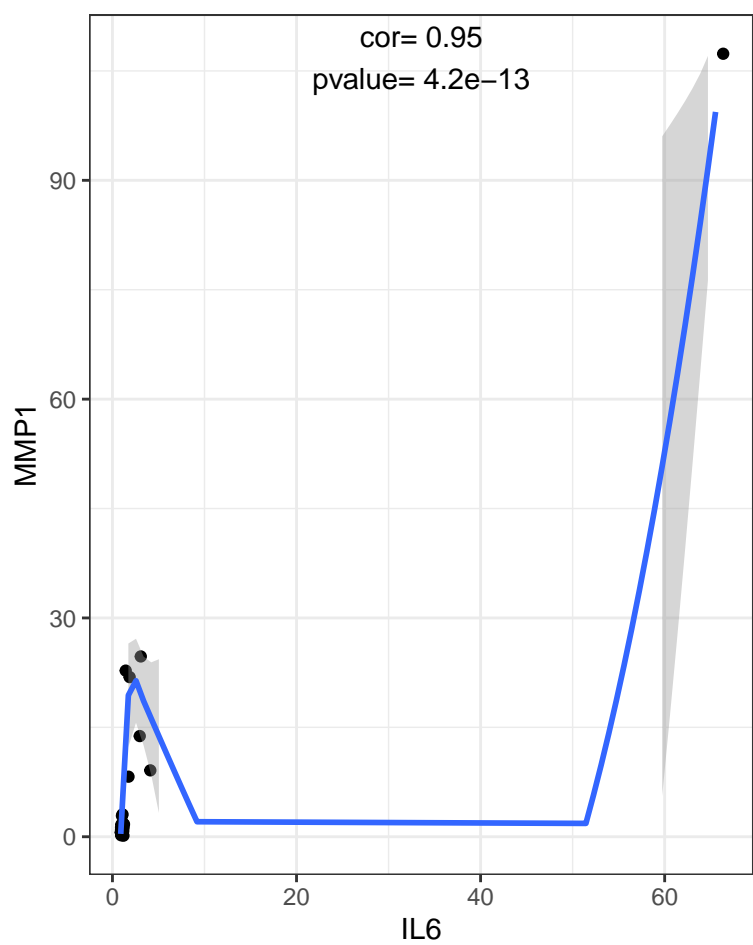

Supplement: Supplementary file 1 [file DataSheet3.zip › Input data and script2/DiseaseGene/MMP1 ~ IL6.pdf]

Expression level

Tissue Control CD

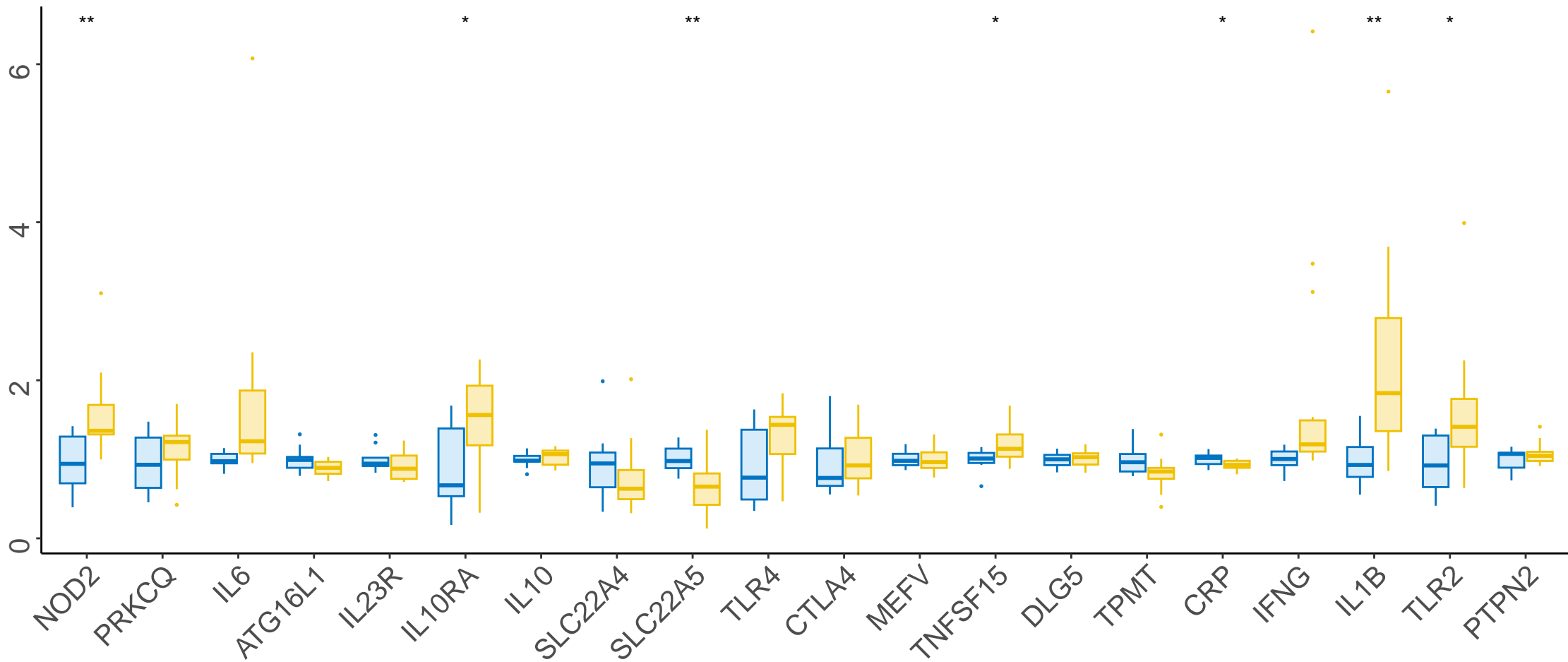

Supplement: Supplementary file 1 [file DataSheet3.zip › Input data and script2/DiseaseGene/Expression level.pdf]

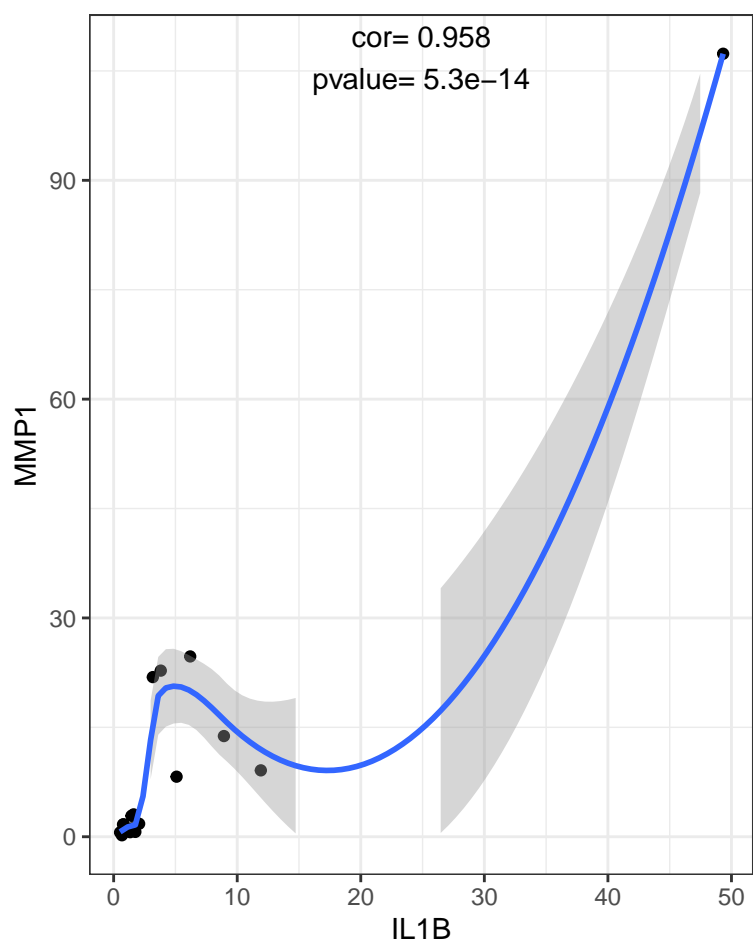

Supplement: Supplementary file 1 [file DataSheet3.zip › Input data and script2/DiseaseGene/MMP1 ~ IL1B.pdf]

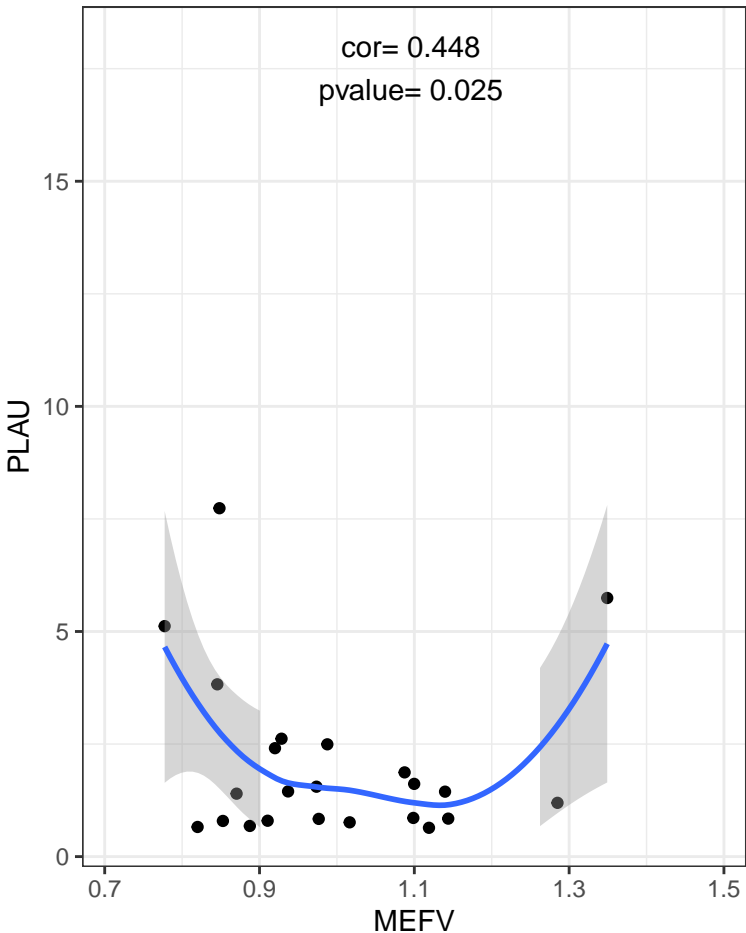

Supplement: Supplementary file 1 [file DataSheet3.zip › Input data and script2/DiseaseGene/PLAU ~ MEFV.pdf]

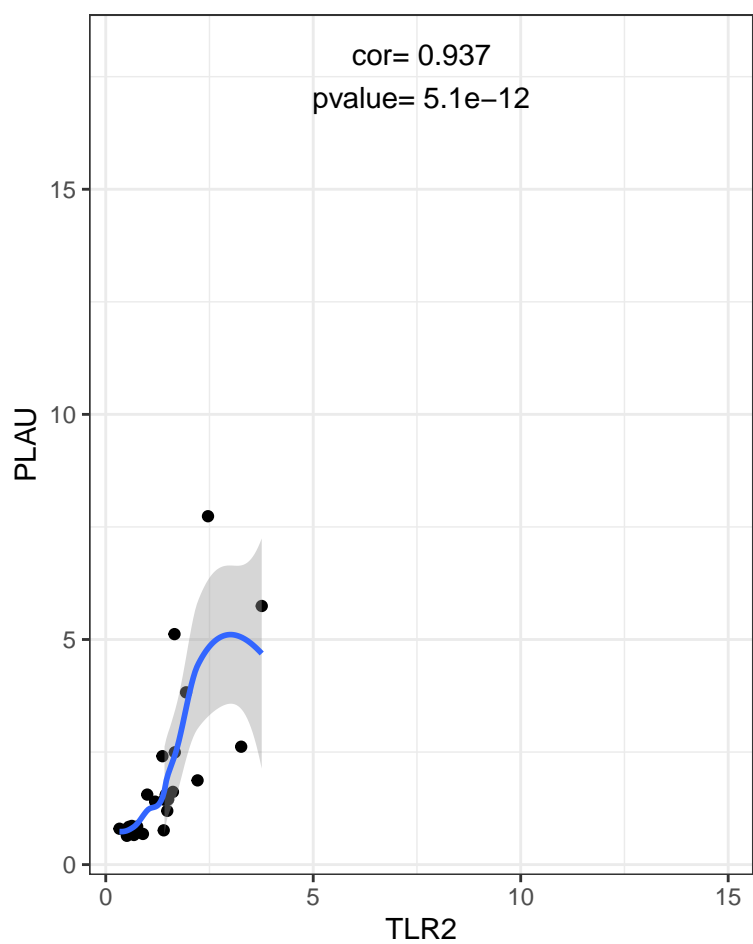

Supplement: Supplementary file 1 [file DataSheet3.zip › Input data and script2/DiseaseGene/PLAU ~ TLR2.pdf]

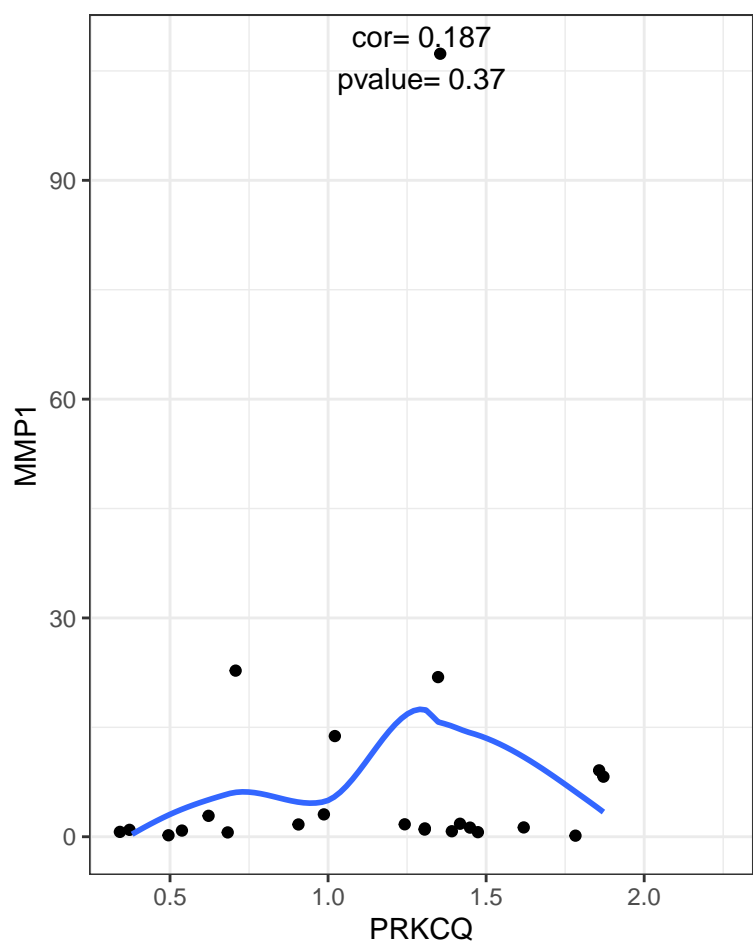

Supplement: Supplementary file 1 [file DataSheet3.zip › Input data and script2/DiseaseGene/MMP1 ~ PRKCQ.pdf]

SLC22A5 Expression

Anova,  $p = 0.0093$

Control

CD

Control  
CD

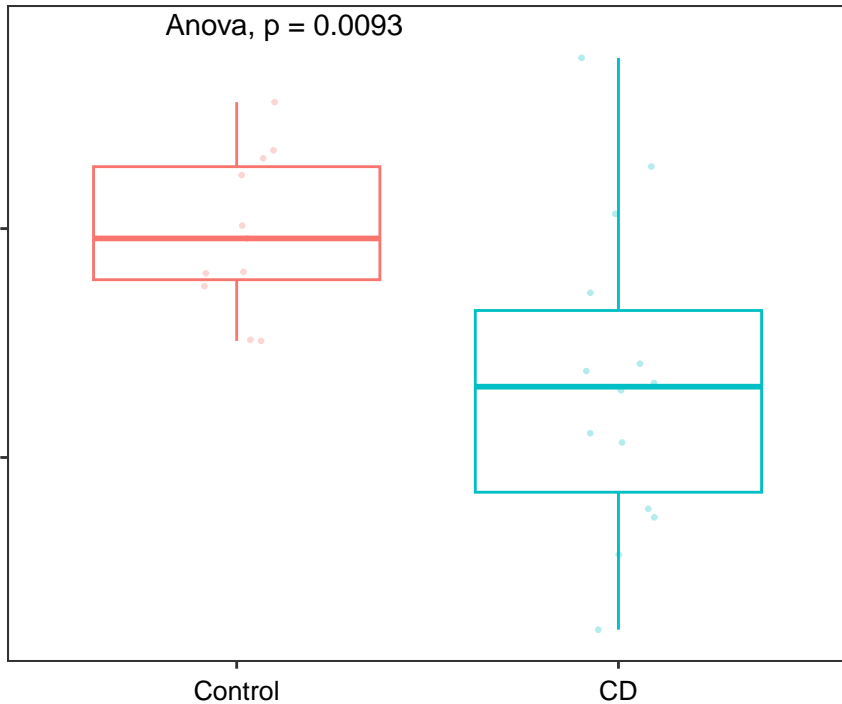

Supplement: Supplementary file 1 [file DataSheet3.zip › Input data and script2/DiseaseGene/SLC22A5.HealthyDisease.pdf]

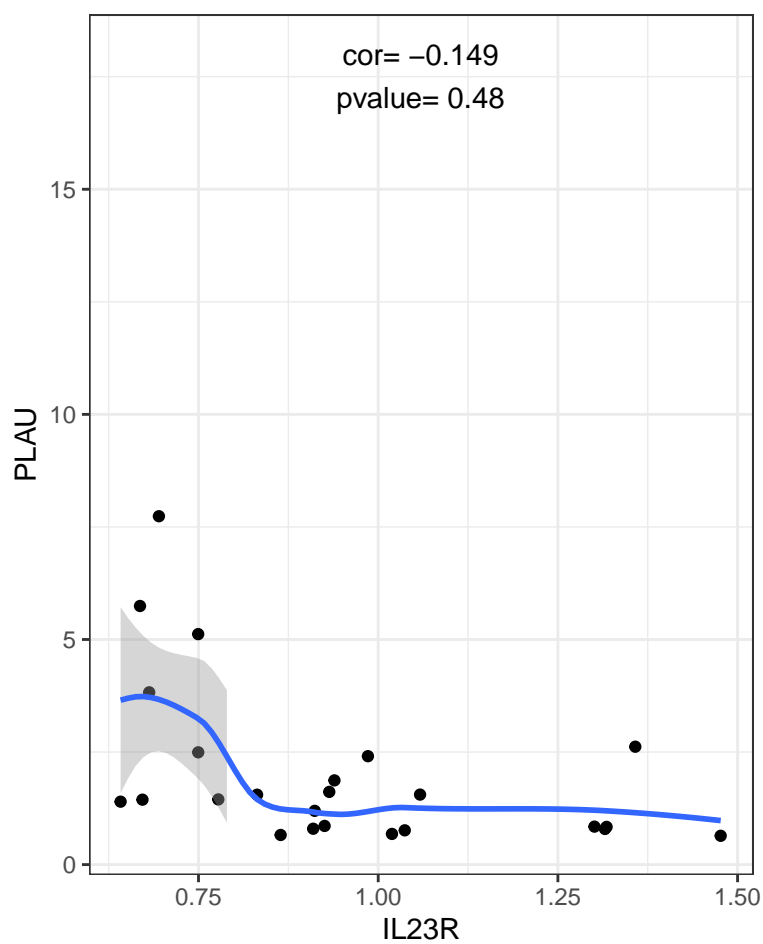

Supplement: Supplementary file 1 [file DataSheet3.zip › Input data and script2/DiseaseGene/PLAU ~ IL23R.pdf]

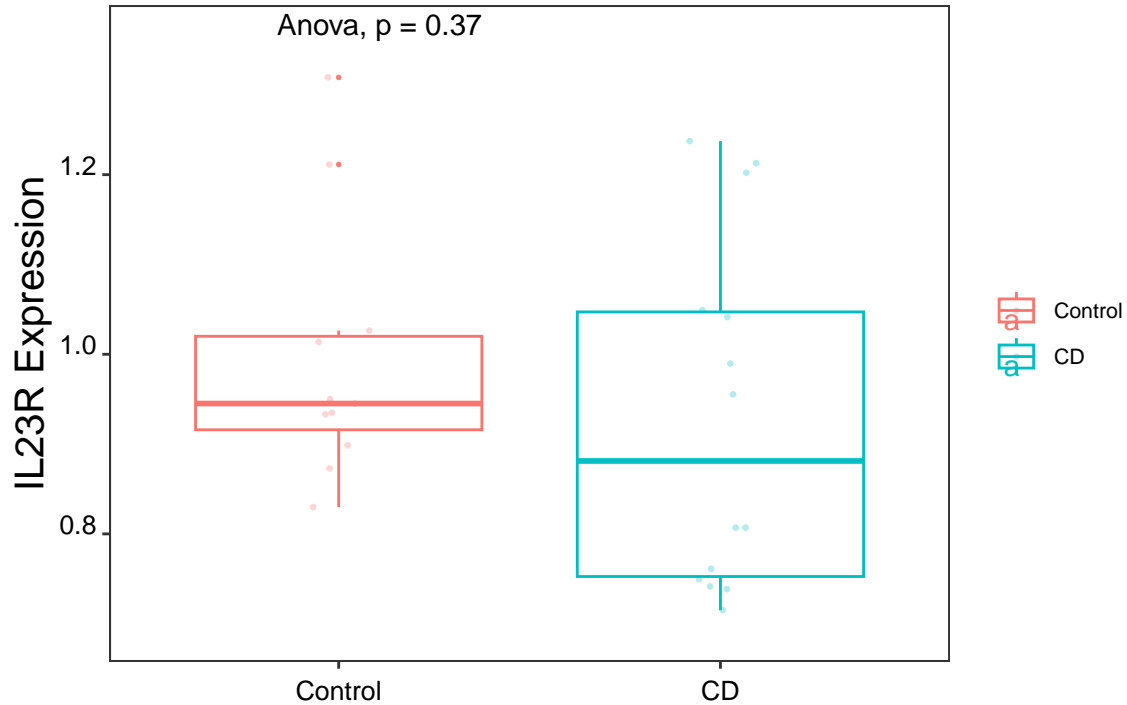

Supplement: Supplementary file 1 [file DataSheet3.zip › Input data and script2/DiseaseGene/IL23R.HealthyDisease.pdf]

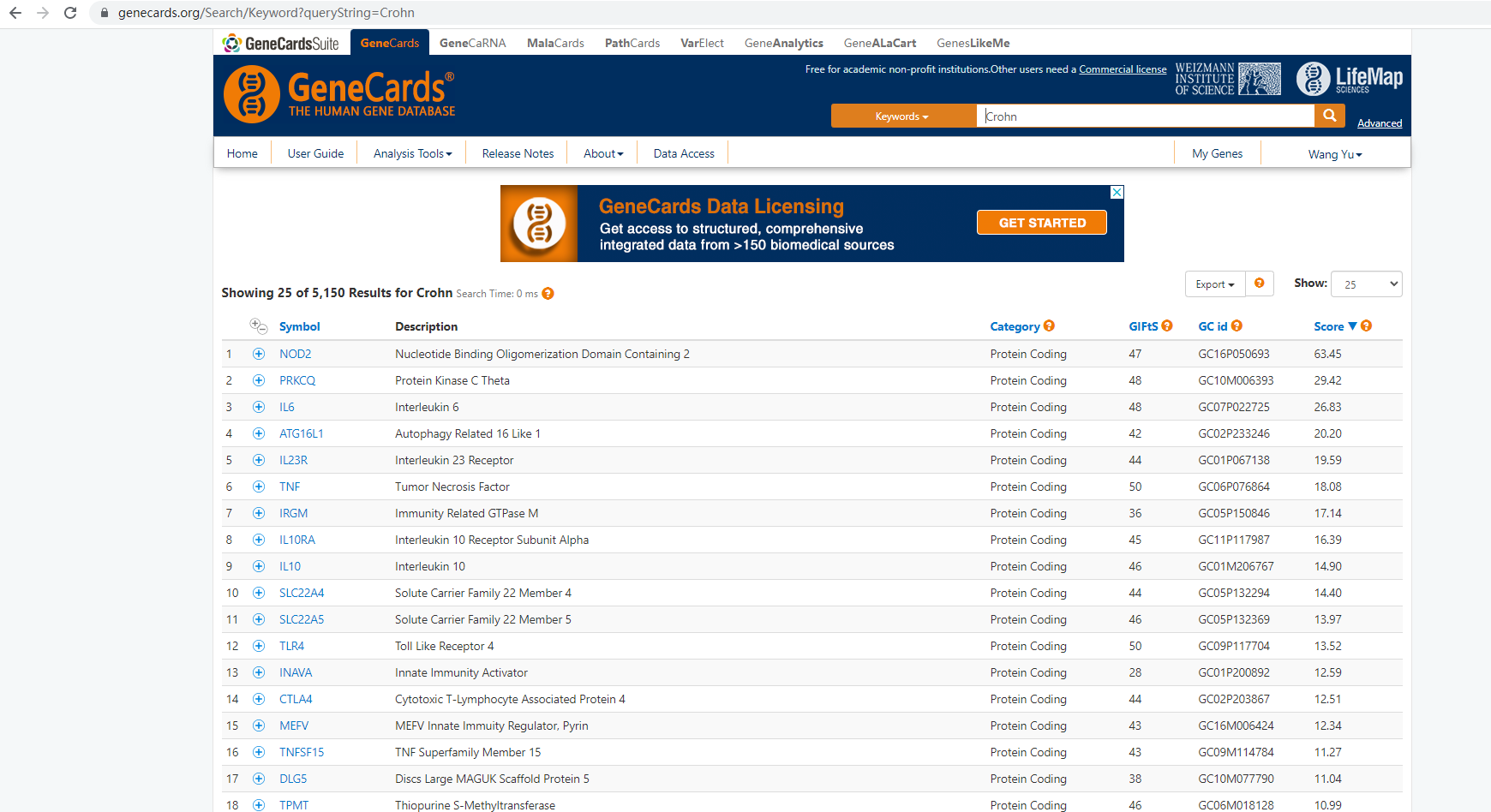

Supplement: Supplementary file 1 [file DataSheet3.zip › Input data and script2/DiseaseGene/σ▒Åσ╣òμê¬σ¢╛ 2022-04-07 092532.png]

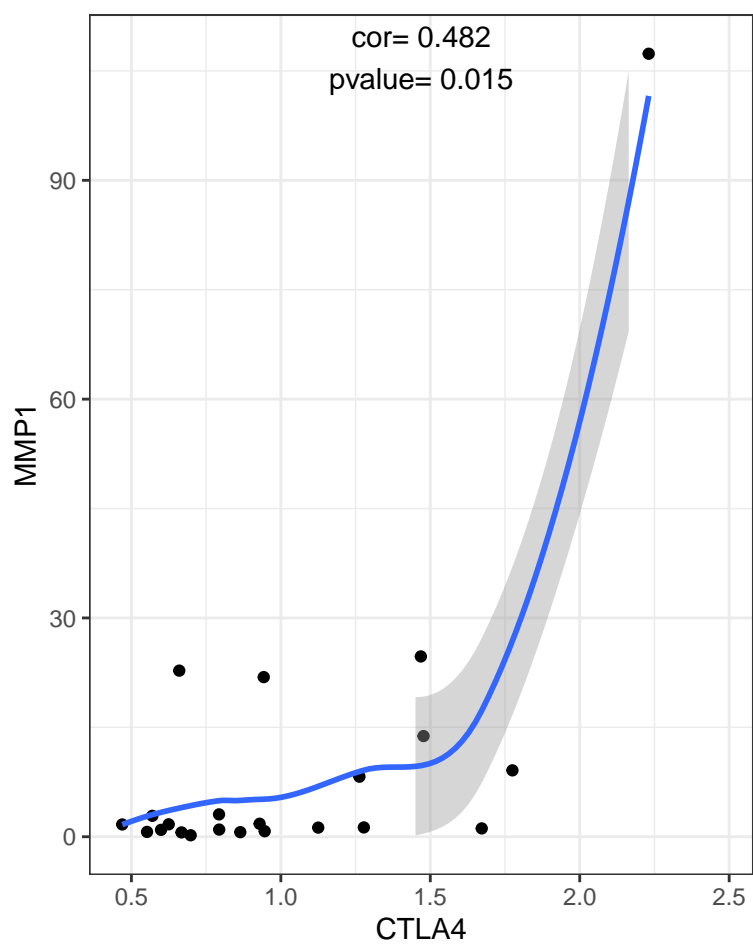

Supplement: Supplementary file 1 [file DataSheet3.zip › Input data and script2/DiseaseGene/MMP1 ~ CTLA4.pdf]

Anova,  $p = 0.0026$

PLAU Expression

4

3

2

1

Control

CD

Control  
CD

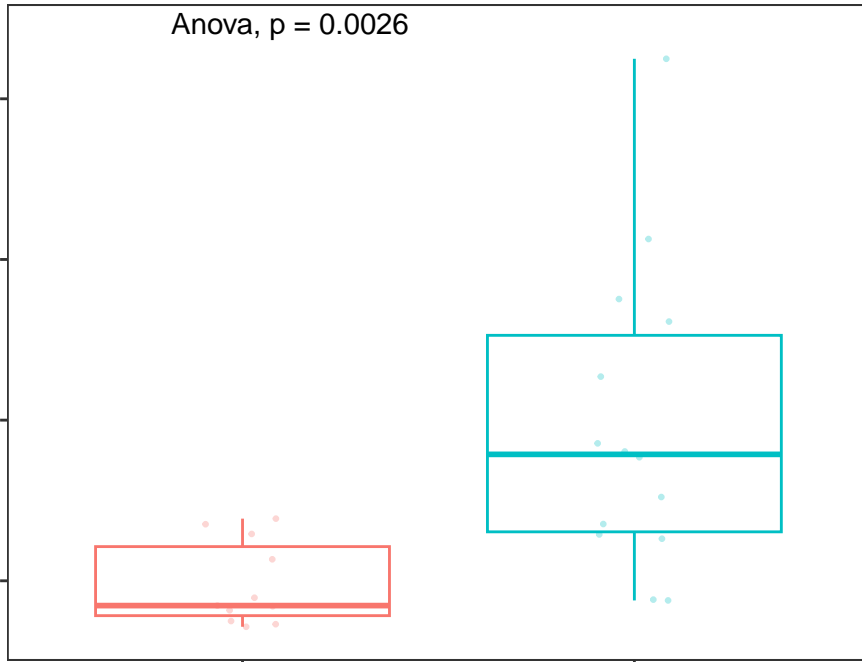

Supplement: Supplementary file 1 [file DataSheet3.zip › Input data and script2/DiseaseGene/PLAU.HealthyDisease.pdf]

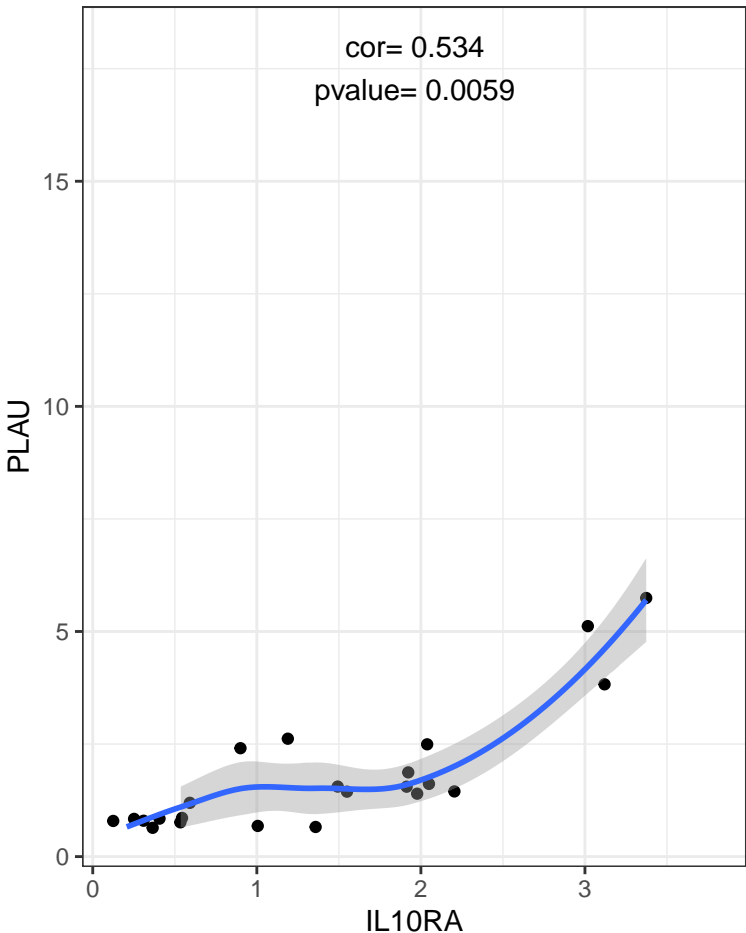

Supplement: Supplementary file 1 [file DataSheet3.zip › Input data and script2/DiseaseGene/PLAU ~ IL10RA.pdf]

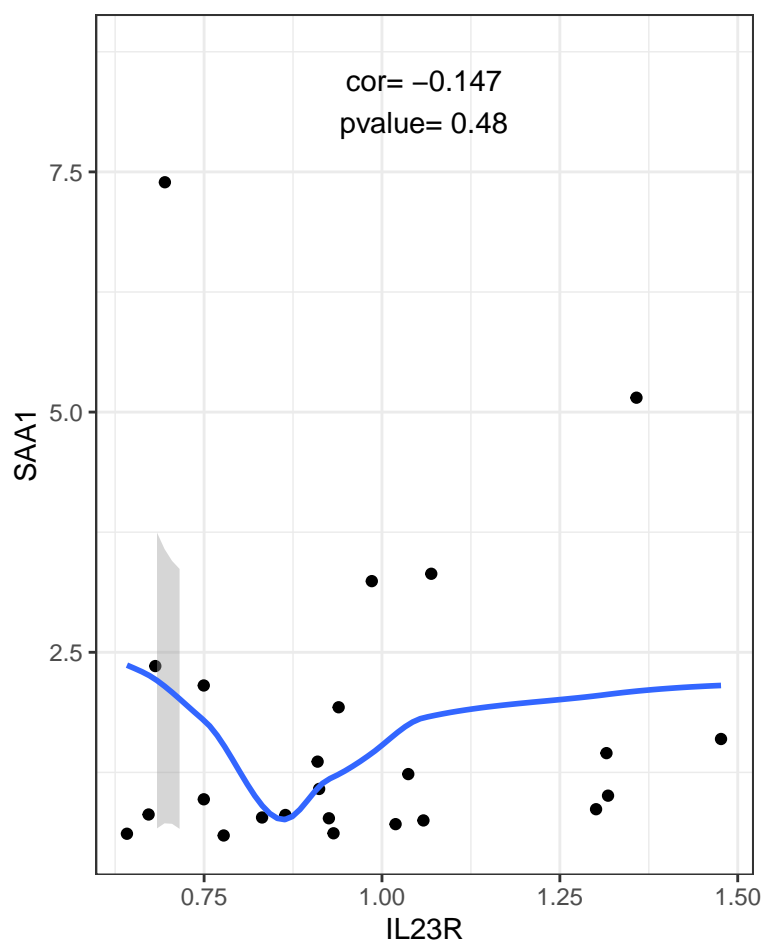

Supplement: Supplementary file 1 [file DataSheet3.zip › Input data and script2/DiseaseGene/SAA1 ~ IL23R.pdf]

CTLA4 Expression

Anova,  $p = 0.7$

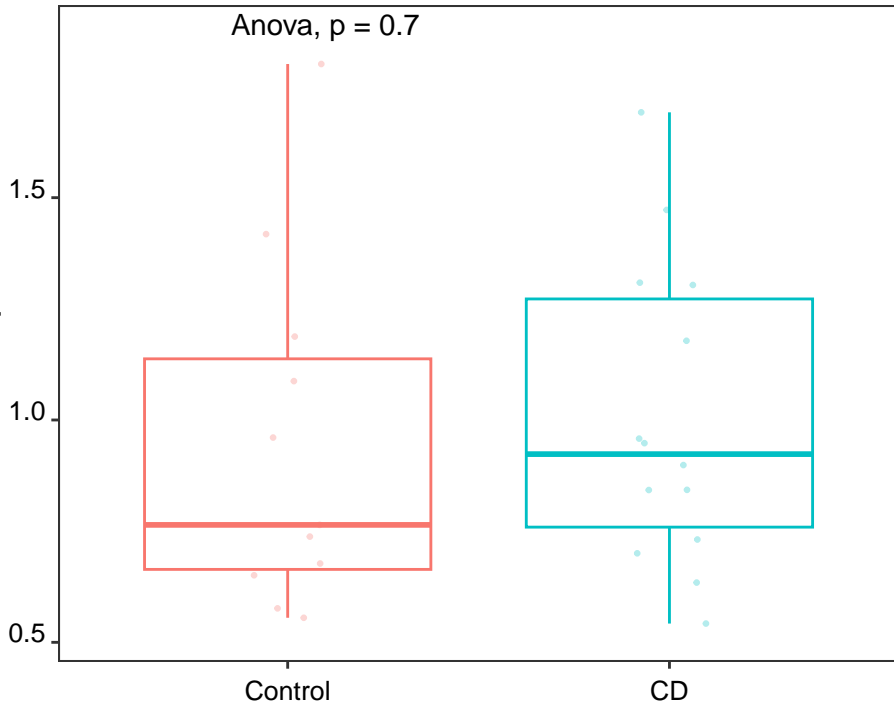

Control  
CD

Supplement: Supplementary file 1 [file DataSheet3.zip › Input data and script2/DiseaseGene/CTLA4.HealthyDisease.pdf]

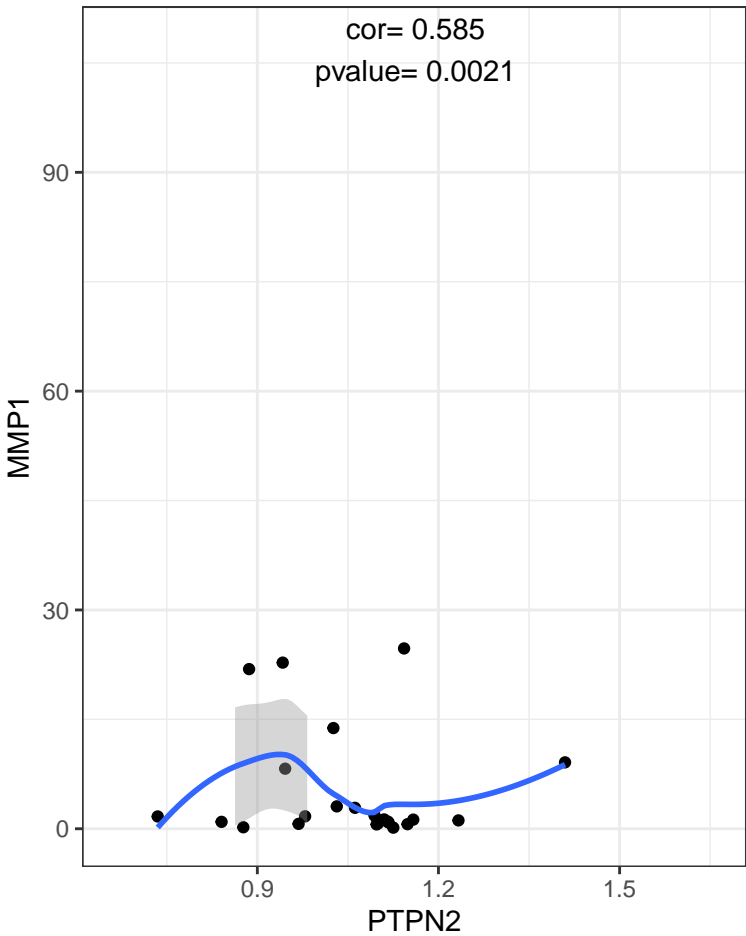

Supplement: Supplementary file 1 [file DataSheet3.zip › Input data and script2/DiseaseGene/MMP1 ~ PTPN2.pdf]

Tissue Control CD

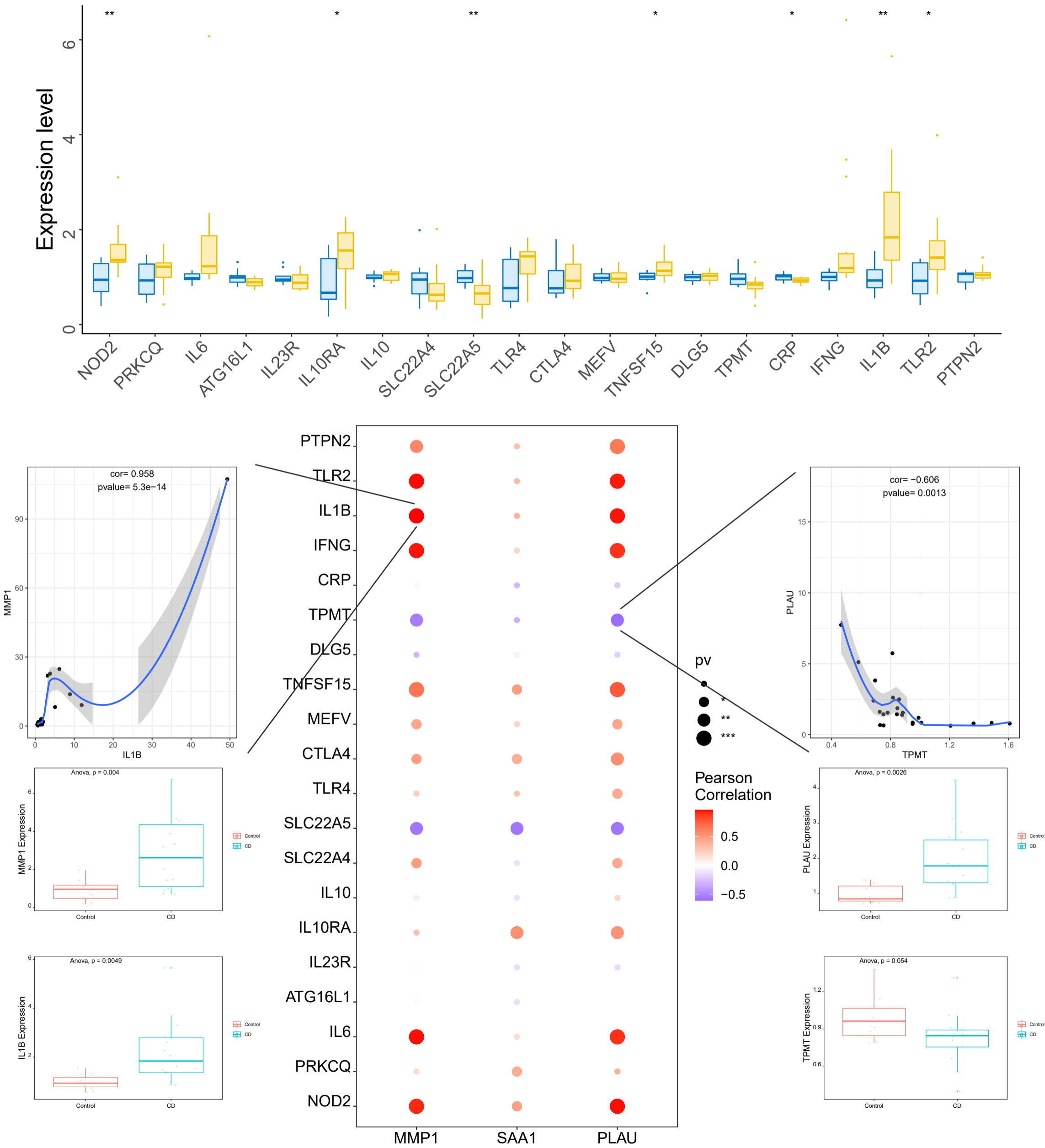

Supplement: Supplementary file 1 [file DataSheet3.zip › Input data and script2/DiseaseGene/Disease gene Expression level.pdf]

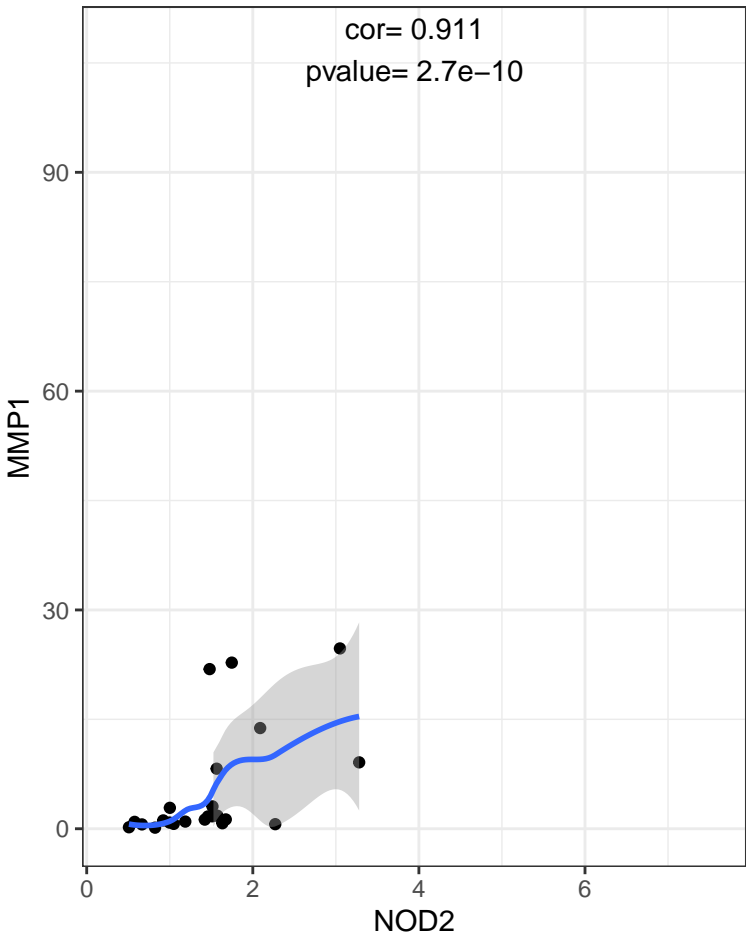

Supplement: Supplementary file 1 [file DataSheet3.zip › Input data and script2/DiseaseGene/MMP1 ~ NOD2.pdf]

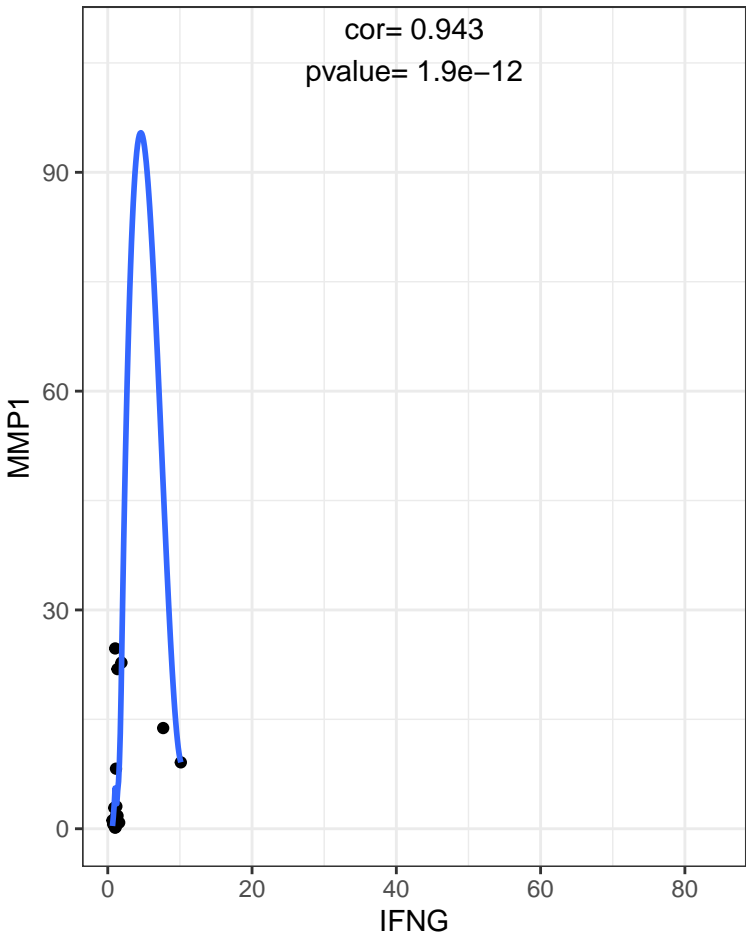

Supplement: Supplementary file 1 [file DataSheet3.zip › Input data and script2/DiseaseGene/MMP1 ~ IFNG.pdf]

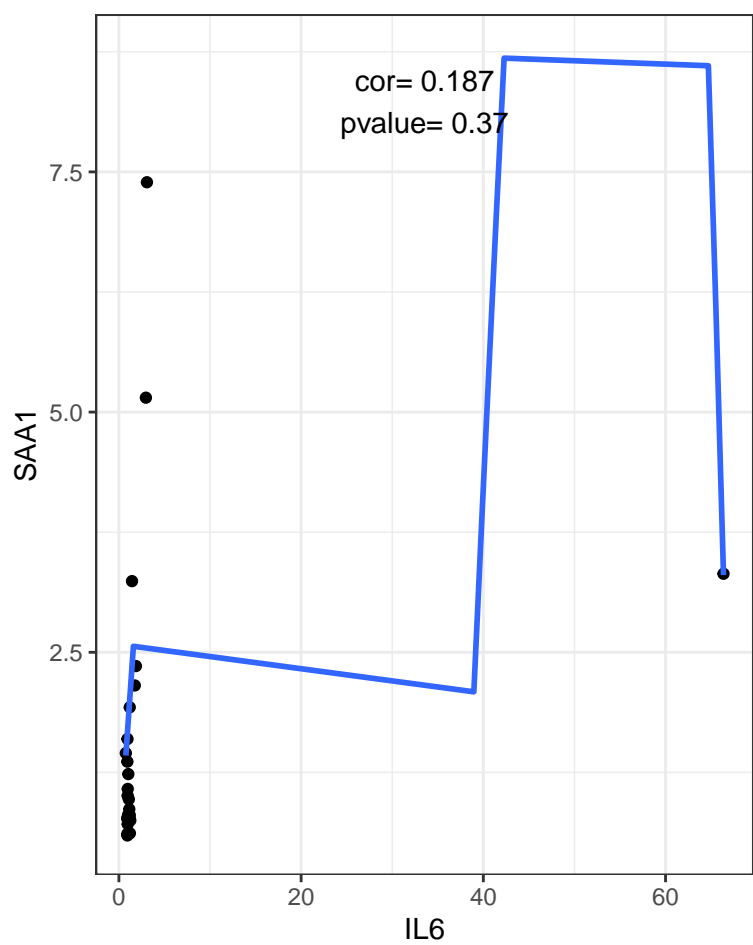

Supplement: Supplementary file 1 [file DataSheet3.zip › Input data and script2/DiseaseGene/SAA1 ~ IL6.pdf]

Anova,  $p = 0.077$

IFNG Expression

6

4

2

Control

CD

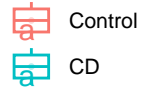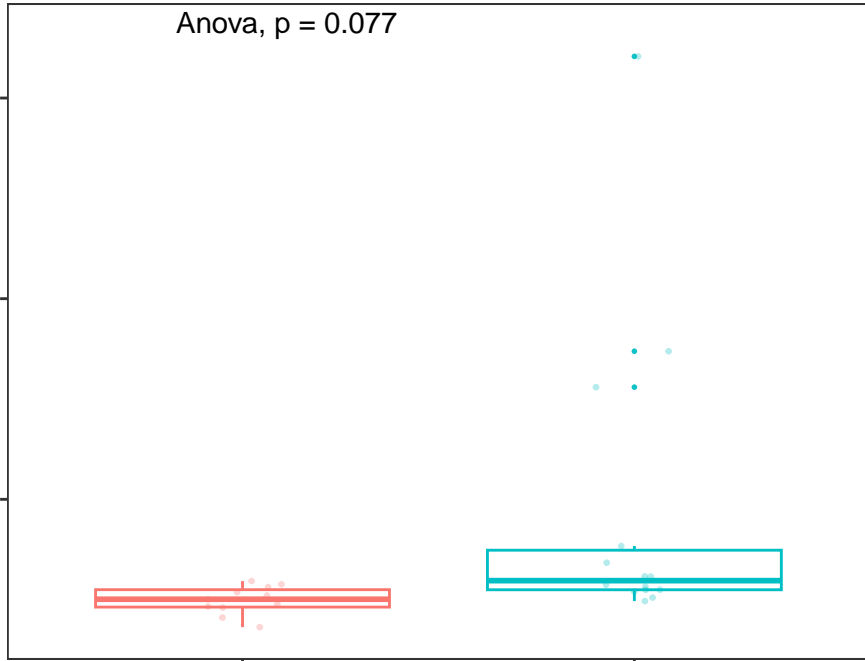

Supplement: Supplementary file 1 [file DataSheet3.zip › Input data and script2/DiseaseGene/IFNG.HealthyDisease.pdf]

Anova,  $p = 0.023$

TNFSF15 Expression

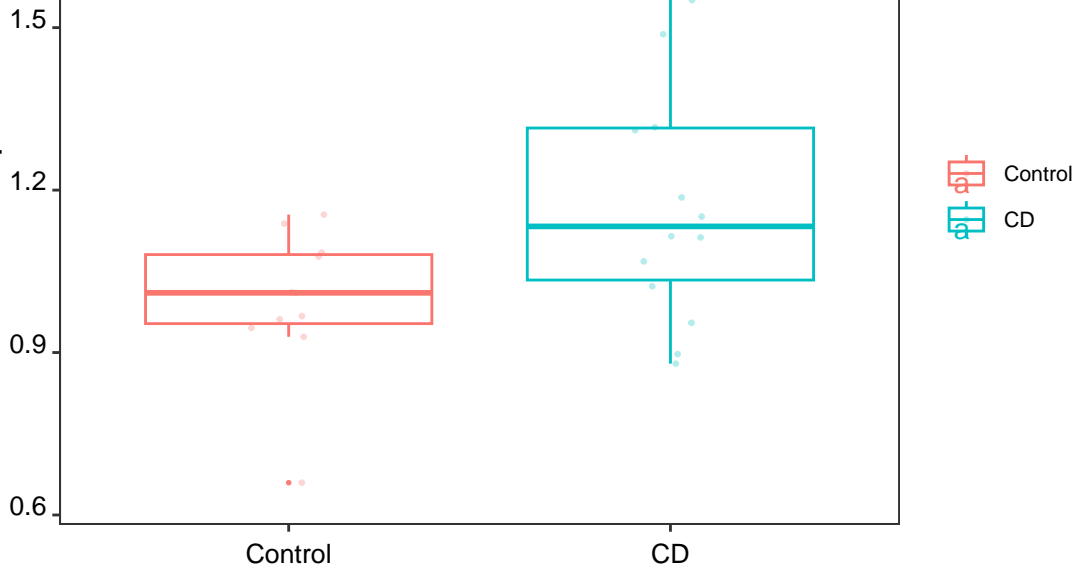

Supplement: Supplementary file 1 [file DataSheet3.zip › Input data and script2/DiseaseGene/TNFSF15.HealthyDisease.pdf]

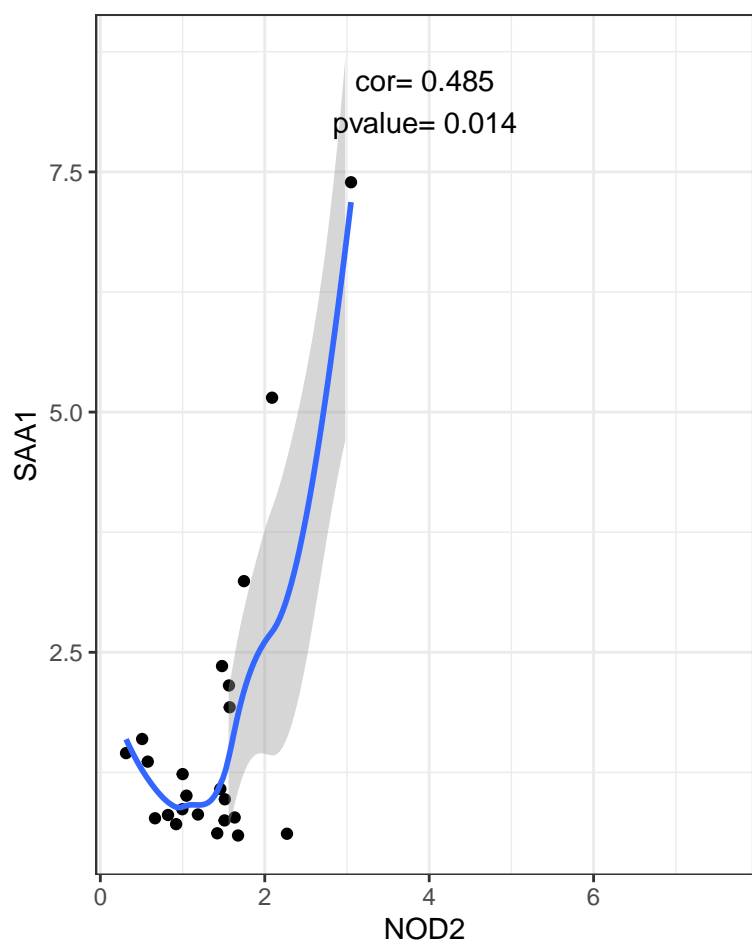

Supplement: Supplementary file 1 [file DataSheet3.zip › Input data and script2/DiseaseGene/SAA1 ~ NOD2.pdf]

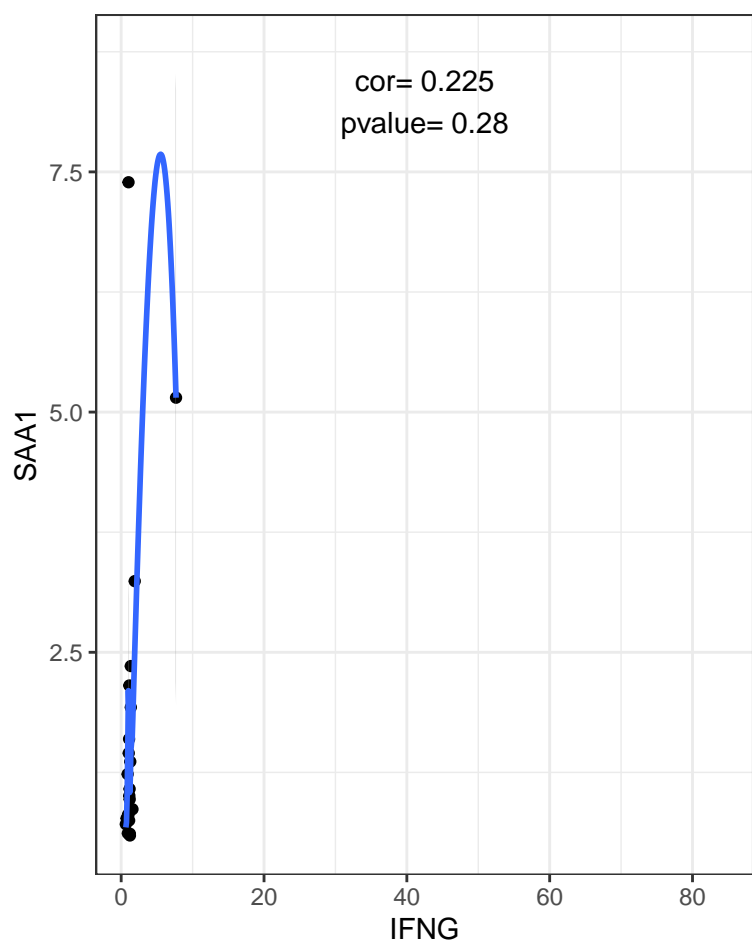

Supplement: Supplementary file 1 [file DataSheet3.zip › Input data and script2/DiseaseGene/SAA1 ~ IFNG.pdf]

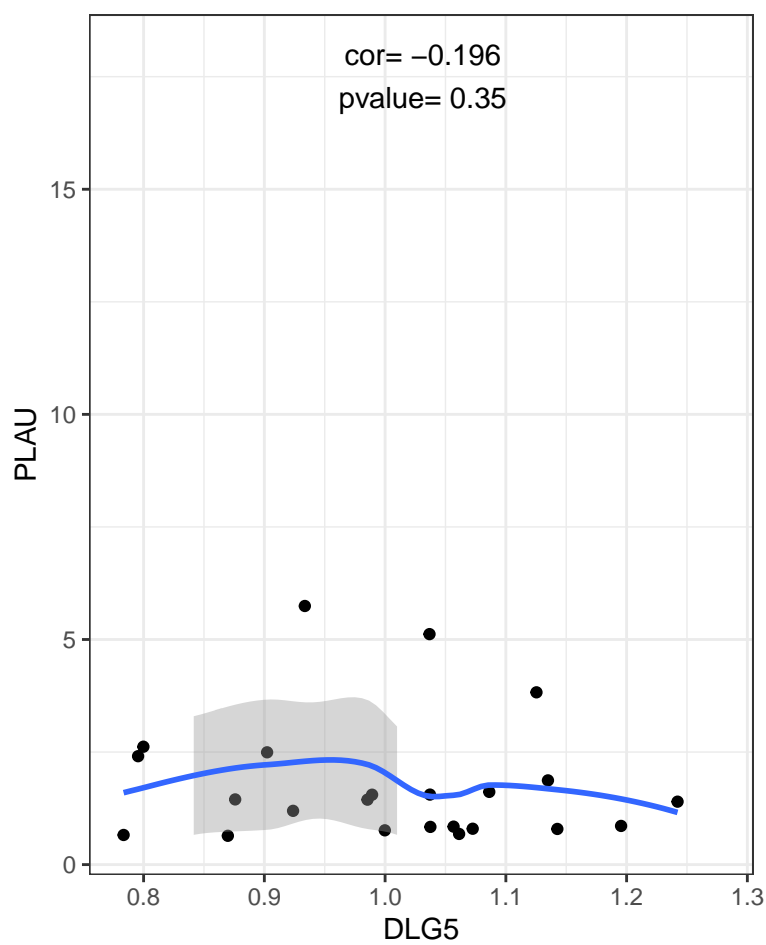

Supplement: Supplementary file 1 [file DataSheet3.zip › Input data and script2/DiseaseGene/PLAU ~ DLG5.pdf]

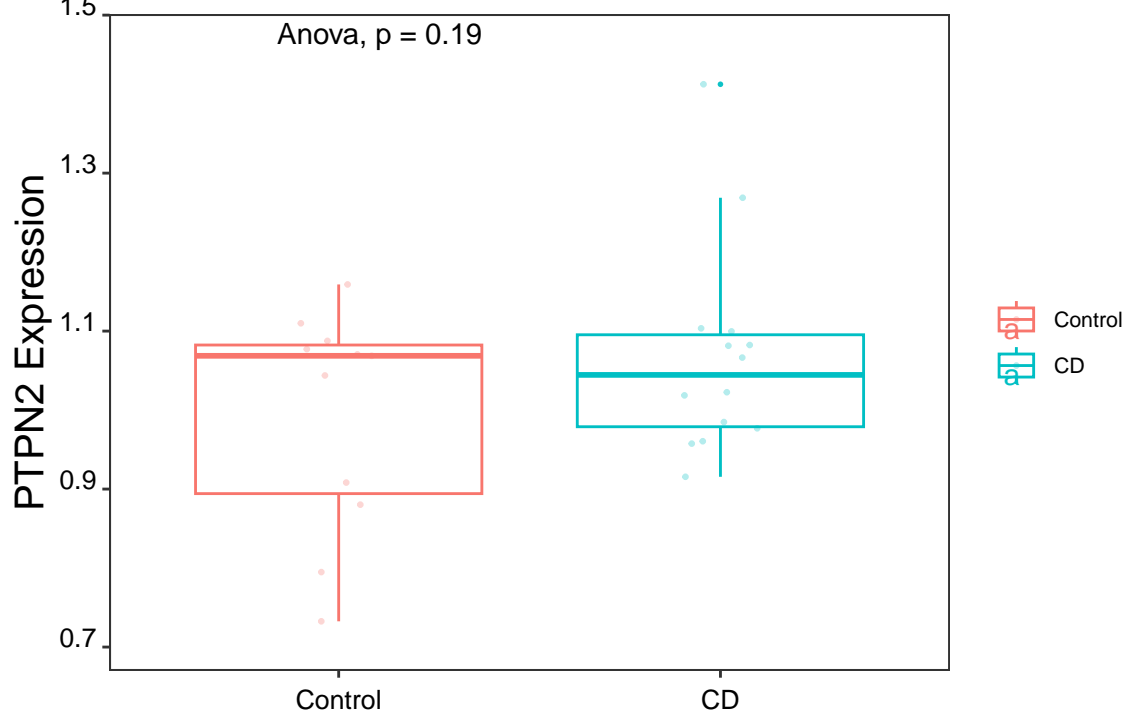

Supplement: Supplementary file 1 [file DataSheet3.zip › Input data and script2/DiseaseGene/PTPN2.HealthyDisease.pdf]

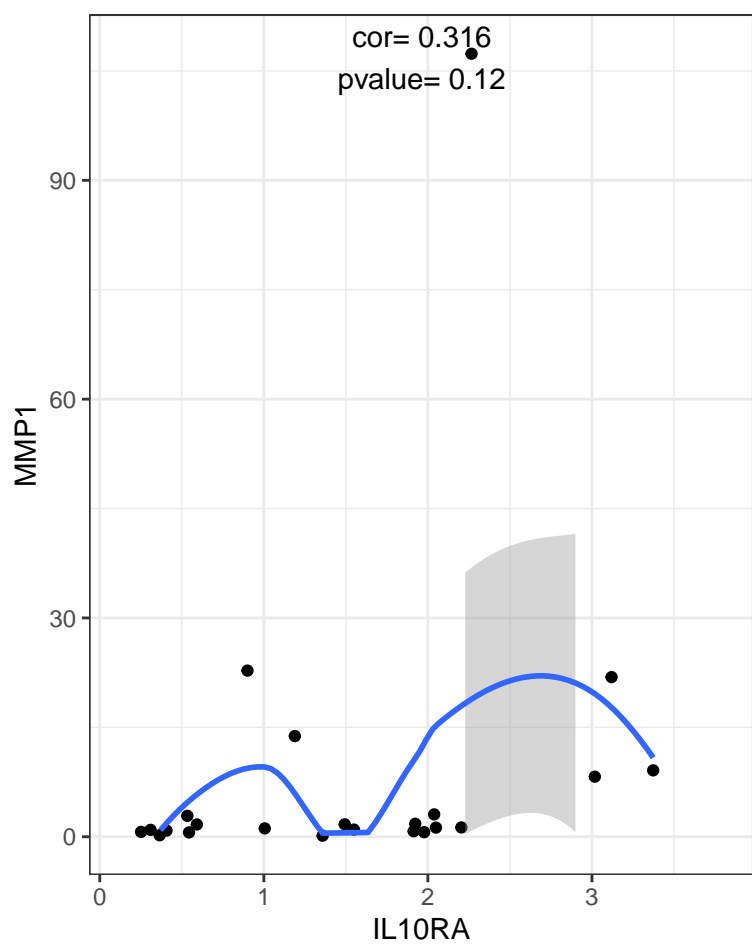

Supplement: Supplementary file 1 [file DataSheet3.zip › Input data and script2/DiseaseGene/MMP1 ~ IL10RA.pdf]

Anova,  $p = 0.22$

PRKCQ Expression

1.6  
1.2  
0.8  
0.4

Control

CD

Control  
CD

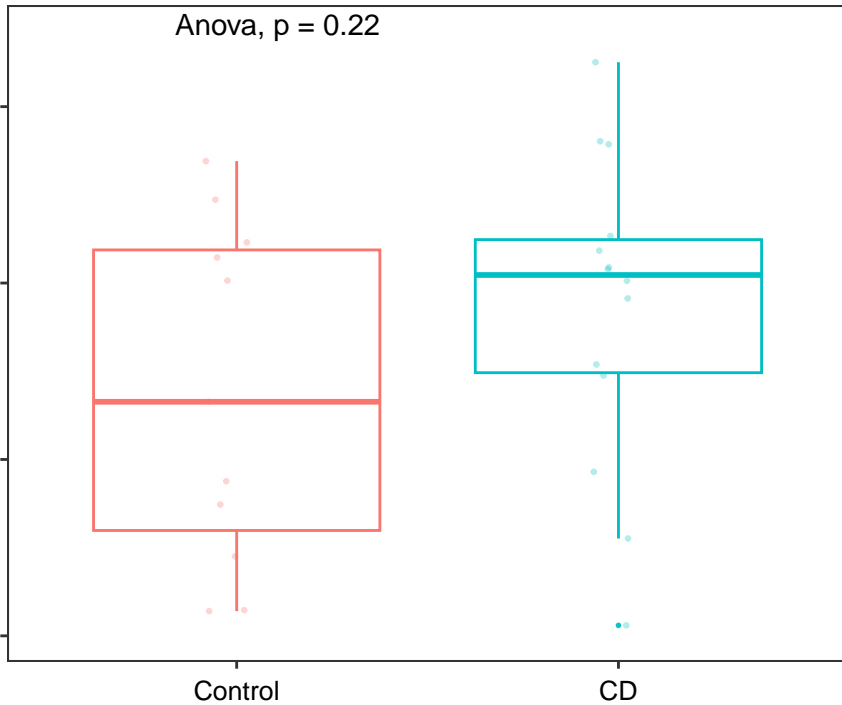

Supplement: Supplementary file 1 [file DataSheet3.zip › Input data and script2/DiseaseGene/PRKCQ.HealthyDisease.pdf]

Anova,  $p = 0.027$

TLR2 Expression

4  
3  
2  
1

Control

CD

Control  
CD

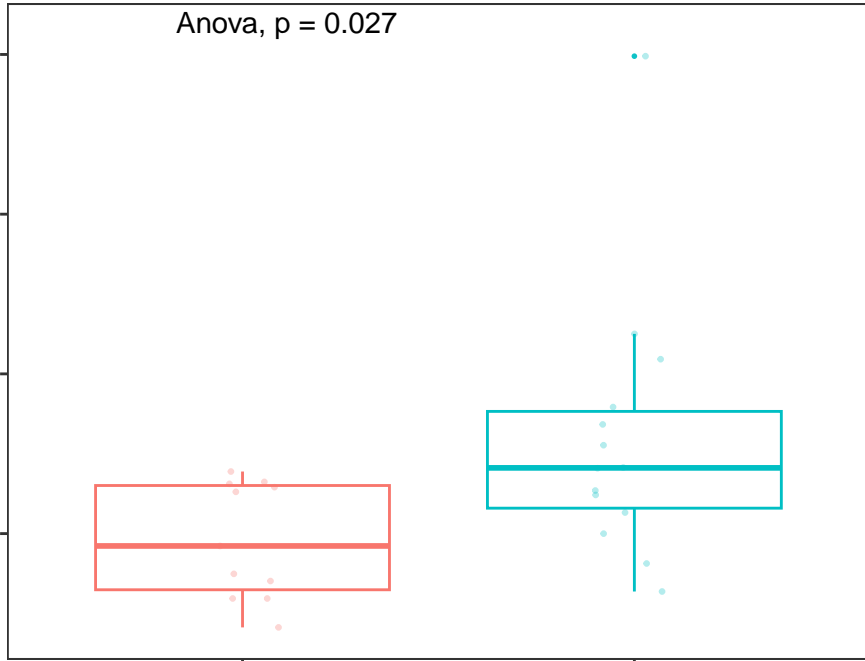

Supplement: Supplementary file 1 [file DataSheet3.zip › Input data and script2/DiseaseGene/TLR2.HealthyDisease.pdf]

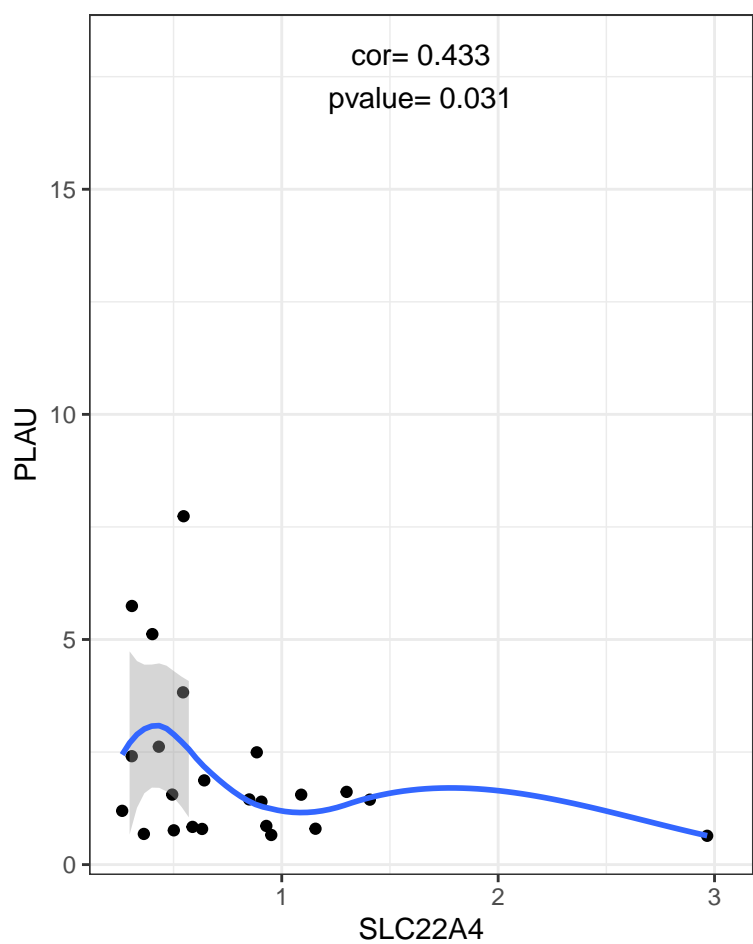

Supplement: Supplementary file 1 [file DataSheet3.zip › Input data and script2/DiseaseGene/PLAU ~ SLC22A4.pdf]

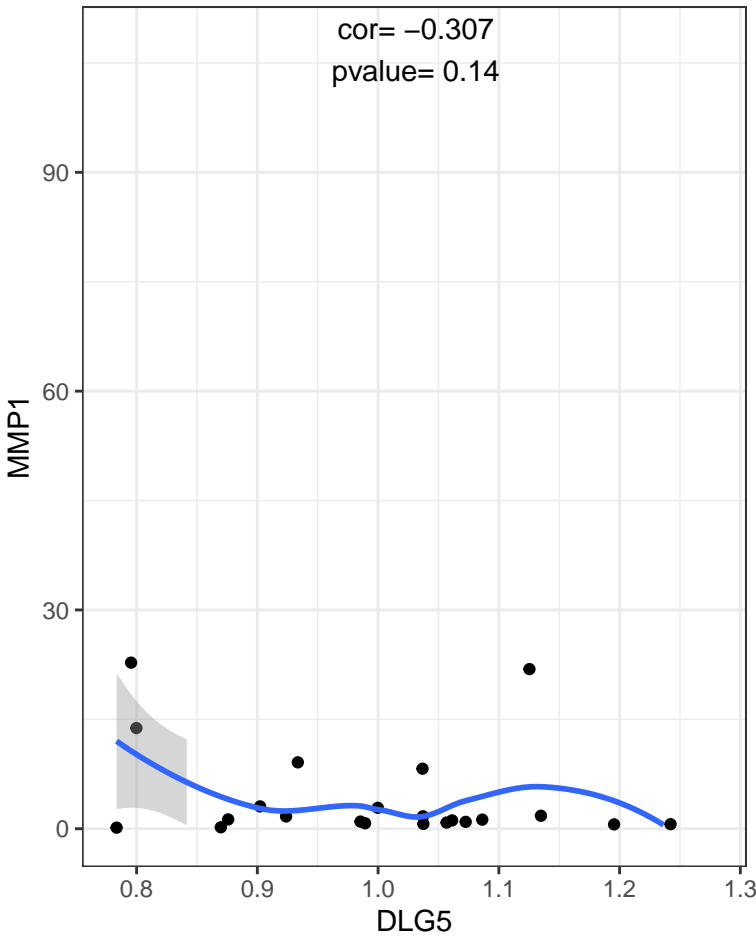

Supplement: Supplementary file 1 [file DataSheet3.zip › Input data and script2/DiseaseGene/MMP1 ~ DLG5.pdf]

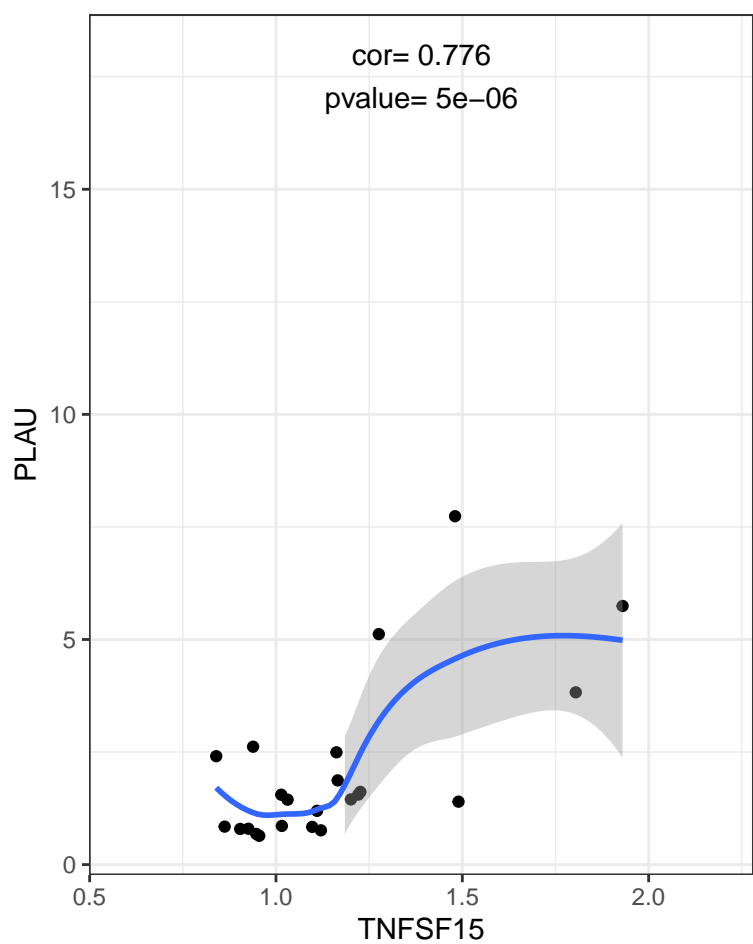

Supplement: Supplementary file 1 [file DataSheet3.zip › Input data and script2/DiseaseGene/PLAU ~ TNFSF15.pdf]

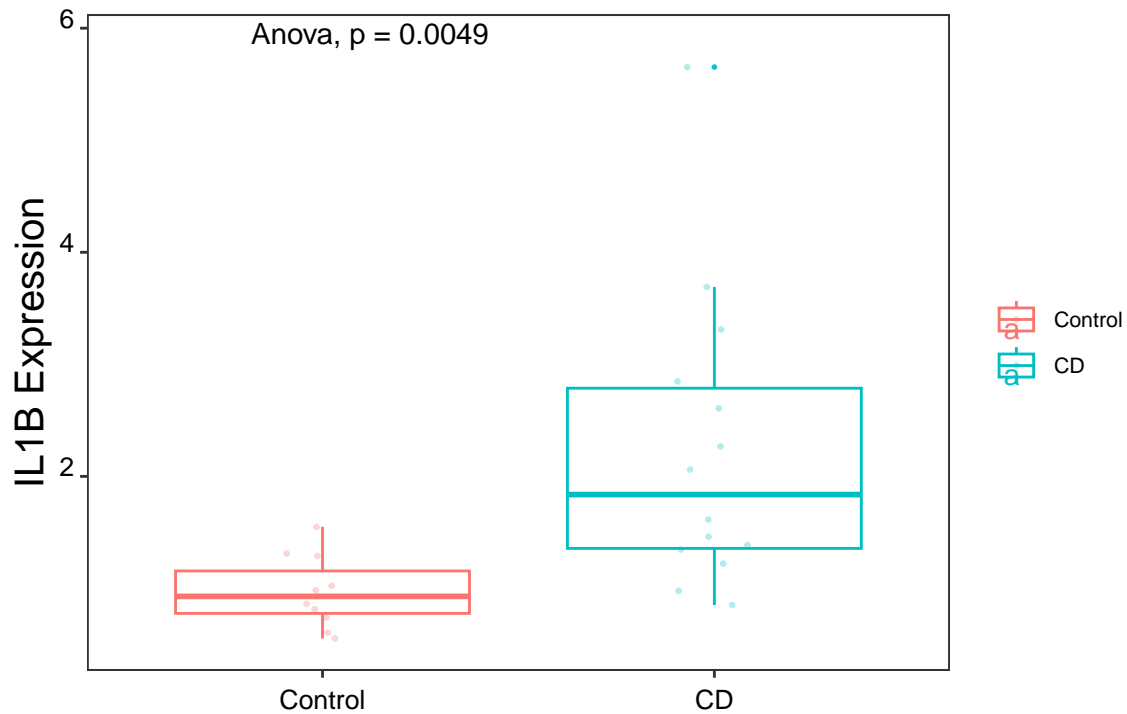

Supplement: Supplementary file 1 [file DataSheet3.zip › Input data and script2/DiseaseGene/IL1B.HealthyDisease.pdf]

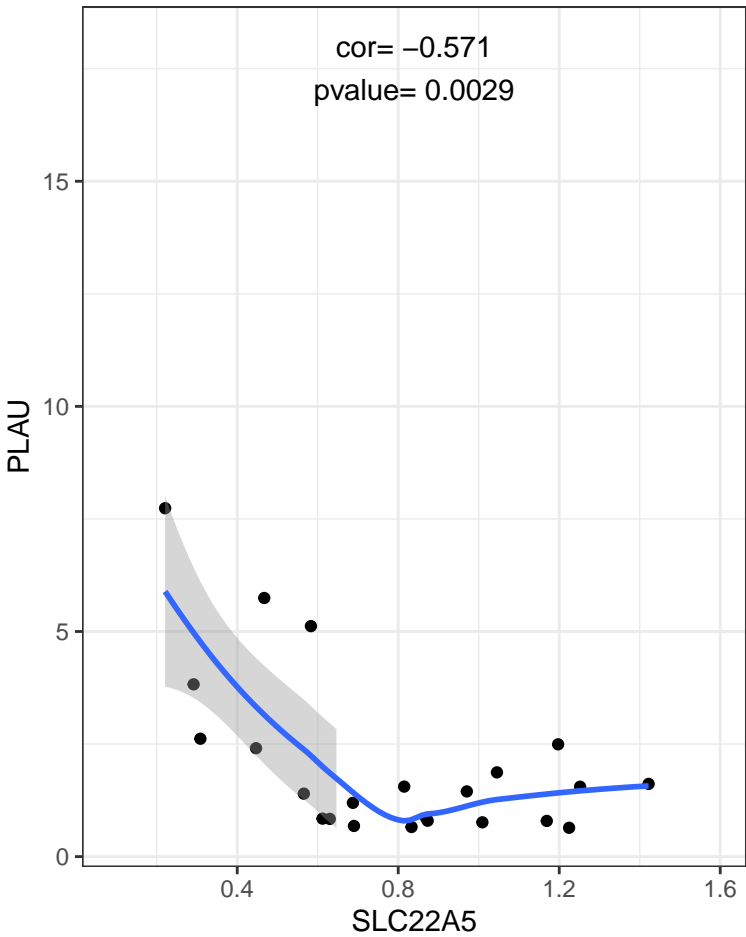

Supplement: Supplementary file 1 [file DataSheet3.zip › Input data and script2/DiseaseGene/PLAU ~ SLC22A5.pdf]

Anova,  $p = 0.024$

IL10RA Expression

2.0  
1.5  
1.0  
0.5

Control

CD

Control  
CD

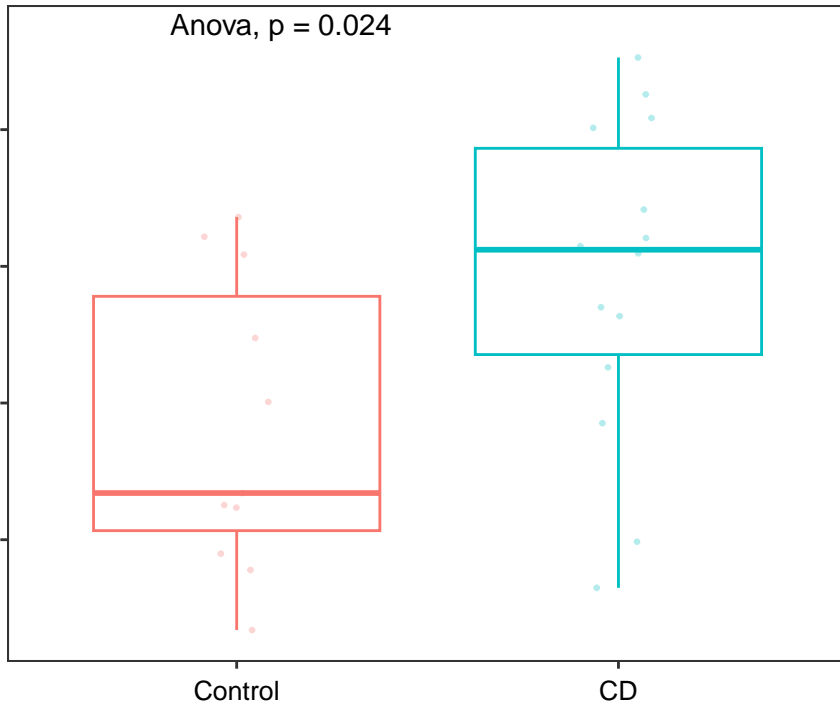

Supplement: Supplementary file 1 [file DataSheet3.zip › Input data and script2/DiseaseGene/IL10RA.HealthyDisease.pdf]

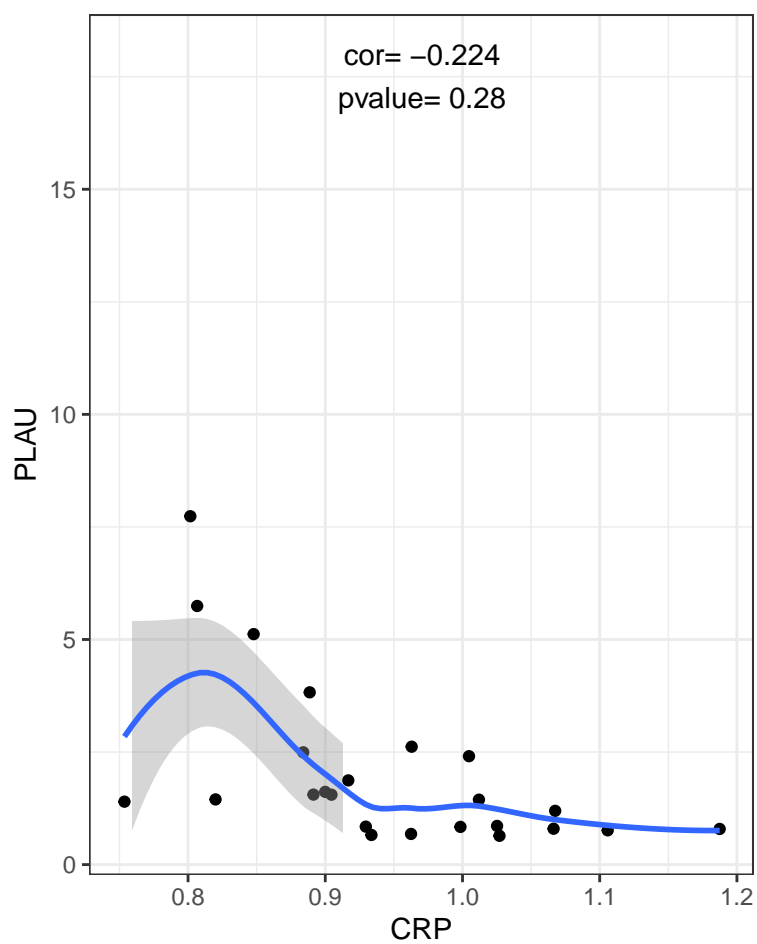

Supplement: Supplementary file 1 [file DataSheet3.zip › Input data and script2/DiseaseGene/PLAU ~ CRP.pdf]

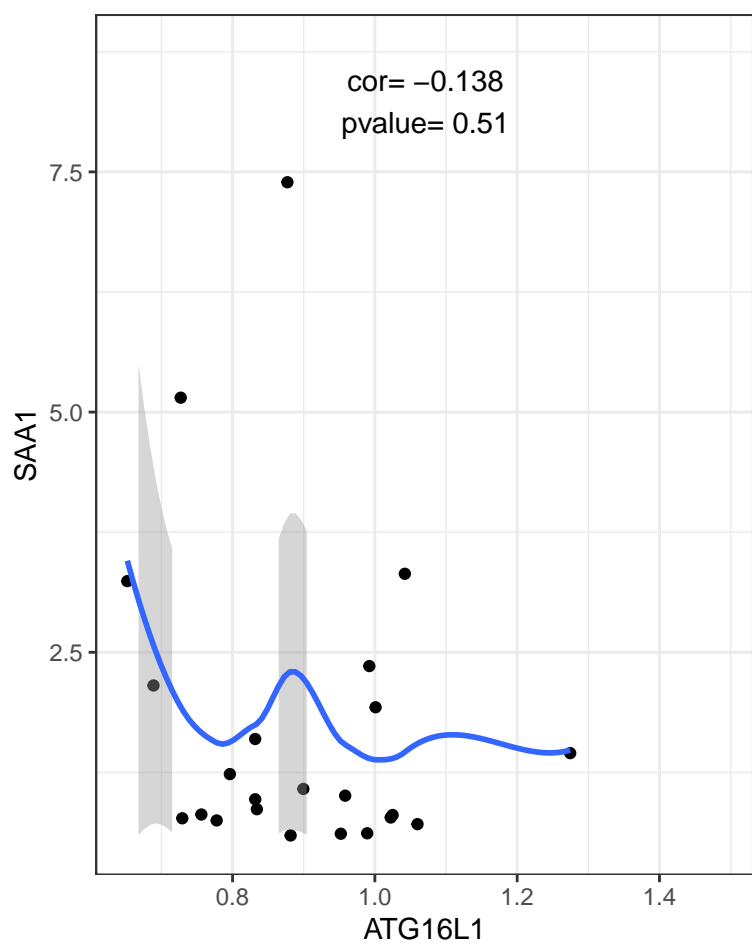

Supplement: Supplementary file 1 [file DataSheet3.zip › Input data and script2/DiseaseGene/SAA1 ~ ATG16L1.pdf]

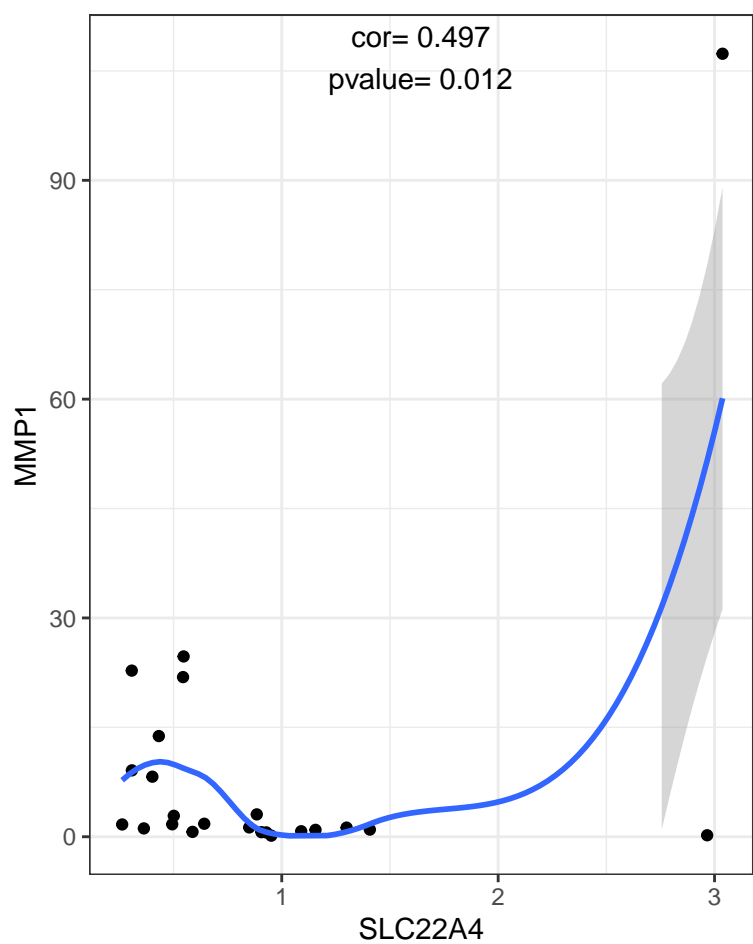

Supplement: Supplementary file 1 [file DataSheet3.zip › Input data and script2/DiseaseGene/MMP1 ~ SLC22A4.pdf]

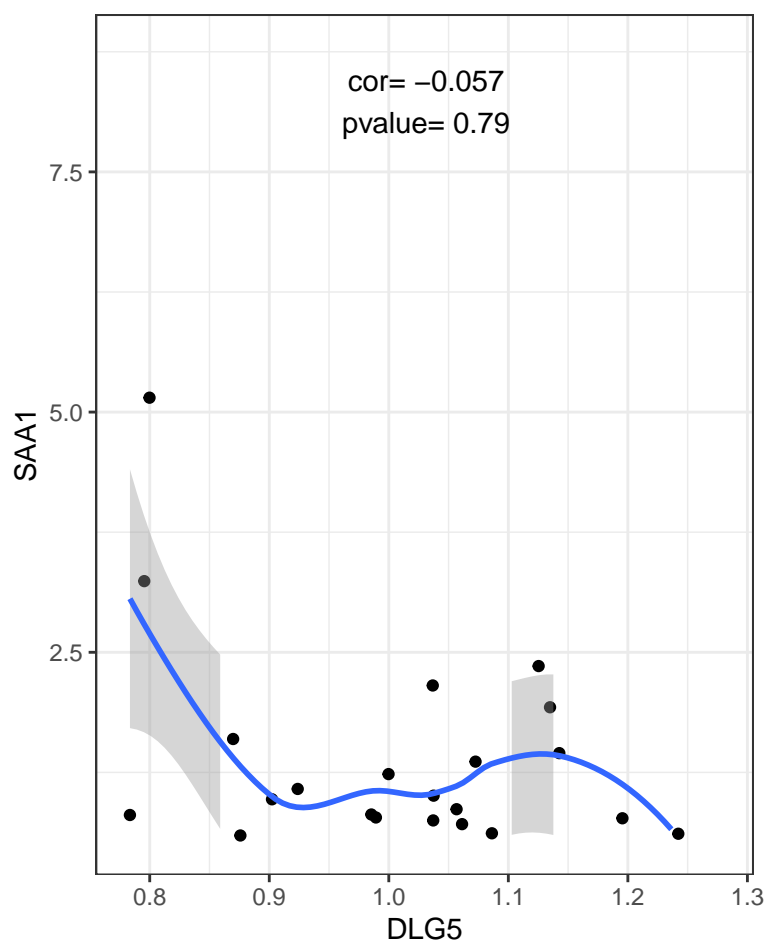

Supplement: Supplementary file 1 [file DataSheet3.zip › Input data and script2/DiseaseGene/SAA1 ~ DLG5.pdf]

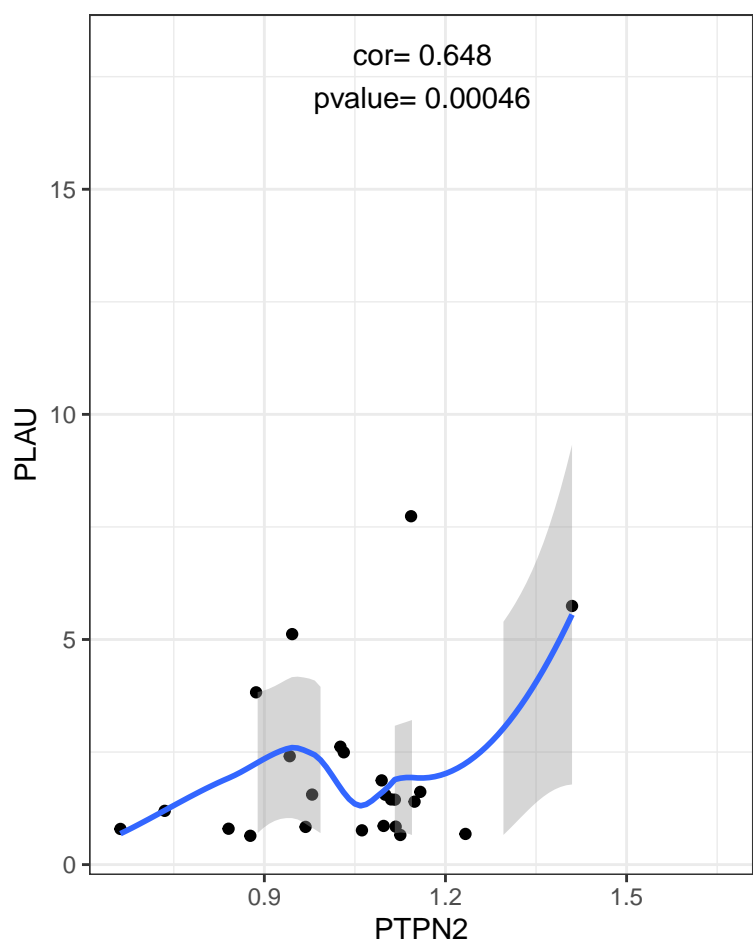

Supplement: Supplementary file 1 [file DataSheet3.zip › Input data and script2/DiseaseGene/PLAU ~ PTPN2.pdf]

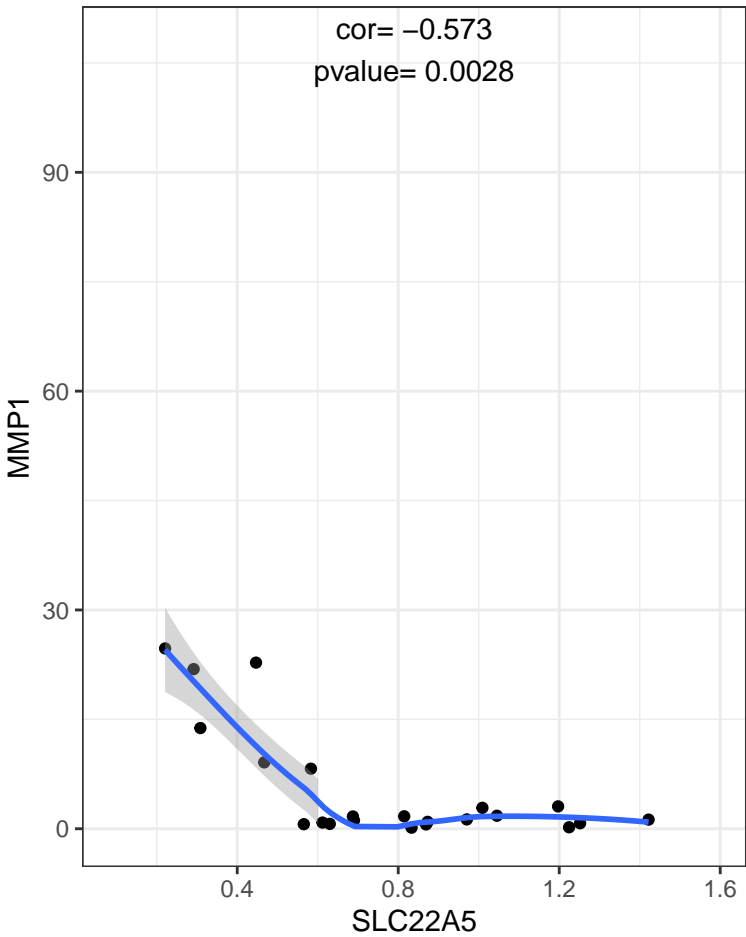

Supplement: Supplementary file 1 [file DataSheet3.zip › Input data and script2/DiseaseGene/MMP1 ~ SLC22A5.pdf]

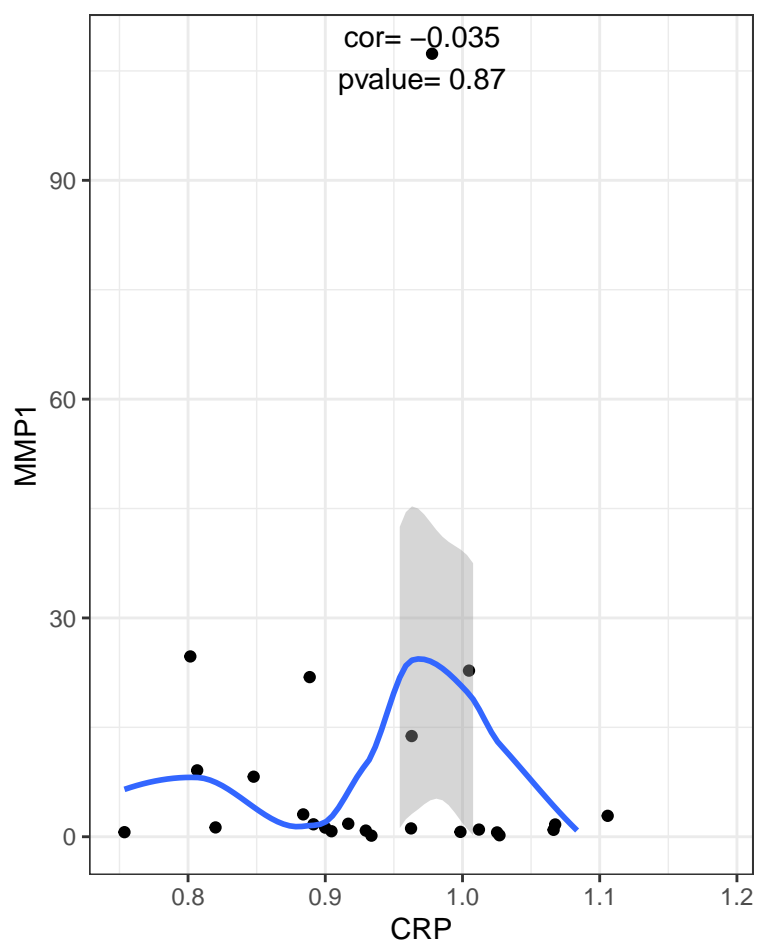

Supplement: Supplementary file 1 [file DataSheet3.zip › Input data and script2/DiseaseGene/MMP1 ~ CRP.pdf]

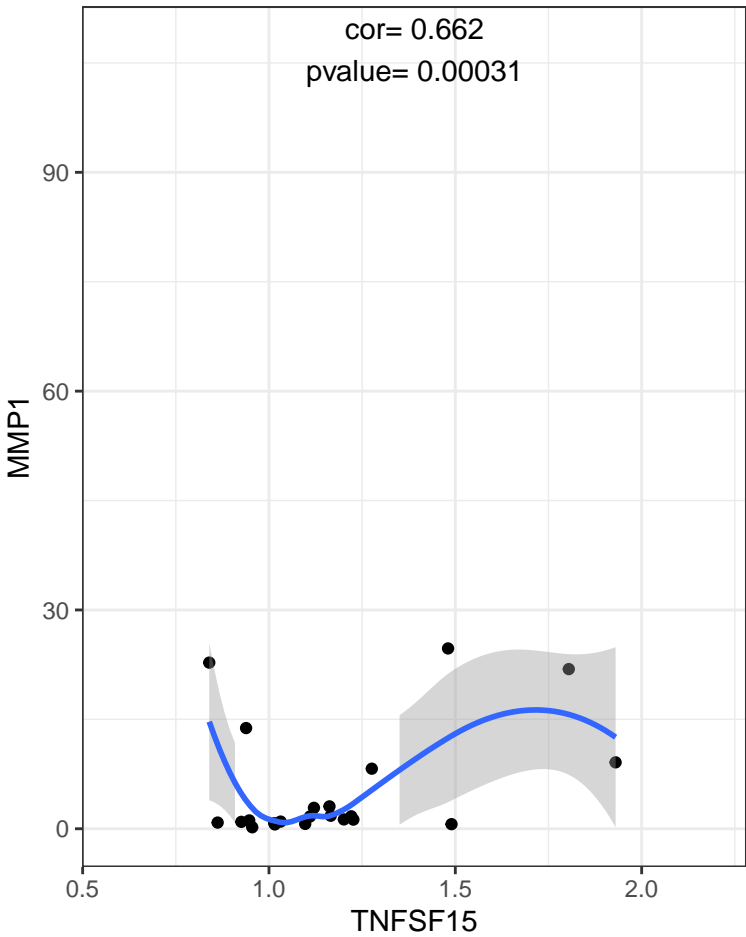

Supplement: Supplementary file 1 [file DataSheet3.zip › Input data and script2/DiseaseGene/MMP1 ~ TNFSF15.pdf]

Anova,  $p = 0.88$

DLG5 Expression

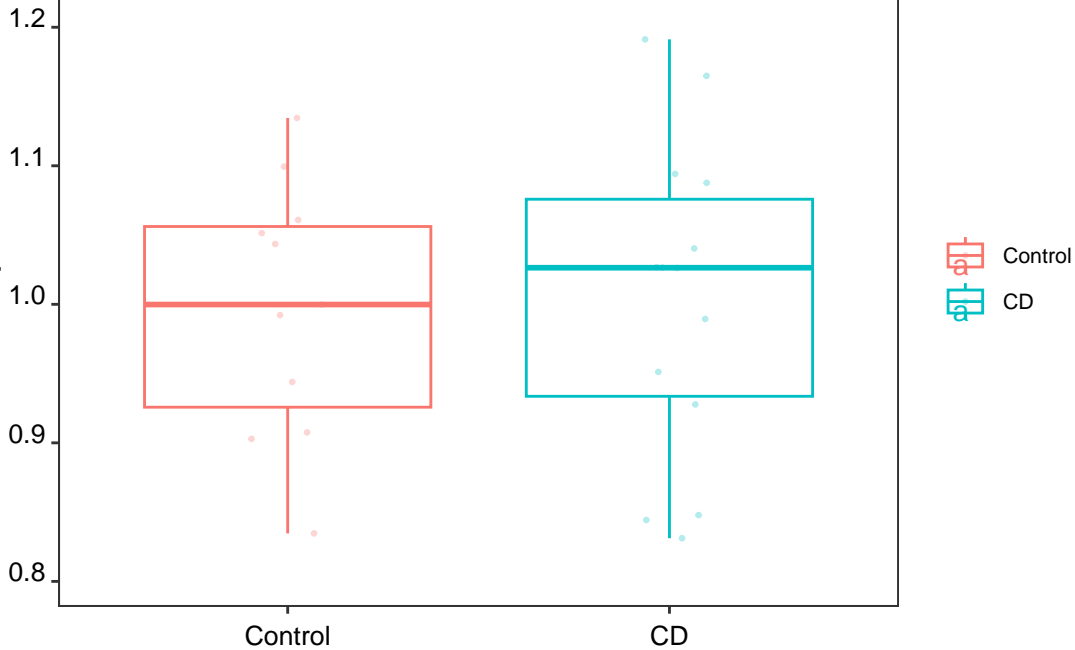

Supplement: Supplementary file 1 [file DataSheet3.zip › Input data and script2/DiseaseGene/DLG5.HealthyDisease.pdf]

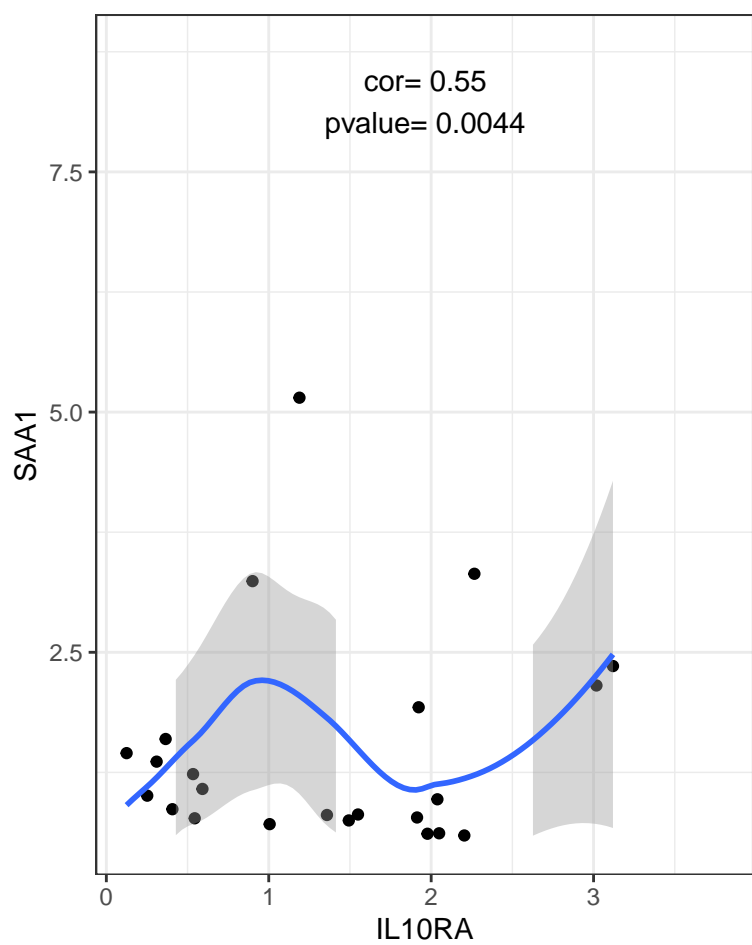

Supplement: Supplementary file 1 [file DataSheet3.zip › Input data and script2/DiseaseGene/SAA1 ~ IL10RA.pdf]

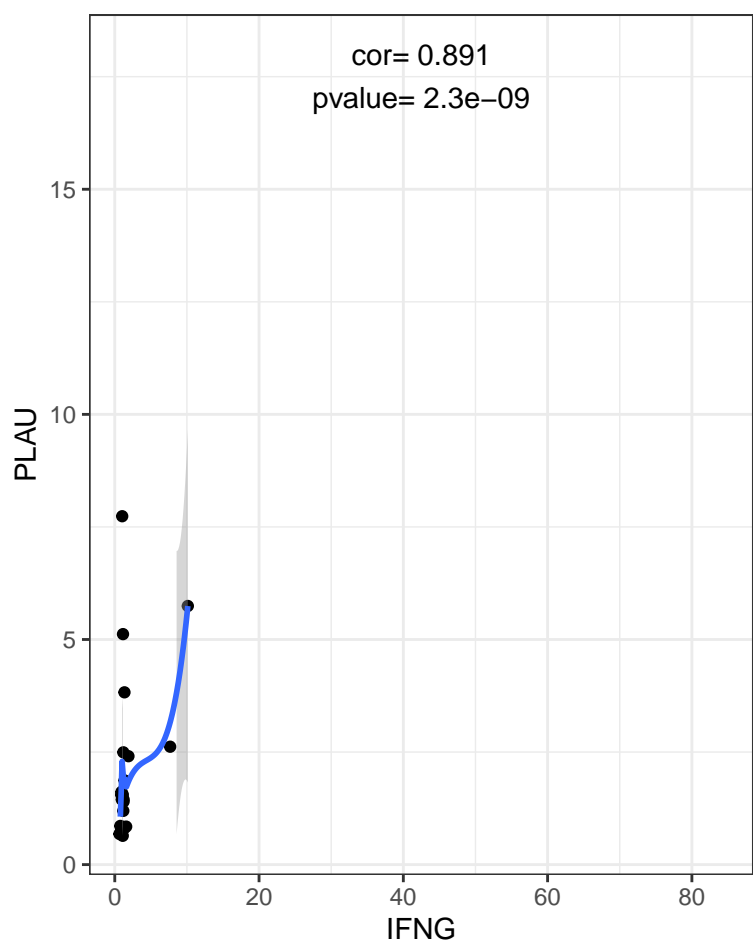

Supplement: Supplementary file 1 [file DataSheet3.zip › Input data and script2/DiseaseGene/PLAU ~ IFNG.pdf]

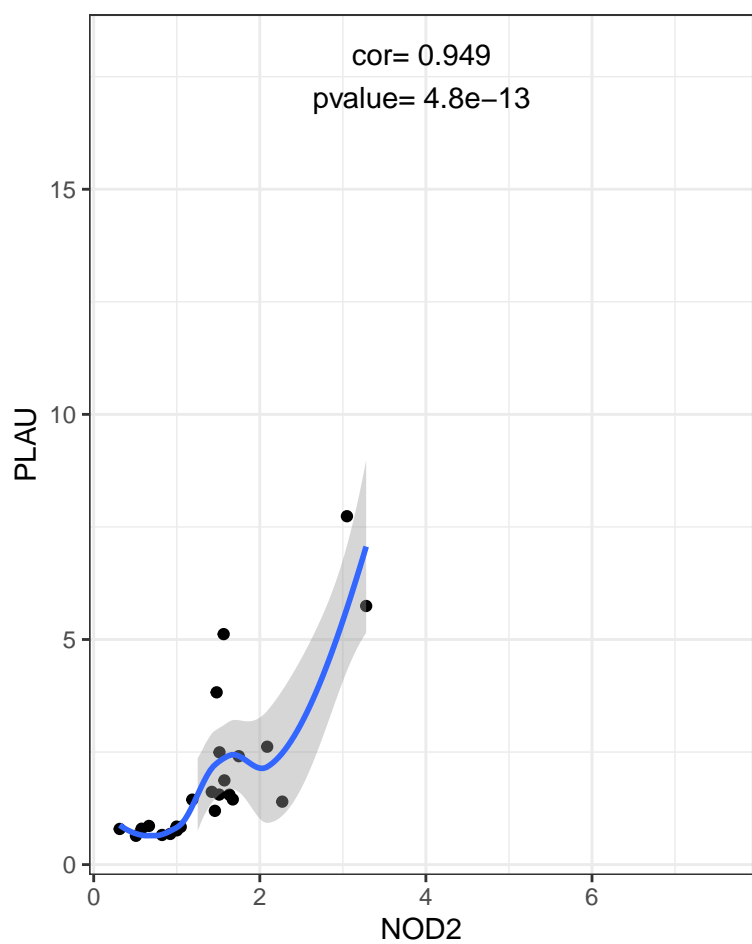

Supplement: Supplementary file 1 [file DataSheet3.zip › Input data and script2/DiseaseGene/PLAU ~ NOD2.pdf]

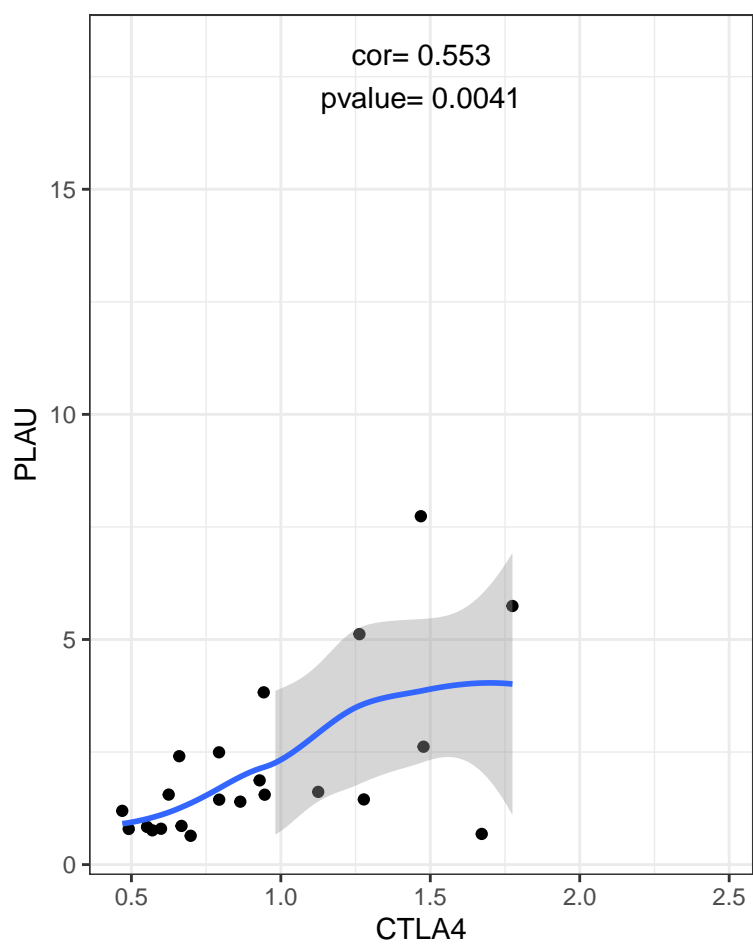

Supplement: Supplementary file 1 [file DataSheet3.zip › Input data and script2/DiseaseGene/PLAU ~ CTLA4.pdf]

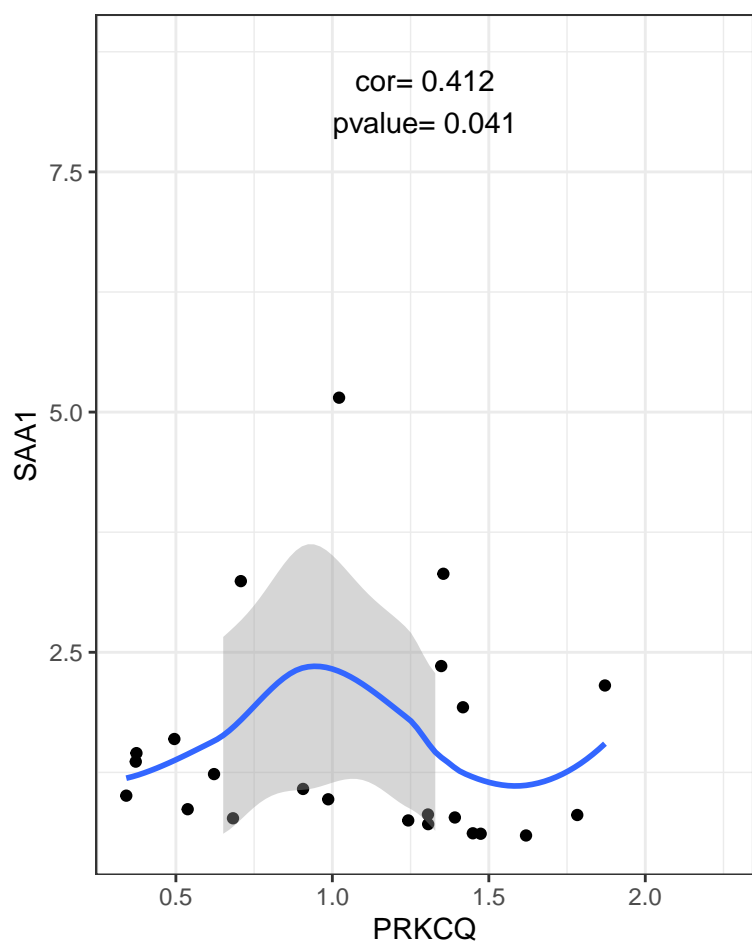

Supplement: Supplementary file 1 [file DataSheet3.zip › Input data and script2/DiseaseGene/SAA1 ~ PRKCQ.pdf]

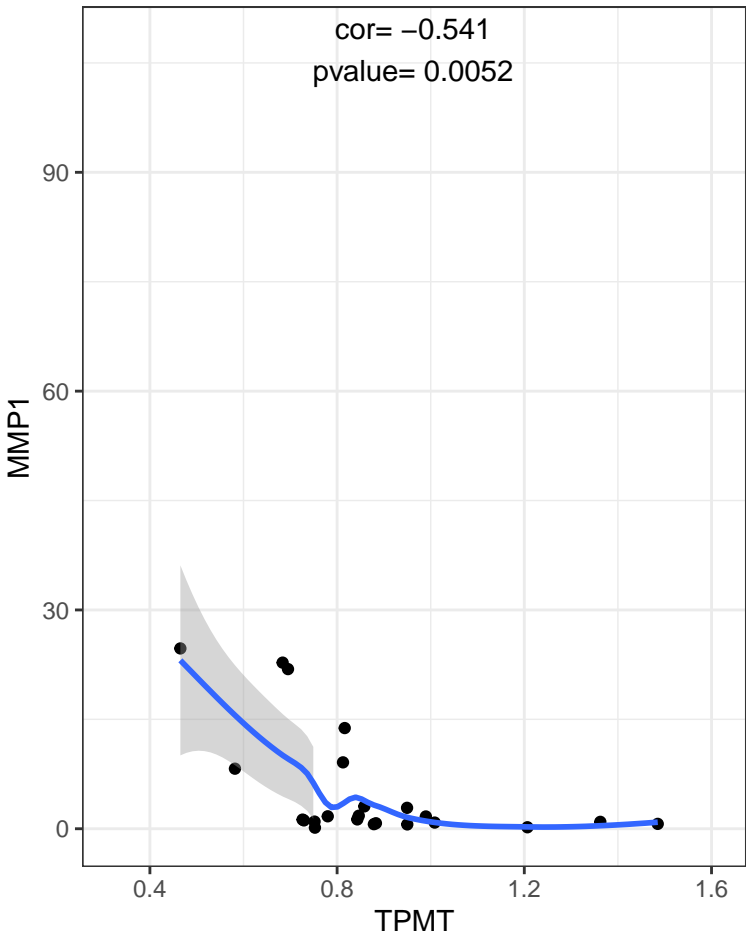

Supplement: Supplementary file 1 [file DataSheet3.zip › Input data and script2/DiseaseGene/MMP1 ~ TPMT.pdf]

Anova,  $p = 0.021$

CRP Expression

1.1  
1.0  
0.9  
0.8

Control

CD

Control  
CD

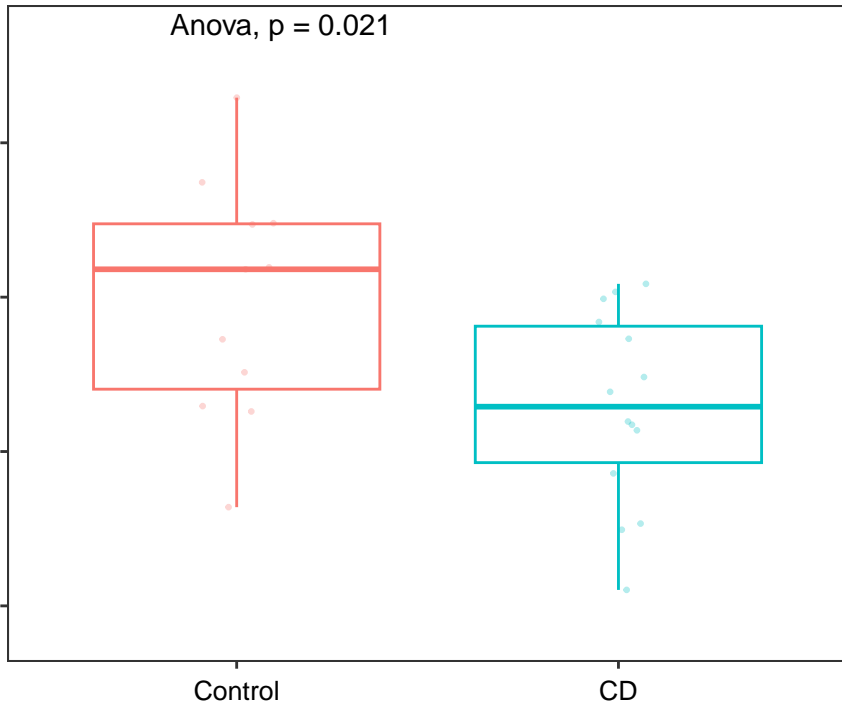

Supplement: Supplementary file 1 [file DataSheet3.zip › Input data and script2/DiseaseGene/CRP.HealthyDisease.pdf]

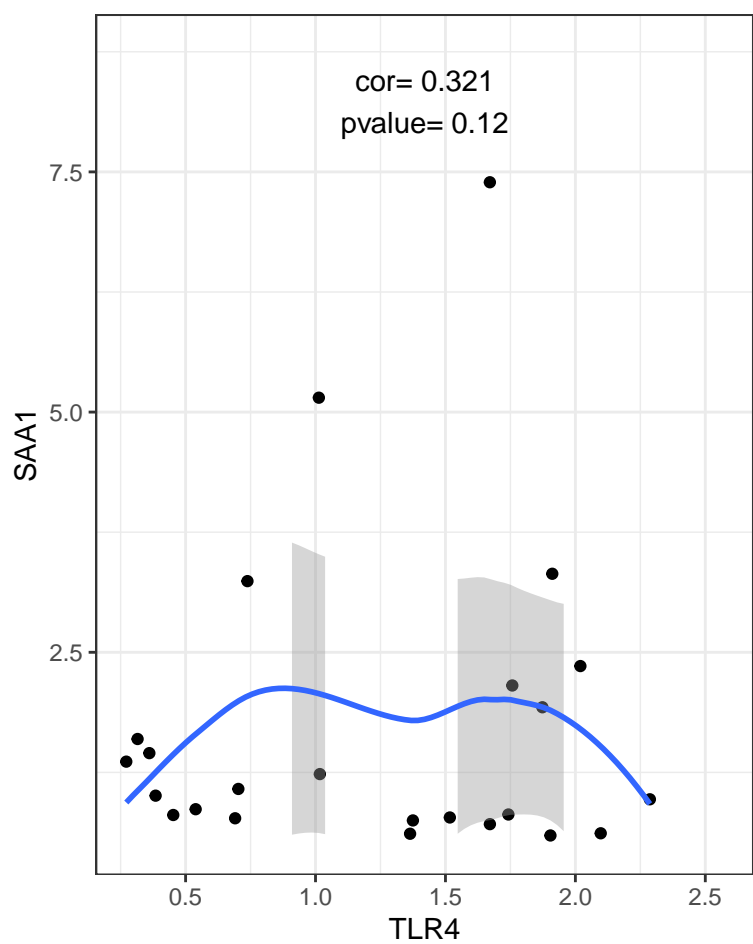

Supplement: Supplementary file 1 [file DataSheet3.zip › Input data and script2/DiseaseGene/SAA1 ~ TLR4.pdf]

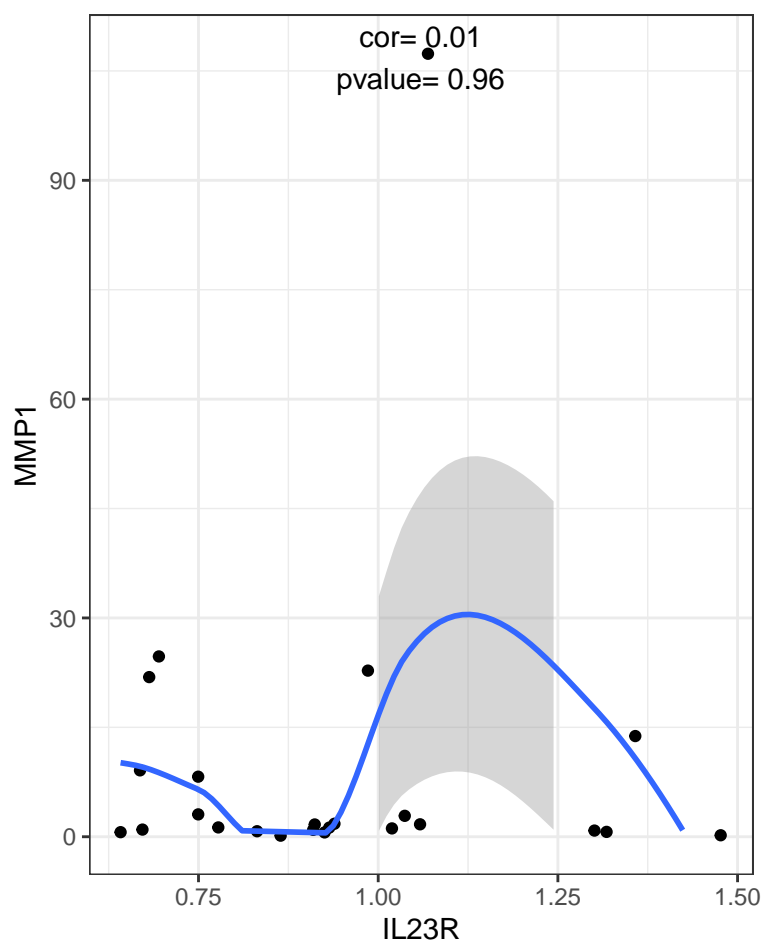

Supplement: Supplementary file 1 [file DataSheet3.zip › Input data and script2/DiseaseGene/MMP1 ~ IL23R.pdf]

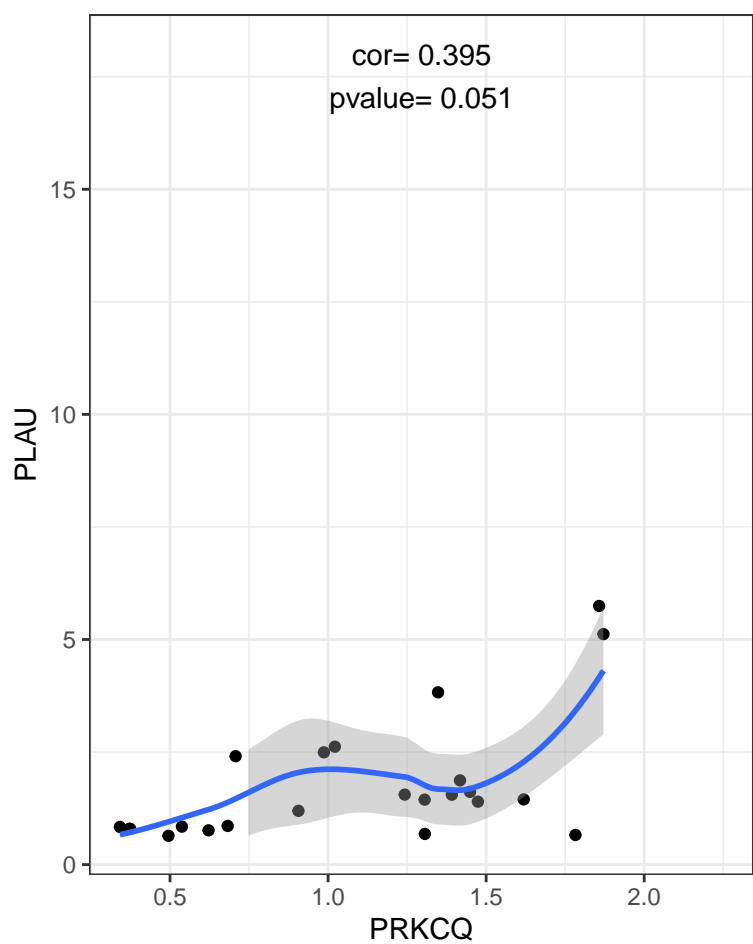

Supplement: Supplementary file 1 [file DataSheet3.zip › Input data and script2/DiseaseGene/PLAU ~ PRKCQ.pdf]

Anova,  $p = 0.085$

IL6 Expression

6

4

2

Control

CD

Control  
CD

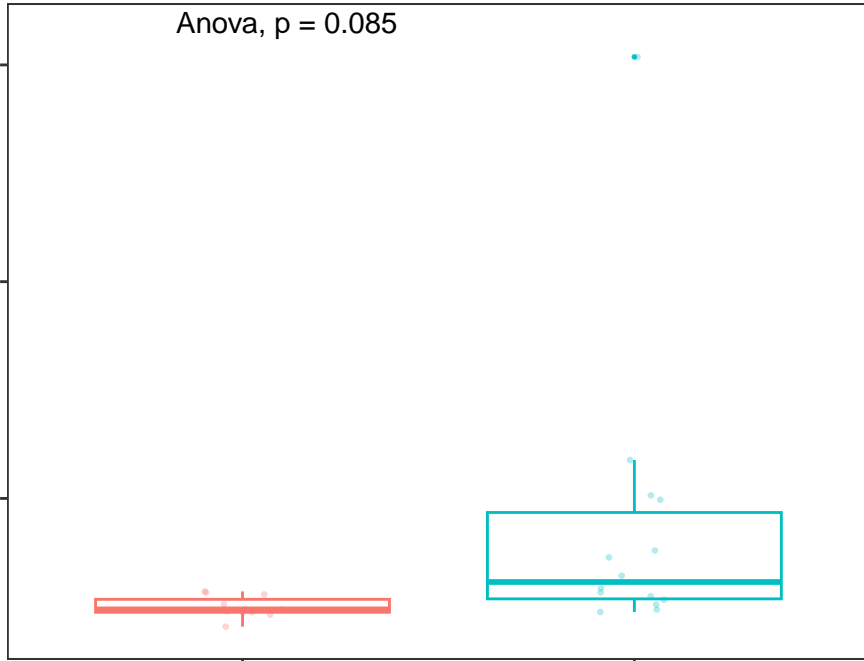

Supplement: Supplementary file 1 [file DataSheet3.zip › Input data and script2/DiseaseGene/IL6.HealthyDisease.pdf]

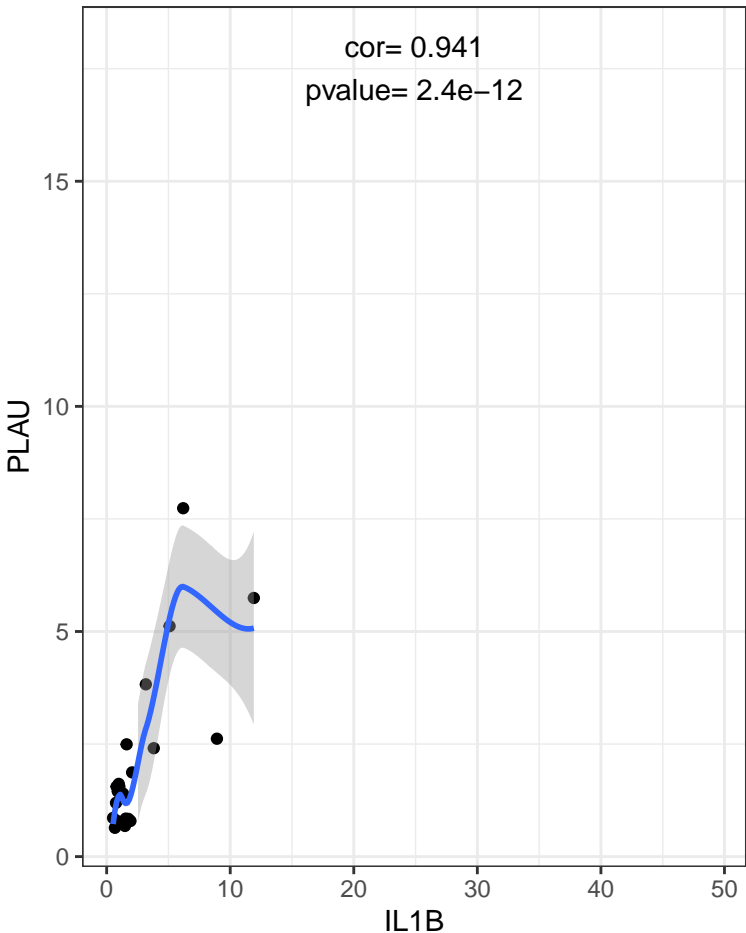

Supplement: Supplementary file 1 [file DataSheet3.zip › Input data and script2/DiseaseGene/PLAU ~ IL1B.pdf]

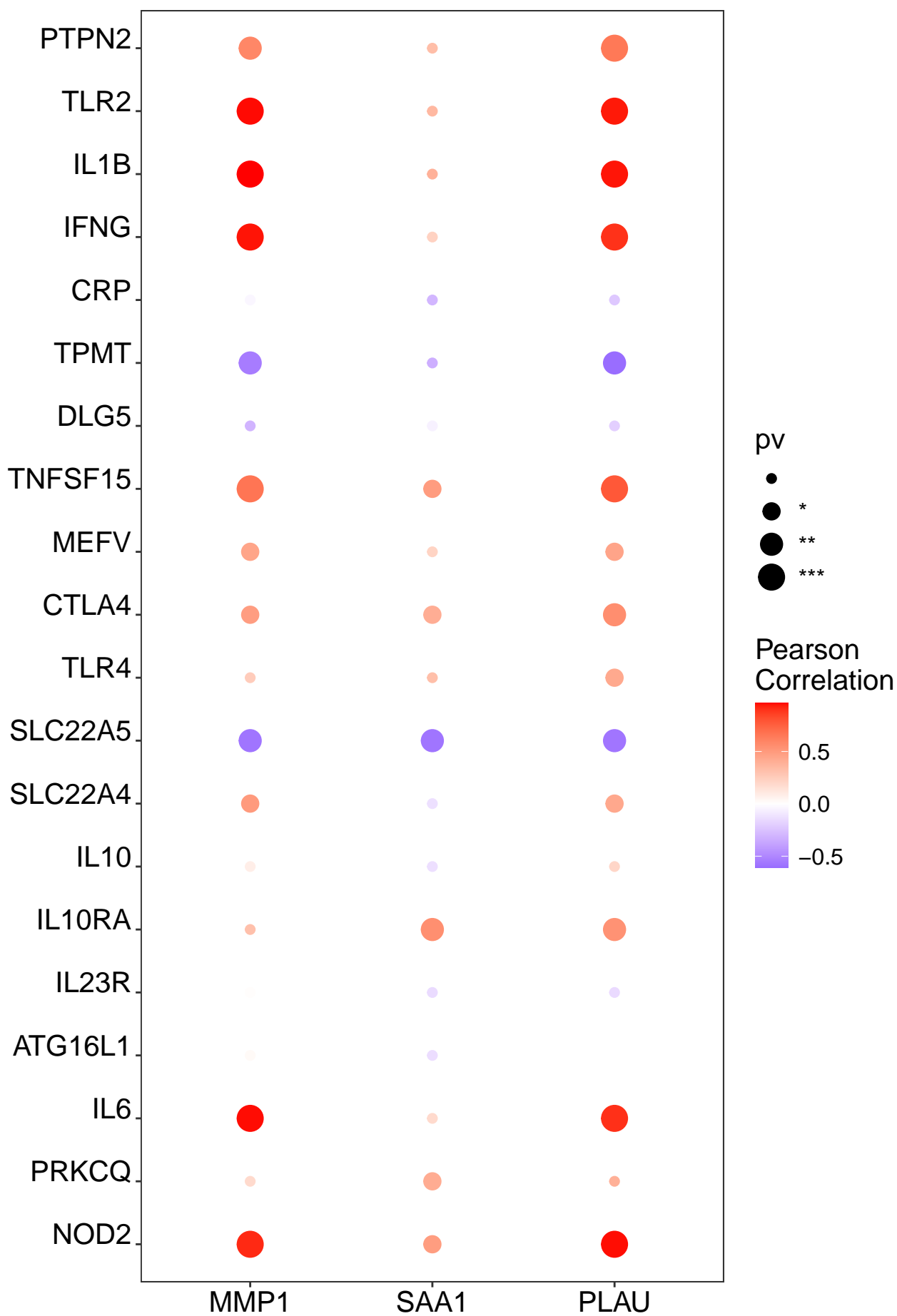

Supplement: Supplementary file 1 [file DataSheet3.zip › Input data and script2/DiseaseGene/DiseaseGene ~ cormap.pdf]

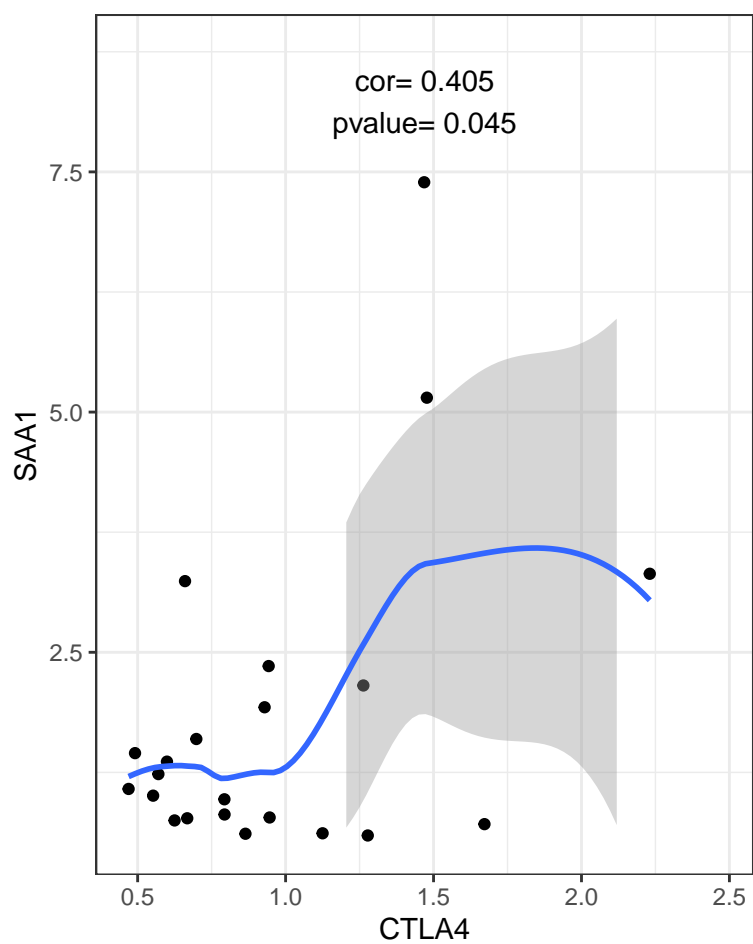

Supplement: Supplementary file 1 [file DataSheet3.zip › Input data and script2/DiseaseGene/SAA1 ~ CTLA4.pdf]

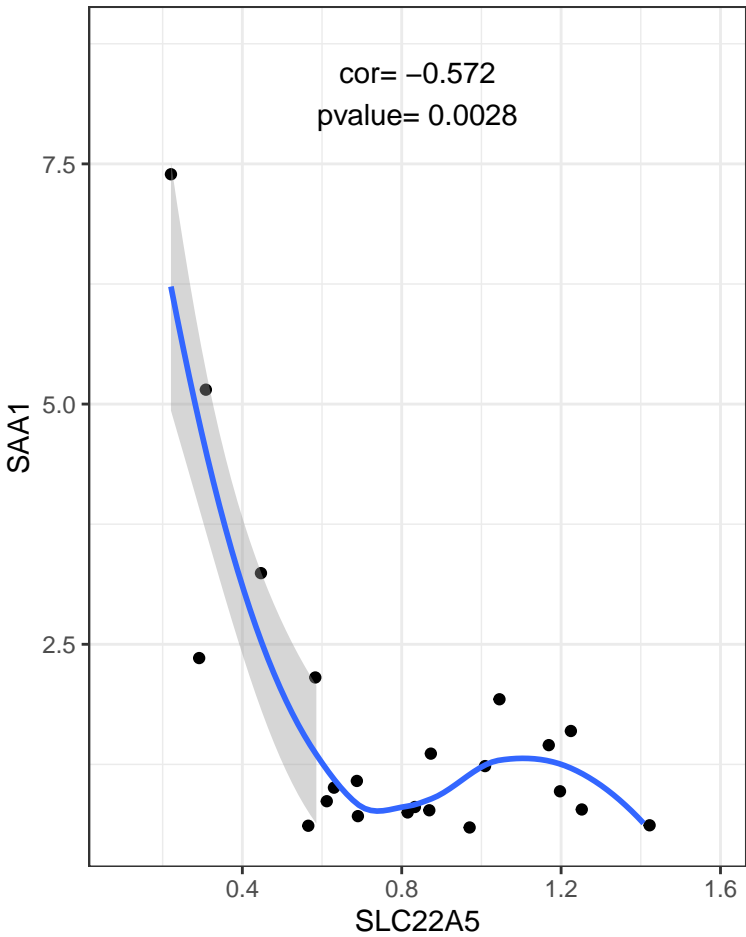

Supplement: Supplementary file 1 [file DataSheet3.zip › Input data and script2/DiseaseGene/SAA1 ~ SLC22A5.pdf]

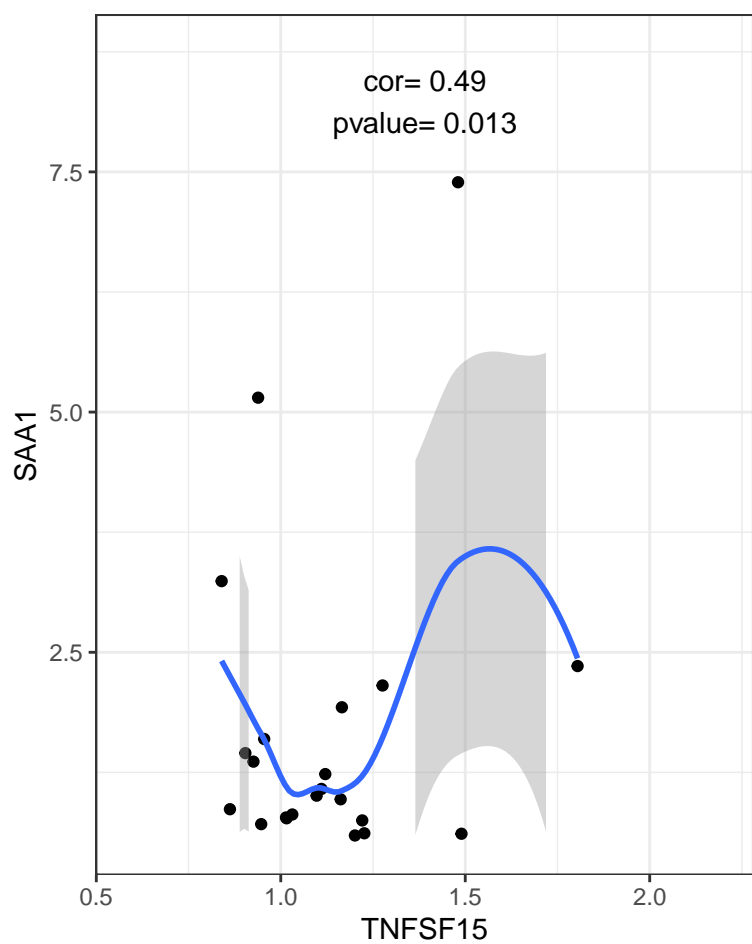

Supplement: Supplementary file 1 [file DataSheet3.zip › Input data and script2/DiseaseGene/SAA1 ~ TNFSF15.pdf]

SLC22A4 Expression

Anova,  $p = 0.31$

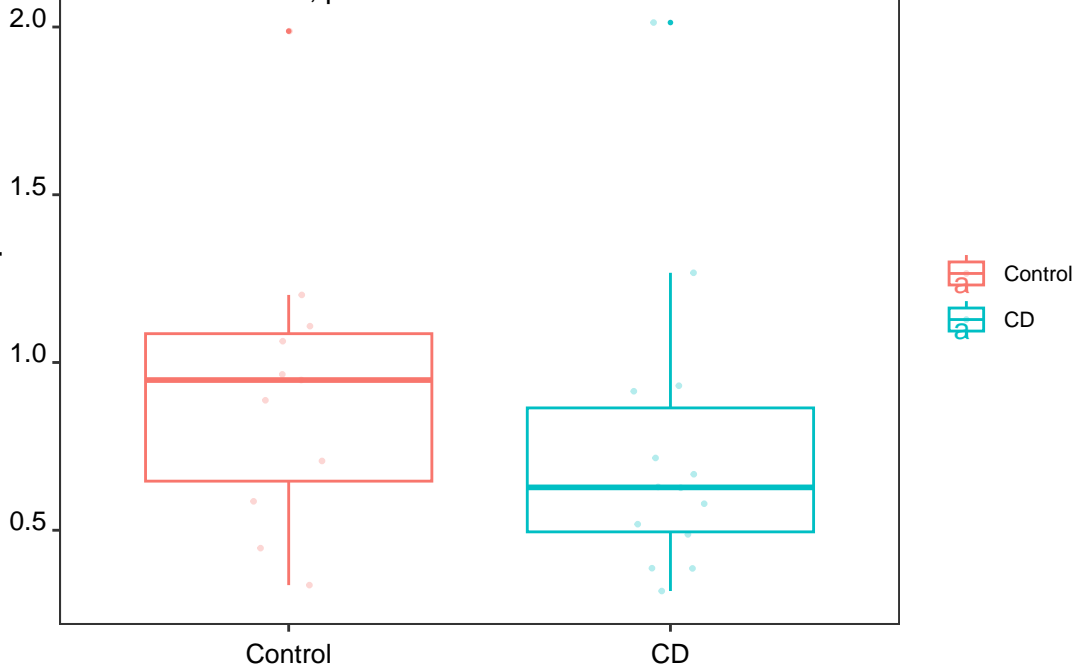

Supplement: Supplementary file 1 [file DataSheet3.zip › Input data and script2/DiseaseGene/SLC22A4.HealthyDisease.pdf]

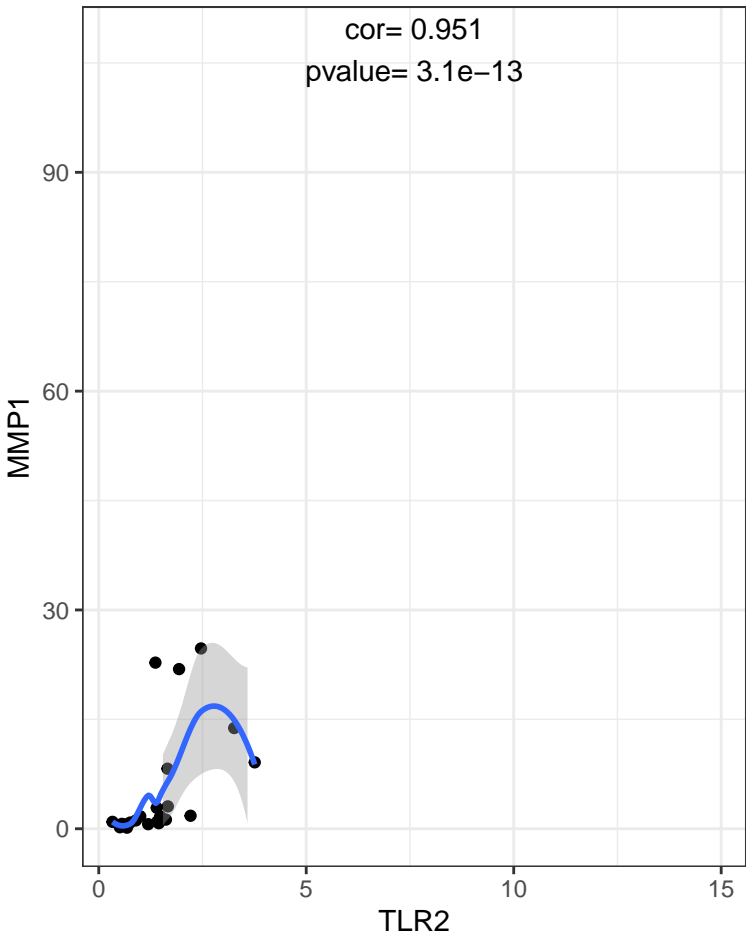

Supplement: Supplementary file 1 [file DataSheet3.zip › Input data and script2/DiseaseGene/MMP1 ~ TLR2.pdf]

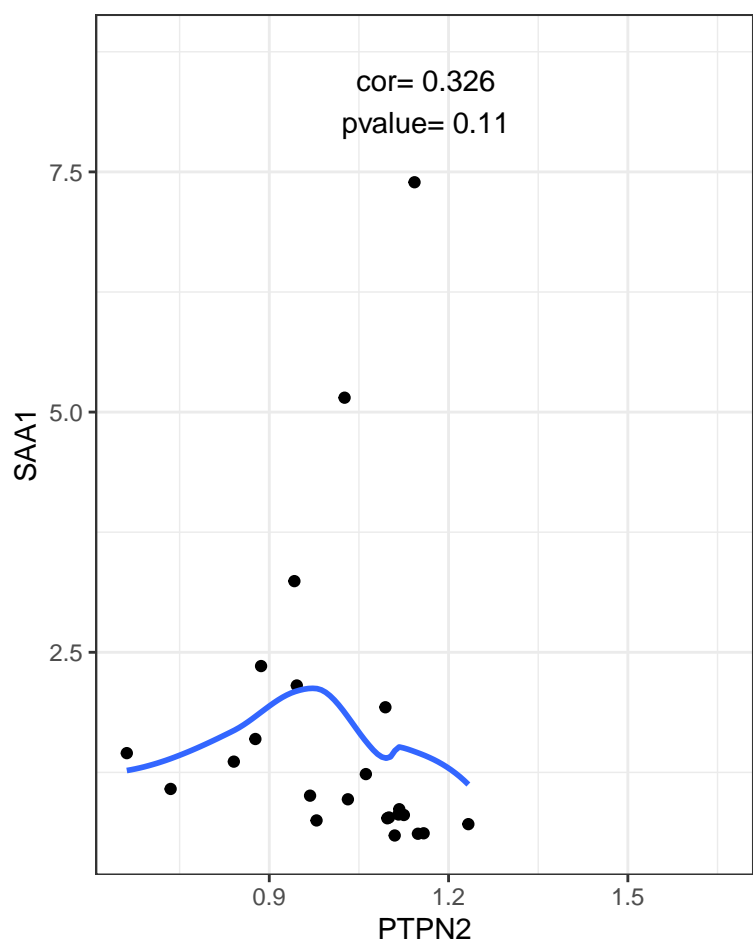

Supplement: Supplementary file 1 [file DataSheet3.zip › Input data and script2/DiseaseGene/SAA1 ~ PTPN2.pdf]

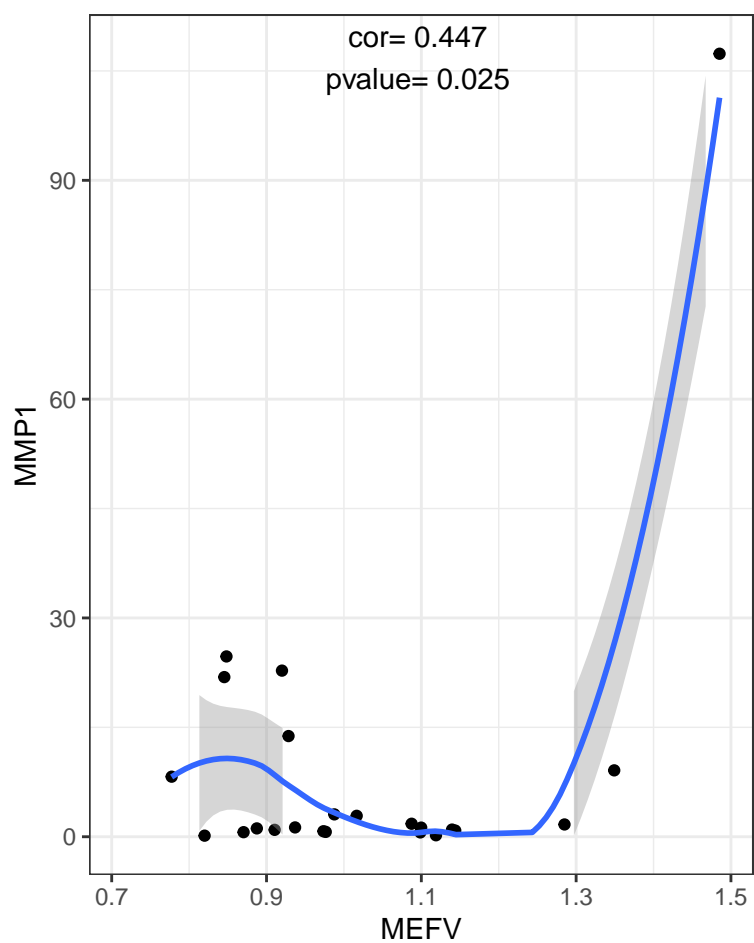

Supplement: Supplementary file 1 [file DataSheet3.zip › Input data and script2/DiseaseGene/MMP1 ~ MEFV.pdf]

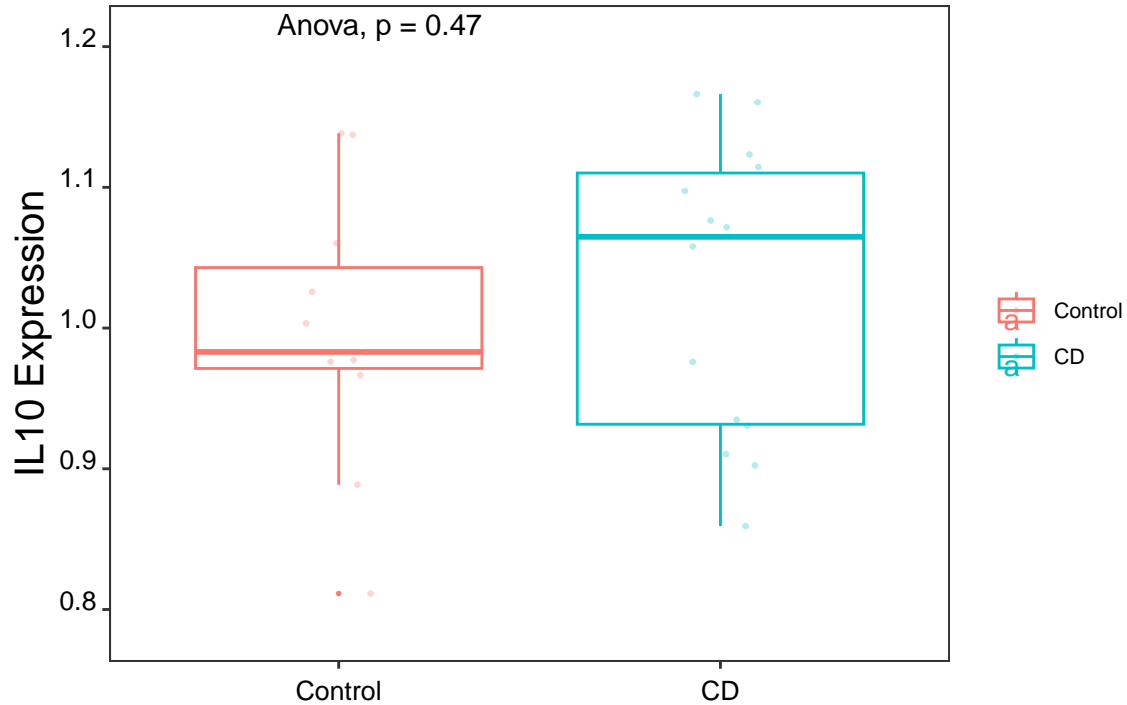

Supplement: Supplementary file 1 [file DataSheet3.zip › Input data and script2/DiseaseGene/IL10.HealthyDisease.pdf]

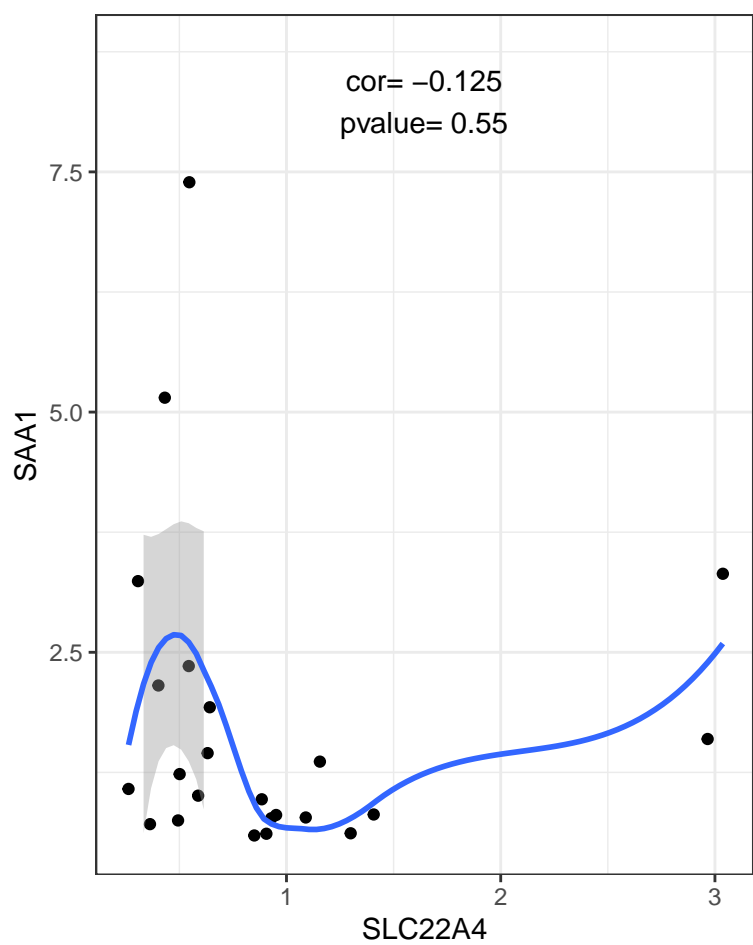

Supplement: Supplementary file 1 [file DataSheet3.zip › Input data and script2/DiseaseGene/SAA1 ~ SLC22A4.pdf]

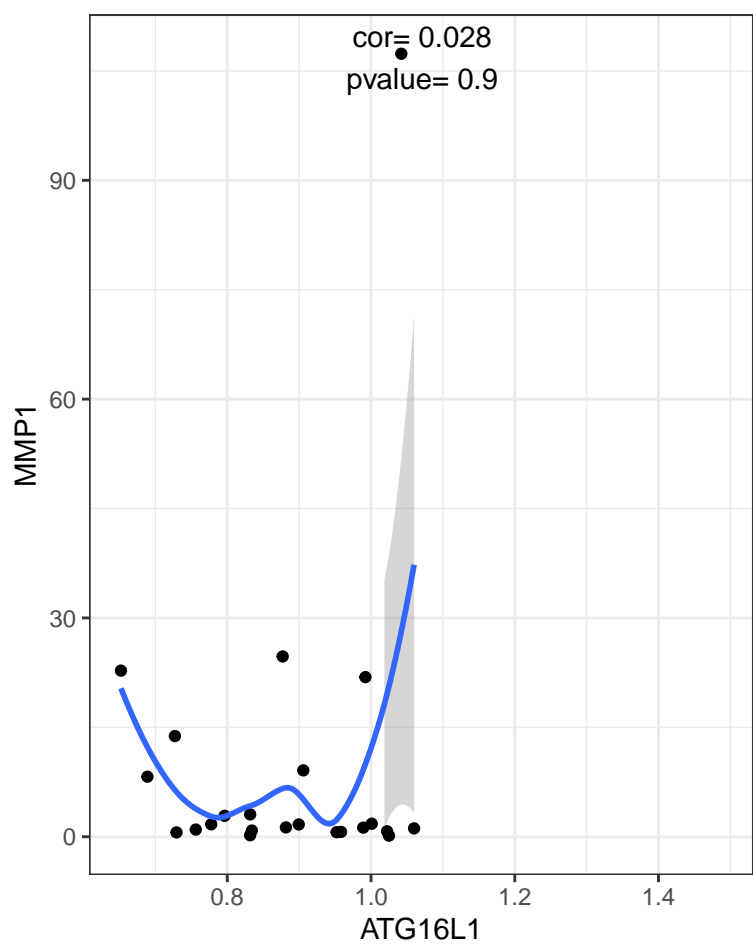

Supplement: Supplementary file 1 [file DataSheet3.zip › Input data and script2/DiseaseGene/MMP1 ~ ATG16L1.pdf]

Anova,  $p = 0.98$

MEFV Expression

1.2  
1.0  
0.8

Control

CD

Control  
CD

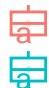

Supplement: Supplementary file 1 [file DataSheet3.zip › Input data and script2/DiseaseGene/MEFV.HealthyDisease.pdf]

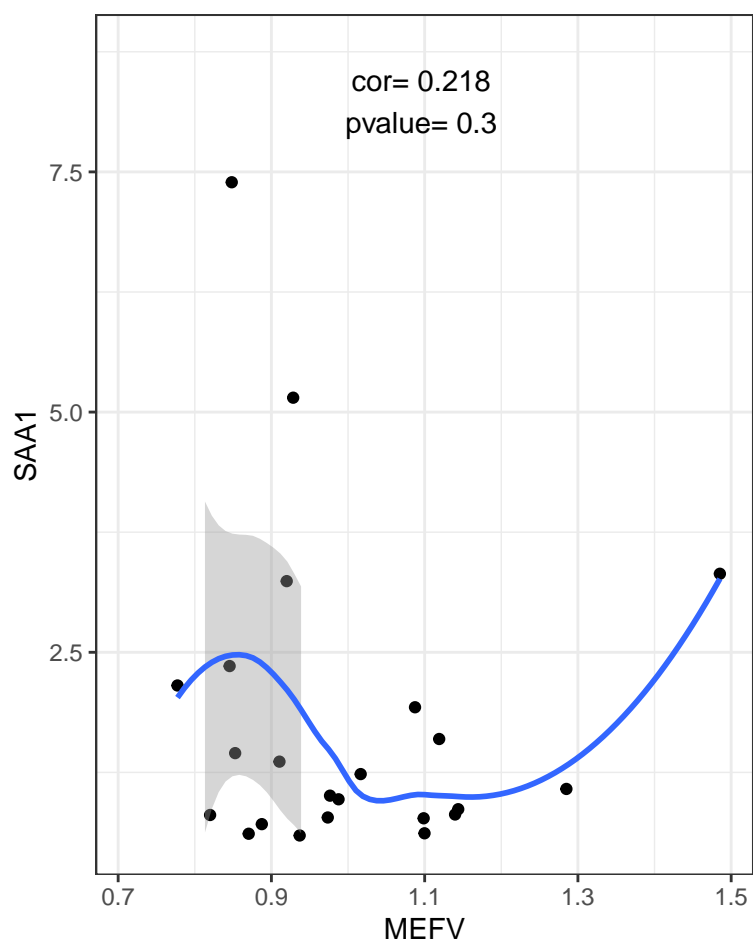

Supplement: Supplementary file 1 [file DataSheet3.zip › Input data and script2/DiseaseGene/SAA1 ~ MEFV.pdf]

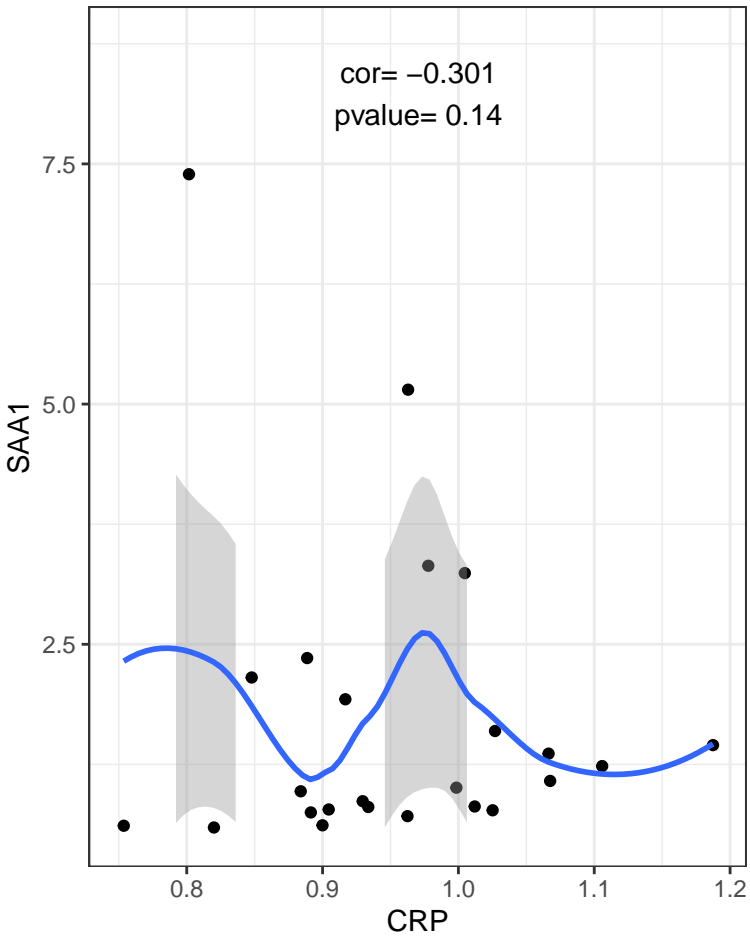

Supplement: Supplementary file 1 [file DataSheet3.zip › Input data and script2/DiseaseGene/SAA1 ~ CRP.pdf]

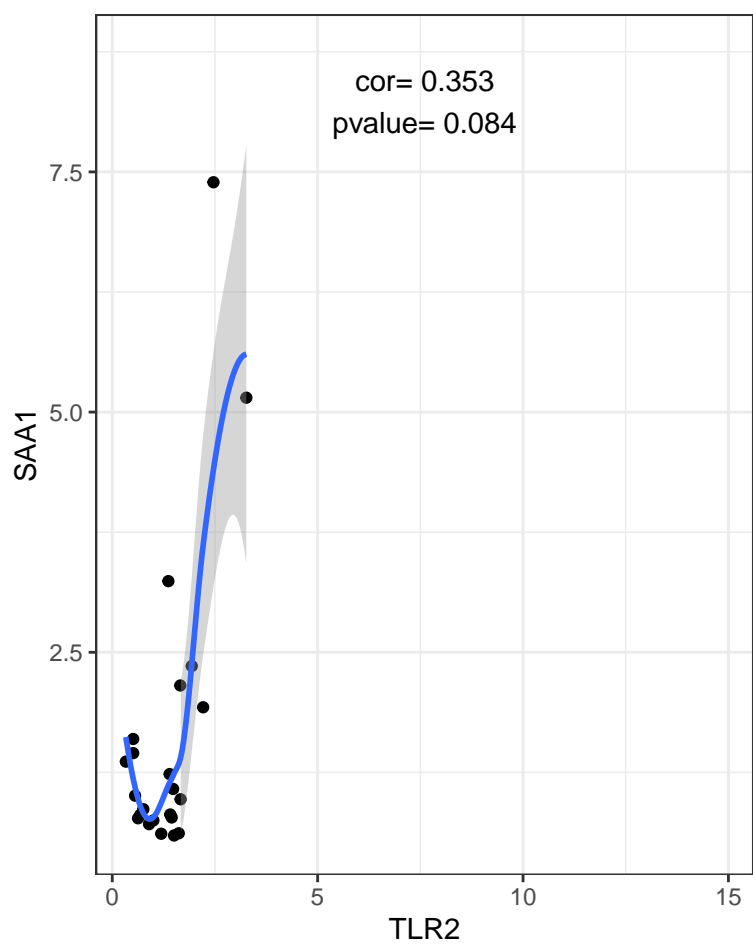

Supplement: Supplementary file 1 [file DataSheet3.zip › Input data and script2/DiseaseGene/SAA1 ~ TLR2.pdf]

Anova,  $p = 0.059$

TLR4 Expression

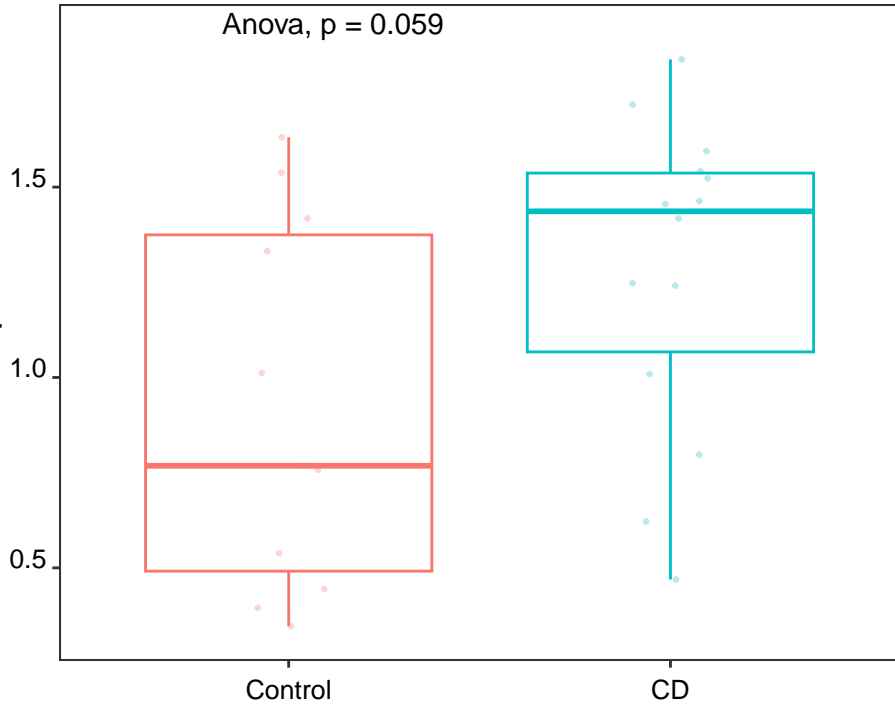

Control  
CD

Supplement: Supplementary file 1 [file DataSheet3.zip › Input data and script2/DiseaseGene/TLR4.HealthyDisease.pdf]

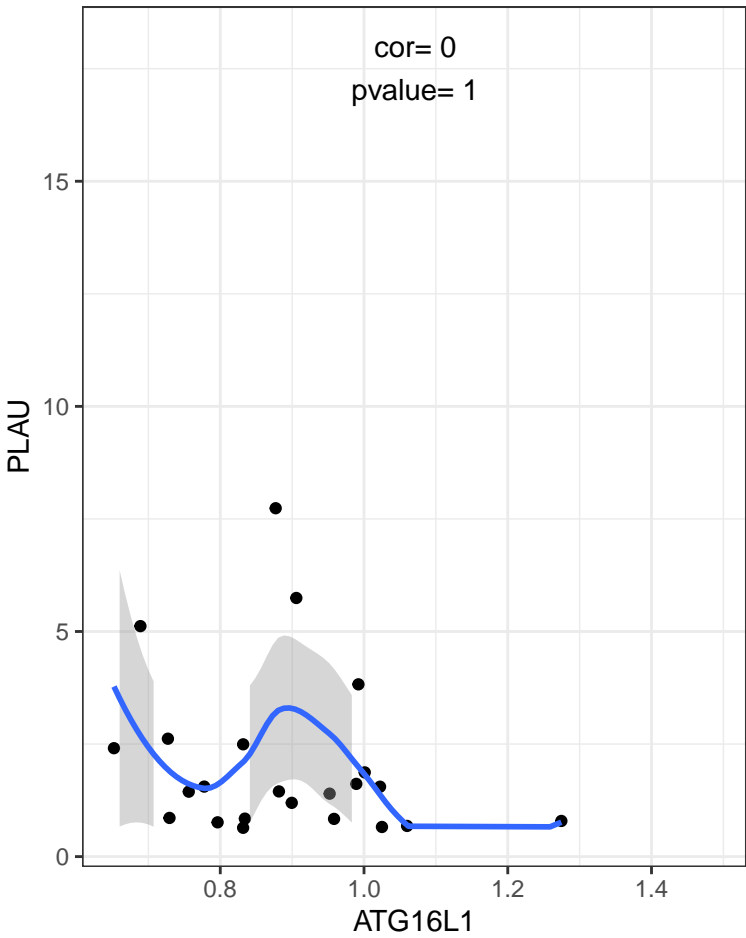

Supplement: Supplementary file 1 [file DataSheet3.zip › Input data and script2/DiseaseGene/PLAU ~ ATG16L1.pdf]

Anova,  $p = 0.018$

SAA1 Expression

3

2

1

Control

CD

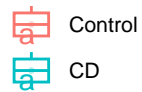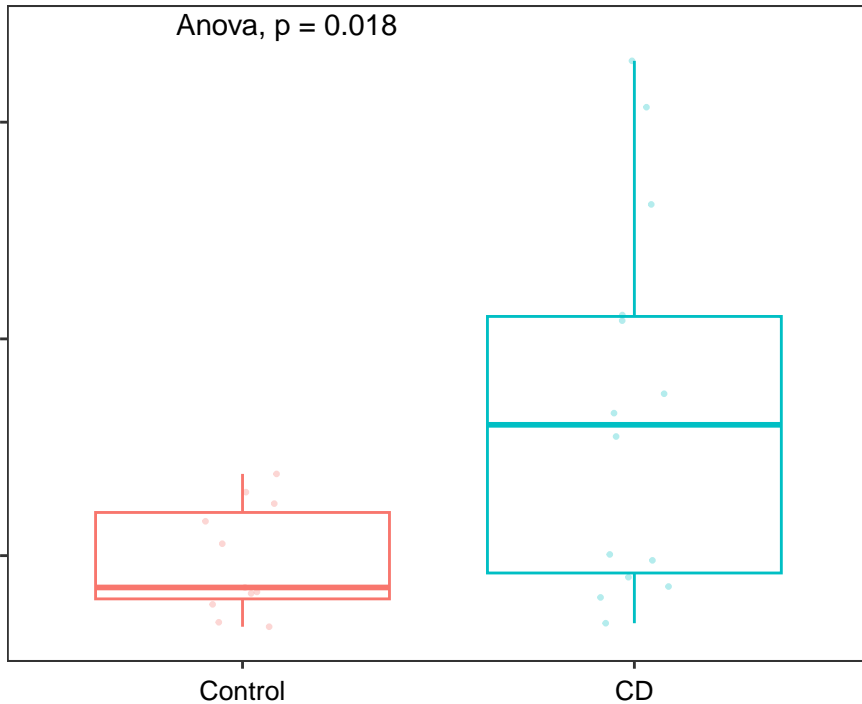

Supplement: Supplementary file 1 [file DataSheet3.zip › Input data and script2/DiseaseGene/SAA1.HealthyDisease.pdf]

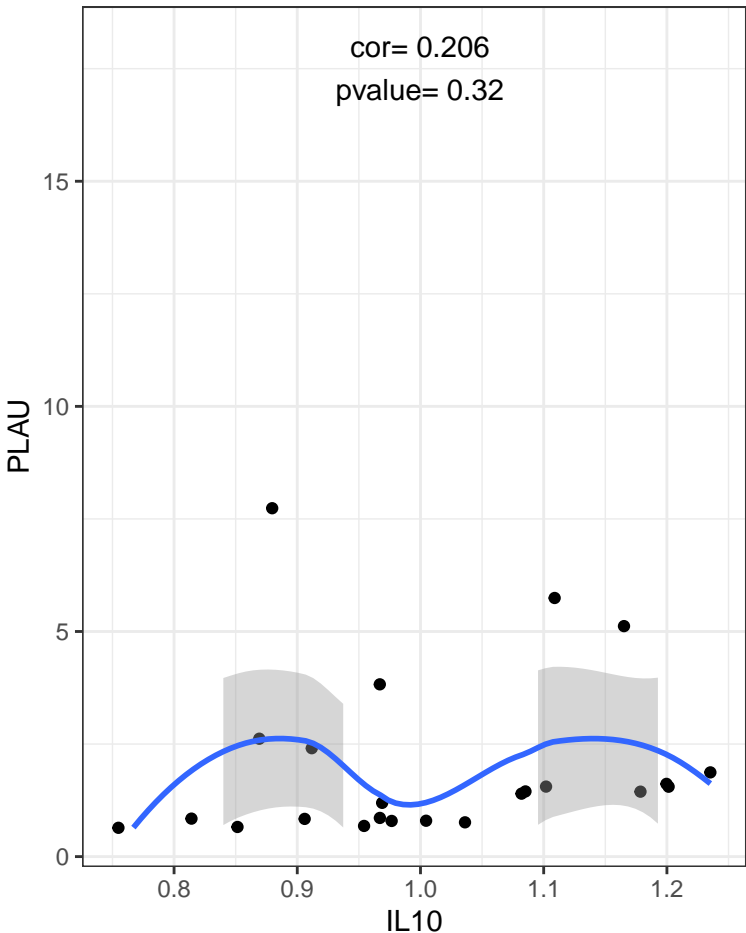

Supplement: Supplementary file 1 [file DataSheet3.zip › Input data and script2/DiseaseGene/PLAU ~ IL10.pdf]

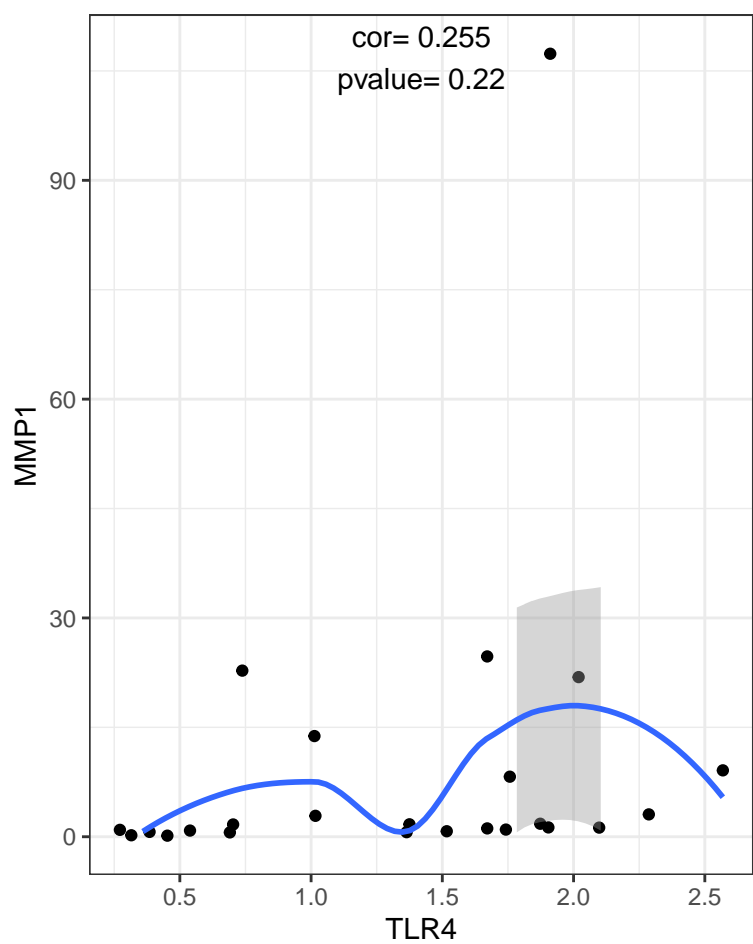

Supplement: Supplementary file 1 [file DataSheet3.zip › Input data and script2/DiseaseGene/MMP1 ~ TLR4.pdf]

Anova,  $p = 0.004$

MMP1 Expression

6

4

2

0

Control

CD

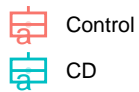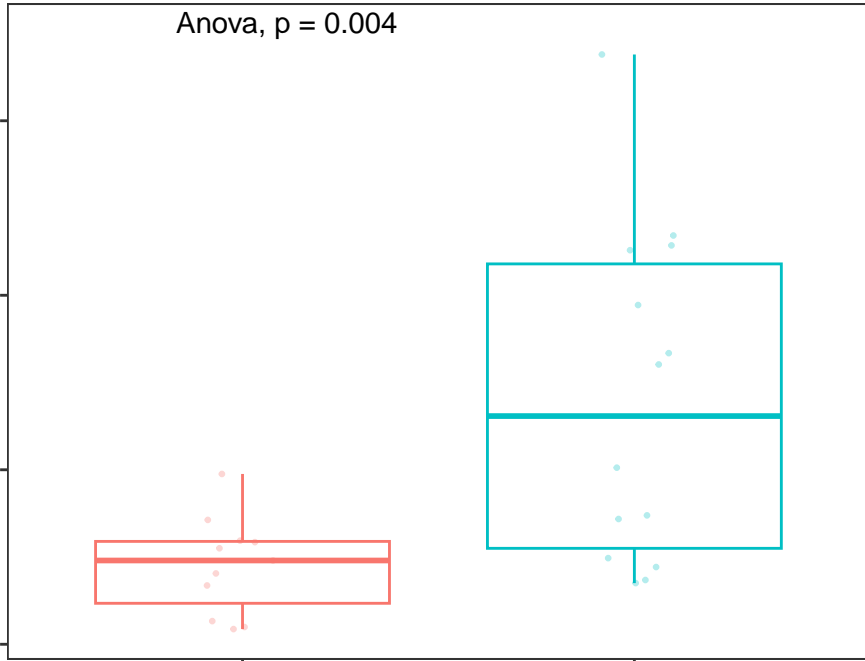

Supplement: Supplementary file 1 [file DataSheet3.zip › Input data and script2/DiseaseGene/MMP1.HealthyDisease.pdf]

Anova,  $p = 0.0046$

NOD2 Expression

3

2

1

Control

CD

Control  
CD

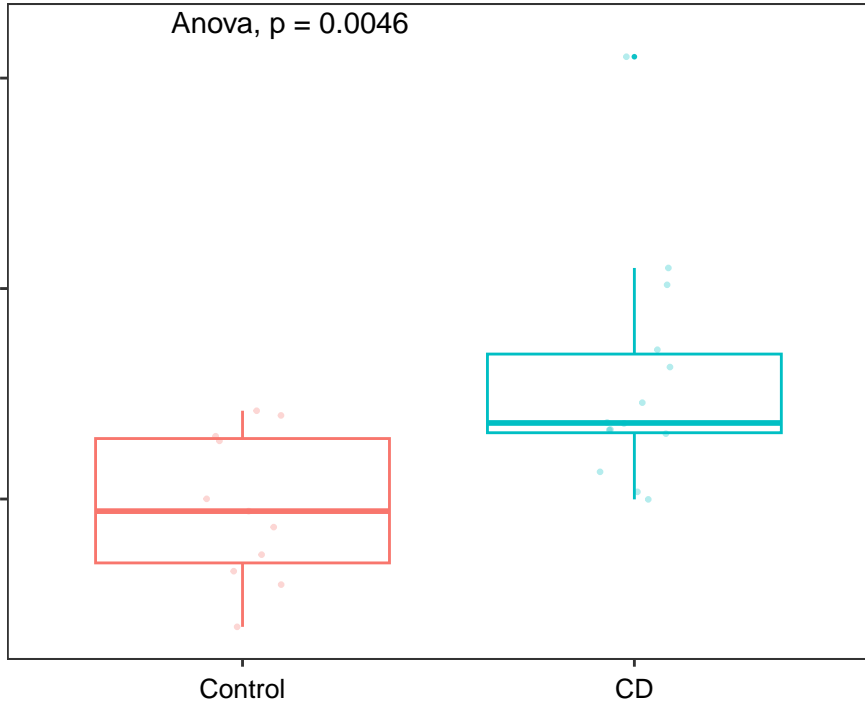

Supplement: Supplementary file 1 [file DataSheet3.zip › Input data and script2/DiseaseGene/NOD2.HealthyDisease.pdf]

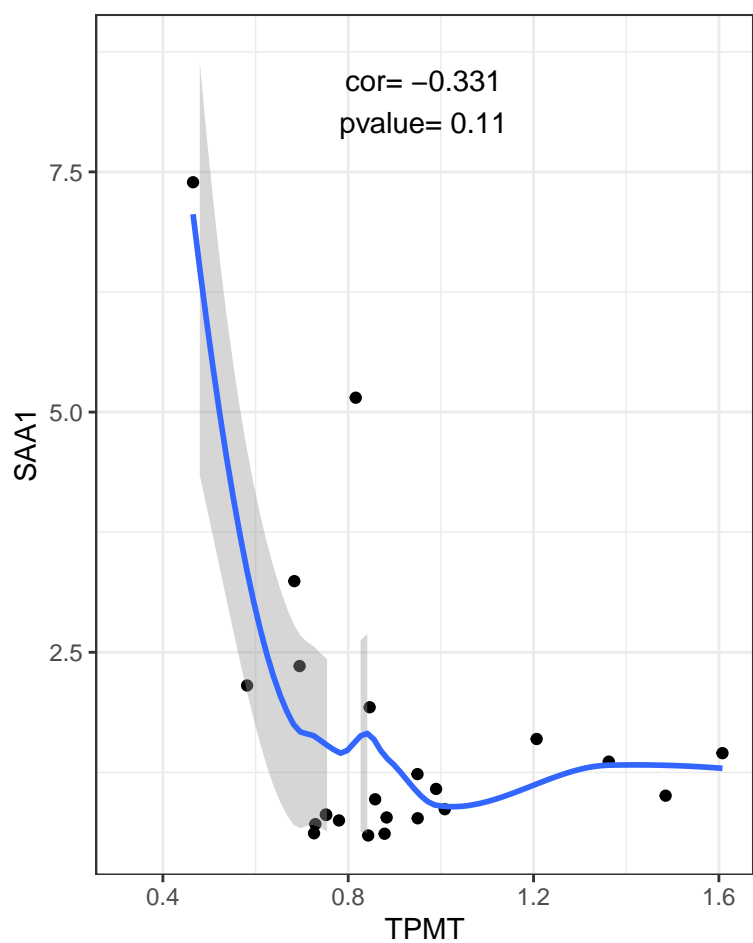

Supplement: Supplementary file 1 [file DataSheet3.zip › Input data and script2/DiseaseGene/SAA1 ~ TPMT.pdf]

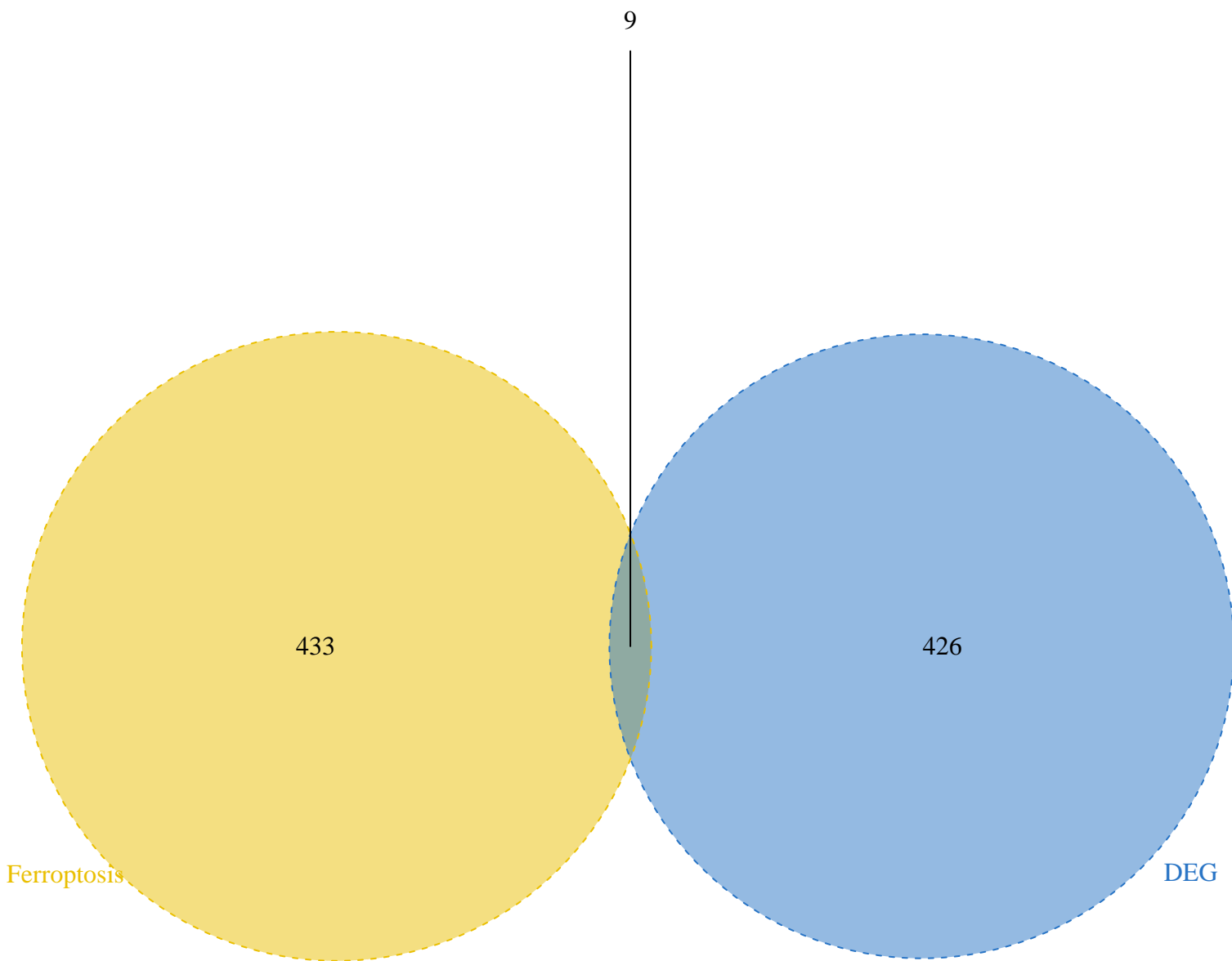

Supplement: Supplementary file 1 [file DataSheet3.zip › Input data and script2/venn/Venn_Ferroptosis.pdf]

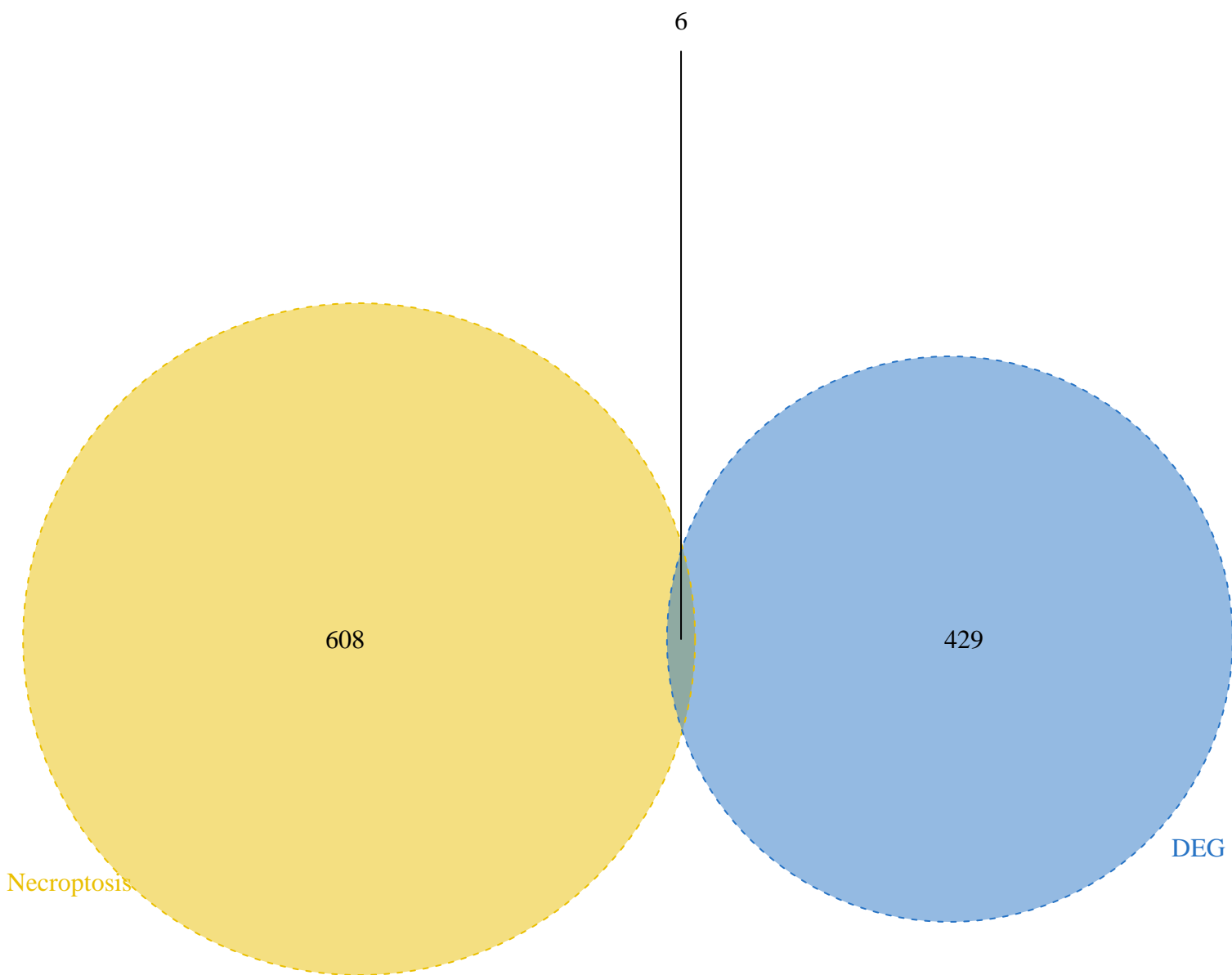

Supplement: Supplementary file 1 [file DataSheet3.zip › Input data and script2/venn/Venn_Necroptosis.pdf]

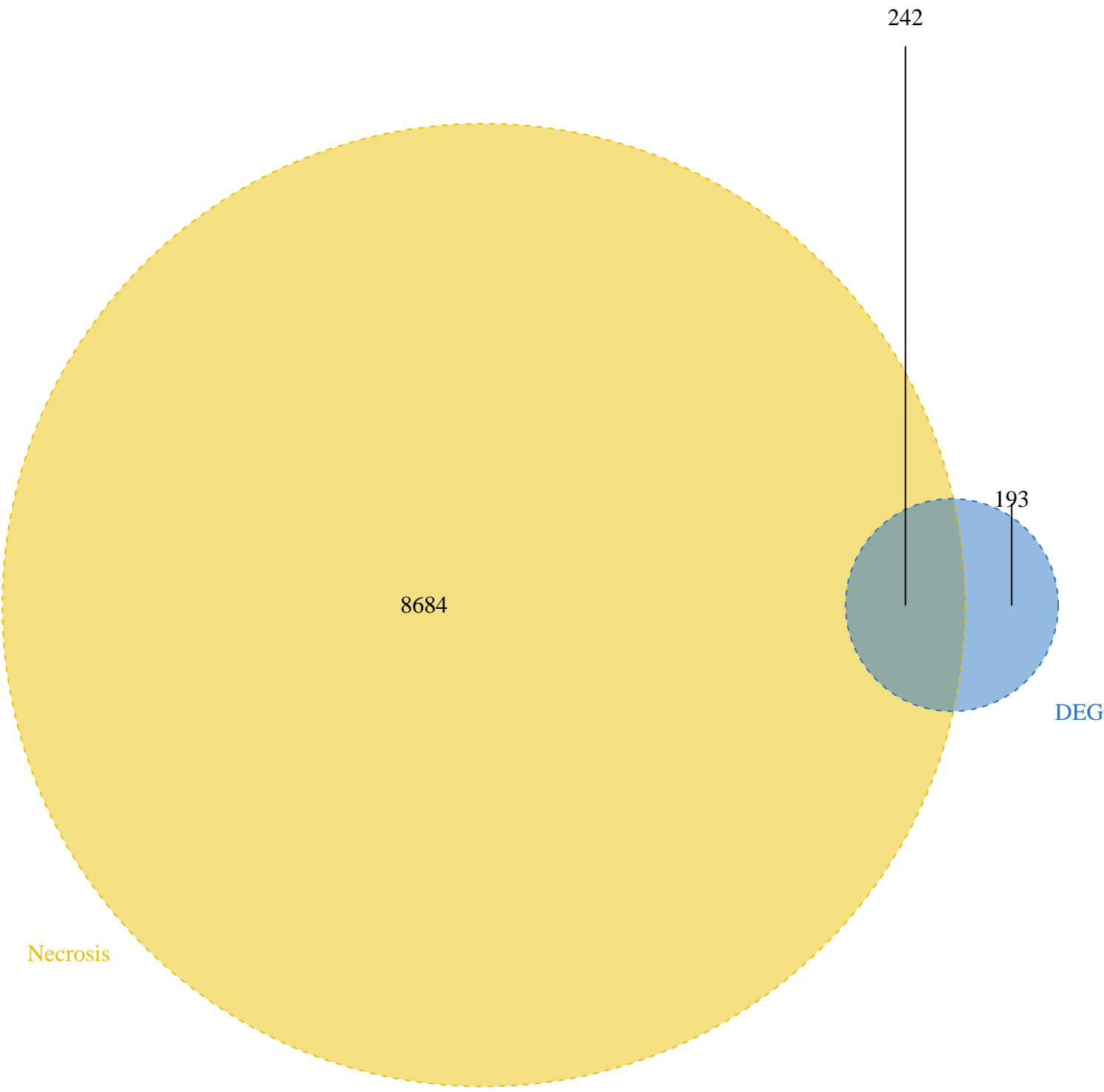

Supplement: Supplementary file 1 [file DataSheet3.zip › Input data and script2/venn/Venn_Necrosis.pdf]

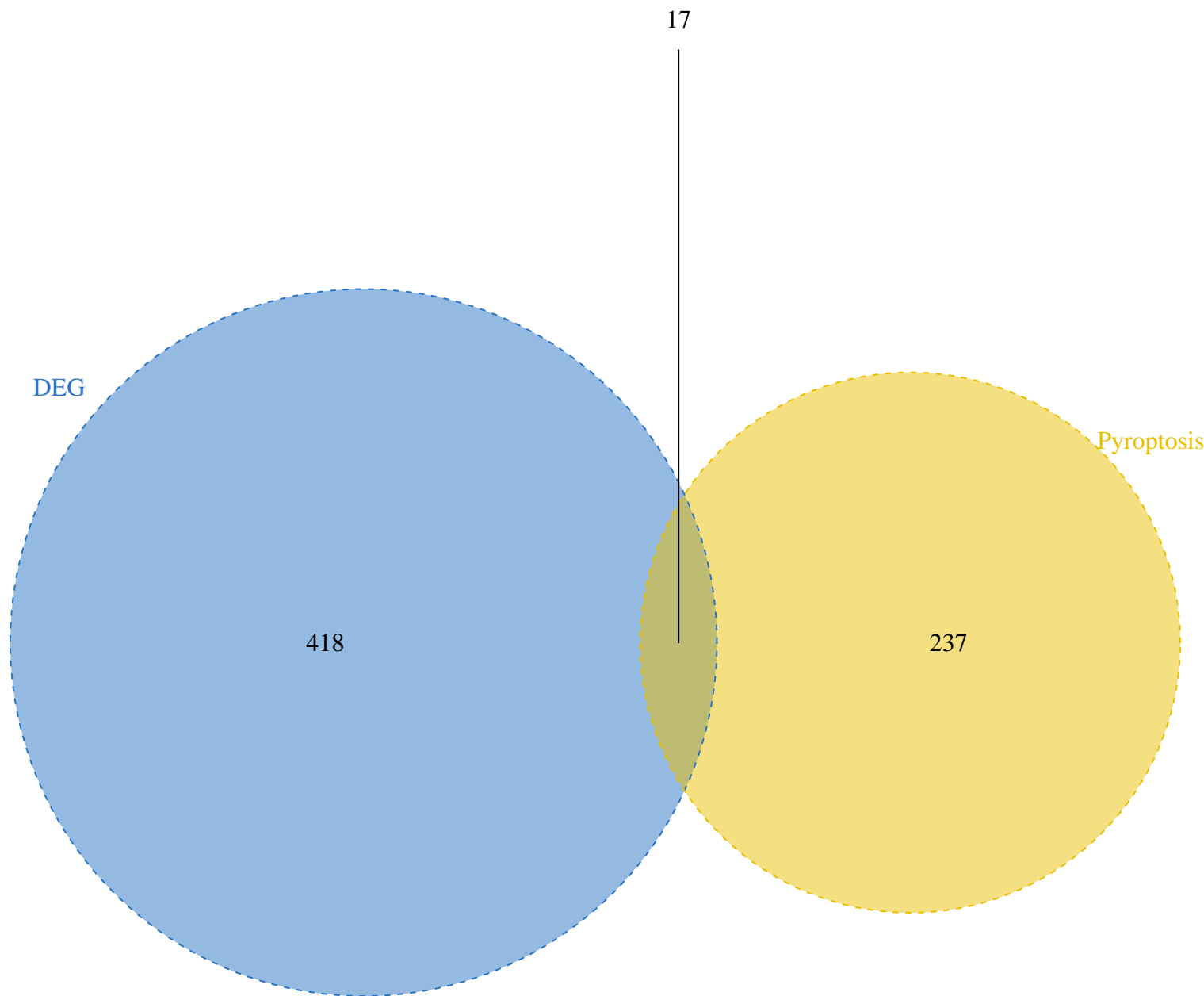

Supplement: Supplementary file 1 [file DataSheet3.zip › Input data and script2/venn/Venn_Pyroptosis.pdf]

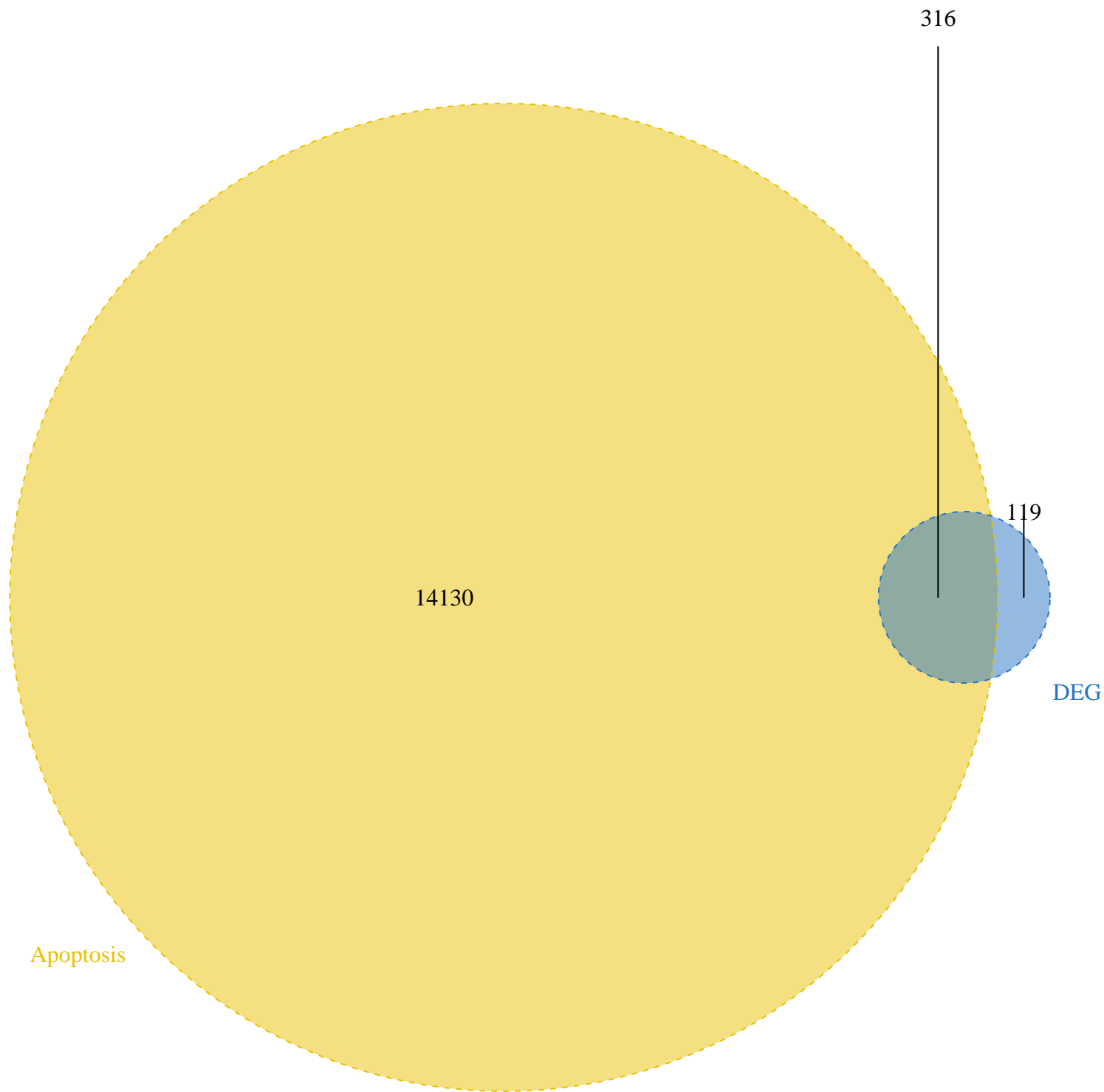

Supplement: Supplementary file 1 [file DataSheet3.zip › Input data and script2/venn/Venn_Apoptosis.pdf]

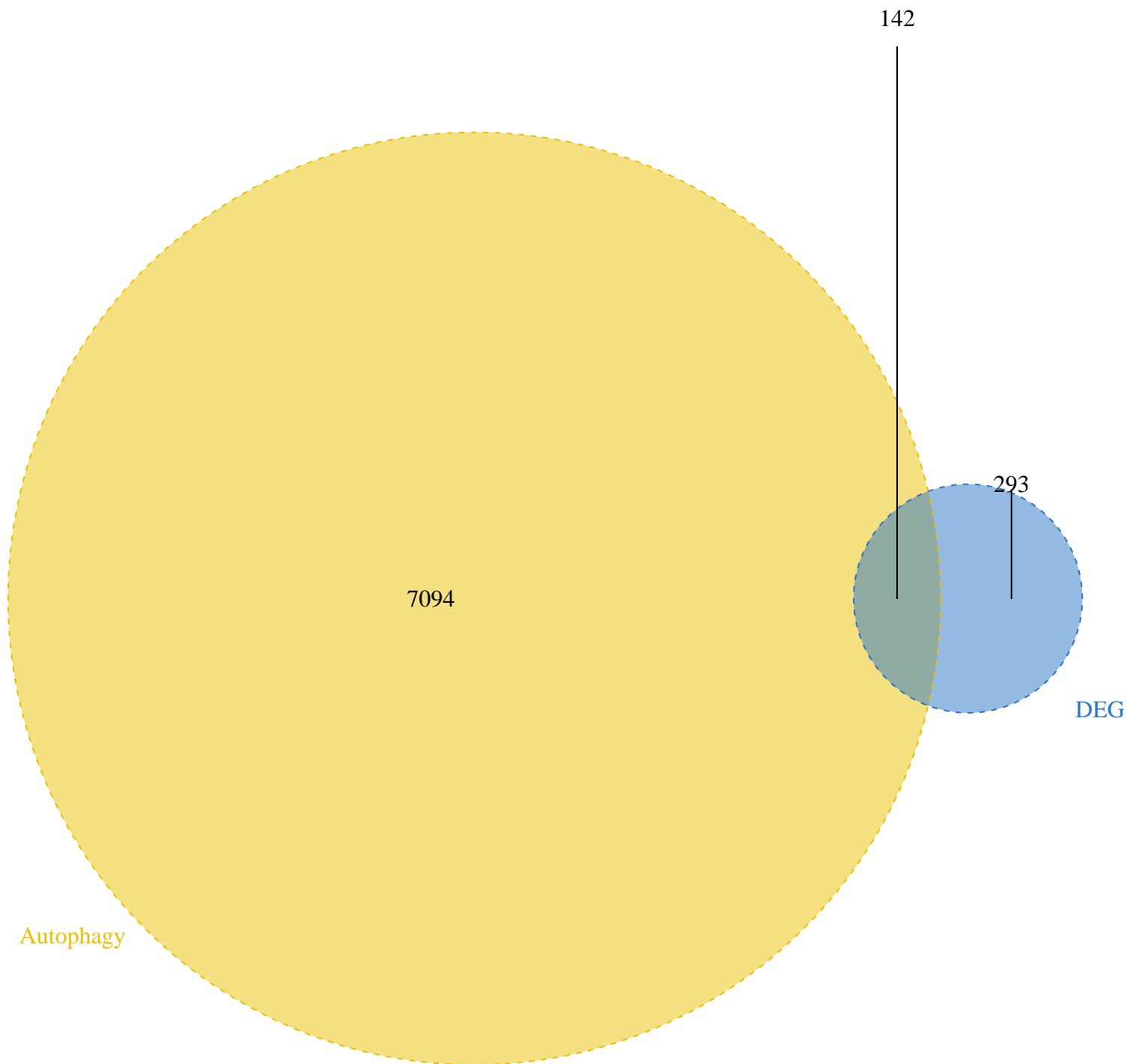

Supplement: Supplementary file 1 [file DataSheet3.zip › Input data and script2/venn/Venn_Autophagy.pdf]

rogrammedCellDeath

167

3

25

MCOD

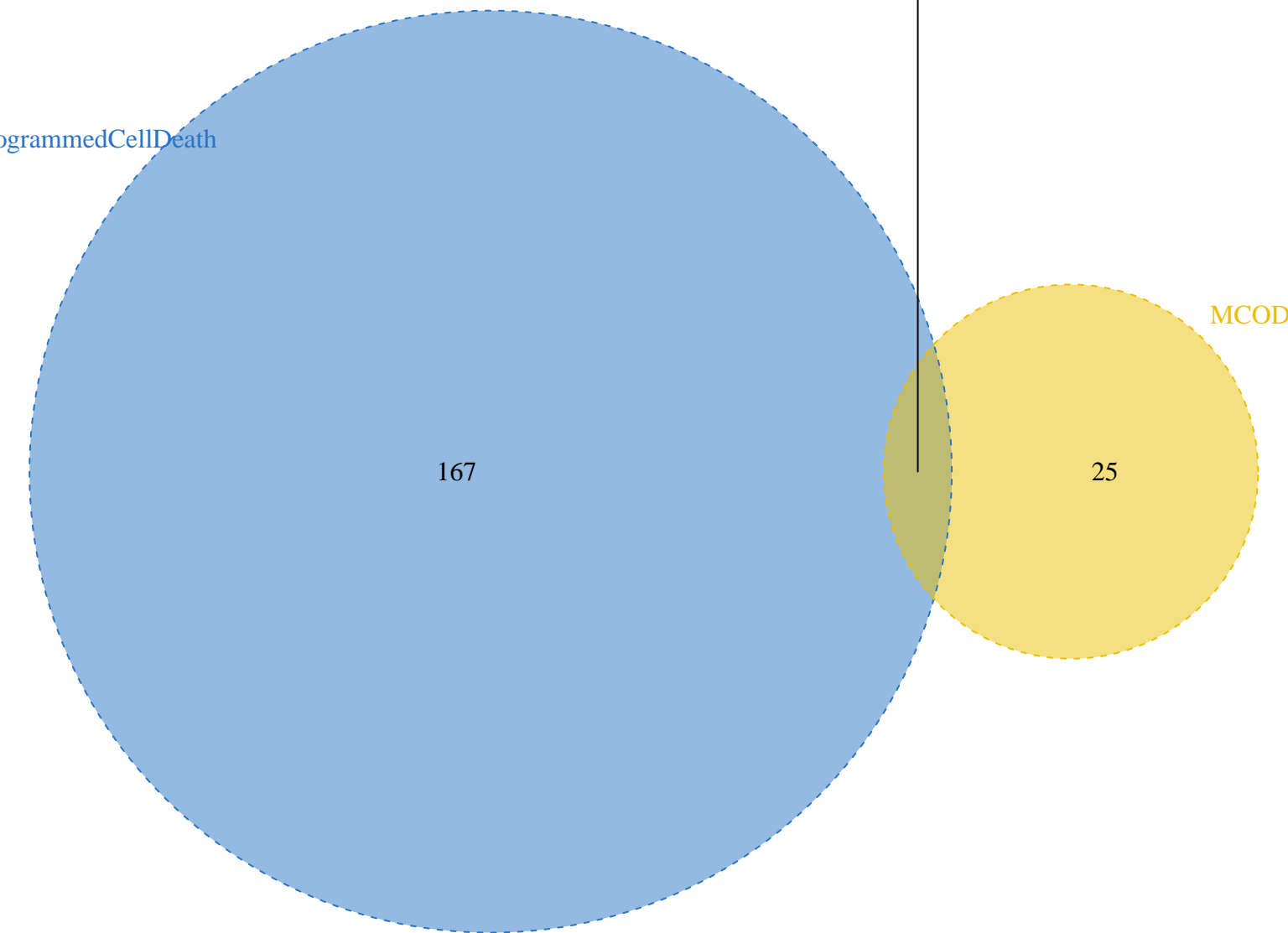

Supplement: Supplementary file 1 [file DataSheet3.zip › Input data and script2/venn/Venn_UP.pdf]

# Volcano

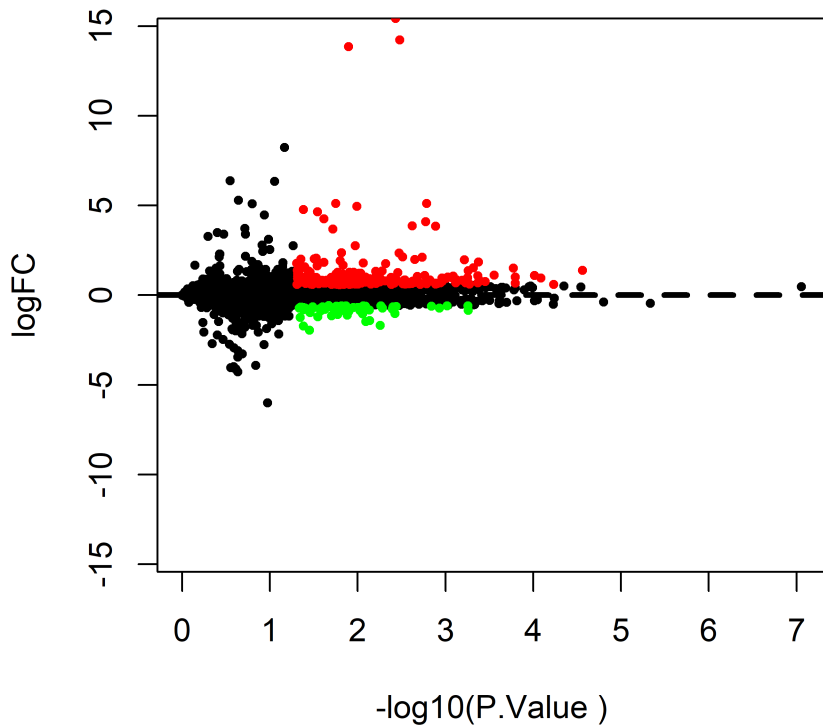

Supplement: Supplementary file 1 [file DataSheet3.zip › Input data and script2/diff/volcano-GSE10616.pdf]

# Volcano

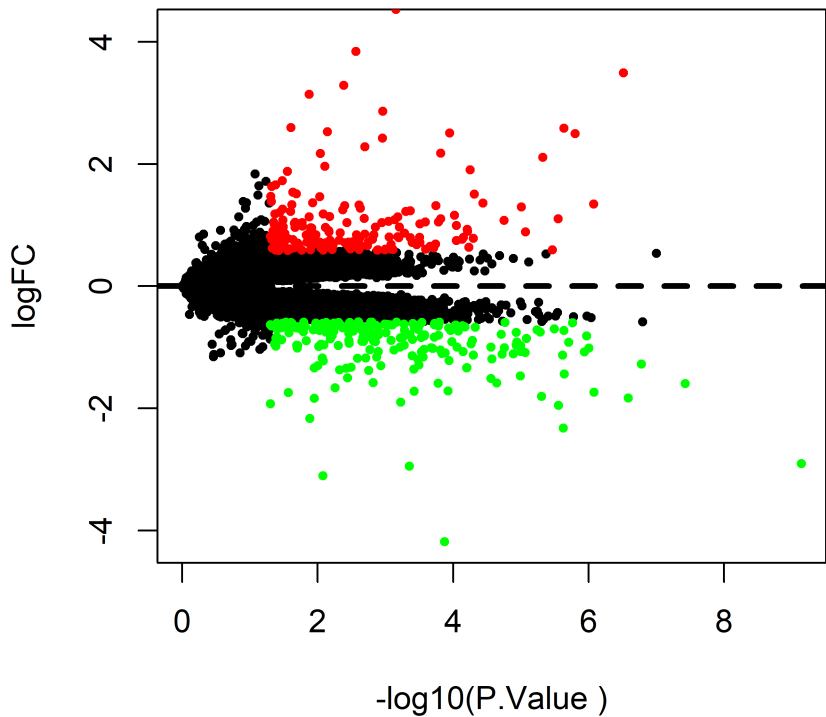

Supplement: Supplementary file 1 [file DataSheet3.zip › Input data and script2/diff/volcano-GSE36807.pdf]

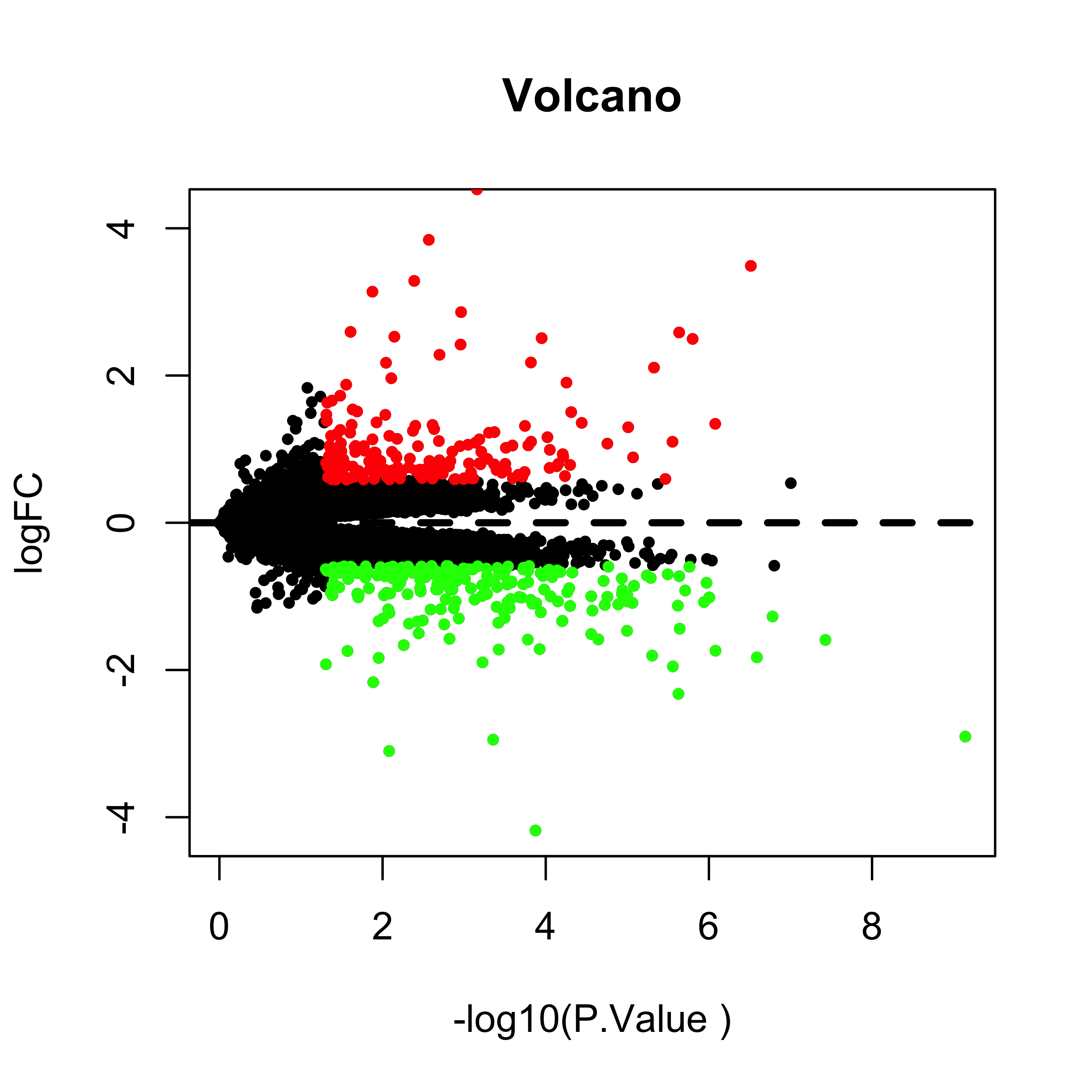

Supplement: Supplementary file 1 [file DataSheet3.zip › Input data and script2/diff/volcano-GSE36807.tiff]

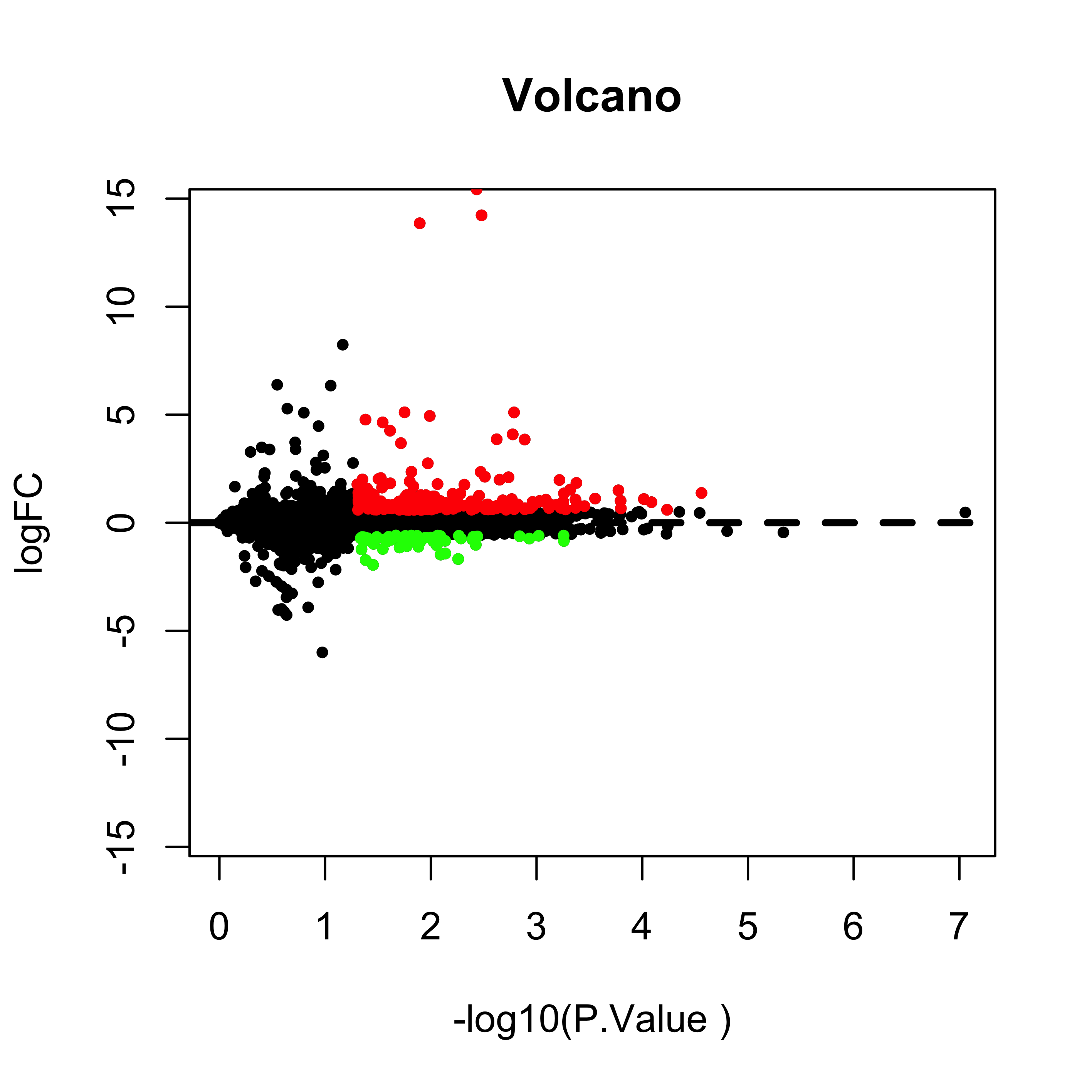

Supplement: Supplementary file 1 [file DataSheet3.zip › Input data and script2/diff/volcano-GSE10616.tiff]

Enrichment Score

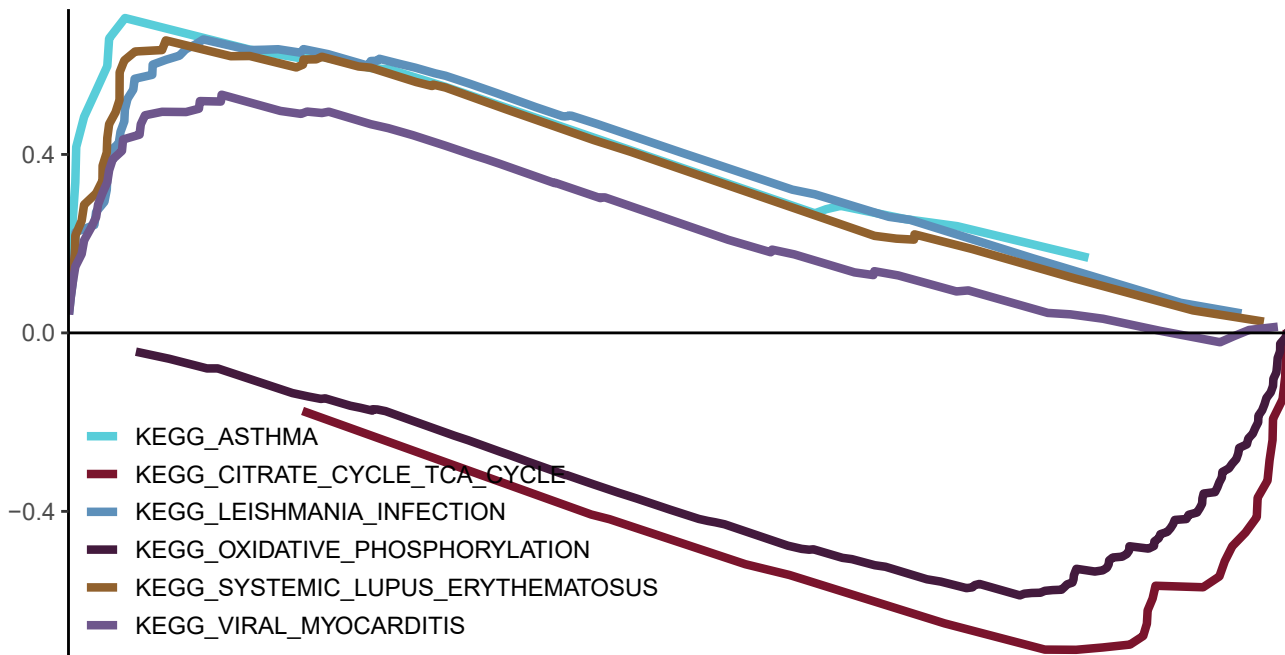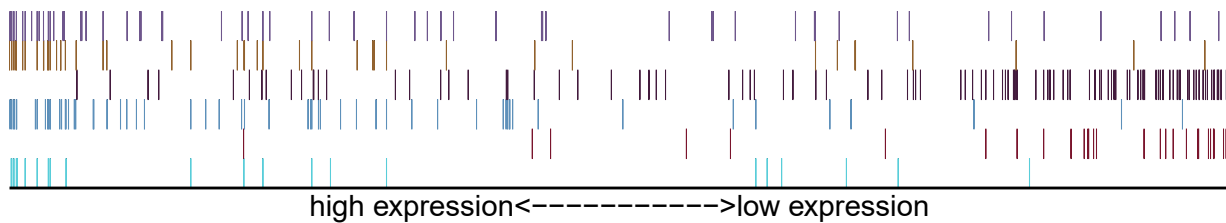

Supplement: Supplementary file 1 [file DataSheet3.zip › Input data and script2/GSEA analysis/PLAU/2.GSEA KEGG.pdf]

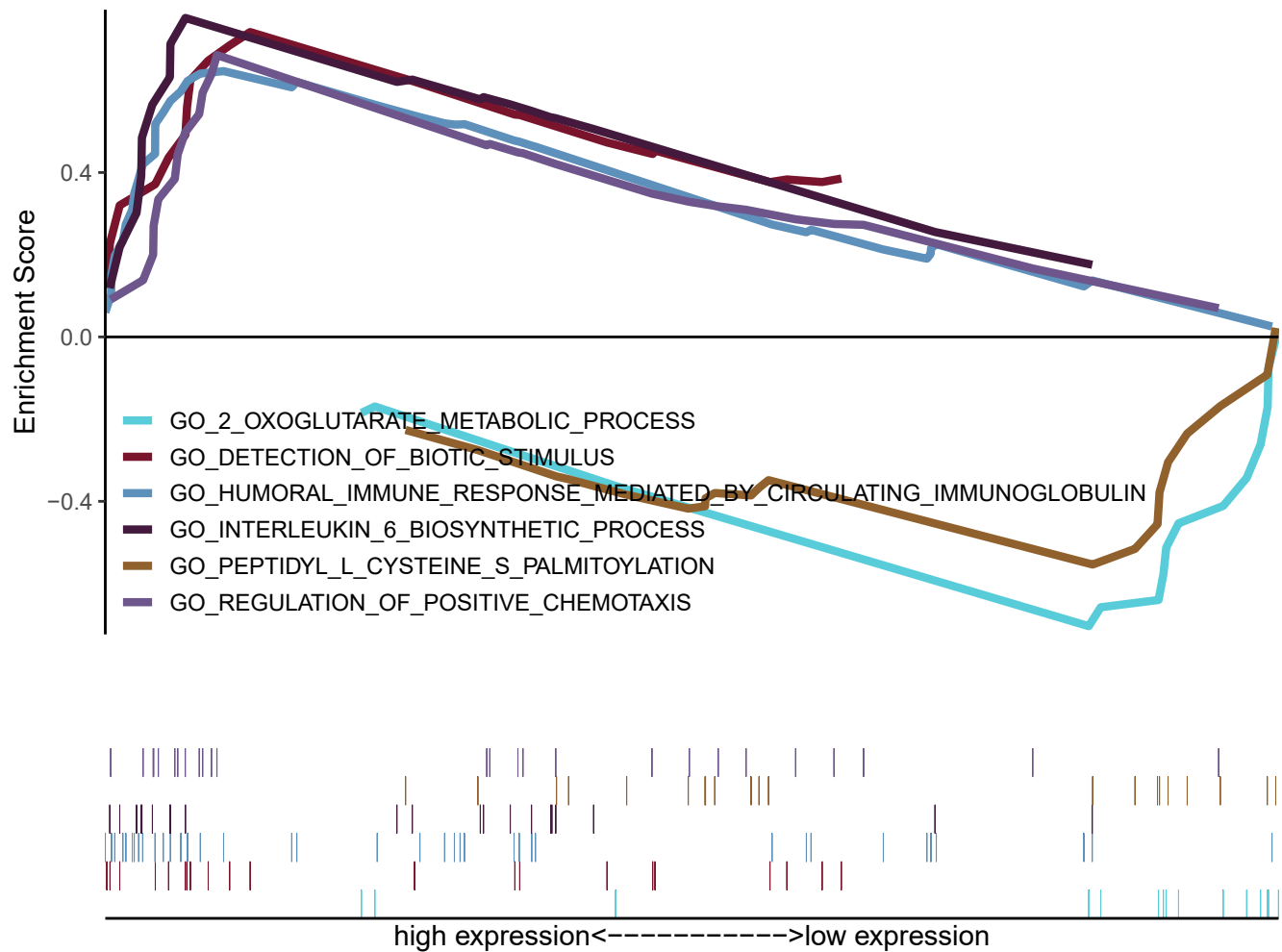

Supplement: Supplementary file 1 [file DataSheet3.zip › Input data and script2/GSEA analysis/PLAU/1.GSEA GO.pdf]
