# Supplementary material for: Identification of hub programmed cell death-related genes and immune infiltration in Crohn’s disease using bioinformatics
Source: Front Genet. 2024 Dec 18;15:1425062. doi: 10.3389/fgene.2024.1425062 (PMC11688285; doi:10.3389/fgene.2024.1425062)
Supplement: Supplementary file 2 [file DataSheet4.zip › Input data and script3/Xcell-Immune infiltration/Immunomodulator_and_chemokines ~ receptor.pdf]

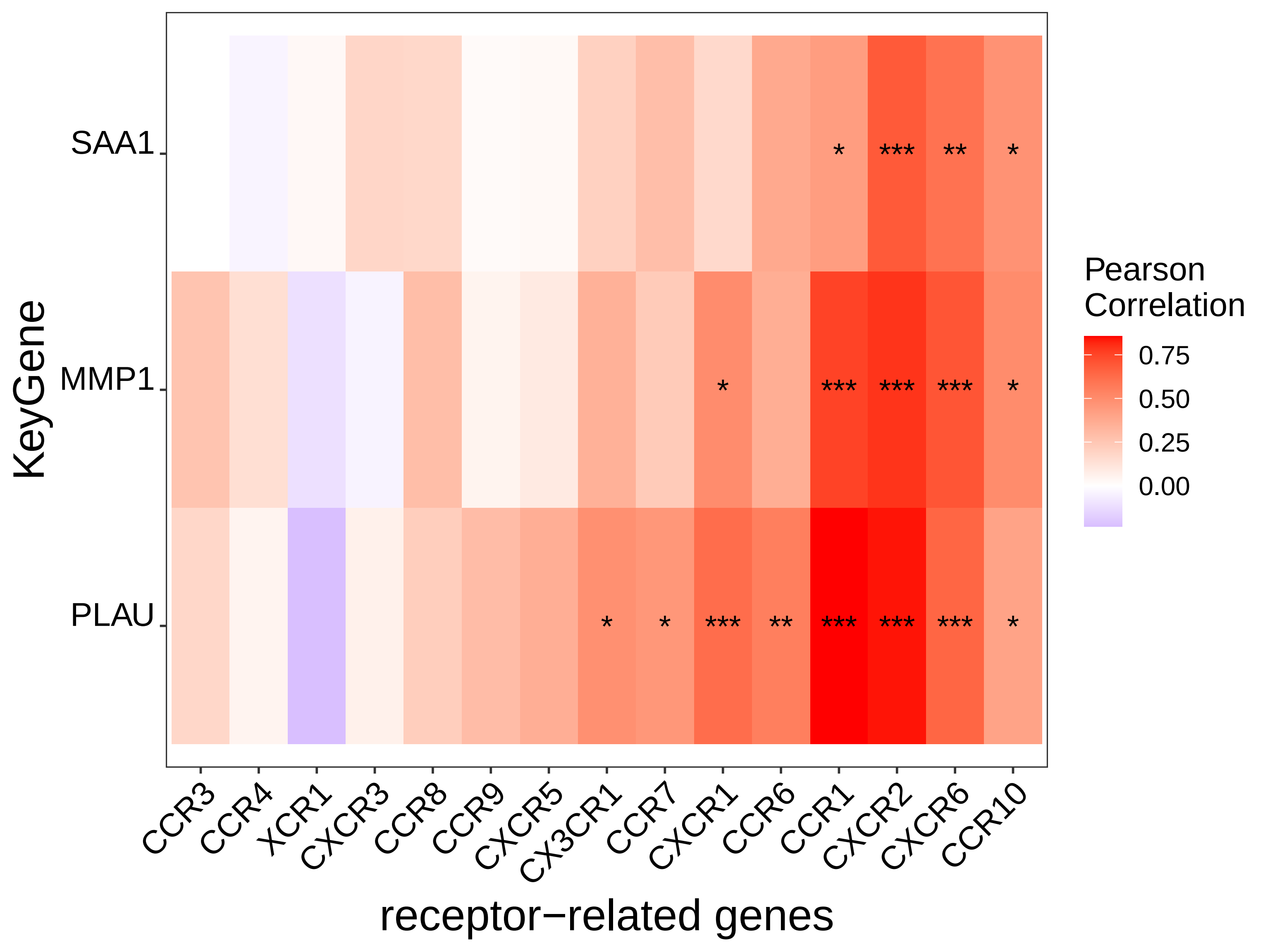

KeyGene
SAA1
MMP1
PLAU
*
***
**
*
*
***
***
***
*
*
*
***
**
***
***
***
*
Pearson
Correlation
0.75
0.50
0.25
0.00
CCR3
CCR4
XCR1
CXCR3
CCR8
CCR9
CXCR5
CX3CR1
CCR7
CXCR1
CCR6
CCR1
CXCR2
CXCR6
CCR10
receptor−related genes
