# Supplementary material for: Identification of hub programmed cell death-related genes and immune infiltration in Crohn’s disease using bioinformatics
Source: Front Genet. 2024 Dec 18;15:1425062. doi: 10.3389/fgene.2024.1425062 (PMC11688285; doi:10.3389/fgene.2024.1425062)
Supplement: Supplementary file 2 [file DataSheet4.zip › Input data and script3/Xcell-Immune infiltration/Immunomodulator_and_chemokines ~ chemokine.pdf]

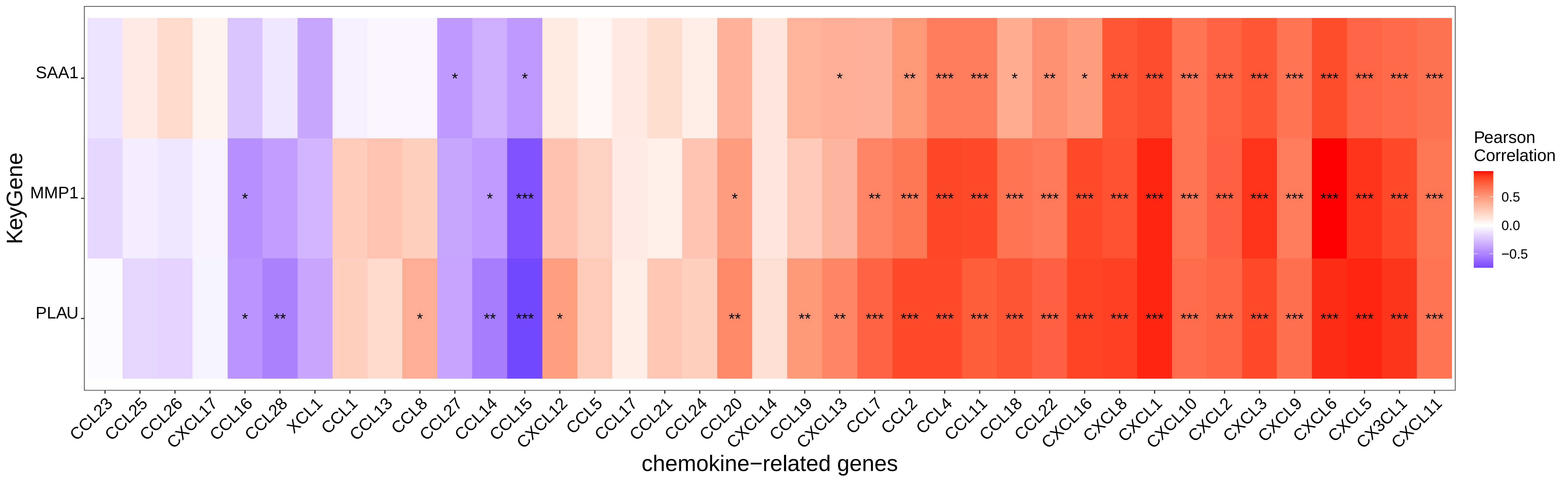

KeyGene
SAA1
MMP1
PLAU
CCL23
CCL25
CCL26
CXCL17
CCL16
CCL28
XCL1
CCL1
CCL13
CCL8
CCL27
CCL14
CCL15
CXCL12
CCL5
CCL17
CCL21
CCL24
CCL20
CXCL14
CCL19
CXCL13
CCL7
CCL2
CCL4
CCL11
CCL18
CCL22
CXCL16
CXCL8
CXCL1
CXCL10
CXCL2
CXCL3
CXCL9
CXCL6
CXCL5
CX3CL1
CXCL11
chemokine−related genes
Pearson Correlation
0.5
0.0
−0.5
