# Supplementary material for: Identification of hub programmed cell death-related genes and immune infiltration in Crohn’s disease using bioinformatics
Source: Front Genet. 2024 Dec 18;15:1425062. doi: 10.3389/fgene.2024.1425062 (PMC11688285; doi:10.3389/fgene.2024.1425062)
Supplement: Supplementary file 2 [file DataSheet4.zip › Input data and script3/Xcell-Immune infiltration/Immunomodulator_and_chemokines ~ Immunostimulator.pdf]

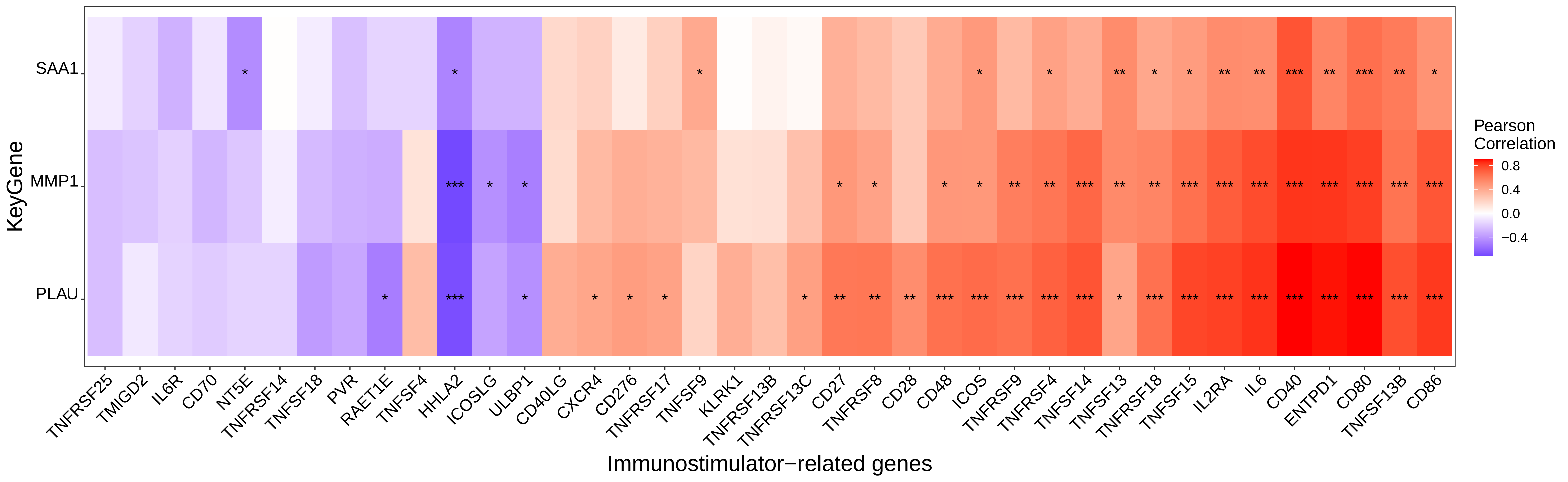

SAA1
MMP1
PLAU
KeyGene
TNFRSF25
TMIGD2
IL6R
CD70
NT5E
TNFRSF14
TNFSF18
PVR
RAET1E
TNFSF4
HHLA2
ICOSLG
ULBP1
CD40LG
CXCR4
CD276
TNFRSF17
TNFSF9
KLRK1
TNFRSF13B
TNFRSF13C
CD27
TNFRSF8
CD28
CD48
ICOS
TNFRSF9
TNFRSF4
TNFSF14
TNFSF13
TNFRSF18
TNFSF15
IL2RA
IL6
CD40
ENTPD1
CD80
TNFSF13B
CD86
Immunostimulator−related genes
Pearson Correlation
0.8
0.4
0.0
−0.4
