# Supplementary material for: Identification of hub programmed cell death-related genes and immune infiltration in Crohn’s disease using bioinformatics
Source: Front Genet. 2024 Dec 18;15:1425062. doi: 10.3389/fgene.2024.1425062 (PMC11688285; doi:10.3389/fgene.2024.1425062)
Supplement: Supplementary file 2 [file DataSheet4.zip › Input data and script3/Xcell-Immune infiltration/Immunomodulator_and_chemokines ~ Immunoinhibitor.pdf]

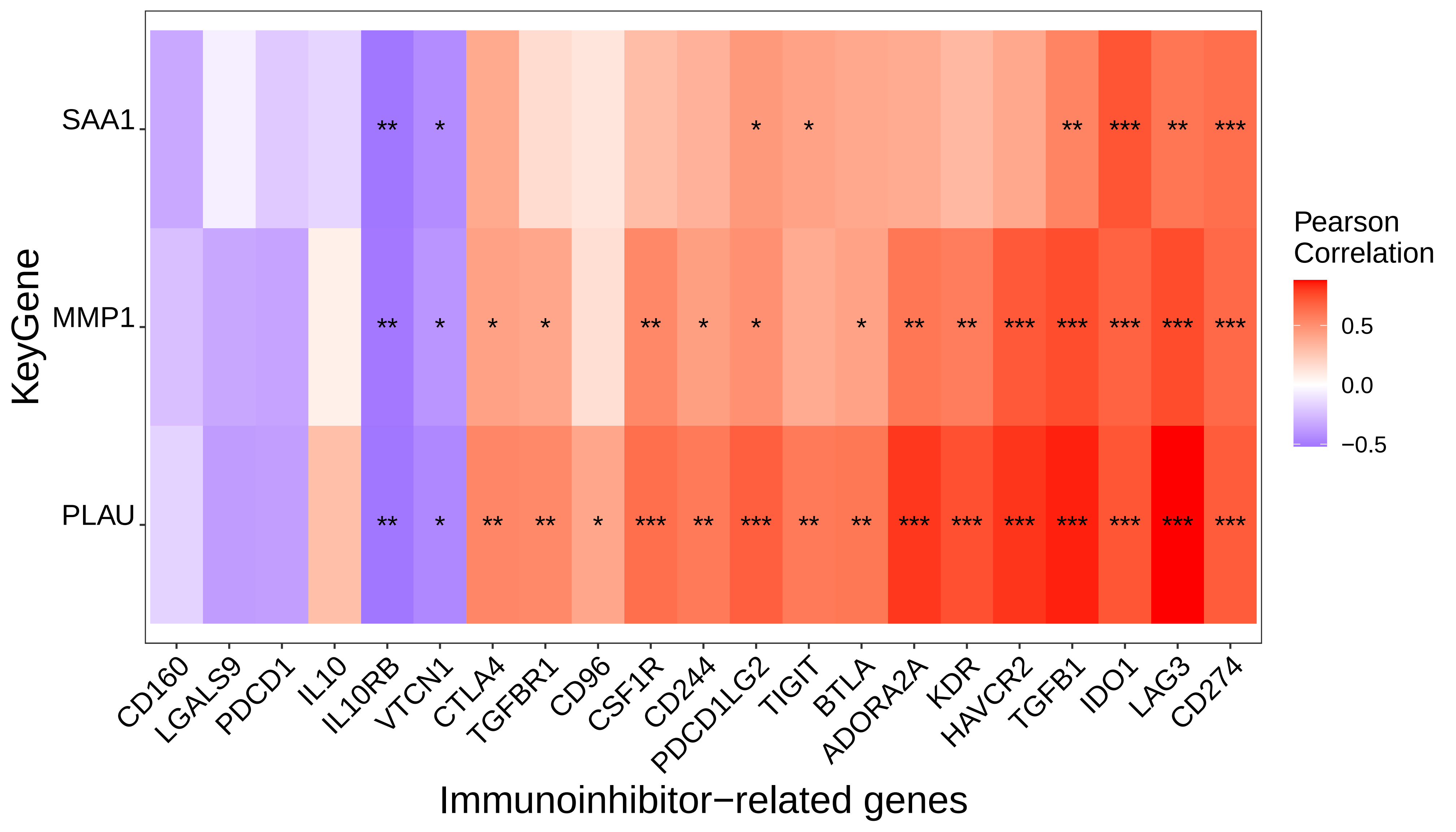

KeyGene
SAA1
MMP1
PLAU
** * * * ** *** ** ***
** * * * ** * * * ** ** *** *** *** *** ***
** * ** ** * *** ** *** ** ** *** *** *** *** *** *** ***
CD160
LGALS9
PDCD1
IL10
IL10RB
VTCN1
CTLA4
TGFBR1
CD96
CSF1R
CD244
PDCD1LG2
TIGIT
BTLA
ADORA2A
KDR
HAVCR2
TGFB1
IDO1
LAG3
CD274
Immunoinhibitor−related genes
Pearson Correlation
0.5
0.0
−0.5
