# Supplementary material for: Identification of hub programmed cell death-related genes and immune infiltration in Crohn’s disease using bioinformatics
Source: Front Genet. 2024 Dec 18;15:1425062. doi: 10.3389/fgene.2024.1425062 (PMC11688285; doi:10.3389/fgene.2024.1425062)
Supplement: Supplementary file 2 [file DataSheet4.zip › Input data and script3/Xcell-Immune infiltration/Immunomodulator_and_chemokines ~ MHC.pdf]

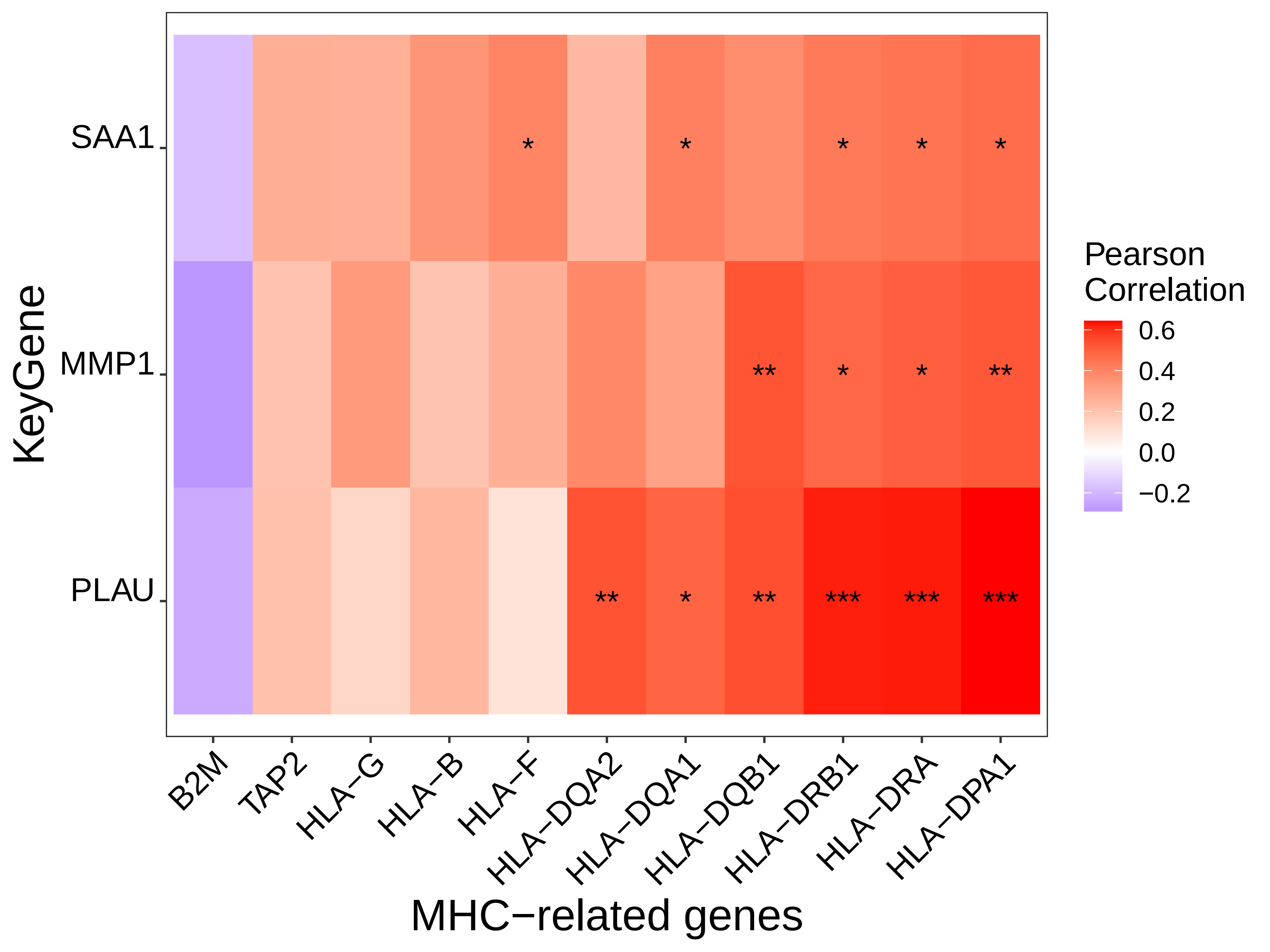

Pearson Correlation
0.6
0.4
0.2
0.0
−0.2
KeyGene
SAA1
MMP1
PLAU
B2M
TAP2
HLA−G
HLA−B
HLA−F
HLA−DQA2
HLA−DQA1
HLA−DQB1
HLA−DRB1
HLA−DRA
HLA−DPA1
MHC−related genes
