# Supplementary material for: Identification of hub programmed cell death-related genes and immune infiltration in Crohn’s disease using bioinformatics
Source: Front Genet. 2024 Dec 18;15:1425062. doi: 10.3389/fgene.2024.1425062 (PMC11688285; doi:10.3389/fgene.2024.1425062)
Supplement: Supplementary file 3 [file DataSheet1.pdf]

Supplementary Material

1 Supplementary Figures

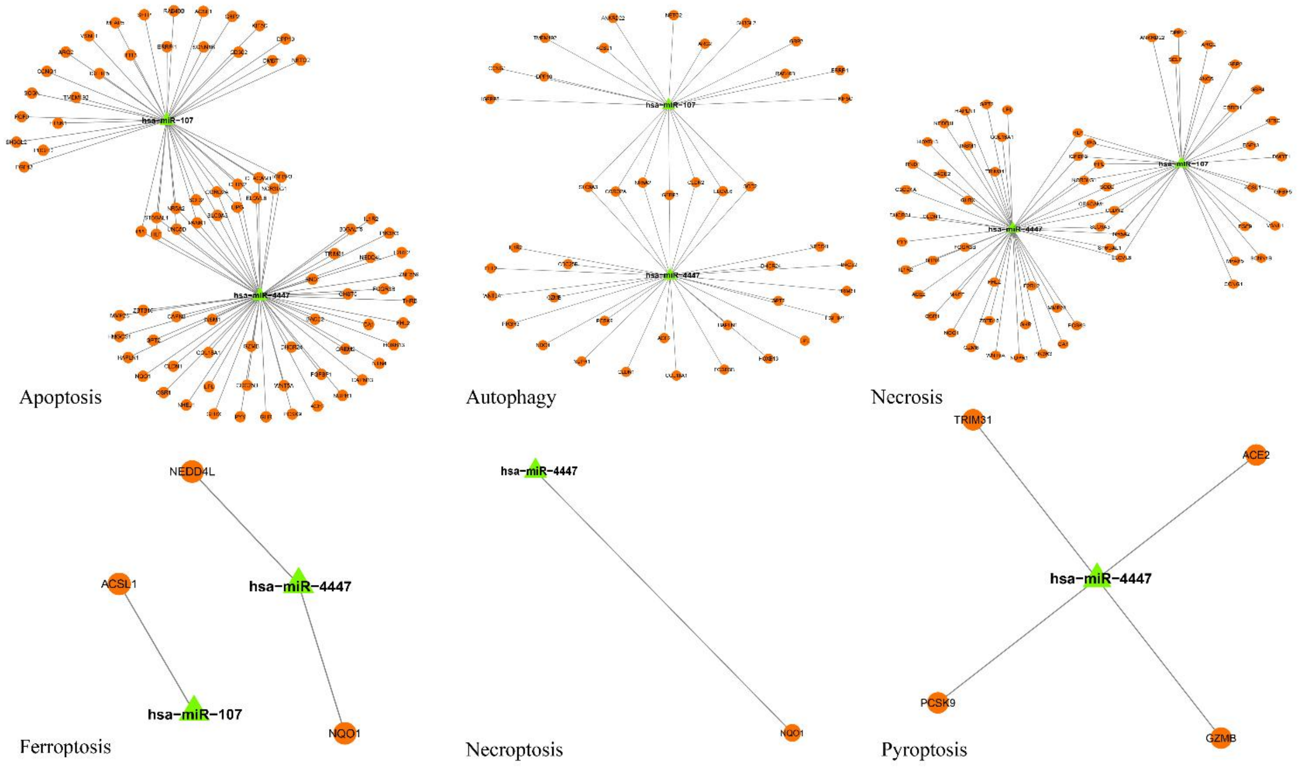

Supplementary Figure 1. Interaction network between genes of PCD related DEGs in GSE36807 dataset and its targeted miRNAs. Genes were colored in orange; miRNAs were colored in blue.

A

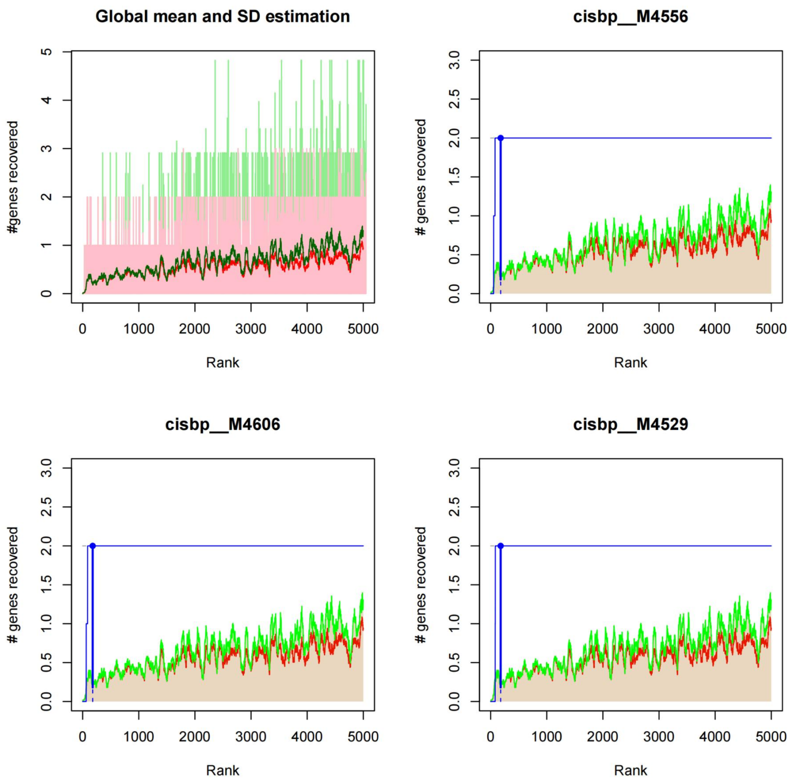

B

	logo	geneSet	motif	NES	AUC	TF_highConf	TF_lowConf	nEnrGenes	rankAtMax	enrichedGenes
	All	All	All	Al	Al	All	All	All	All	All
1		Key_Gene	cisbp__M4556	11.6	0.627	CEBPB (directAnnotation).	CEBPA; CEBPD; CEBPE; CEBPG; DBP; DDIT3; EP300; MYC (inferredBy_MotifSimilarity).	2	179	MMP1;SAA1
2		Key_Gene	cisbp__M4606	11.5	0.621	CEBPB (directAnnotation).	ATF4; CEBPA; CEBPD; CEBPE; CEBPG; DBP; DDIT3; EP300; HLF; PPARGC1A (inferredBy_MotifSimilarity).	2	179	MMP1;SAA1
3		Key_Gene	cisbp__M4529	11.4	0.617	CEBPB (directAnnotation).	ATF4; CEBPA; CEBPD; CEBPE; CEBPG; EP300 (inferredBy_MotifSimilarity).	2	179	MMP1;SAA1
4		Key_Gene	cisbp__M2268	11.2	0.609	CEBPB (directAnnotation).	CEBPA; CEBPD; CEBPE; CEBPG; EP300; MYC (inferredBy_MotifSimilarity).	2	179	MMP1;SAA1
5		Key_Gene	cisbp__M1925	11.1	0.603	CEBPA (directAnnotation).	CEBPB; CEBPD; CEBPE; CEBPG; EP300 (inferredBy_MotifSimilarity).	2	179	MMP1;SAA1

Supplementary Figure 2. Enrichment analysis for transcription factors of hub genes. (A) The top three motifs with the highest area under the curve (AUC) values. The red line represents the average recovery curve for each motif, the green line denotes the mean + standard deviation, and the blue line represents the recovery curve for the specific motif. The point of maximum distance (mean + SD) between the motif's recovery curve (blue) and the average recovery curve (green) is used to indicate the highest enrichment level. The cisbp_M4556 motif showed significant enrichment in hub genes, specifically in MMP1 and SAA1. (B)Demonstration of the highest motif enrichment values, including the standardized enrichment score (NES), AUC, and transcription factors annotated to each significant motif (TF_highConf).

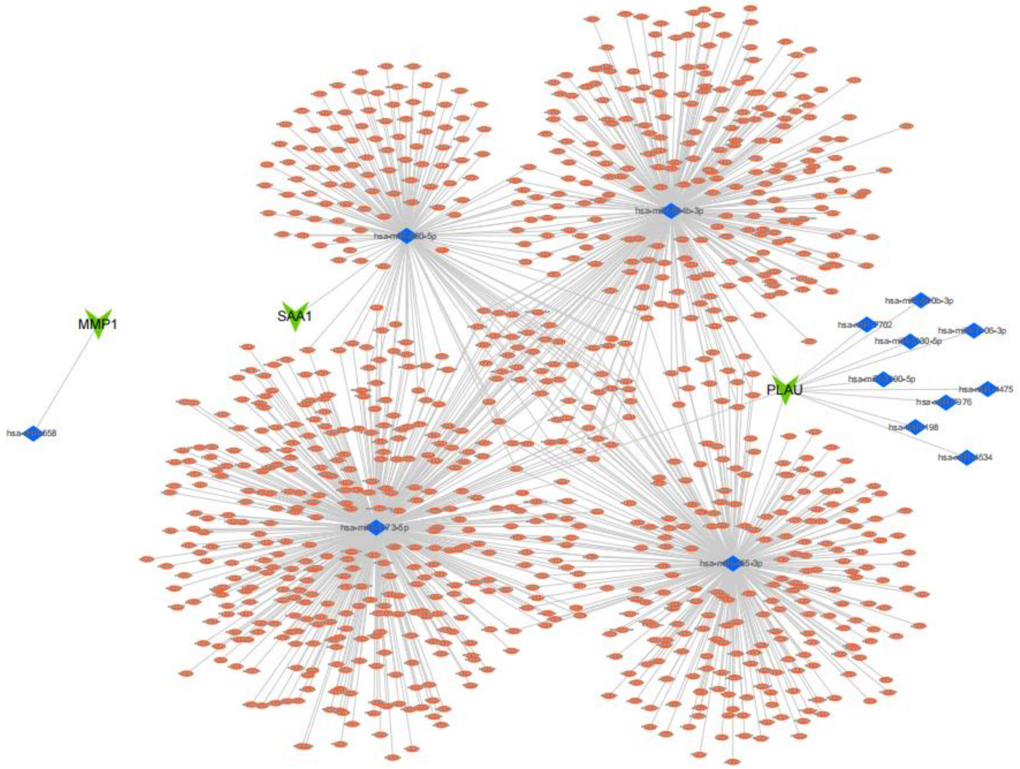

Supplementary Figure 3. Hub gene multi-factor regulatory network. Interaction network between MMP1, SAA1, and PLAU and its targeted miRNAs. Genes were colored in green, miRNAs were colored in blue, and the lncRNAs were colored in orange.

Supplementary Table 1. Model performance information

Model	Cutoff	AUC (95%CI)	Accuracy	Sensitive	Specificity	Positive Predictive Value	Negative Predictive Value	KAPPA
MMP1	1.695	0.825(0.659-0.990)	0.800	0.909	0.714	0.714	0.909	0.606
SAA1	1.763	0.753(0.555-0.951)	0.760	1.000	0.571	0.647	1.000	0.540
PLAU	1.743	0.890(0.766-1.000)	0.800	1.000	0.643	0.688	1.000	0.613

AUC; area under the curve
