# Supplementary material for: Identification of hub programmed cell death-related genes and immune infiltration in Crohn’s disease using bioinformatics
Source: Front Genet. 2024 Dec 18;15:1425062. doi: 10.3389/fgene.2024.1425062 (PMC11688285; doi:10.3389/fgene.2024.1425062)
Supplement: Supplementary file 1 [file DataSheet3.zip › Input data and script2/GSEA analysis/SAA1/plot_go/1.GSEA GO.pdf]

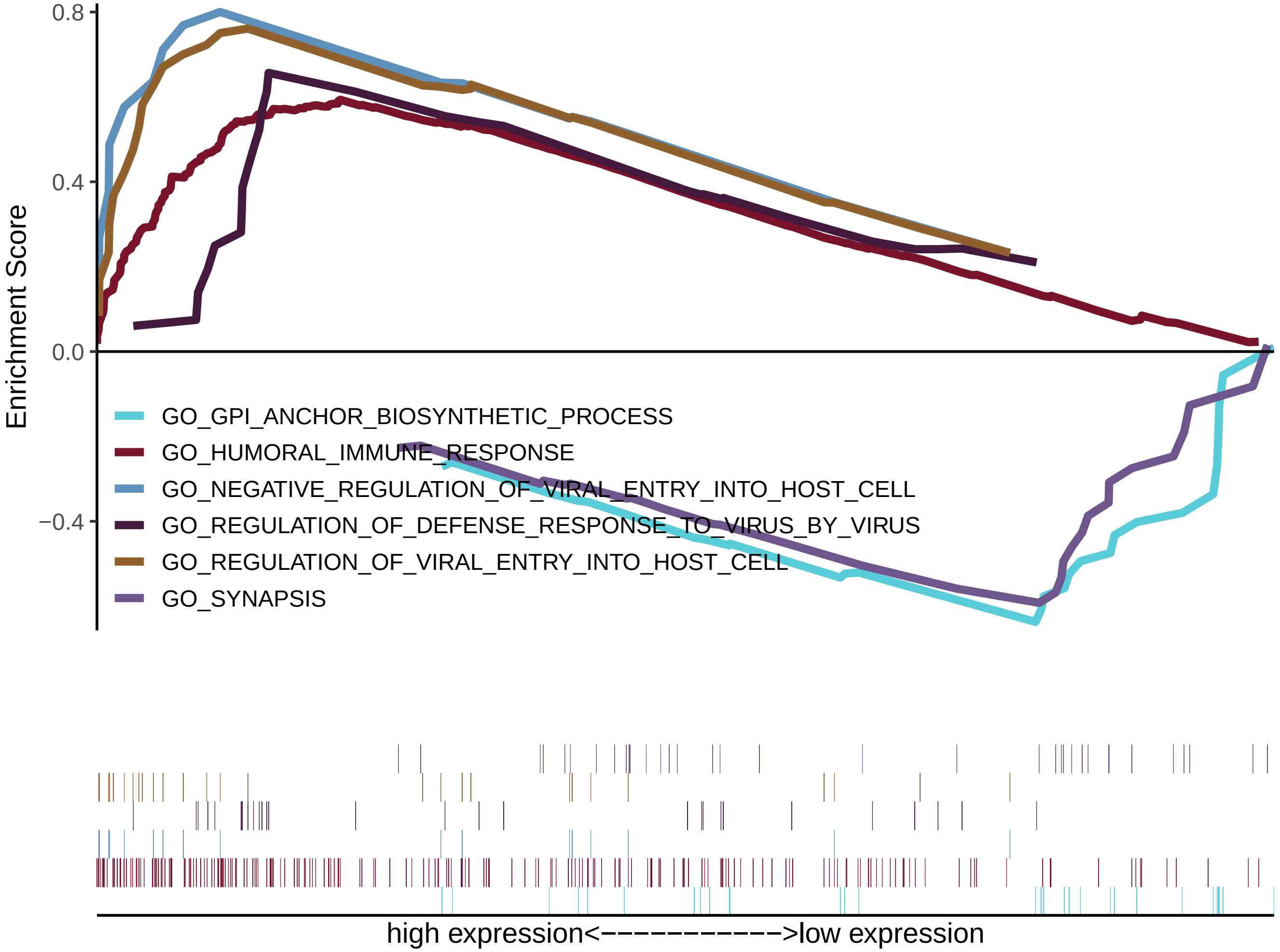

Enrichment Score
0.8
0.4
0.0
−0.4
GO_GPI_ANCHOR_BIOSYNTHETIC_PROCESS
GO_HUMORAL_IMMUNE_RESPONSE
GO_NEGATIVE_REGULATION_OF_VIRAL_ENTRY_INTO_HOST_CELL
GO_REGULATION_OF_DEFENSE_RESPONSE_TO_VIRUS_BY_VIRUS
GO_REGULATION_OF_VIRAL_ENTRY_INTO_HOST_CELL
GO_SYNAPSIS
high expression<−−−−−−−−−−−>low expression
