# Supplementary material for: Identification of hub programmed cell death-related genes and immune infiltration in Crohn’s disease using bioinformatics
Source: Front Genet. 2024 Dec 18;15:1425062. doi: 10.3389/fgene.2024.1425062 (PMC11688285; doi:10.3389/fgene.2024.1425062)
Supplement: Supplementary file 1 [file DataSheet3.zip › Input data and script2/GSEA analysis/SAA1/plot_kegg/2.GSEA KEGG.pdf]

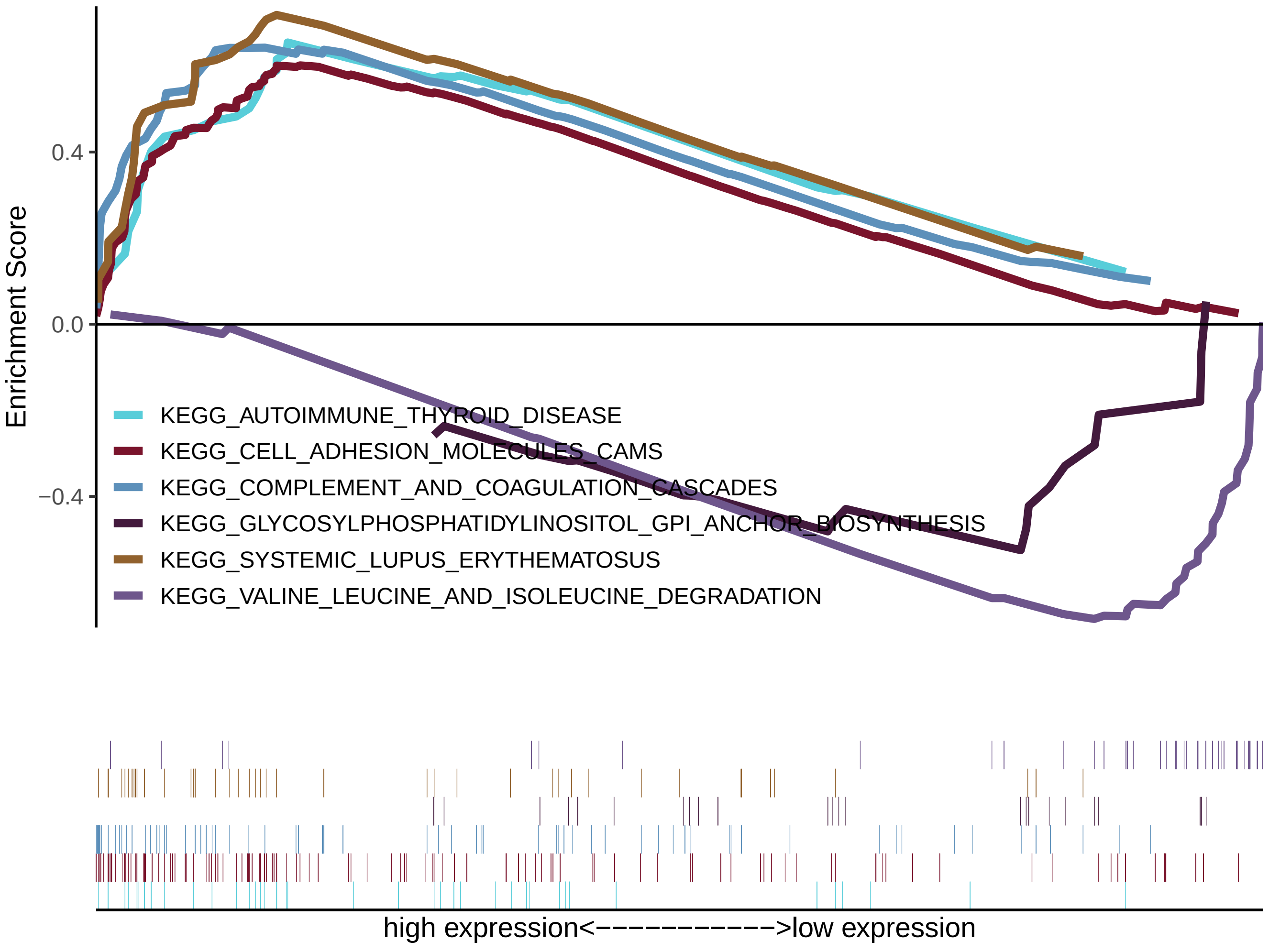
Enrichment Score
0.4
0.0
−0.4
KEGG_AUTOIMMUNE_THYROID_DISEASE
KEGG_CELL_ADHESION_MOLECULES_CAMS
KEGG_COMPLEMENT_AND_COAGULATION_CASCADES
KEGG_GLYCOSYLPHOSPHATIDYLINOSITOL_GPI_ANCHOR_BIOSYNTHESIS
KEGG_SYSTEMIC_LUPUS_ERYTHEMATOSUS
KEGG_VALINE_LEUCINE_AND_ISOLEUCINE_DEGRADATION
high expression<−−−−−−−−−−−−>low expression
