# Supplementary material for: Identification of hub programmed cell death-related genes and immune infiltration in Crohn’s disease using bioinformatics
Source: Front Genet. 2024 Dec 18;15:1425062. doi: 10.3389/fgene.2024.1425062 (PMC11688285; doi:10.3389/fgene.2024.1425062)
Supplement: Supplementary file 2 [file DataSheet4.zip › Input data and script3/ceRNAnet/Pyroptosis_gene/net1.pdf]

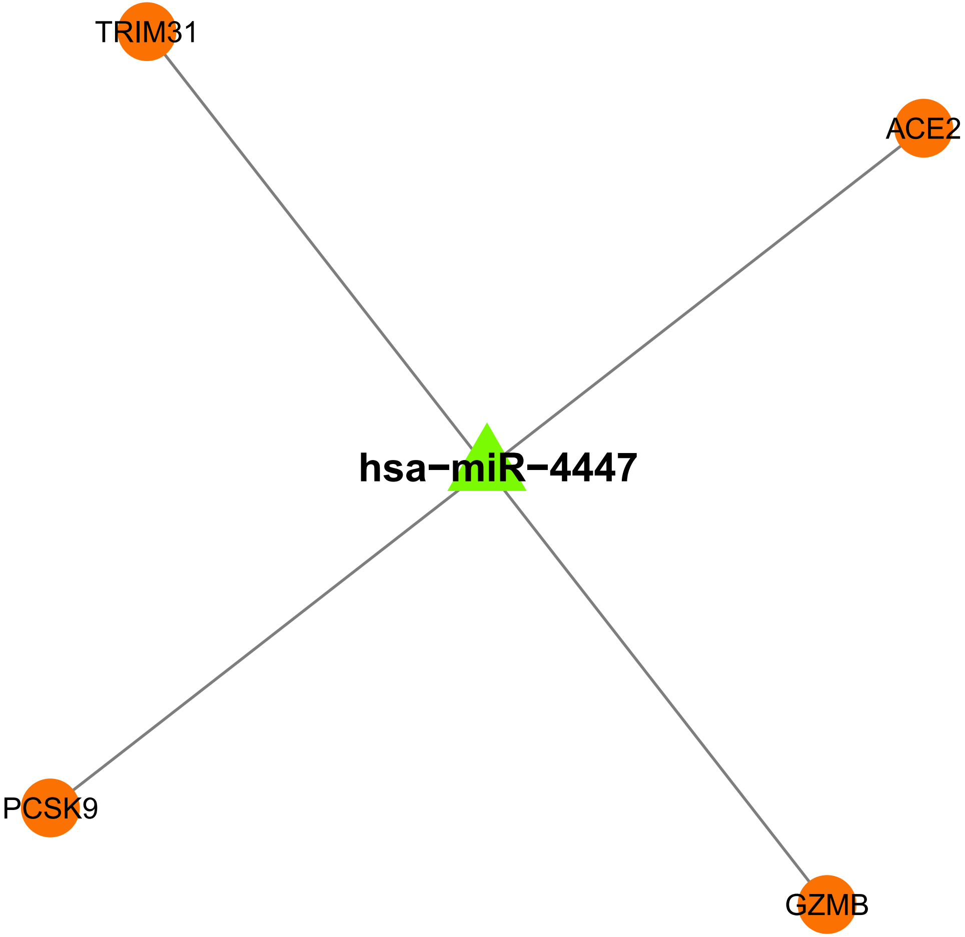
TRIM31
ACE2
hsa−miR−4447
PCSK9
GZMB
