# Supplementary material for: Identification of hub programmed cell death-related genes and immune infiltration in Crohn’s disease using bioinformatics
Source: Front Genet. 2024 Dec 18;15:1425062. doi: 10.3389/fgene.2024.1425062 (PMC11688285; doi:10.3389/fgene.2024.1425062)
Supplement: Supplementary file 2 [file DataSheet4.zip › Input data and script3/Xcell-Immune infiltration/correlation/correlation between xcell and expression of PLAU.pdf]

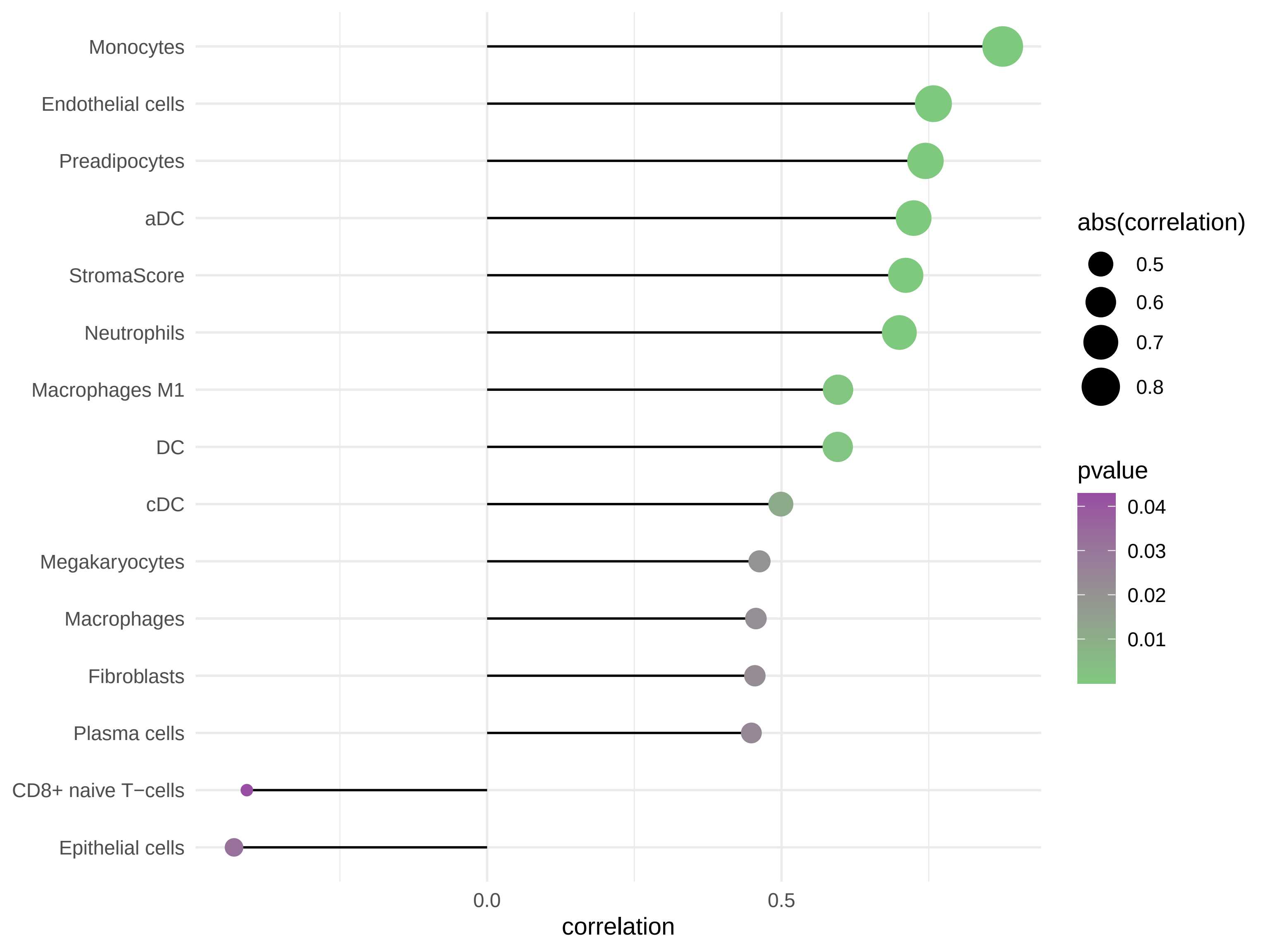

Monocytes
Endothelial cells
Preadipocytes
aDC
StromaScore
Neutrophils
Macrophages M1
DC
cDC
Megakaryocytes
Macrophages
Fibroblasts
Plasma cells
CD8+ naive T−cells
Epithelial cells
0.0
0.5
correlation
abs(correlation)
0.5
0.6
0.7
0.8
pvalue
0.04
0.03
0.02
0.01
