# Supplementary material for: Identification of hub programmed cell death-related genes and immune infiltration in Crohn’s disease using bioinformatics
Source: Front Genet. 2024 Dec 18;15:1425062. doi: 10.3389/fgene.2024.1425062 (PMC11688285; doi:10.3389/fgene.2024.1425062)
Supplement: Supplementary file 2 [file DataSheet4.zip › Input data and script3/Xcell-Immune infiltration/correlation/correlation between xcell and expression of SAA1.pdf]

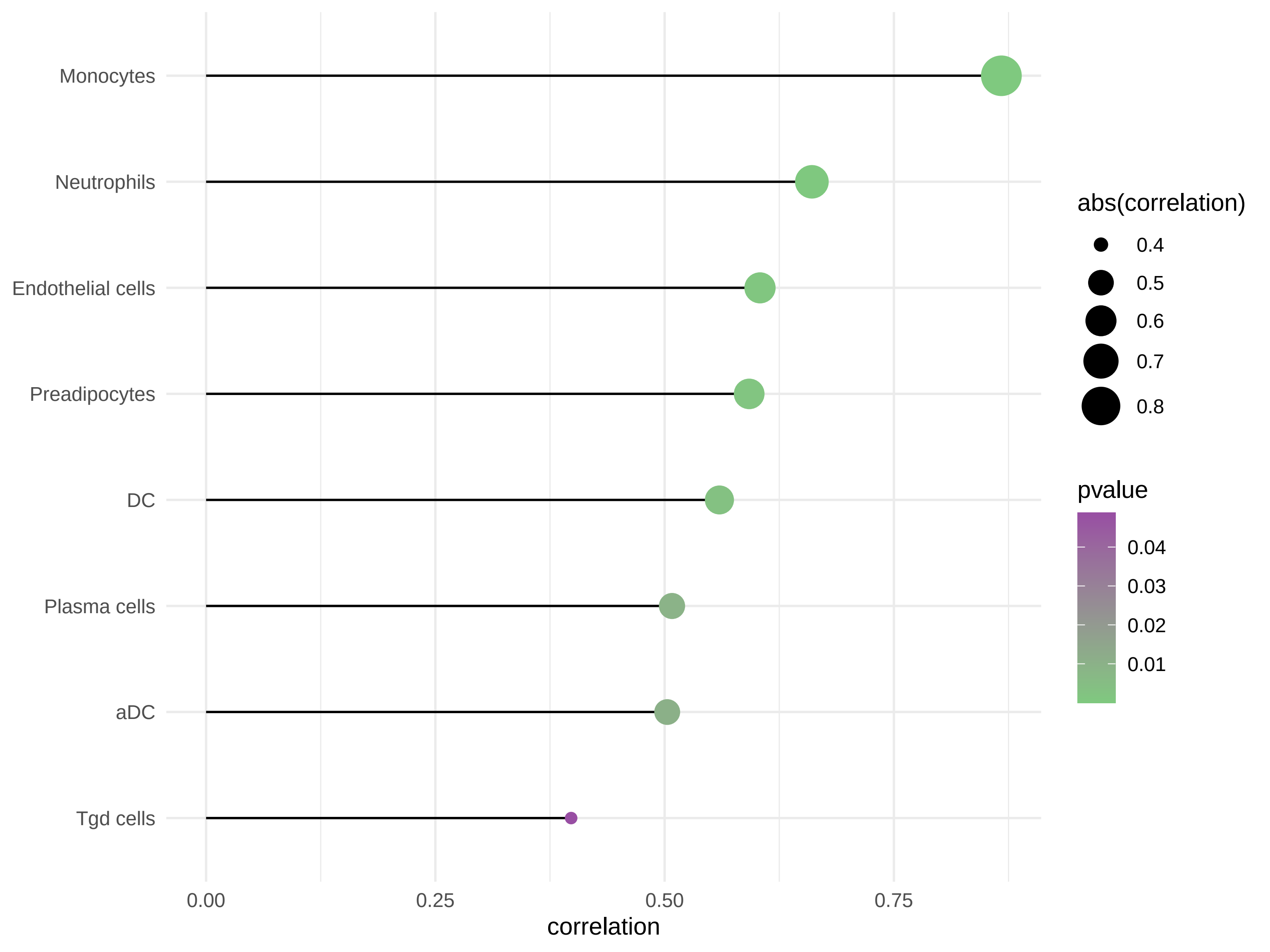
Monocytes
Neutrophils
Endothelial cells
Preadipocytes
DC
Plasma cells
aDC
Tgd cells
0.00
0.25
0.50
0.75
correlation
abs(correlation)
0.4
0.5
0.6
0.7
0.8
pvalue
0.04
0.03
0.02
0.01
